# Decarboxylative Palladium(II)-Catalyzed Synthesis of Aryl Amidines from Aryl Carboxylic Acids: Development and Mechanistic Investigation

**DOI:** 10.1002/chem.201301809

**Published:** 2013-08-28

**Authors:** Jonas Rydfjord, Fredrik Svensson, Alejandro Trejos, Per J R Sjöberg, Christian Sköld, Jonas Sävmarker, Luke R Odell, Mats Larhed

**Affiliations:** [a] Organic Pharmaceutical Chemistry, Department of Medicinal Chemistry, Uppsala UniversityBox-574, 751 23 Uppsala (Sweden); [b] Department of Chemistry–BMC, Uppsala UniversityBMC, Box-599, 751 23 Uppsala (Sweden)

**Keywords:** decarboxylation, density functional calculations, mass spectrometry, microwave chemistry, palladium

## Abstract

A fast and convenient synthesis of aryl amidines starting from carboxylic acids and cyanamides is reported. The reaction was achieved by palladium(II)-catalysis in a one-step microwave protocol using [Pd(O_2_CCF_3_)_2_], 6-methyl-2,2′-bipyridyl and trifluoroacetic acid (TFA) in *N*-methylpyrrolidinone (NMP), providing the corresponding aryl amidines in moderate to excellent yields. The protocol is very robust with regards to the cyanamide coupling partner but requires electron-rich *ortho*-substituted aryl carboxylic acids. Mechanistic insight was provided by a DFT investigation and direct ESI-MS studies of the reaction. The results of the DFT study correlated well with the experimental findings and, together with the ESI-MS study, support the suggested mechanism. Furthermore, a scale-out (scale-up) was performed with a non-resonant microwave continuous-flow system, achieving a maximum throughput of 11 mmol h^−1^ by using a glass reactor with an inner diameter of 3 mm at a flow rate of 1 mL min^−1^.

## Introduction

During the past few years aryl carboxylic acids have emerged as synthetically useful aryl-metal precursors.^1^, ^2^ The formation of the aryl-metal species occurs through a metal-mediated decarboxylative process, which, although long known,^3^ has only recently captured the attention of synthetic chemists. This has led to the development of numerous decarboxylative palladium(II)-catalyzed cross-coupling,^1^, ^4^–^9^ 1,2-addition^10^, ^11^ and Heck-type reactions.^12^, ^13^ Carboxylic acids are attractive coupling partners due to their low cost, wide-spread availability, inert nature and most importantly, CO_2_ is the only byproduct produced in the formation of the arylpalladium species. Aryl carboxylic acids also present some distinct advantages over organoboron derivatives, the most commonly employed substrates in palladium(II)-catalyzed cross-couplings, as they offer a more environmentally benign and less-toxic class of arylating agents.^14^ Additionally, sterically congested aryl carboxylic acids are often excellent substrates in palladium-catalyzed transformations as *ortho*-substituents are known to facilitate the decarboxylation process.^15^

Compounds with the amidine motif are of considerable interest in drug discovery and have been indicated as potential agents for the treatment of Alzheimer’s disease,^16^ malaria,^17^ and as inhibitors of acid-sensing ion channels,^18^ platelet aggregation^19^ and serine proteases.^20^ Amidines are also useful precursors for the synthesis of a wide variety of heterocyclic ring systems such as quinazolines,^21^, ^22^ quinazolinones,^23^ pyrimidines,^24^, ^25^ triazoles,^26^ and benzimidazoles.^27^ Amidines are traditionally prepared by the nucleophilic addition of an amine to a nitrile^28^ and they are also accessible by nucleophilic substitution of thioamides and imidates.^29^ More recent methodologies include Pd^0^-catalyzed three-component methods,^30^–^33^ addition of alkylchloroaluminum amides to nitriles,^34^ aryne insertion into thioureas^35^ and ytterbium-catalyzed addition of amines to nitriles.^36^

**Scheme 1:**
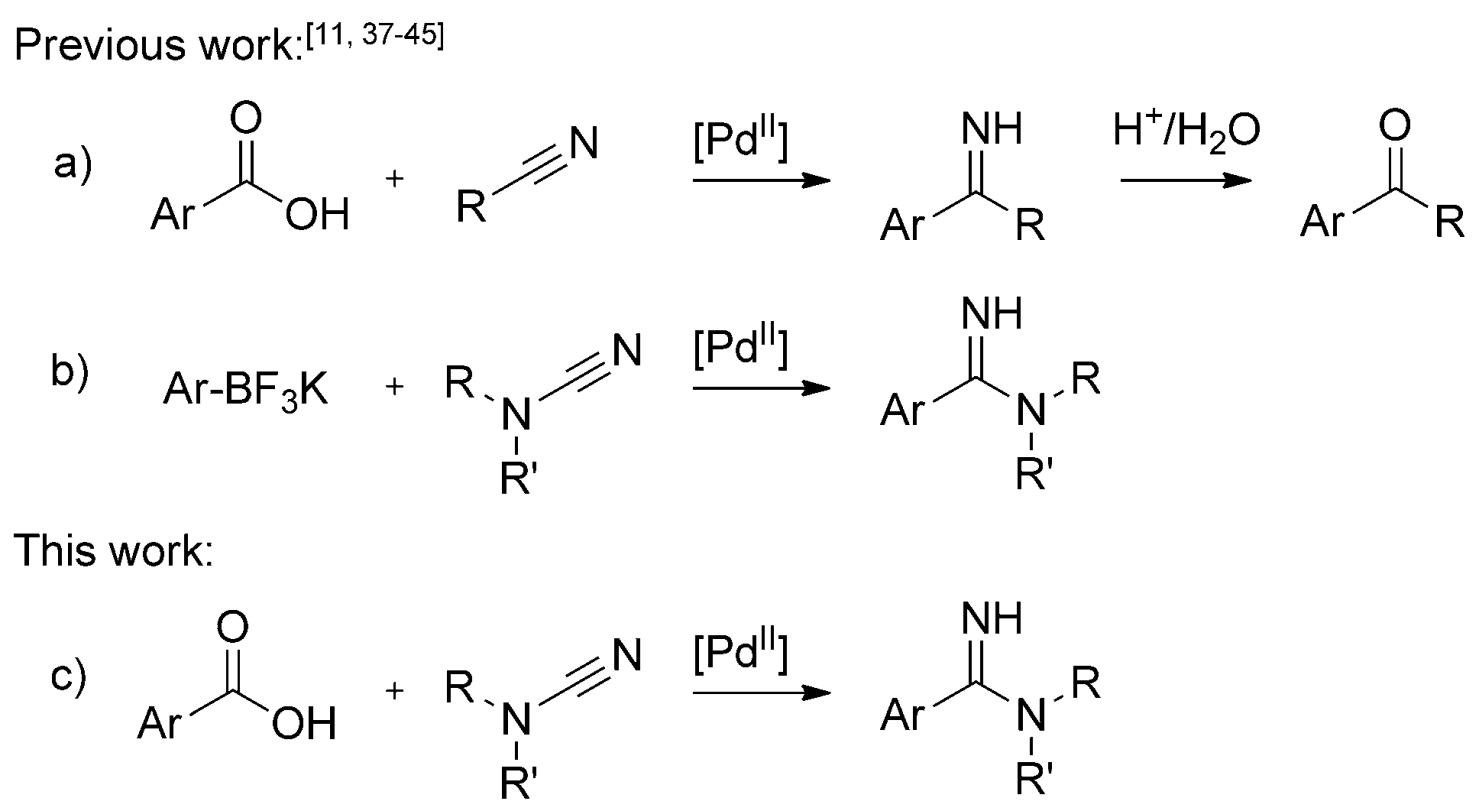
Palladium(II)-catalyzed protocols for the 1,2-carbopalladation of nitrile derivatives.

We^11^, ^37^ and others^38^–^44^ have previously developed palladium(II)-catalyzed protocols for the 1,2-carbopalladation of nitrile derivatives, generating aryl ketones through a ketimine intermediate (Scheme 1 a). Recently, we demonstrated that aryl amidines could also be conveniently accessed through a palladium(II)-catalyzed 1,2-carbopalladation of cyanamides with aryltrifluoroborates (Scheme 1 b).^45^ To further extend the utility of this direct amidine synthesis we decided to investigate the viability of carboxylic acids as the arylpalladium precursor. Herein, we present a fast and convenient protocol for the preparation of aryl amidines from aryl carboxylic acids and cyanamides (Scheme 1 c).

## Results and Discussion

As a starting point for our investigation we conducted a solvent screen by using our previously developed catalytic system for aryl amidine synthesis from aryl trifluoroborates ([Pd(O_2_CCF_3_)_2_], 6-methyl-2,2′-bipyridyl (**4**) as the ligand and trifluoroacetic acid (TFA) as the proton donor).^45^ 2,4,6-Trimethoxybenzoic acid (**1 a**) and *N*-cyanopiperidine (**2 a**) were chosen as model substrates and the catalyst and ligand loadings were set at 2 and 3 %, respectively. The reactions were then microwave (MW) heated^46^ at 120 °C for 30 min using the appropriate solvent and 1 equivalent of TFA in sealed vessels. As can be seen from Table 1, polar aprotic solvents performed best, with *N*-methylpyrrolidinone (NMP) giving 96 % yield of the isolated product (Table 1, entry 5), whereas dimethylformamide (DMF) and dimethylacetamide (DMA) gave slightly lower yields of 88 and 92 % (Table 1, entries 3 and 4), respectively. The use of dioxane gave the product in a reduced yield of 68 % (Table 1, entry 1), whereas toluene afforded less than 15 % yield (Table 1, entry 2).

**Table 1:** Selecting the optimal reaction conditions.
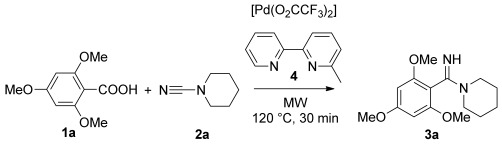

Entry	Pd [%]	Solvent	Yield [%][a]
1	2	dioxane	68
2	2	toluene	<15[b]
3	2	DMF	88
4	2	DMA	92
5	2	NMP	96
6	4	NMP	98
7	8	NMP	96
8	1	NMP	94
9[c]	2	NMP	92
10[d]	2	NMP	42
11[e]	0	NMP	not detected
12[f]	2	NMP	<15[b]
13[g]	2	NMP	92 (11 mmol h^−1^)

[a] Yield of the isolated product (>95 % pure by ^1^H NMR spectroscopic analysis). Reaction conditions: [Pd(O_2_CCF_3_)_2_], 6-methyl-2,2′-bipyridyl (**4**; **4**/[Pd(O_2_CCF_3_)_2_], 1.5:1), TFA (1 mmol), benzoic acid **1 a** (1.1 mmol), cyanamide **2 a** (1 mmol) and solvent (3 mL), were MW heated in a sealed vial at 120 °C for 30 min;

[b] yield determined by ^1^H NMR spectroscopy;

[c] 1.0 mmol of **1 a**;

[d] no ligand (**4**) added;

[e] no [Pd(O_2_CCF_3_)_2_] or ligand (**4**);

[f] no TFA;

[g] continuous-flow scale-out example, 1 mL min^−1^ of the reaction mixture corresponding to 1 min in the heated zone (temperature set at 140 °C, 0.2 m in NMP of yield-determining **2 a**, 1.1 equiv of **1 a**), the yield is based on the work-up of an aliquot of 5 mL with a theoretical yield of 1 mmol of **3 a**.

Having identified NMP as the solvent of choice we next set about exploring the remaining parameters (temperature, time, palladium loading, ligand loading, and stoichiometry). Table 1 reveals that the reaction between **1 a** and **2 a** is a robust process as the majority of changes had only a minor influence on the reaction outcome. Control experiments performed without the addition of a ligand (Table 1, entry 10), [Pd(O_2_CCF_3_)_2_] (Table 1, entry 11) and TFA (Table 1, entry 12) demonstrated that each component is crucial for a productive reaction. Interestingly, omission of the ligand was not deleterious and the reaction gave a moderate yield of 42 %. Based on these results we chose our original NMP conditions for further investigation (Table 1, entry 5).

Next, the scope of the addition reaction with regards to the cyanamide partner was investigated and the results are presented in Table 2. Cyanamide **2 b** furnished the primary aryl amidine **3 b** in 76 % yield (Table 2, entry 2) and *tert*-butyl cyanamide **2 c** gave the corresponding aryl amidine **3 c** isolated in an excellent yield of 90 % (Table 2, entry 3) showing that *N*,*N*-disubstitution is not required. Dimethyl and diethyl cyanamide (**2 d** and **2 e**) also performed well, providing 93 and 98 % of **3 d** and **3 e**, respectively (Table 2, entries 4 and 5). The bulkier cyanamide derivative **2 f** furnished a lower yield of **3 f** (64 %, Table 2, entry 6), presumably due to unfavorable steric effects. Cyclic cyanamides **2 g** and **2 h** were also effective substrates, affording **3 g** and **3 h** in 93 and 74 % yield, respectively (Table 2, entries 7 and 8). Dibenzyl derivative **2 i** reacted smoothly, providing 68 % of **3 i** (Table 2, entry 9). The reaction was unsuccessful with carboxybenzyl-protected substrate **2 j** and only trace amounts of the hydrolyzed product **3 b** were detected by HPLC-MS.

**Table 2:** Scope of 2,4,6-trimethoxybenzoic acid with different cyanamides.
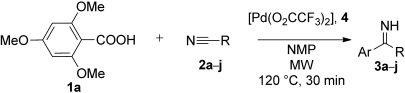

Entry	R	Product	Yield [%][a]
1	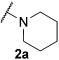	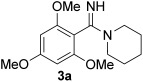	96
2	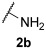	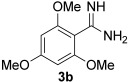	76
3	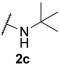	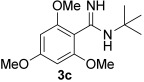	90
4	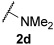	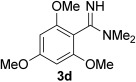	93
5	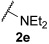	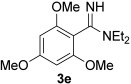	98
6	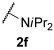	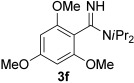	64
7	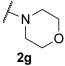	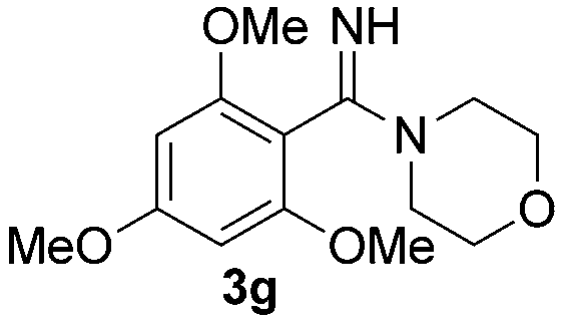	93
8	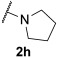	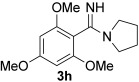	74
9	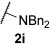	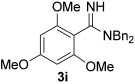	68
10	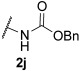	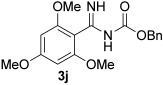	not observed

[a] Yield of the isolated product (>95 % pure by ^1^H NMR spectroscopic analysis). Reaction conditions: [Pd(O_2_CCF_3_)_2_] (0.02 mmol), **4** (0.03 mmol), TFA (1 mmol), benzoic acid **1 a** (1.1 mmol), cyanamides **2 a–j** (1 mmol), and NMP (3 mL) were MW heated in a sealed vial at 120 °C for 30 min.

Next, we extended the scope of the carboxylic acid substrate to include electron-rich di- or trimethoxy-substituted benzoic acid derivatives **1 b–d**. Unfortunately, these substrates proved to be much less reactive than **1 a**, requiring both elevated temperature (140 °C) and an extended reaction time (60 min) to achieve full conversion. Substrate **1 b** provided a good yield (65 %) of the corresponding amidine **3 k** (Table 3, entry 1), whereas **1 c** and **1 d** furnished yields of only 9 and 8 %, respectively (Table 3, entries 2 and 3). Several other carboxylic acid derivatives known to undergo decarboxylative coupling reactions were also tested,^47^ however, only traces of the desired products were detected by HPLC-MS. Finally, the addition of silver or copper salts were evaluated in an attempt to promote decarboxylation,^1^ and, disappointingly, these additives did not improve the outcome of the reaction.^48^

**Table 3:** Scope of various carboxylic acid derivatives with different cyanamides.
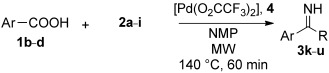

Entry	Ar	Cyanamide	Product	Yield [%][a]
1	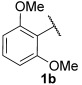	**2a**	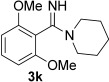	65
2	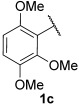	**2 a**	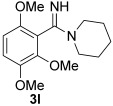	9[b]
3	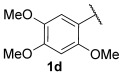	**2 a**	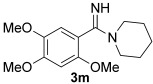	8[b]
4	**1 b**	**2 b**	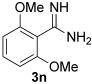	36[b]
5	**1 b**	**2 c**	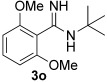	52
6	**1 b**	**2 d**	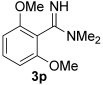	37[b]
7	**1 b**	**2 e**	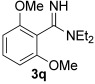	48
8	**1 b**	**2 f**	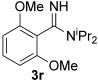	74
9	**1 b**	**2 g**	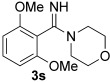	63
10	**1 b**	**2 h**	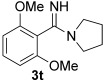	32[b]
11	**1 b**	**2 i**	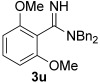	74

[a] Yield of the isolated product (>95 % pure by ^1^H NMR spectroscopic analysis). Reaction conditions: [Pd(O_2_CCF_3_)_2_] (0.08 mmol), **4** (0.12 mmol), TFA (1 mmol), benzoic acids **1 b–d** (1.1 mmol), cyanamides **2 a–i** (1 mmol), and NMP (3 mL) were MW heated in a sealed vial at 140 °C for 60 min;

[b] purified by using preparative HPLC.

The scope of the cyanamides was also investigated further with carboxylic acid **1 b** (Table 3). The substrate was effectively reacted with various cyanamides, however, the yields were in general lower than that obtained with **1 a**, with the exception of cyanamides **2 f** and **2 i**, both affording 74 % yield of the corresponding products **3 r** and **3 u** (Table 3, entries 8 and 11; compare Table 2, entries 6 and 9).

**Figure 1:**
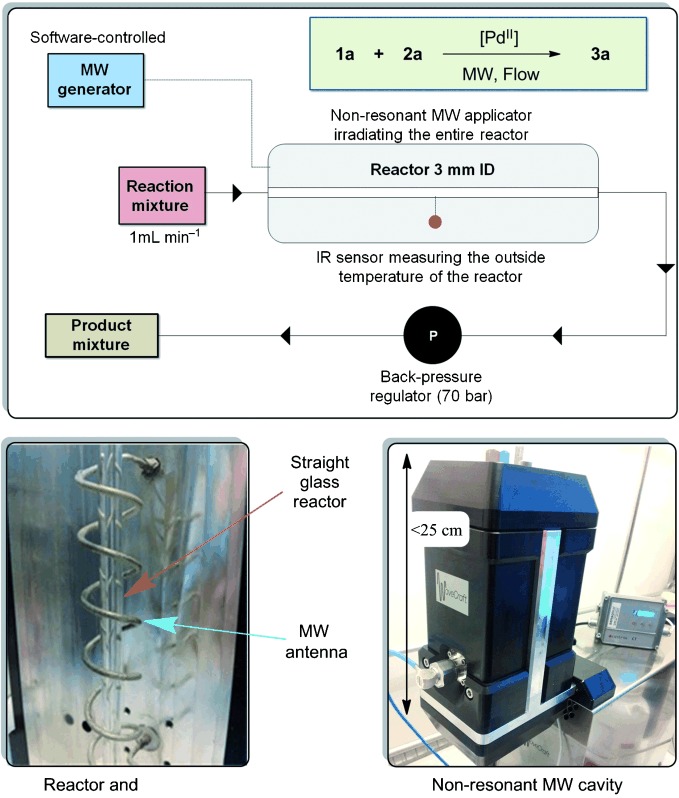
Top: schematic illustration of continuous-flow setup utilizing a non-resonant MW-cavity for heating of the reaction mixture. Bottom left: inside the reactor cavity, the glass reactor and MW antenna are displayed. Bottom right: closed reactor cavity.

Finally, a scale-out (scale-up) of the reaction (Figure 1), utilizing a non-resonant microwave system for continuous-flow organic synthesis,^49^–^52^ was performed. With this new equipment for direct scale-out we were able to achieve a high throughput of 11 mmol h^−1^ (corresponding to 3 g h^−1^). The experiment used a stock solution with 0.2 m of yield-determining **2 a**, compound **1 a** (1.1 equiv), along with [Pd(O_2_CCF_3_)_2_] (2 %), compound **4** (3 %), and TFA (1 equiv) in *N*-methylpyrrolidinone (NMP). After a minor adjustment of the reaction conditions, we achieved an excellent yield of 92 % of **3 a** (Table 1, entry 13) at 1 mL min^−1^ and 120 °C, which corresponds to 1 min in the heated zone.

The reaction of **1 a** proceeds smoothly with a range of different cyanamide derivatives, containing bulky groups, small groups, and unsubstituted cyanamide. However, the carboxylic acid substrate scope is limited and requires electron-rich *ortho*-substituted aryl carboxylic acids. Somewhat disappointed with this narrow scope we proceeded to investigate the reaction mechanism by using density functional theory (DFT) calculations to better understand our experimental findings.

**Figure 2:**
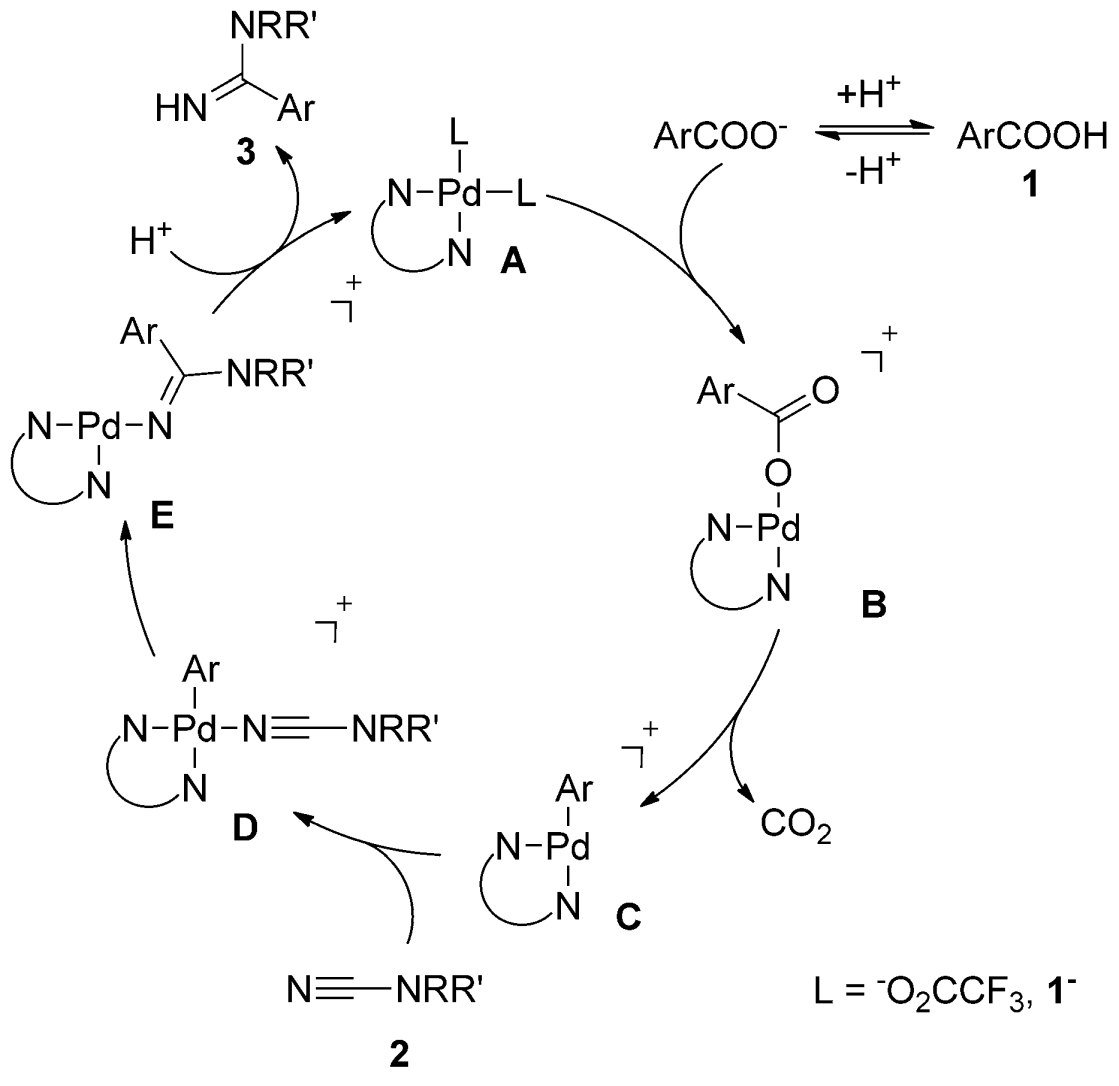
Proposed catalytic cycle as adapted from Lindh et al.^11^

**Figure 3:**
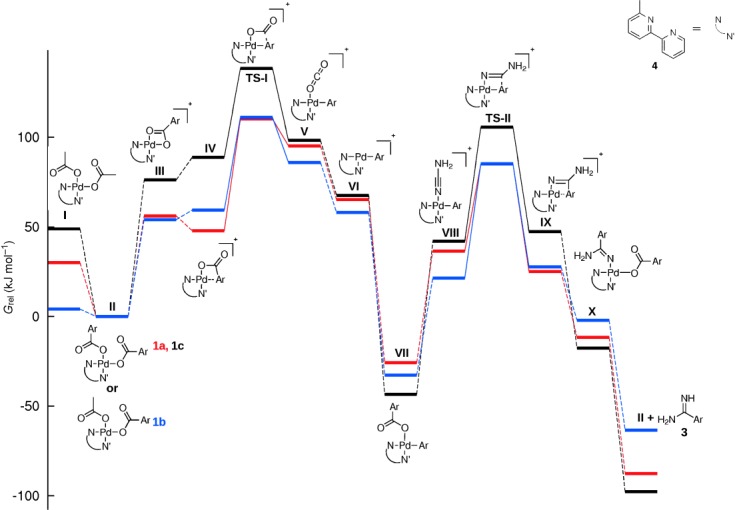
Calculated Gibbs energy profile of the reaction of carboxylic acids 1 a (red), 1 b (blue), and 1 c (black) with cyanamide 2 b to give product 3. Full lines indicate that the complexes are verified to be directly connected. For 1 a and 1 c, complex II has two coordinated aryl acids, whereas for 1 b it has one acid and one acetate.

Previously we have investigated the reaction path and mechanism for the 1,2-carbopalladation of nitriles with aryl carboxylic acids.^11^, ^53^ Based on our results from the nitrile study^11^ we suggest that amidine formation occurs through the catalytic pathway outlined in Figure 2. Starting with the ligand chelated Pd^II^-complex **A**, coordination of the aryl carboxylic acid **1** generates complex **B**. Next, decarboxylation occurs to form arylpalladium intermediate **C**, followed by coordination of the cyanamide to form complex **D**. 1,2-Carbopalladation of the cyanamide generates complex **E**, followed by protonation to afford the free amidine product **3** and a catalytically active Pd^II^-species. DFT calculations were then performed, based on the proposed mechanism in Figure 2. To shed light on the narrow substrate scope, the calculations focused on comparing the energy profiles for the most electron-rich and the least electron-rich benzoic acid analogues in the series, **1 a** and **1 c**, respectively. Compound **1 b** was also included to represent a benzoic acid with electronic properties in between **1 a** and **1 c**. Only the di-*ortho*-substituted carboxylic acids were included to avoid having to account for the *ortho*-effect when interpreting the results. In the calculations [Pd(OAc)_2_]^54^ was used as the palladium source, compound **4** as the ligand, and **2 b** was used as cyanamide. The results of the calculations are shown in Figure 3 and relative energies for all complexes are provided in the Supporting Information.

The calculations start with the diacetate-chelated species **I** with an associated ligand. Exchange of one acetate for **1 b** or both acetates for **1 a** or **1 c** gave intermediate **II**, which was identified as the lowest-energy complex prior to decarboxylation. Thus, complex **II** was chosen as the starting point of the catalytic cycle and the energies of all catalytic intermediates are reported relative to this. To proceed with decarboxylation, one coordination site needs to be made available. Dissociation of one anionic ligand to the cationic Pd complex **III** was associated with a significant increase in energy, approximately half of the required energy for the decarboxylation process, which may partly explain the benefits of a polar solvent reaction medium in the reaction. A change in binding mode of **1** giving organopalladium cation **IV** provides a suitable arrangement for decarboxylation over transition state **TS-I**. The DFT calculations show that the required free-energy of decarboxylation is 110.3 kJ mol^−1^ for **1 a**, compared with 138.5 kJ mol^−1^ for **1 c**, which suggests that decarboxylation of **1 a** is significantly more facile than for **1 c**. From the formed arylpalladium complex **V** dissociation of carbon dioxide gives σ-complex **VI**. Association of **1** to this complex furnishes the most stable intermediate **VII** prior to the 1,2-carbopalladation step. To initiate 1,2-carbopalladation, compound **1** was exchanged with **2 b** to give the ionic species **VIII**. As in the case for decarboxylation, charge separation was calculated to account for approximately half the required energy for the 1,2-carbopalladation process. From **VIII** 1,2-carbopalladation via transition state **TS-II** leads to the desired aryl amidine **IX**. When comparing the energy profiles of **1 a** and **1 c**, the former has a required free-energy of 110.8 kJ mol^−1^ for 1,2-carbopalladation, which is substantially lower than the 149.1 kJ mol^−1^ calculated for 1,2-carbopalladation using **1 c**. Association of **1** gives the neutral complex **X** and a subsequent product release by replacement of with **1**, or in the case of **1 b**, acetate, regenerates complex **II** and gives an overall exergonic reaction.

Overall, the reaction profiles support a substantially faster reaction rate for **1 a** compared with **1 c** and could explain the more favorable experimental outcome of this substrate. Comparison of the Gibbs energy profiles employing **1 a** and **1 b**, shows a much more subtle difference between these two analogues. Decarboxylation proceeds with very similar energy requirements, 110.3 and 111.1 kJ mol^−1^, respectively; however, the 1,2-carbopalladation step for **1 a** is slightly more favored (110.8 kJ mol^−1^) compared with **1 b** (117.9 kJ mol^−1^). For all three benzoic acid analogues examined, the 1,2-carbopalladation was found to be the rate-determining step. These calculations, together with the experimental results, support the requirement for an electron-rich benzoic acid analogue in the current reaction.

**Figure 4:**
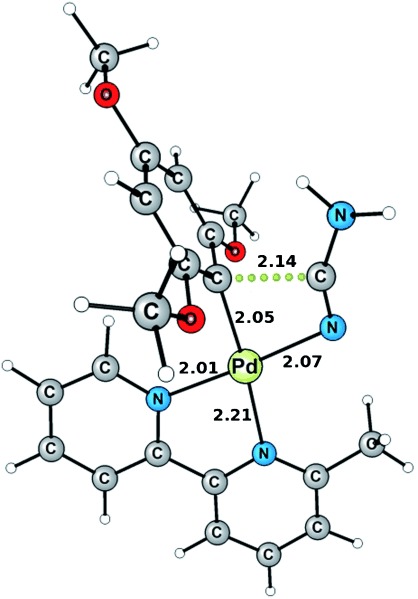
Optimized geometry for the 1,2-carbopalladation transition state TS-II. Bond lengths [Å] between Pd and coordinated atoms as well as the newly forming C–C bond are presented.

The overall reaction path is highly similar to the corresponding decarboxylative addition of benzoic acid analogues to acetonitrile.^53^ The decarboxylation process is identical and 1,2-carbopalladation using cyanamide is calculated to proceed in an analogous fashion compared with the reaction using acetonitrile. The 1,2-carbopalladation transition state is presented in Figure 4. In the previously reported investigation of nitriles, it was established that a more polar reaction media would be favorable.^53^ This was experimentally verified by using an increased amount of water, which also was needed for the hydrolysis step. The same conclusion should be valid in the present study and a more polar reaction media should reduce the energy cost for charge separation. This protocol is, however, incompatible with water and to some extent alcohols due to a competing urea byproduct formation. In addition, it was observed in the previous investigation that a significant byproduct when including water in the reaction is the decarboxylated aryl product, which would also be a source for the decreased yield in the reaction.^53^

To further validate and support the proposed mechanism and the assumptions made for the DFT calculations we performed a mass spectrometry (MS) study by using electrospray ionization (ESI). ESI is considered a soft mass spectrometric ionization technique and is therefore a valuable tool for analyzing sensitive organometallic species.^55^, ^56^ In addition, this technique also allows for the identification of palladium complexes present in an ongoing reaction. The reaction samples were removed for ESI-MS analysis after approximately 30–70 % conversion. By monitoring the pressure developed during the reaction, due to the carbon dioxide liberation, we were able to abort the reaction once it was midway through. ESI-MS detection afforded several cationic complexes that were readily identified as single palladium species based on the isotopic pattern of palladium. However, to determine the composition of these palladium(II) complexes we performed additional studies by substituting one component at a time.

**Figure 5:**
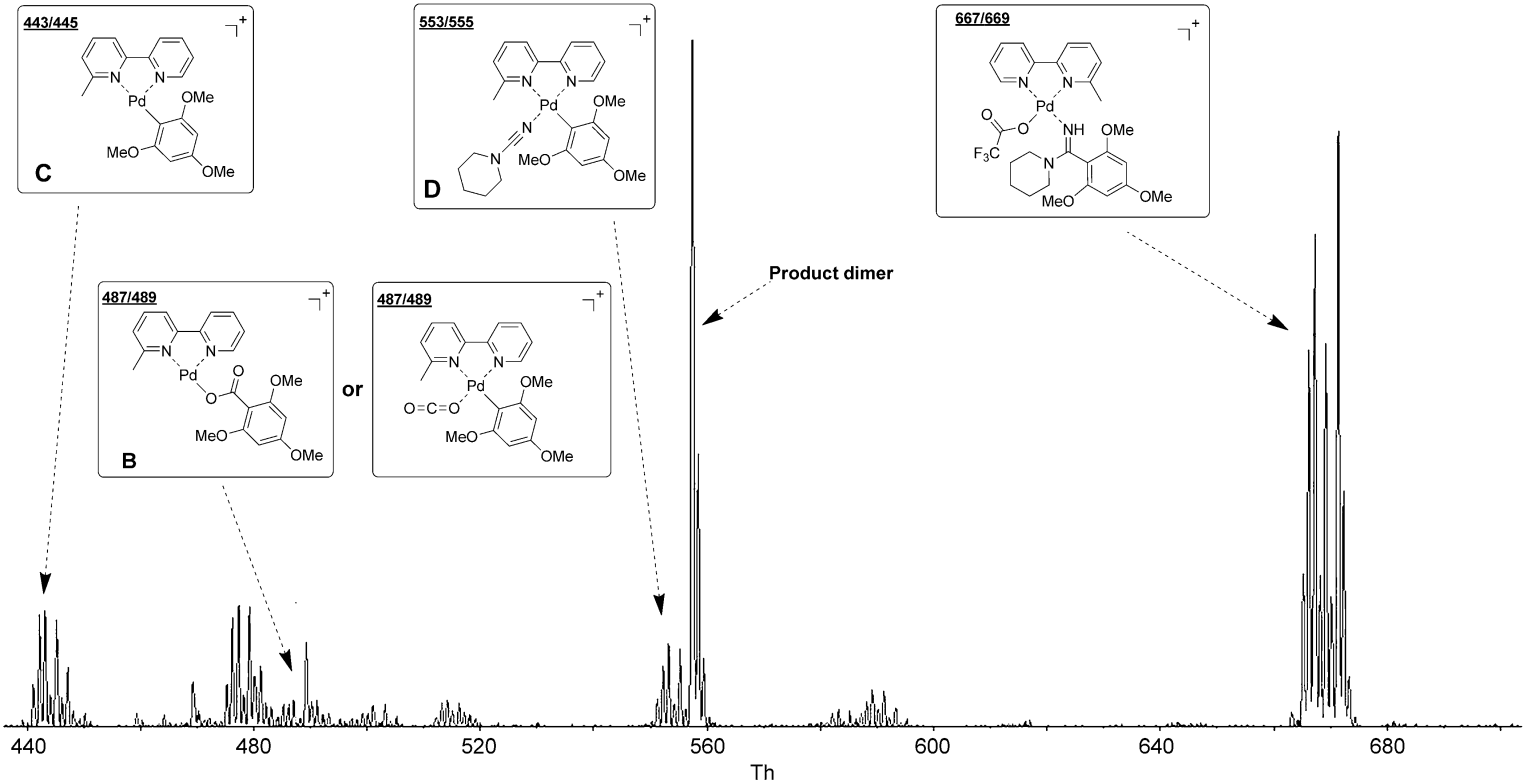
ESI-MS scan of reaction mixture at approximately 30–70 % conversion. Spectrum from reaction conditions: [Pd(O_2_CCF_3_)_2_] (0.02 mmol), compound 4 (0.03 mmol), TFA (1 mmol), benzoic acid 1 a (1.1 mmol), cyanamides 2 a (1 mmol), and NMP (3 mL). Also shown are the proposed Pd^II^ intermediates. The *cis* and *trans* geometries are based on the corresponding DFT calculated configurations with [Pd(OAc)_2_].

This strategy allowed us to identify four cationic species that were present in all of the reactions (Figure 5 and the Supporting Information). The suggested structures were assigned and classified according to the labels in the reaction pathway (Figure 2). Suggestions were supported by MS/MS studies along with neutral loss experiments. One interesting finding was that the intermediate with palladium, ligand, and carboxylic acid (**B** in Figure 2) produced a neutral loss of 44 Da, corresponding to the molecular mass of carbon dioxide. This finding suggests an alternate structure corresponding to the same molecular mass as **B**, in which the decarboxylation has already occurred and carbon dioxide is bonded to the palladium center (Figure 5). Alternatively, decarboxylation may occur in the mass spectrometer and the neutral loss of 44 Da is due to this process rather than an isolated complex. Examining the complexes that were consistently found in all reactions we were able to identify three out of the four suggested intermediates in the catalytic cycle (Figure 2). The complex prior to decarboxylation, **B**, was identified along with the arylpalladium complex **C** that is formed after decarboxylation. In addition, the σ-complex **D**, which is formed upon coordination of cyanamide to **C**, was identified by neutral loss experiments. Complex **E**, which is formed upon 1,2-carbopalladation, would have the same molecular mass as species **D**, but could not be conclusively identified. Taken together, the findings in this ESI-MS study are consistent with the suggested catalytic pathway.

## Conclusion

We have developed a direct palladium(II)-catalyzed protocol for the synthesis of aryl amidines from aryl carboxylic acids and cyanamides. The 1,2-addition reaction proceeds smoothly with good to excellent yields using different cyanamides and the preferred coupling partner, 2,4,6-trimethoxy benzoic acid **1 a**.

Presently, the scope is limited to aryl carboxylic acids containing *ortho*-methoxy substituents (**1 a–d**), which is in accordance with the known limitations associated with palladium(II)-catalyzed decarboxylation reactions.^1^ Furthermore, a continuous-flow scale-out was conducted with a purpose built non-resonant tubular MW reactor, achieving 11 mmol h^−1^ (92 % yield of the isolated product) after slight adjustment of the reaction conditions. DFT calculations and an accompanying ESI-MS study support the proposed reaction mechanism, and the improved outcome with di-*ortho*-methoxy-substituted benzoic acids.

## Experimental Section

**General procedure for the synthesis of aryl amidines 3 a–i**: [Pd(O_2_CCF_3_)_2_] (6.6 mg, 0.02 mmol), 6-methyl-2,2′-bipyridyl (5.1 mg, 0.03 mmol), and NMP (3.0 mL) were added to a 2–5 mL process vial and the mixture was stirred for 2 min before cyanamide, **2**, (1 mmol), benzoic acid, **1 a**, (1.1 mmol), and trifluoroacetic acid (114 mg, 1 mmol) were added. The vial was instantly capped under air and then heated by using microwave irradiation at 120 °C for 30 min. The reaction mixture was then diluted with aq. NaHCO_3_ (20 mL) and washed with diethyl ether (20 mL). The organic phase was further extracted with aq. NaHCO_3_ (2×20 mL). The combined aqueous phases were basified to pH≈14 by the addition of aq. NaOH and extracted with CH_2_Cl_2_ (3×30 mL). The combined organic phases were dried over a cotton plug and then concentrated and dried in vacuo to provide the pure isolated product in the yield stated in Tables 1, 2, and 3.

**Computational details**: All calculations were performed by using Jaguar version 7.8^57^ with the B3LYP^58^, ^59^ functional employing the LACVP* basis set.^60^ The complexes were optimized in the gas phase and the optimized structures were subsequently subjected to a single-point energy calculation using the PBF solvation model^61^, ^62^ with parameters suitable for NMP (dielectric constant, *ε*=32.2 and probe radius=2.67). The dispersion correction was applied by using the DFT-D3 program.^63^ Vibrational analysis was performed on the optimized geometry in the gas phase and the Gibbs free-energy in solvent was calculated by adding the gas phase thermodynamic contribution from the vibrational analysis at 373.15 K, including zero-point energy, and the dispersion correction to the single-point solution phase energy.

The presented transition states are shown to be connected to the presented proceeding and preceding energy minima, respectively. The presented transition states and stationary minima are verified to have one and zero imaginary frequencies, respectively.

## Notes

As a service to our authors and readers, this journal provides supporting information supplied by the authors. Such materials are peer reviewed and may be re-organized for online delivery, but are not copy-edited or typeset. Technical support issues arising from supporting information (other than missing files) should be addressed to the authors.
