# Supplementary material for: Decarboxylative Palladium(II)-Catalyzed Synthesis of Aryl Amidines from Aryl Carboxylic Acids: Development and Mechanistic Investigation
Source: Chemistry. 2013 Aug 28;19(41):13803–10. doi: 10.1002/chem.201301809 (PMC3935511; doi:10.1002/chem.201301809)

CHEMISTRY
A EUROPEAN JOURNAL

Supporting Information

© Copyright Wiley-VCH Verlag GmbH & Co. KGaA, 69451 Weinheim, 2013

Decarboxylative Palladium(II)-Catalyzed Synthesis of Aryl Amidines from Aryl Carboxylic Acids: Development and Mechanistic Investigation

Jonas Rydfjord,[a] Fredrik Svensson,[a] Alejandro Trejos,[a] Per J. R. Sjöberg,[b] Christian Sköld,[a] Jonas Sävmarker,[a] Luke R. Odell,[a] and Mats Larhed*[a]

chem_201301809_sm_miscellaneous_information.pdf

EXPERIMENTAL SECTION

General Information and Materials	2
General Procedure for the Synthesis of Aryl Amidines 3a-3i	2
General Procedure for the Synthesis of Aryl Amidines 3k, 3o, 3q-s and 3u	2
General Procedure for the Synthesis of Aryl Amidines 3l-n, 3p and 3t	2
Characterization Data for Compounds 3a-i and 3k-u	3
NMR Spectra for Compounds 3a-i and 3k-u	9
Continuous-Flow Scale-Out	60
DFT Study: Energies of Reported Complexes	61
DFT Study: Optimized Geometries of Reported Complexes	65
ESI-MS Study: General Procedure	85
ESI-MS Study: Observed species	86
ESI-MS Study: Spectra	87

General Information and Materials

The microwave heating was performed in a *Biotage* Initiator single mode reactor, which produces controlled irradiation at 2450 MHz. The reaction temperature was determined using the built-in online IR sensor. Microwave mediated reactions were performed in sealed *Smith* process vials designed for 2-5 mL reaction volumes. NMR spectra were recorded on a *Varian* Mercury plus at 25 °C and 400 MHz for ^{1}H and 101 MHz for ^{13}C. Chemical shifts (δ) are reported in ppm and referenced indirectly to TMS via the solvent (or residual solvent) signals. Molecular masses (HR-ESI-MS) were determined on a mass spectrometer equipped with an electrospray ion source. All starting materials, reagents and solvents are commercially available and were used as received.

General Procedure for the Synthesis of Aryl Amidines 3a-3i

To a 2-5 mL process vial $Pd(O_2CCF_3)_2$ (6.6 mg, 0.02 mmol), 6-methyl-bipyridyl (5.1 mg, 0.03 mmol) and NMP (3.0 mL) were added and the mixture was stirred for 2 min before cyanamide, **2**, (1 mmol), carboxylic acid, **1a**, (1.1 mmol) and trifluoroacetic acid (114 mg, 1 mmol) was added to the reaction mixture and the vial was instantly capped under air and then heated using microwave irradiation at 120 °C for 30 minutes. The reaction mixture was then diluted with 20 mL $NaHCO_3$ aq. and washed with 20 mL diethylether. The organic phase was further extracted with 2 x 20 mL $NaHCO_3$ aq. The combined aqueous phases were basified to pH ~ 14 by the addition of NaOH aq. and extracted with 3 x 30 mL DCM. The combined organic phases were concentrated and dried *in vacuo* to provide the pure isolated product in the yield stated in Tables 1, 2 and 3.

General Procedure for the Synthesis of Aryl Amidines 3k, 3o, 3q-s and 3u

To a 2-5 mL process vial $Pd(O_2CCF_3)_2$ (26.6 mg, 0.08 mmol), 6-methyl-bipyridyl (20.4 mg, 0.12 mmol) and NMP (3.0 mL) were added and the mixture was stirred for 2 min before cyanamide, **2**, (1 mmol), carboxylic acid, **1**, (1.1 mmol) and trifluoroacetic acid (114 mg, 1 mmol) was added to the reaction mixture and the vial was instantly capped under air and then heated using microwave irradiation at 140 °C for 60 minutes. The reaction mixture was then diluted with 20 mL $NaHCO_3$ aq. and washed with 20 mL diethylether. The organic phase was further extracted with 2 x 20 mL $NaHCO_3$ aq. The combined aqueous phases were basified to pH ~ 14 by the addition of NaOH aq. and extracted with 3 x 30 mL DCM. The combined organic phases were concentrated and dried *in vacuo* to provide the pure isolated product in the yield stated in Table 4.

General Procedure for the Synthesis of Aryl Amidines 3l-n, 3p and 3t

The compounds were prepared according to the above general procedure for **3k, 3o, 3q-s, 3u** and then further purified using preparative reversed-phase HPLC (RP-HPLC), running a gradient from 5-80% acetonitrile in water for 40 minutes to provide the pure isolated product in the yield stated in Table 4.

Characterization Data for Compounds 3a-i and 3k-u

Initial comment: Carbons close to the amidine nitrogen gives broad or non-appearing signals. This problem is circumvented for compounds **3a, 3d-e, 3g-h, 3k-l, 3p** and **3s-t** by acid addition, effectively locking the compound in one conformation and thereby making it possible to see all carbon signals.

Example:

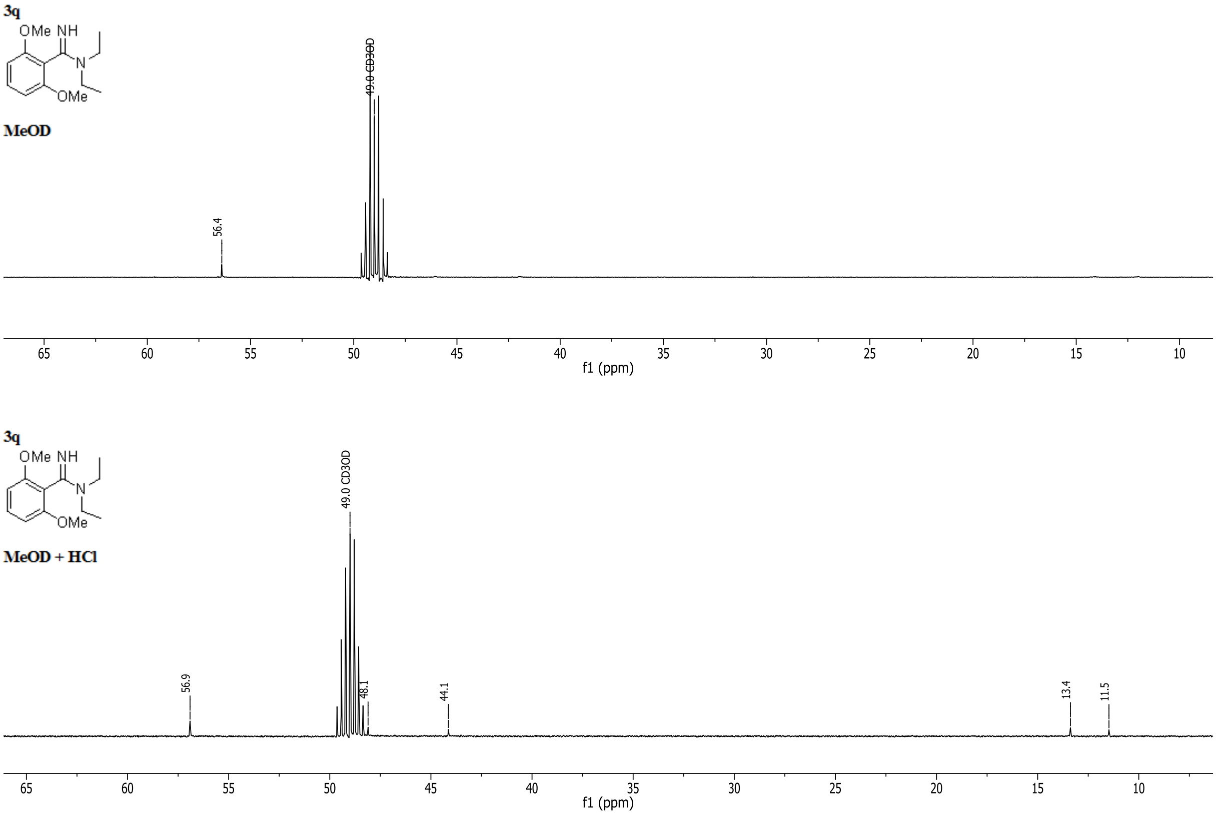

Figure S1.

Top: ^{13}C NMR spectrum of **3q** in deuterated methanol.
Bottom: ^{13}C NMR spectrum of **3q** in deuterated methanol with added HCl(aq) – compound protonated.

3a

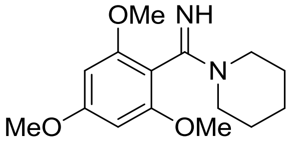

The title compound was prepared according to the general procedure to yield aryl amidine, **3a** (267 mg, 96% yield) as a semi-solid. ^{1}H NMR (400 MHz, $CDCl_3$) δ = 6.07 (s, 2H), 3.78 (s, 3H), 3.73 (s, 6H), 3.64-3.00 (m, 4H), 1.64-1.37 (m, 6H). ^{13}C NMR (101 MHz, $CDCl_3$) δ = 162.0, 161.3, 157.2, 109.9, 90.6, 55.8, 55.4, 25.9, 24.9. ^{13}C NMR (400 MHz, MeOD + 1 drop H_2SO_4) δ = 165.9, 160.3, 159.4, 100.8, 91.9, 56.8, 56.4, 51.7, 47.8, 27.1, 26.3, 24.4. HRMS calc. 279.1709 found 279.1710.

3b

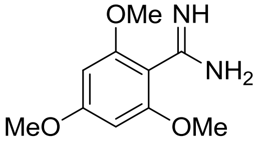

The title compound was prepared according to the general procedure to yield the aryl amidine **3b** (160 mg, 76% yield) as a semi-solid. ^{1}H NMR (400 MHz, $CDCl_3$) δ = 6.12 (s, 2H), 3.81 (s, 3H), 3.81 (s, 6H). ^{13}C NMR (101 MHz, $CDCl_3$) δ = 162.2, 161.7, 158.5, 107.5 (gHMBC), 91.0, 56.1, 55.6. HRMS calc. 211.1083 found 211.1084.

3c

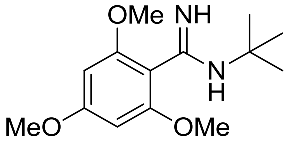

The title compound was prepared according to the general procedure to yield the aryl amidine **3c** (240 mg, 90% yield) as a semi-solid. ^{1}H NMR (400 MHz, $CDCl_3$) δ = 6.08 (s, 2H), 3.80 (s, 3H), 3.77 (s, 6H), 1.43 (s, 9H). ^{13}C NMR (101 MHz, $CDCl_3$) δ = 161.4, 159.6, 157.9, 111.7 (By gHMBC), 90.9, 56.1, 55.5, 51.5, 29.0. HRMS calc. 267.1709 found 267.1708.

3d

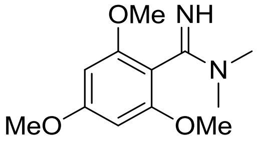

The title compound was prepared according to the general procedure to yield the aryl amidine **3d** (222 mg, 93% yield) as a semi-solid. ^{1}H NMR (400 MHz, $CDCl_3$) δ = 6.09 (s, 2H), 3.80 (s, 3H), 3.75 (s, 6H), 2.88 (br s, 6H). ^{13}C NMR (101 MHz, $CDCl_3$) δ = 163.1, 161.5, 157.2, 109.9, 90.7, 55.9, 55.5. ^{13}C NMR (400 MHz, DMSO + 1 drop 37% HCl) δ = 164.0, 160.4, 157.8, 99.8, 91.3, 56.5, 55.9, 40.5, 38.6. HRMS calc. 239.1396 found 239.1394.

3e

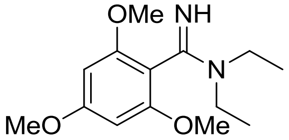

The title compound was prepared according to the general procedure to yield the aryl amidine **3e** (261 mg, 98% yield) as a semi-solid. ^{1}H NMR (400 MHz, $CDCl_3$) δ = 6.06 (s, 2H), 3.77 (s, 3H), 3.72 (s, 6H), 3.65-3.45 (m, 2H), 3.04-2.81 (m, 2H), 1.29-0.74 (m, 6H). ^{13}C NMR (101 MHz, $CDCl_3$) δ = 161.5, 161.2, 157.1, 110.4, 90.6, 55.8, 55.4, 43.4, 39.5, 14.2, 12.1. ^{13}C NMR (400 MHz, DMSO + 1 drop 37% HCl) δ = 163.8, 159.4, 157.7, 100.3, 91.4, 56.5, 56.0, 46.4, 42.7, 13.1, 11.3. HRMS calc. 267.1709 found 267.1708.

3f

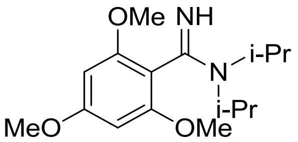

The title compound was prepared according to the general procedure to yield the aryl amidine **3f** (190 mg, 64% yield) as a semi-solid. ^{1}H NMR (400 MHz, $CDCl_3$) δ = 6.08 (s, 2H), 3.79 (s, 3H), 3.74 (s, 6H), 3.69-3.61 (m, 1H), 3.48-3.38 (m, 1H), 1.55 (d, 6H), 0.99 (d, 6H). ^{13}C NMR (101 MHz, $CDCl_3$) δ = 161.1, 160.9, 157.1, 112.5, 90.6, 55.8, 55.4, 51.2, 45.3, 21.1, 20.4.. HRMS calc. 295.2022 found 295.2025.

3g

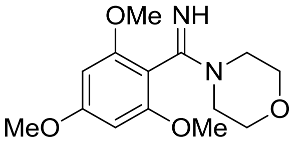

The title compound was prepared according to the general procedure to yield the aryl amidine **3g** (262 mg, 93% yield) as a semi-solid. ^{1}H NMR (400 MHz, $CDCl_3$) δ = 6.08 (s, 2H), 3.78 (s, 3H), 3.74 (s, 6H), 3.65-3.57 (m, 4H), 3.56-3.18 (m, 4H). ^{13}C NMR (101 MHz, $CDCl_3$) δ = 162.4, 161.7, 157.3, 108.5, 90.6, 66.9, 55.8, 55.5. ^{13}C NMR (400 MHz, DMSO + 1 drop 37% HCl) δ = 164.0, 158.7, 157.9, 99.0, 91.3, 66.1, 65.2, 56.4, 55.9, 49.0, 46.5. HRMS calc. 281.1501 found 281.1504.

3h

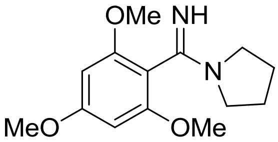

The title compound was prepared according to the general procedure to yield the aryl amidine **3h** (197 mg, 74% yield) as a semi-solid. ^{1}H NMR (400 MHz, $CDCl_3$) δ = 6.07 (s, 2H), 3.77 (s, 3H), 3.73 (s, 6H), 3.65-3.36 (m, 2H), 3.14-2.82 (m, 2H), 1.97-1.66 (m, 4H). ^{13}C NMR (101 MHz, $CDCl_3$) δ = 161.3, 160.4, 157.1, 110.8, 90.6, 55.9, 55.4, 47.8, 45.9, 25.4. ^{13}C NMR (400 MHz, DMSO + 1 drop 37% HCl) δ = 164.0, 158.0, 157.8, 100.8, 91.5, 56.6, 56.1, 50.3, 48.6, 24.9. HRMS calc. 265.1552 found 265.1558.

3i

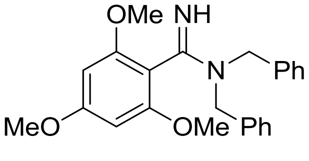

The title compound was prepared according to the general procedure to yield the aryl amidine **3i** (266 mg, 68% yield) as a semi-solid. ^{1}H NMR (400 MHz, $CDCl_3$) δ = 7.46-7.37 (m, 2H), 7.36-7.29 (m, 2H) 7.28-7.19 (m, 4H), 7.18-7.11 (m, 2H), 6.11 (s, 2H), 4.83 (br s, 2H), 4.14 (br s, 2H), 3.81 (s, 3H), 3.80 (s, 6H). ^{13}C NMR (101 MHz, $CDCl_3$) δ = 163.2, 161.8, 157.3, 138.5, 138.1, 128.3, 127.9, 127.7, 127.0, 126.7, 109.2, 90.7, 55.7, 55.5, 52.2, 47.5. HRMS calc. 391.2022 found 391.2018.

3k

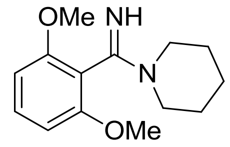

The title compound was prepared according to the general procedure to yield the aryl amidine **3k** (161 mg, 65% yield) as a semi-solid. ^{1}H NMR (400 MHz, $CDCl_3$) δ = 7.22 (t, 1H, ^3J(H,H)=8.4 Hz), 6.54 (d, 2H, ^3J(H,H)=8.4 Hz), 3.77 (s, 6H), 3.55-3.10 (m, 4H), 1.69-1.41 (m, 6H). ^{13}C NMR (101 MHz, $CDCl_3$) δ = 161.9, 156.5, 129.6, 116.8, 104.0, 56.0, 25.9, 25.0. ^{13}C NMR (400 MHz, MeOD + 1 drop 37% HCl) δ = 160.3, 158.4, 135.0, 108.4, 105.4, 56.9, 51.7, 48.0, 27.1, 26.4, 24.4 HRMS calc. 249.1603 found 249.1605.

3l

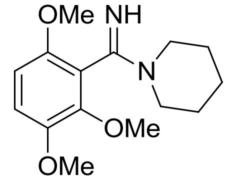

The title compound was prepared according to the general procedure and then further purified using RP-HPLC to yield the aryl amidine **3l** (26 mg, 9% yield) as a semi-solid. ^{1}H NMR (400 MHz, $CDCl_3$) δ = 6.81 (d, 1H, ^3J(H,H)=8.9 Hz), 6.57 (d, 1H, ^3J(H,H)=8.9 Hz), 3.84 (s, 3H), 3.83 (s, 3H), 3.75 (s, 3H), 3.70-2.87 (m, 4H), 1.65-1.47 (m, 6H). ^{13}C NMR (101 MHz, $CDCl_3$) δ =161.8, 150.0, 147.2, 145.9, 123.4, 112.3, 106.1, 61.6, 56.4, 56.3, 26.0, 25.0. ^{13}C NMR (400 MHz, MeOD + 1 drop 37% HCl) δ = 160.1, 151.1, 148.0, 147.5, 117.9, 114.3, 107.7, 61.9, 57.1, 56.9, 52.0, 48.1, 27.0, 26.4, 24.4. HRMS calc. 279.1709 found 279.1714.

3m

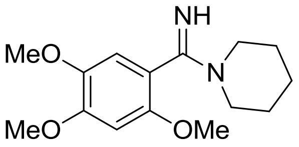

The title compound was prepared according to the general procedure and then further purified using RP-HPLC to yield the aryl amidine **3m** (23 mg, 8% yield) as a semi-solid. ^{1}H NMR (400 MHz, $CDCl_3$) δ = 6.74 (s, 1H), 6.51 (s, 1H), 3.90 (s, 3H), 3.82 (s, 3H), 3.79 (s, 3H), 3.50-3.20 (m, 4H), 1.77-1.42 (m, 6H). ^{13}C NMR (101 MHz, $CDCl_3$) δ = 165.8, 149.9, 149.8, 143.2, 119.6, 112.0, 97.8, 56.8, 56.7, 56.3, 46.3, 26.0, 24.9. HRMS calc. 279.1709 found 279.1707.

3n

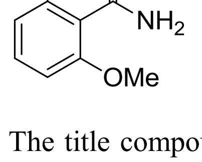

The title compound was prepared according to the general procedure and then further purified using RP-HPLC to yield the aryl amidine **3n** (65 mg, 36% yield) as a semi-solid. ^{1}H NMR (400 MHz, $CDCl_3$) δ = 7.26 (t, 1H, ^3J(H,H)=8.4 Hz), 6.58 (d, 2H, ^3J(H,H)=8.4 Hz), 3.83 (s, 3H). ^{13}C NMR (101 MHz, MeOD) δ = 161.5, 157.0, 130.4, 115.4 (by gHMBC), 104.2, 56.1. HRMS calc. 181.0977 found 181.0975.

3o

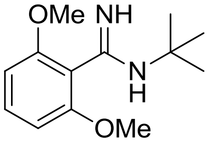

The title compound was prepared according to the general procedure to yield the aryl amidine **3o** (123 mg, 52% yield) as a semi-solid. ^{1}H NMR (400 MHz, $CDCl_3$) δ= 7.21 (t, 1H, ^{3}J(H,H)=8.4 Hz), 6.54 (d, 2H, ^{3}J(H,H)=8.4 Hz), 3.80 (s, 6H), 1.46 (s, 9H). ^{13}C NMR (101 MHz, $CDCl_3$) δ =159.6, 157.0, 129.8, 119.1 (confirmed by gHMBC), 104.3, 56.2, 51.6 (confirmed by gHMBC), 29.0. HRMS calc. 237.1603 found 237.1602.

3p

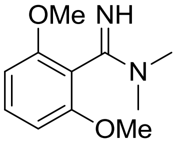

The title compound was prepared according to the general procedure and then further purified using RP-HPLC to yield the aryl amidine **3p** (76 mg, 37% yield) as a semi-solid. ^{1}H NMR (400 MHz, $CDCl_3$) δ =7.22 (t, 1H, ^{3}J(H,H)=8.4 Hz), 6.54 (d, 2H, ^{3}J(H,H)=8.4 Hz), 3.78 (s, 6H), 3.26-2.50 (m, 6H). ^{13}C NMR (101 MHz, $CDCl_3$) δ = 163.0, 156.5, 129.7, 117.0, 104.0, 56.0. ^{13}C NMR (400 MHz, MeOD + 1 drop 37% HCl) δ = 162.8, 158.4, 135.2, 108.1, 105.4, 56.9, 41.4, 38.7. HRMS calc. 209.1290 found 209.1285.

3q

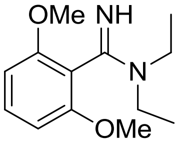

The title compound was prepared according to the general procedure to yield the aryl amidine **3q** (114 mg, 48% yield) as a semi-solid. ^{1}H NMR (400 MHz, $CDCl_3$) δ = 7.22 (t, 1H, ^{3}J(H,H)=8.3 Hz), 6.53 (d, 2H, ^{3}J(H,H)=8.3 Hz), 3.77 (s, 6H), 3.67-3.49 (m, 2 H), 3.07-2.82 (m, 2H), 1.31-1.08 (m, 3H), 1.07-0.83 (m, 3H). ^{13}C NMR (101 MHz, $CDCl_3$) δ = 161.5, 156.4, 129.7, 117.1, 104.0, 55.9, 43.6, 39.7, 14.2, 12.1. HRMS calc. 237.1603 found 237.1605.

3r

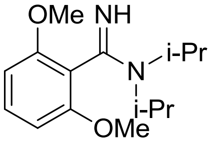

The title compound was prepared according to the general procedure to yield the aryl amidine **3r** (196 mg, 74% yield) as a semi-solid. ^{1}H NMR (400 MHz, $CDCl_3$) δ = 7.20 (t, J = 8.4 Hz, 1H), 6.53 (d, J = 8.4 Hz, 2H), 3.78 (s, 6H), 3.74-3.64 (m, 1H), 3.48-3.34 (m, 1H), 1.58 (d, 6H), 1.02 (d, 6H). ^{13}C NMR (101 MHz, $CDCl_3$) δ = 161.0, 156.4, 129.1, 119.0, 104.1, 55.9, 51.5, 45.5, 21.1, 20.4. HRMS calc. 265.1916 found 265.1917.

3s

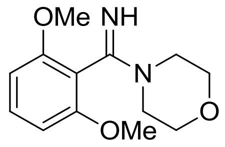

The title compound was prepared according to the general procedure to yield the aryl amidine **3s** (158 mg, 63% yield) as a semi-solid. ^{1}H NMR (400 MHz, $CDCl_3$) δ = 7.24 (t, 1H, ^{3}J(H,H)=8.4 Hz), 6.54, (d, 2H, ^{3}J(H,H)=8.4 Hz), 3.78 (s, 6H), 3.69-3.61 (m, 4H), 3.55-3.24 (m, 4H). ^{13}C NMR (101 MHz, $CDCl_3$) δ = 162.4, 156.6, 130.1, 115.7, 104.0, 66.9, 55.9. ^{13}C NMR (400 MHz, MeOD + 1 drop 37% HCl) δ = 161.3, 158.5, 135.4, 107.5, 105.5, 67.6, 66.6, 56.9, 50.5, 47.4. HRMS calc. 251.1396 found 251.1395.

3t

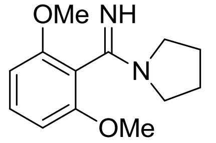

The title compound was prepared according to the general procedure to yield the aryl amidine **3t** (75 mg, 32% yield) as a semi-solid. ^{1}H NMR (400 MHz, $CDCl_3$) δ = 7.24 (t, 1H, ^{3}J(H,H)=8.4 Hz), 6.55 (d, 2H, ^{3}J(H,H)=8.4 Hz), 3.79 (s, 6H), 3.73-2.76 (m, 4H), 2.15-1.68 (m, 4H). ^{13}C NMR (101 MHz, $CDCl_3$) δ = 160.4, 156.5, 129.9, 117.4, 104.1, 56.0. ^{13}C NMR (400 MHz, MeOD + 1 drop 37% HCl) δ = 160.1, 158.2, 135.0, 109.0, 105.5, 56.9, 51.7, 49.3, 26.1. HRMS calc. 235.1447 found 235.1449.

3u

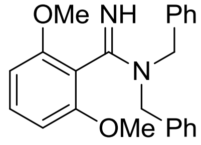

The title compound was prepared according to the general procedure to yield the aryl amidine **3u** (265 mg, 74% yield) as a semi-solid. ^{1}H NMR (400 MHz, $CDCl_3$) δ = 7.48 – 7.12 (m, 11H), 6.56 (d, J = 8.4 Hz, 2H), 4.86 (br s, 2H), 4.14 (br s, 2H), 3.83 (s, 6H). ^{13}C NMR (101 MHz, $CDCl_3$) δ = 163.1, 156.5, 138.4, 138.1, 130.0, 128.3, 127. 8, 127.0, 126.6, 116.4, 104.0, 55.7, 52.1, 47.3. HRMS calc. 361.1916 found. 361.1918.

NMR Spectra for Compounds 3a-i and 3k-u

3a

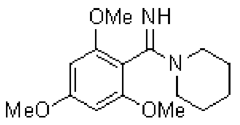
OMe NH
N
MeO
OMe

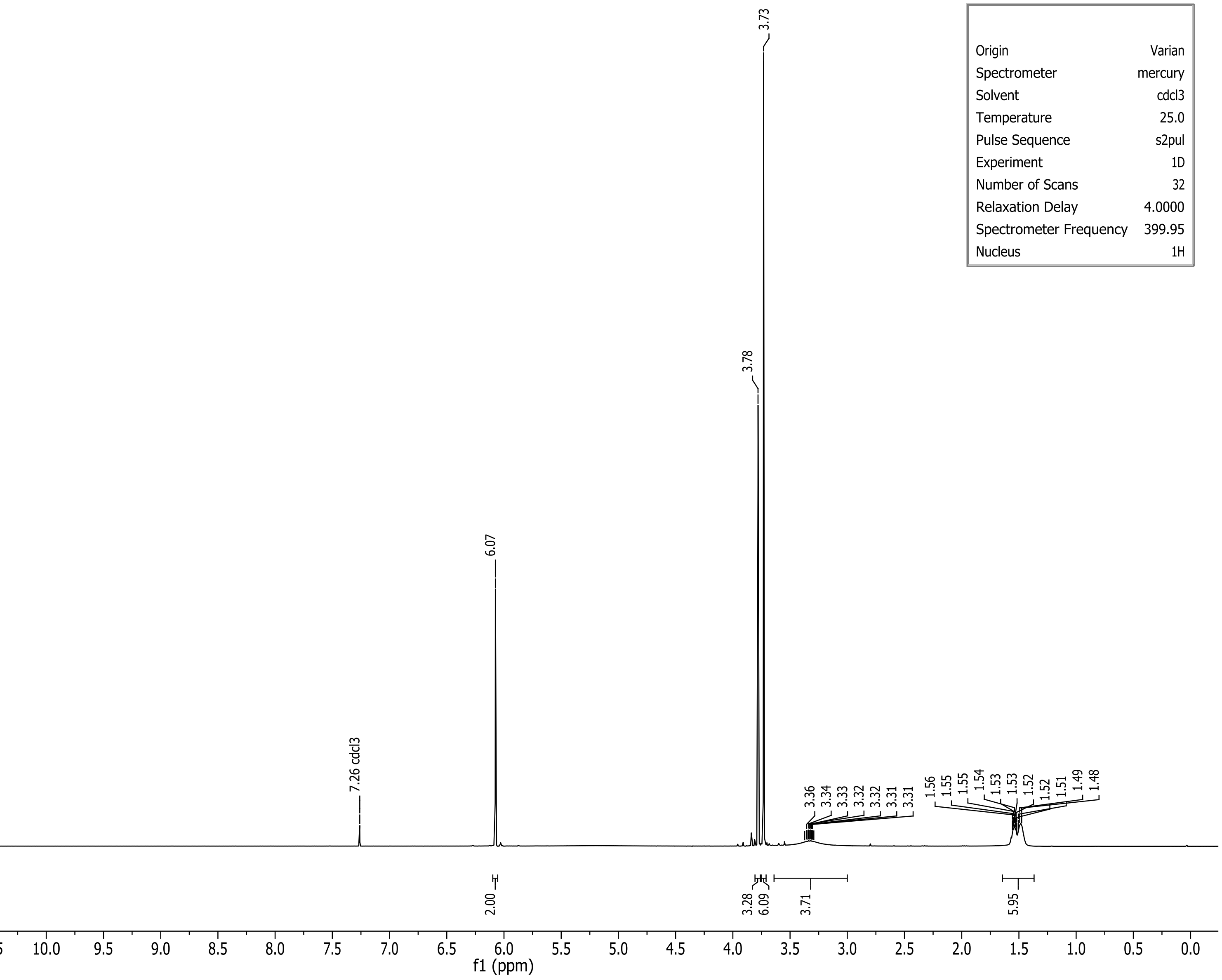
3.73
3.78
6.07
7.26 cdcl3
3.36
3.34
3.33
3.32
3.32
3.31
3.31
1.56
1.55
1.55
1.54
1.53
1.53
1.52
1.52
1.51
1.49
1.48
2.00
3.28
6.09
3.71
5.95
12.0 11.5 11.0 10.5 10.0 9.5 9.0 8.5 8.0 7.5 7.0 6.5 6.0 5.5 5.0 4.5 4.0 3.5 3.0 2.5 2.0 1.5 1.0 0.5 0.0
f1 (ppm)
Origin Varian
Spectrometer mercury
Solvent cdcl3
Temperature 25.0
Pulse Sequence s2pul
Experiment 1D
Number of Scans 32
Relaxation Delay 4.0000
Spectrometer Frequency 399.95
Nucleus 1H

3a

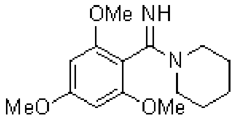

Origin	Varian
Spectrometer	mercury
Solvent	cdcl3
Temperature	25.0
Pulse Sequence	s2pul
Experiment	1D
Number of Scans	512
Relaxation Delay	1.0000
Spectrometer Frequency	100.58
Nucleus	13C

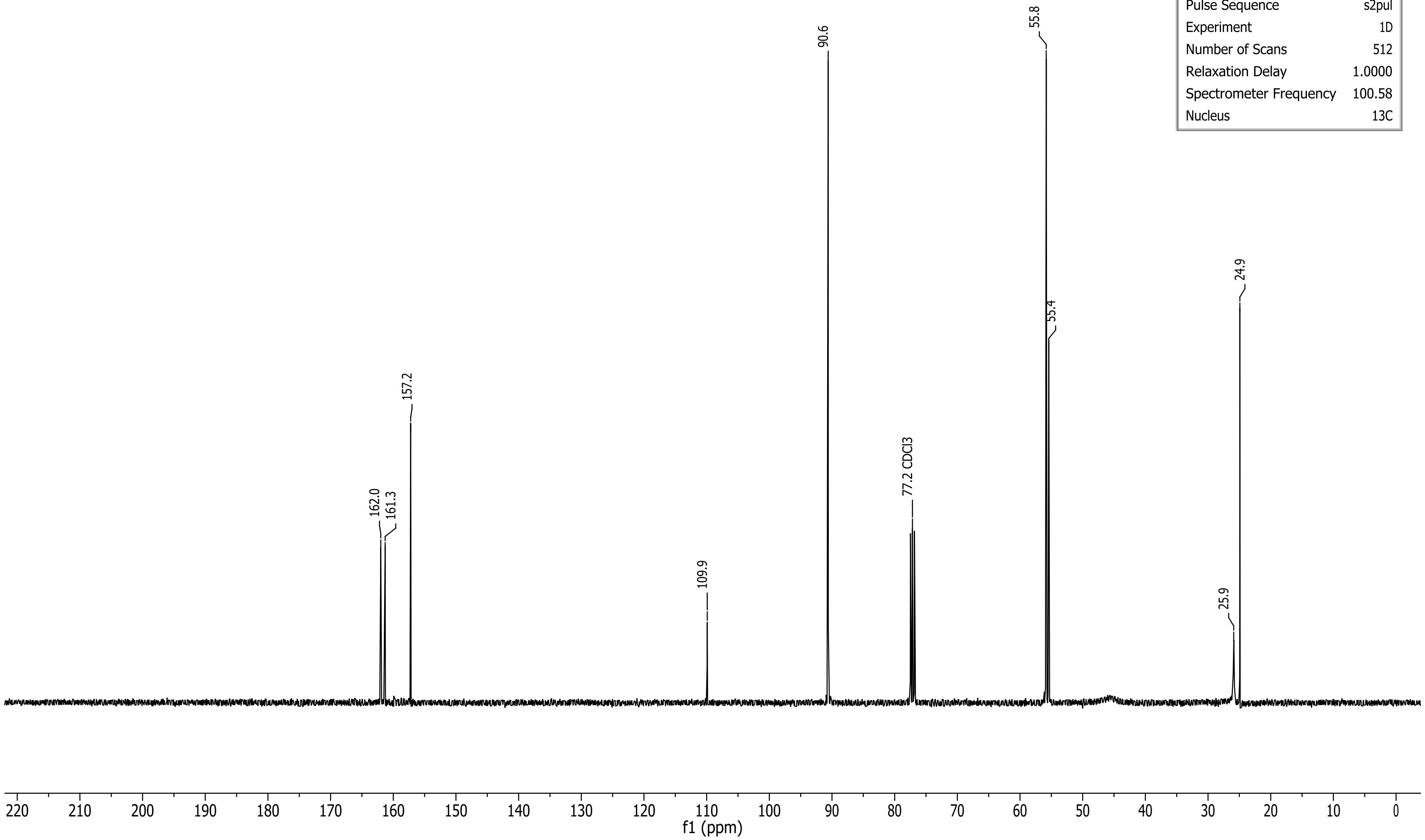

3a

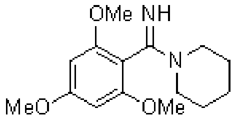

H_2SO_4 added

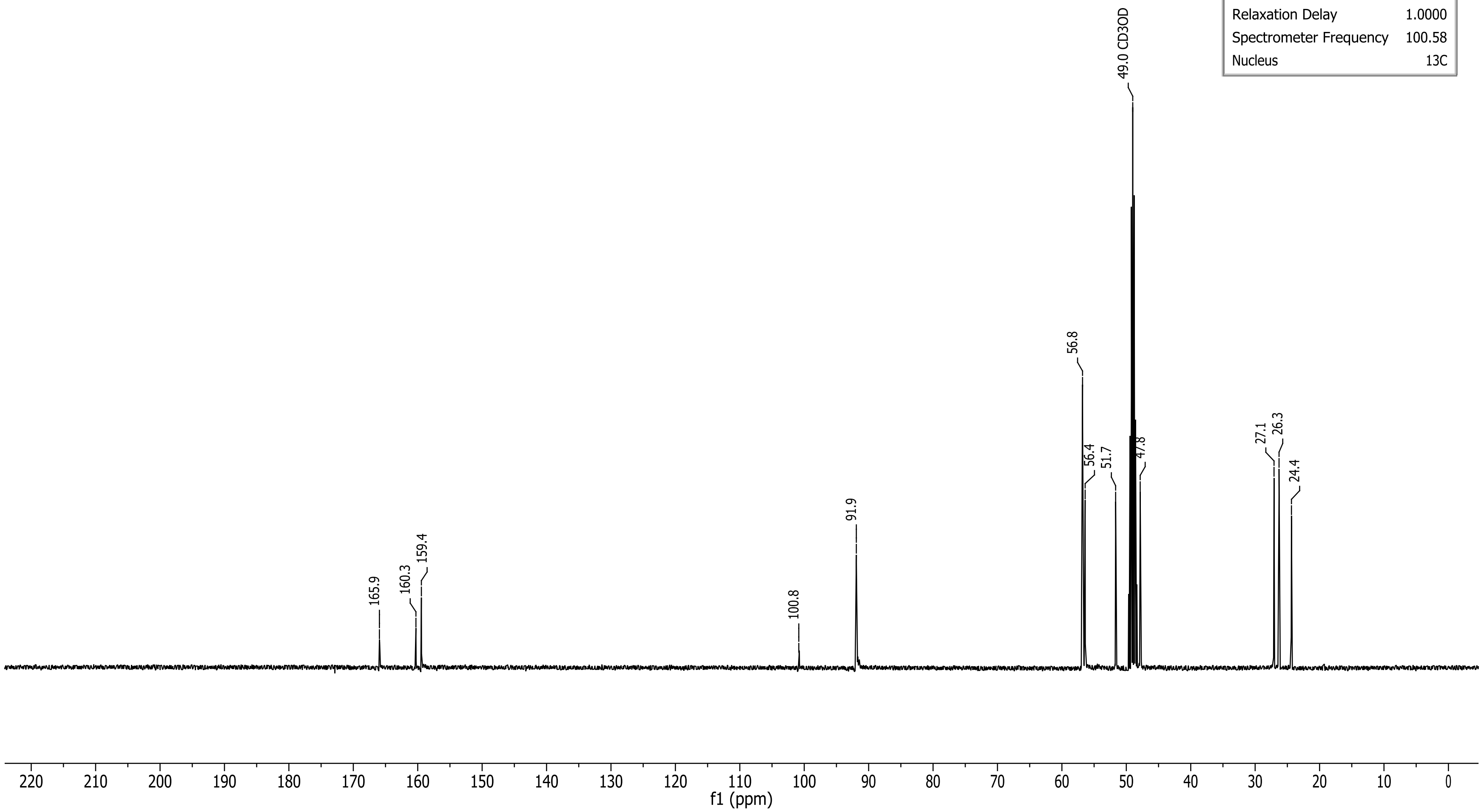

3b

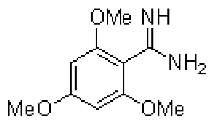

Origin	Varian
Spectrometer	mercury
Solvent	cdcl3
Temperature	25.0
Pulse Sequence	s2pul
Experiment	1D
Number of Scans	32
Relaxation Delay	27.0000
Spectrometer Frequency	399.86
Nucleus	1H

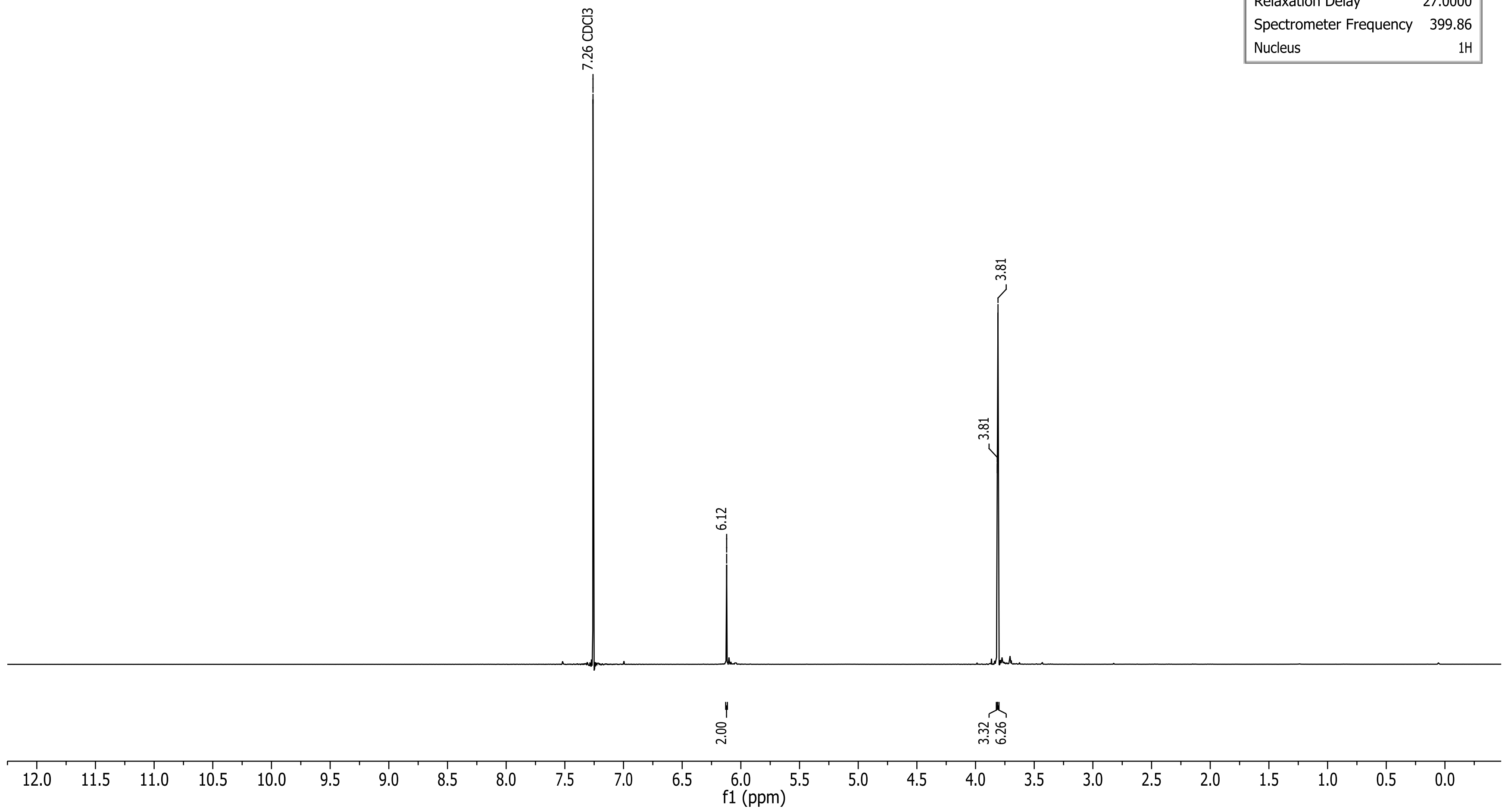

3b

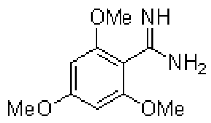

Origin	Varian
Spectrometer	mercury
Solvent	cdcl3
Temperature	25.0
Pulse Sequence	s2pul
Experiment	1D
Number of Scans	4096
Relaxation Delay	1.0000
Spectrometer Frequency	100.55
Nucleus	13C

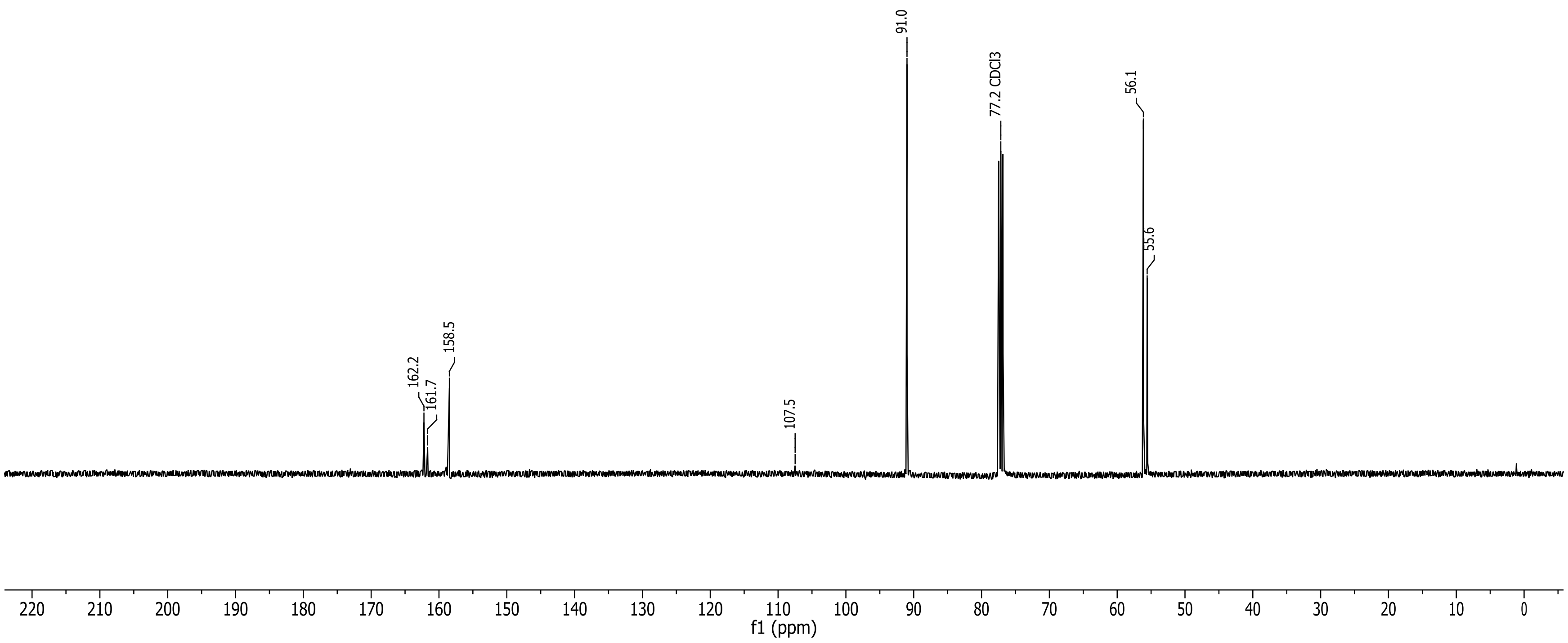

3c

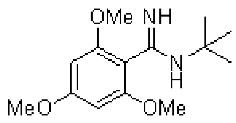

Origin	Varian
Spectrometer	mercury
Solvent	cdcl3
Temperature	25.0
Pulse Sequence	s2pul
Experiment	1D
Number of Scans	32
Relaxation Delay	27.0000
Spectrometer Frequency	399.95
Nucleus	1H

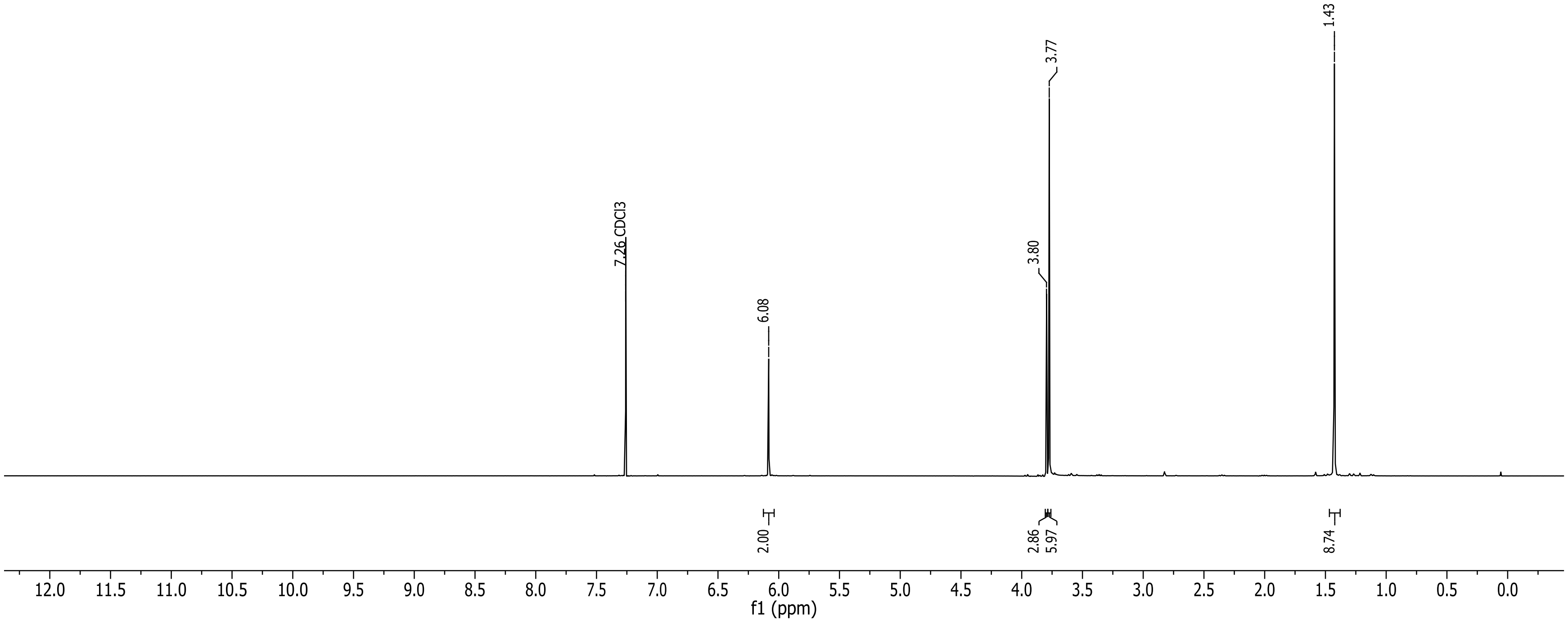

3c

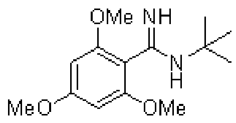

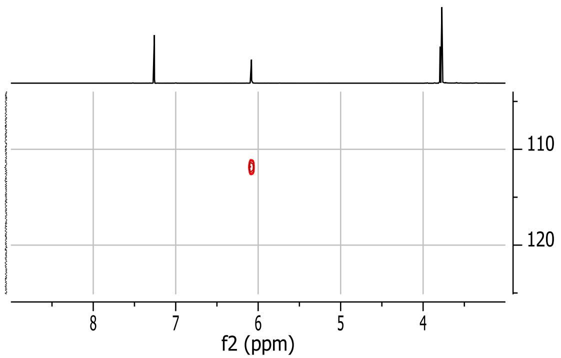

Origin	Varian
Spectrometer	mercury
Solvent	cdcl3
Temperature	25.0
Pulse Sequence	s2pul
Experiment	1D
Number of Scans	1024
Relaxation Delay	1.0000
Spectrometer Frequency	100.58
Nucleus	13C

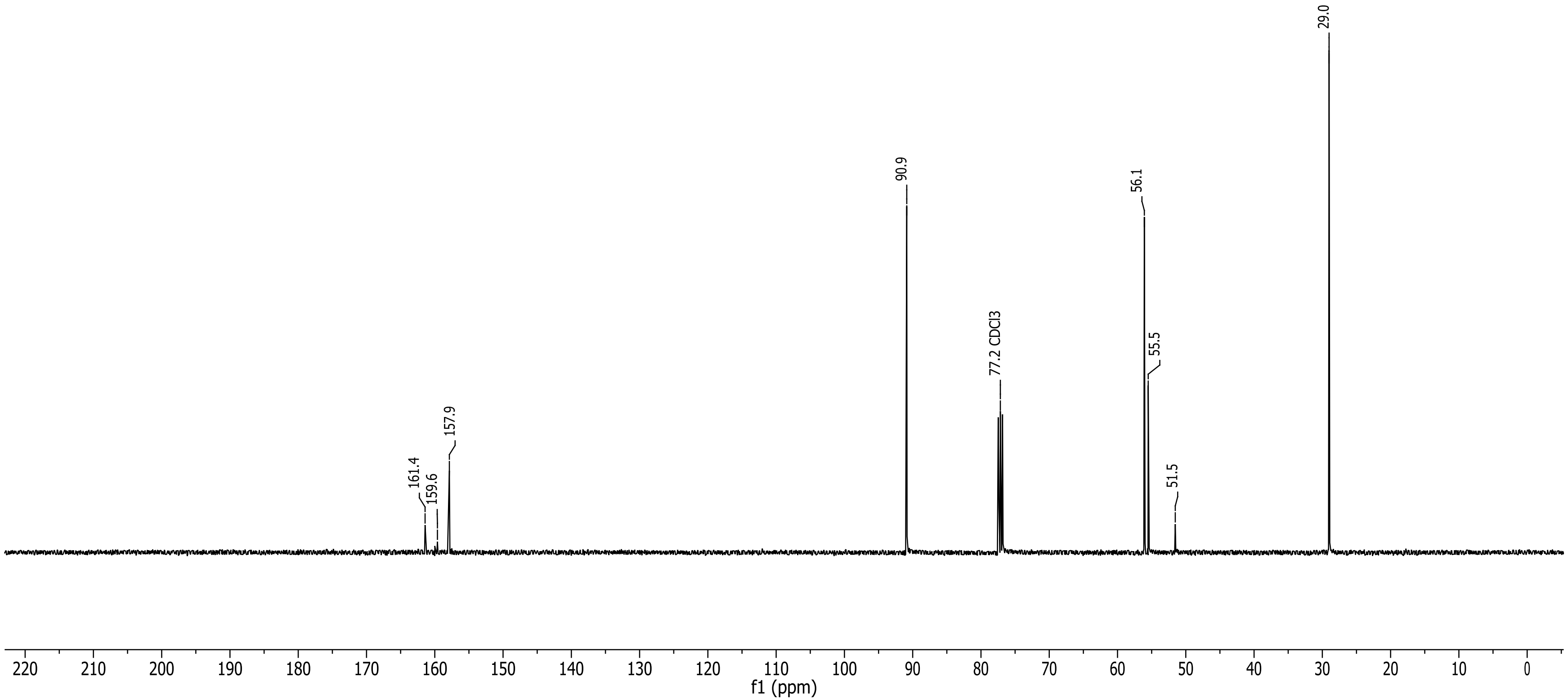

3d

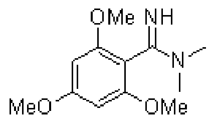

Origin	Varian
Spectrometer	mercury
Solvent	cdcl3
Temperature	25.0
Pulse Sequence	s2pul
Experiment	1D
Number of Scans	32
Relaxation Delay	27.0000
Spectrometer Frequency	399.86
Nucleus	1H

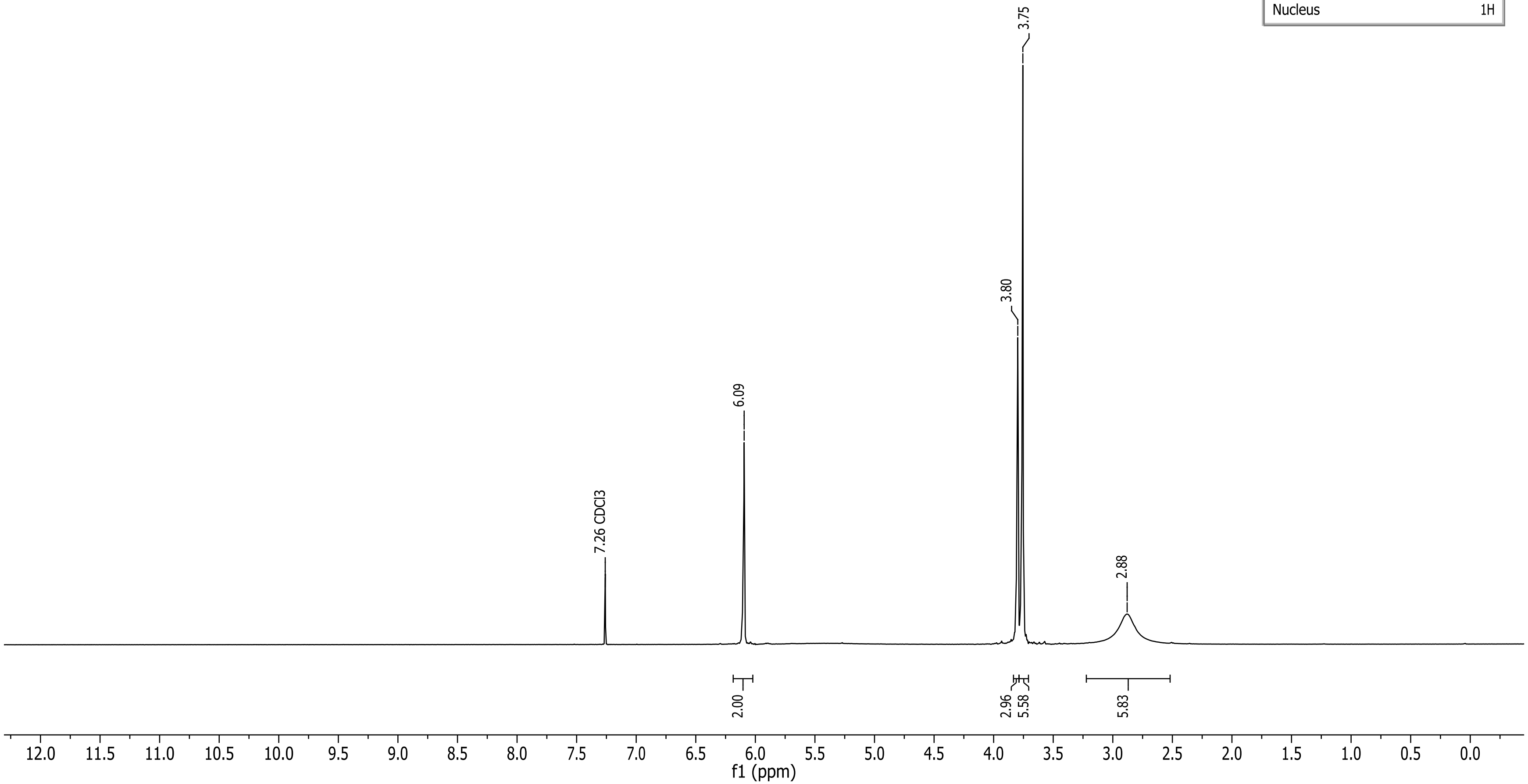

3d

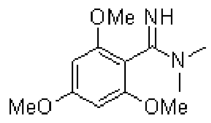

Origin	Varian
Spectrometer	mercury
Solvent	cdcl3
Temperature	25.0
Pulse Sequence	s2pul
Experiment	1D
Number of Scans	1024
Relaxation Delay	1.0000
Spectrometer Frequency	100.55
Nucleus	13C

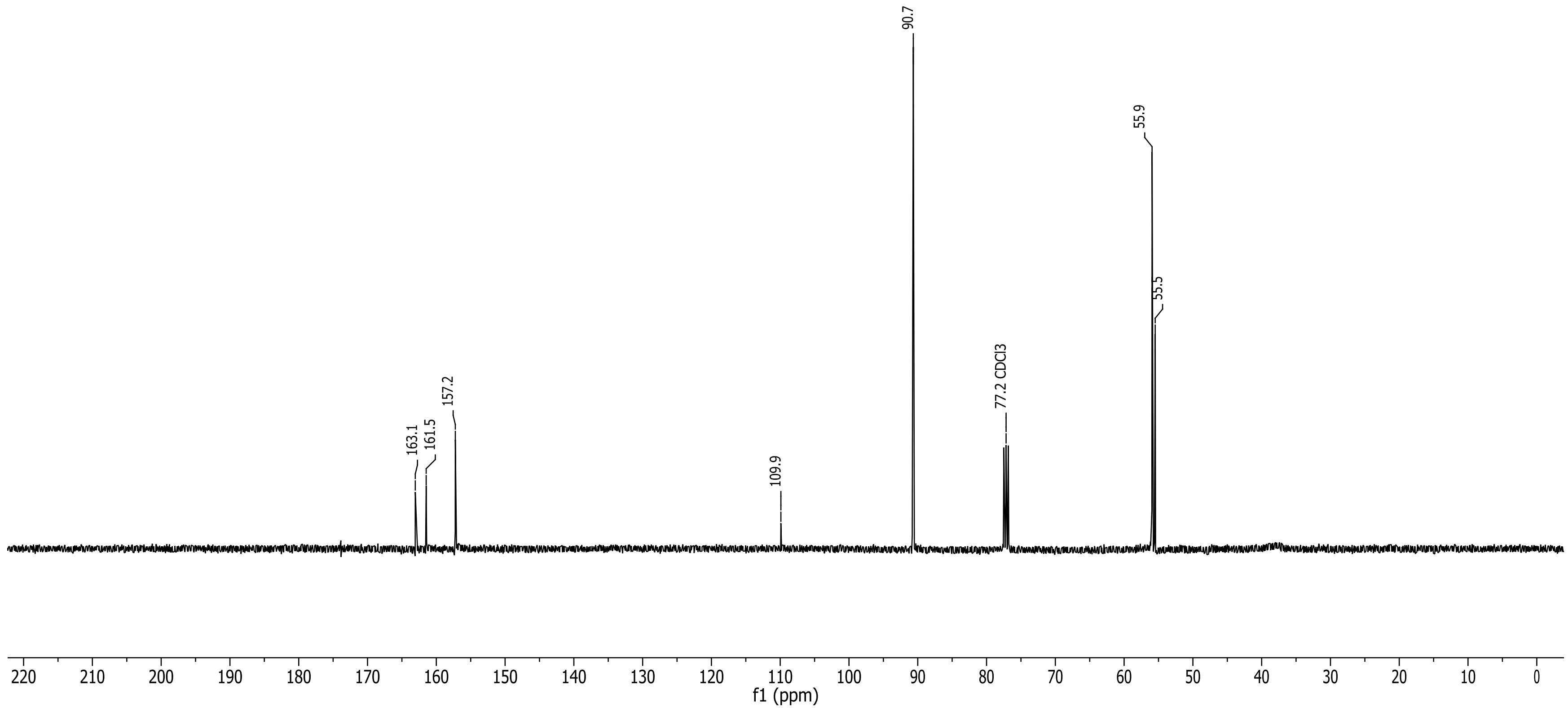

3d

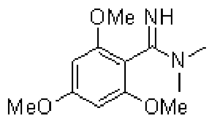
OMe NH
N
MeO
OMe

HCl added

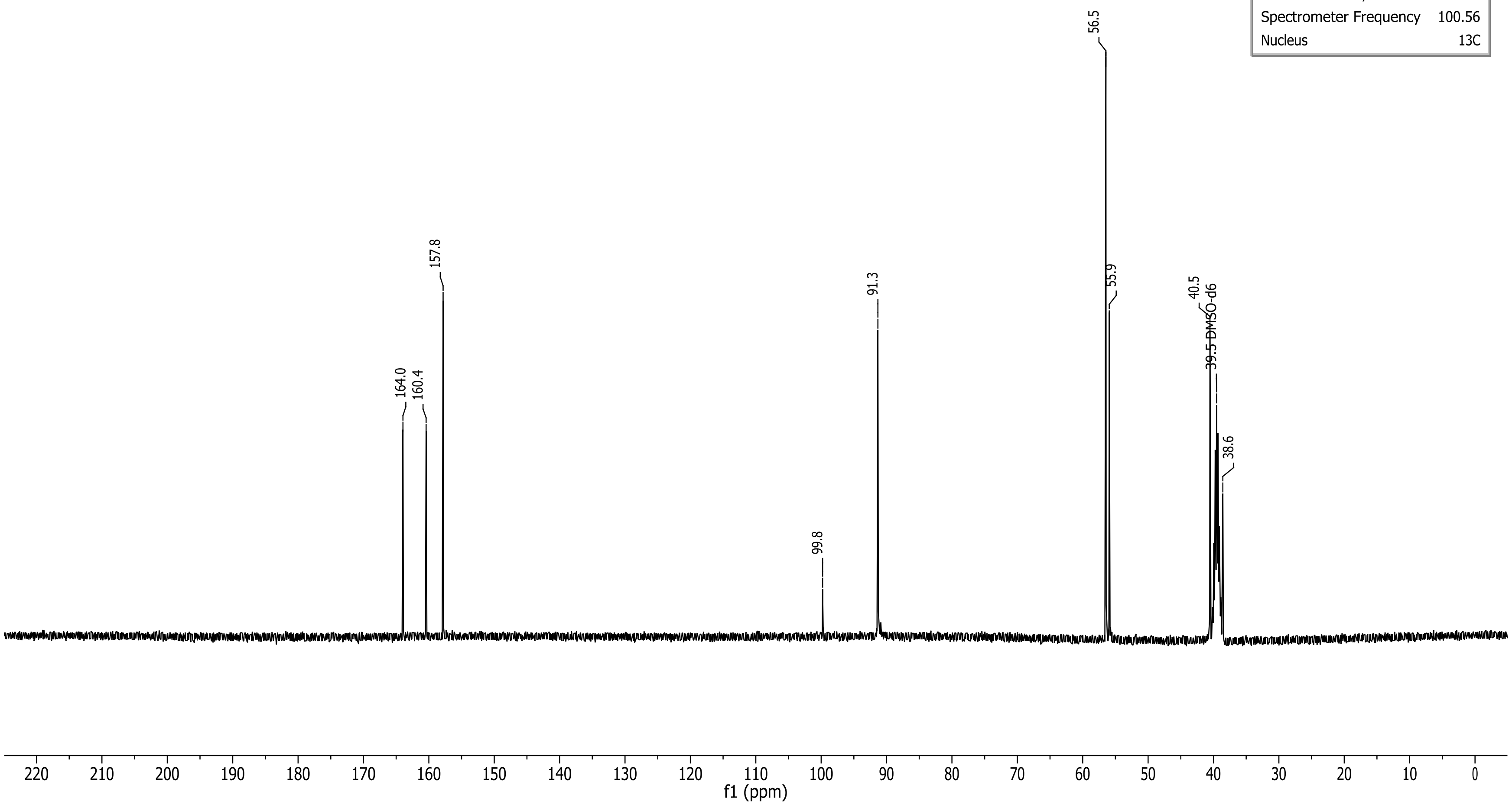
Origin Varian
Spectrometer mercury
Solvent dmso
Temperature 25.0
Pulse Sequence s2pul
Experiment 1D
Number of Scans 1024
Relaxation Delay 1.0000
Spectrometer Frequency 100.56
Nucleus 13C
164.0
160.4
157.8
99.8
91.3
56.5
55.9
40.5
39.5 DMSO-d6
38.6
220 210 200 190 180 170 160 150 140 130 120 110 100 90 80 70 60 50 40 30 20 10 0
f1 (ppm)

3e

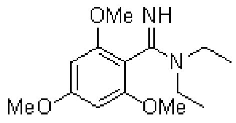

Origin	Varian
Spectrometer	mercury
Solvent	cdcl3
Temperature	25.0
Pulse Sequence	s2pul
Experiment	1D
Number of Scans	32
Relaxation Delay	4.0000
Spectrometer Frequency	399.86
Nucleus	1H

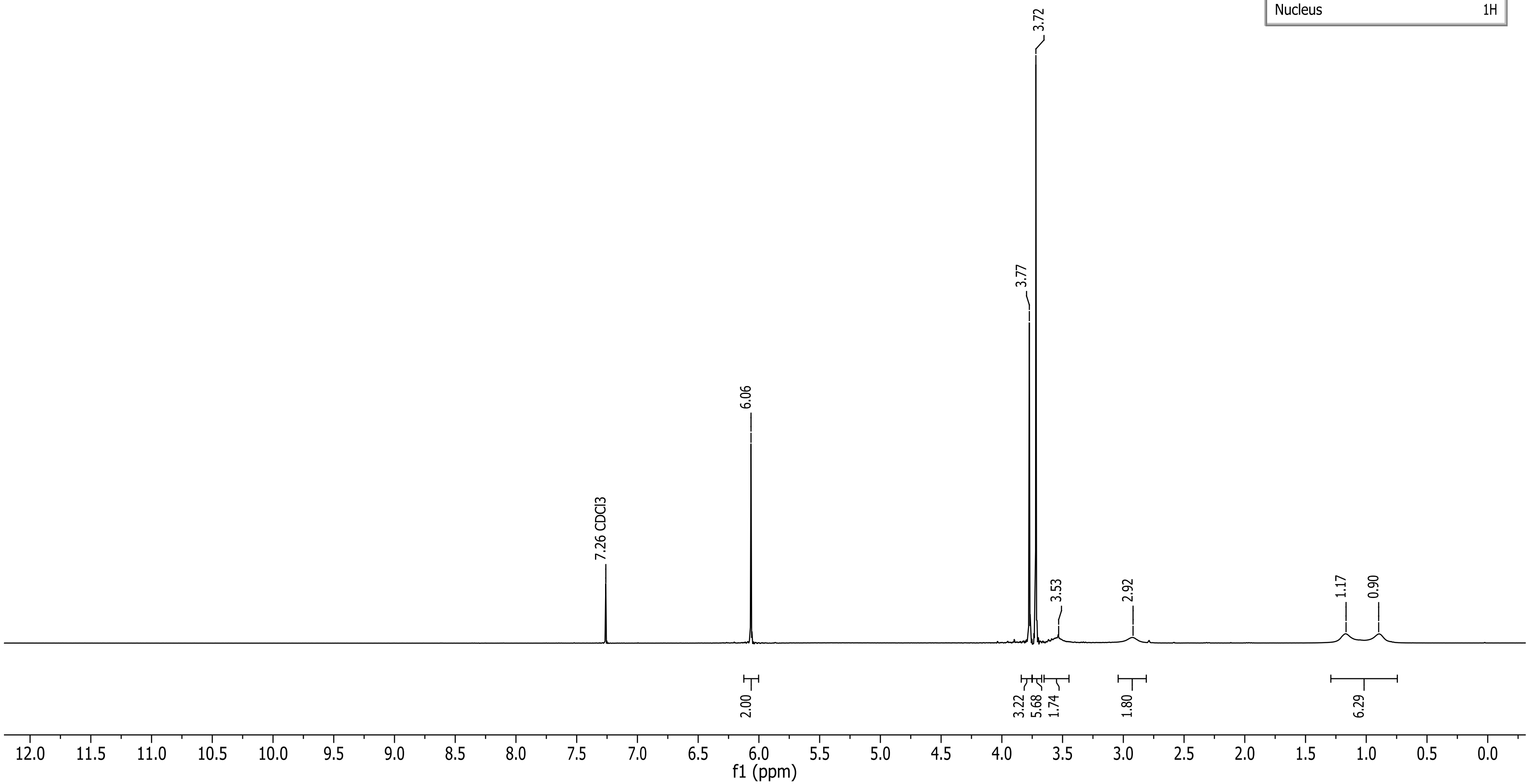

3e

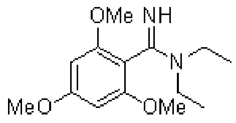

Origin	Varian
Spectrometer	mercury
Solvent	cdcl3
Temperature	25.0
Pulse Sequence	s2pul
Experiment	1D
Number of Scans	512
Relaxation Delay	1.0000
Spectrometer Frequency	100.55
Nucleus	13C

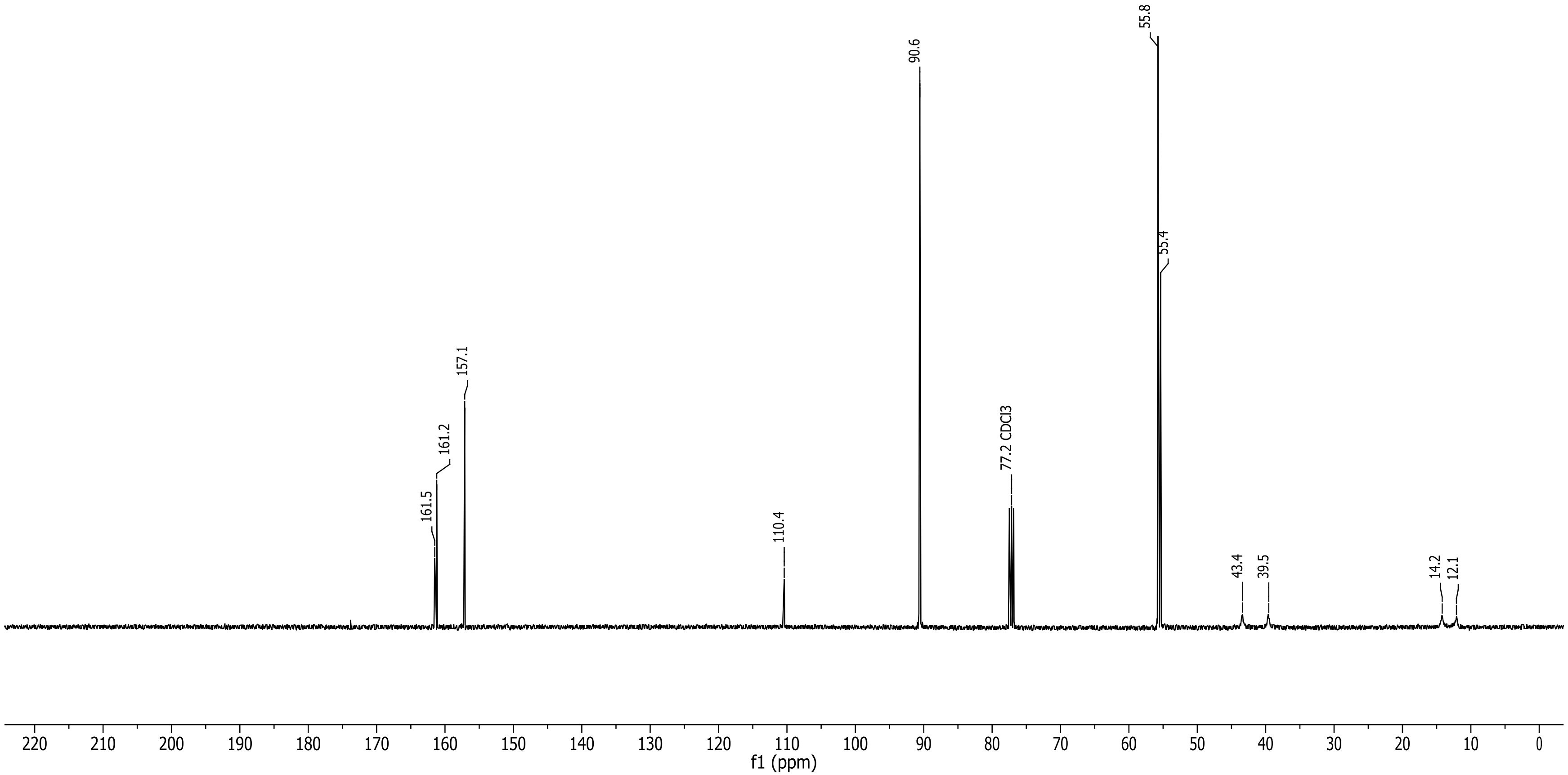

3e

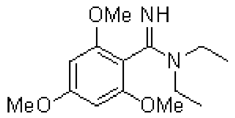

HCl added

Origin	Varian
Spectrometer	mercury
Solvent	dmso
Temperature	25.0
Pulse Sequence	s2pul
Experiment	1D
Number of Scans	1024
Relaxation Delay	1.0000
Spectrometer Frequency	100.56
Nucleus	13C

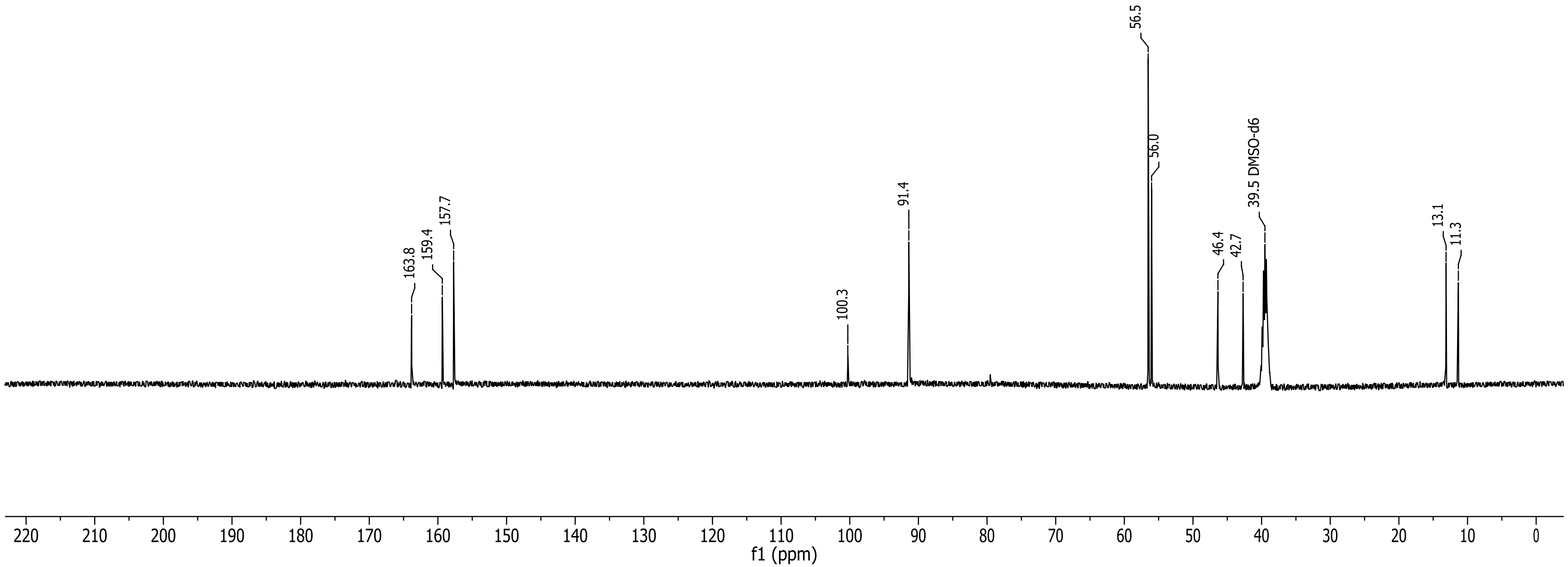

3f

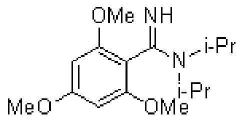

Origin	Varian
Spectrometer	mercury
Solvent	cdcl3
Temperature	25.0
Pulse Sequence	s2pul
Experiment	1D
Number of Scans	32
Relaxation Delay	27.0000
Spectrometer Frequency	399.86
Nucleus	1H

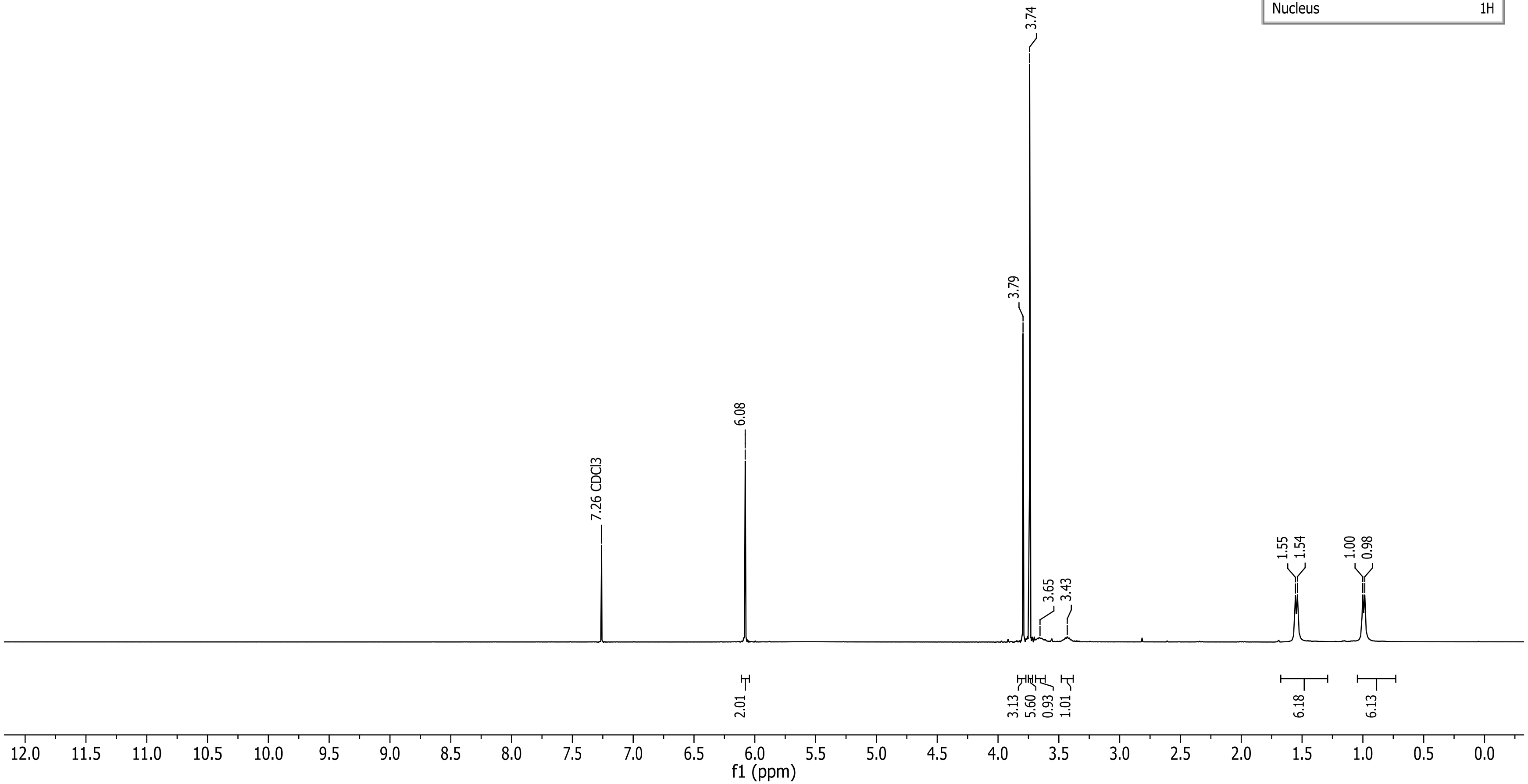

3f

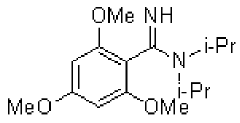

Origin	Varian
Spectrometer	mercury
Solvent	cdcl3
Temperature	25.0
Pulse Sequence	s2pul
Experiment	1D
Number of Scans	1024
Relaxation Delay	1.0000
Spectrometer Frequency	100.55
Nucleus	13C

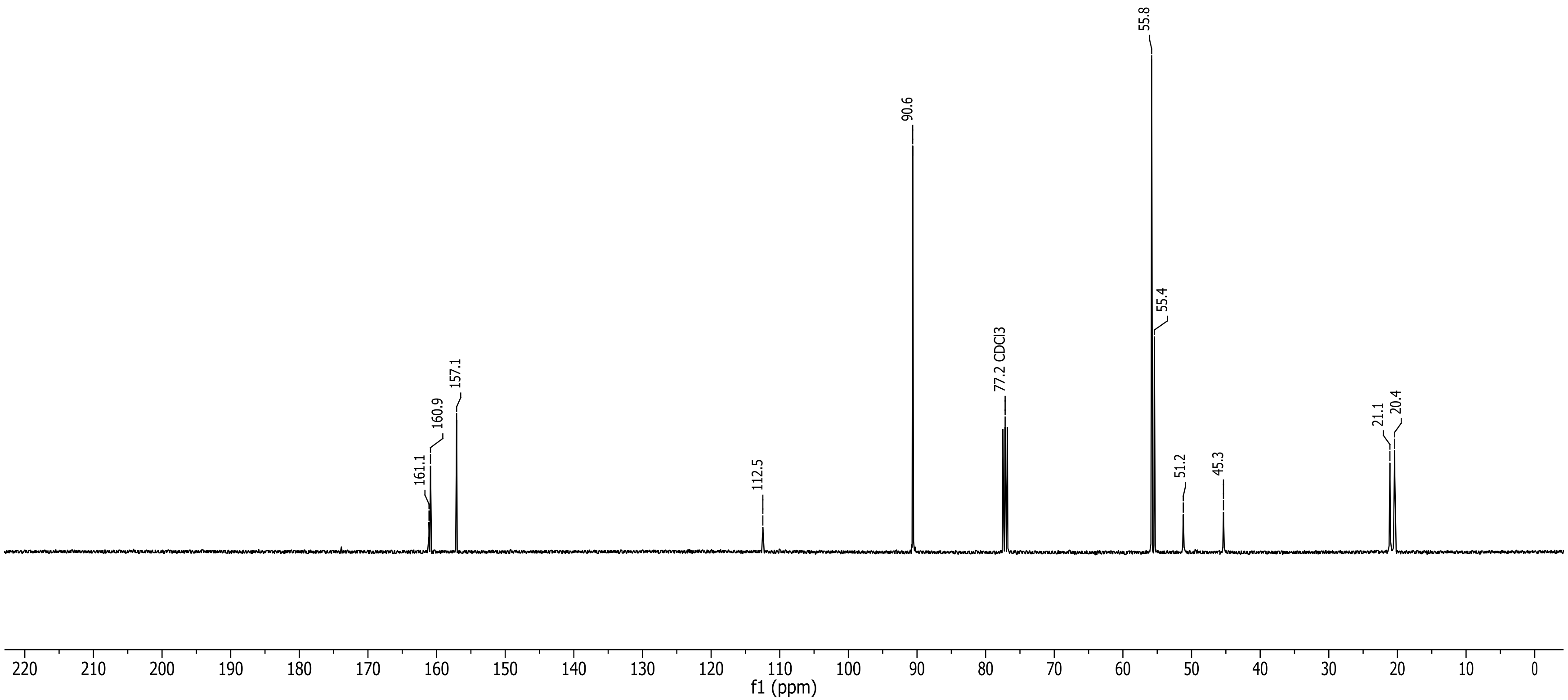

3g

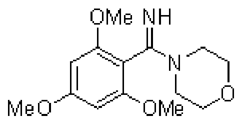

Origin	Varian
Spectrometer	mercury
Solvent	cdcl3
Temperature	25.0
Pulse Sequence	s2pul
Experiment	1D
Number of Scans	32
Relaxation Delay	4.0000
Spectrometer Frequency	399.95
Nucleus	1H

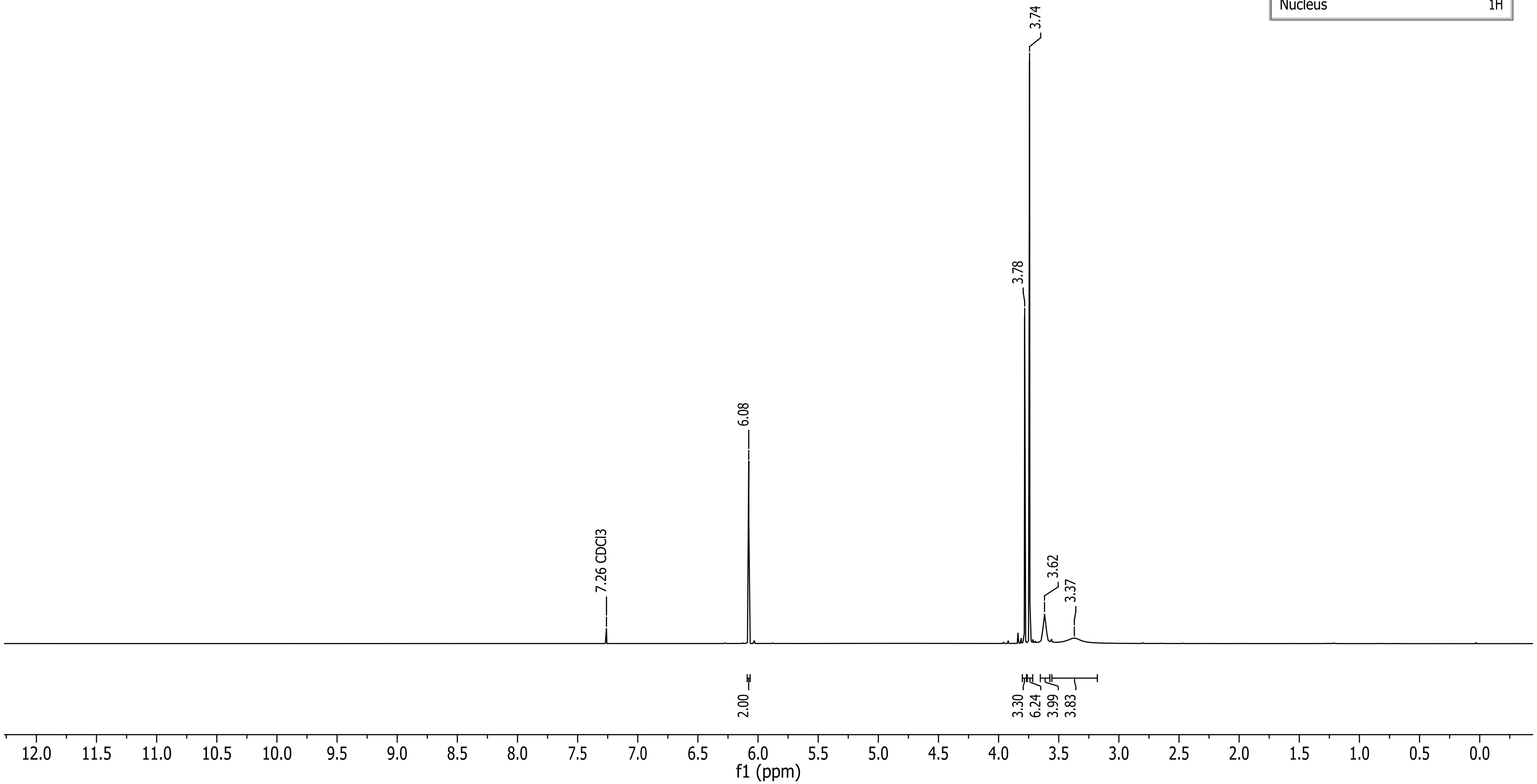

3g

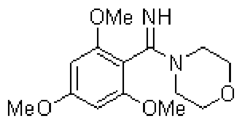

Origin	Varian
Spectrometer	mercury
Solvent	cdcl3
Temperature	25.0
Pulse Sequence	s2pul
Experiment	1D
Number of Scans	512
Relaxation Delay	1.0000
Spectrometer Frequency	100.58
Nucleus	13C

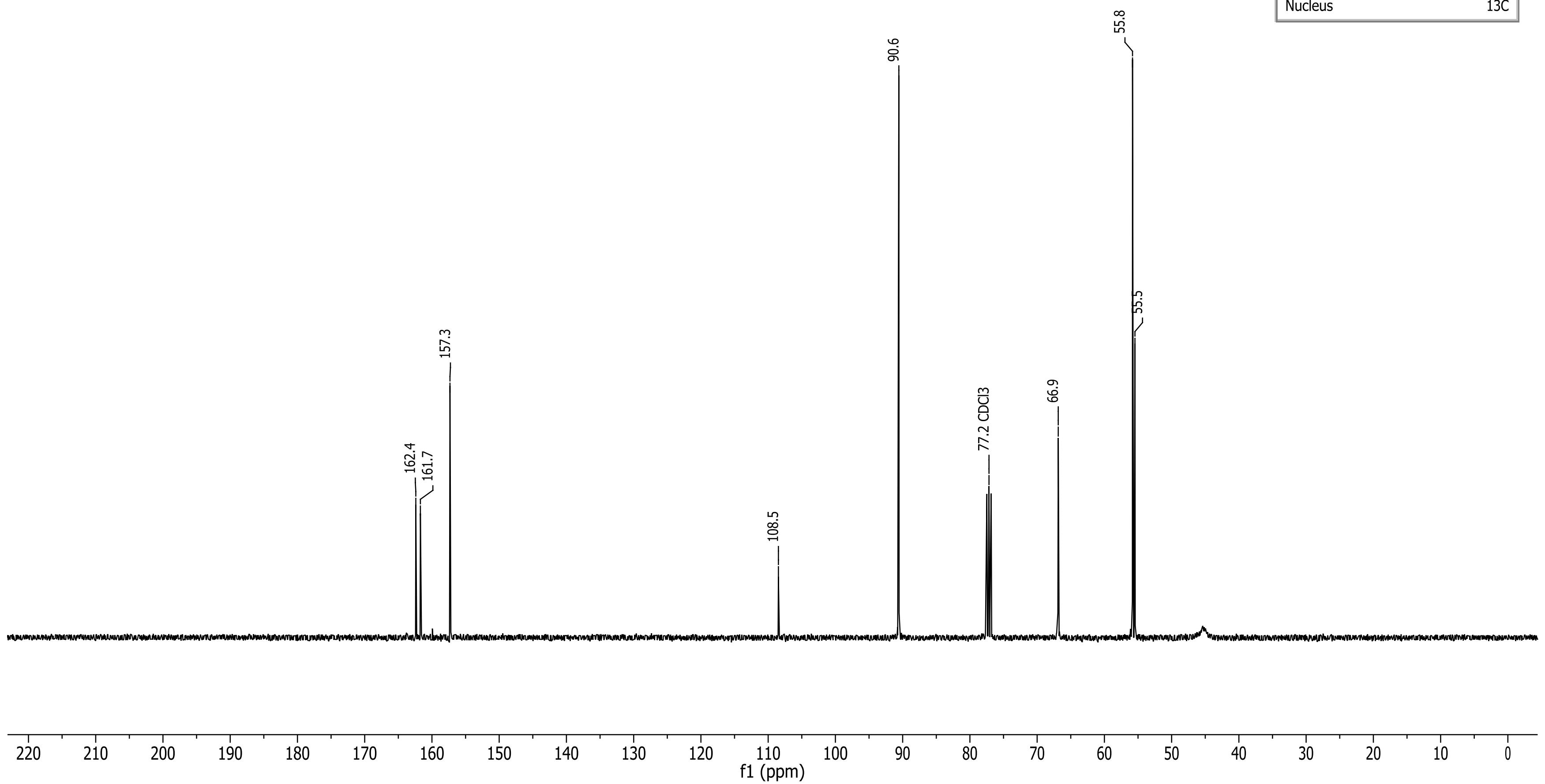

3g

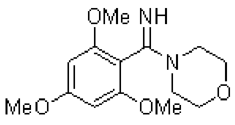

HCl added

Origin	Varian
Spectrometer	mercury
Solvent	dmso
Temperature	25.0
Pulse Sequence	s2pul
Experiment	1D
Number of Scans	1024
Relaxation Delay	1.0000
Spectrometer Frequency	100.56
Nucleus	13C

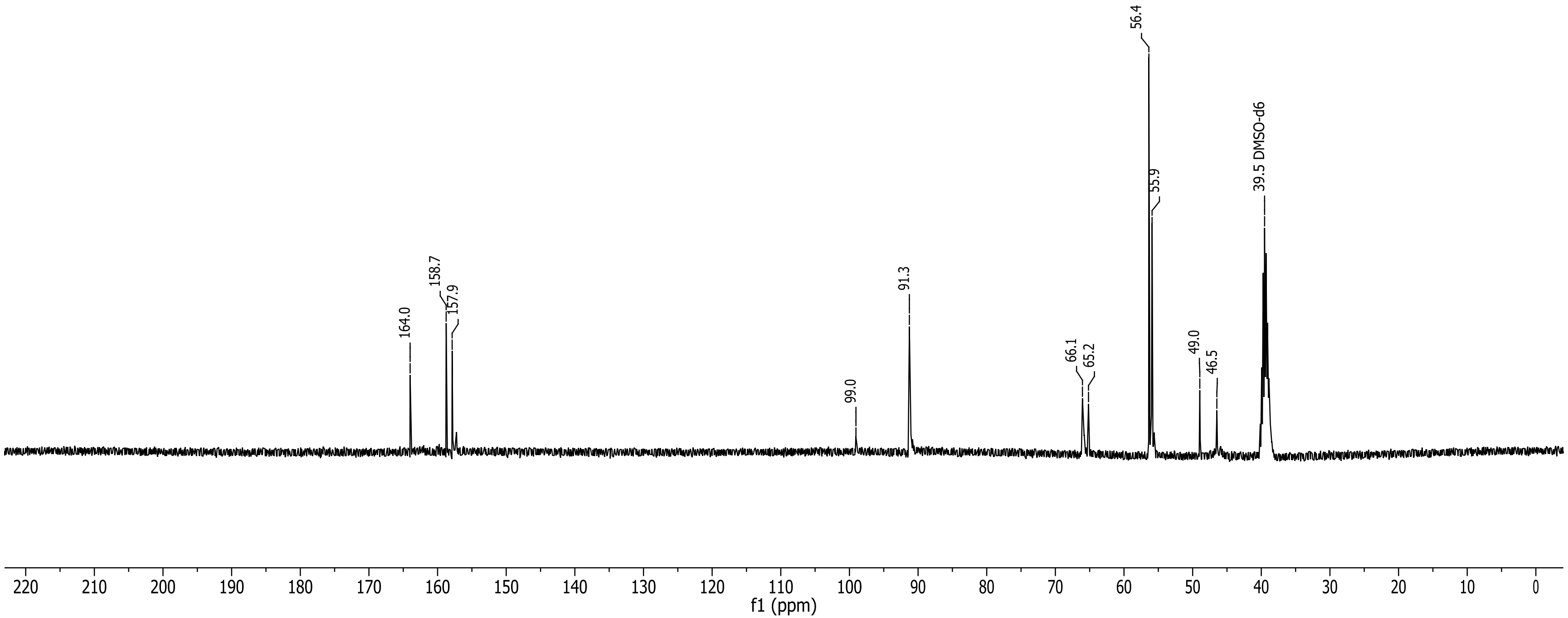

3h

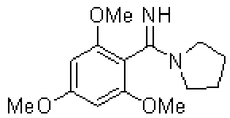

Origin	Varian
Spectrometer	mercury
Solvent	cdcl3
Temperature	25.0
Pulse Sequence	s2pul
Experiment	1D
Number of Scans	32
Relaxation Delay	4.0000
Spectrometer Frequency	399.86
Nucleus	1H

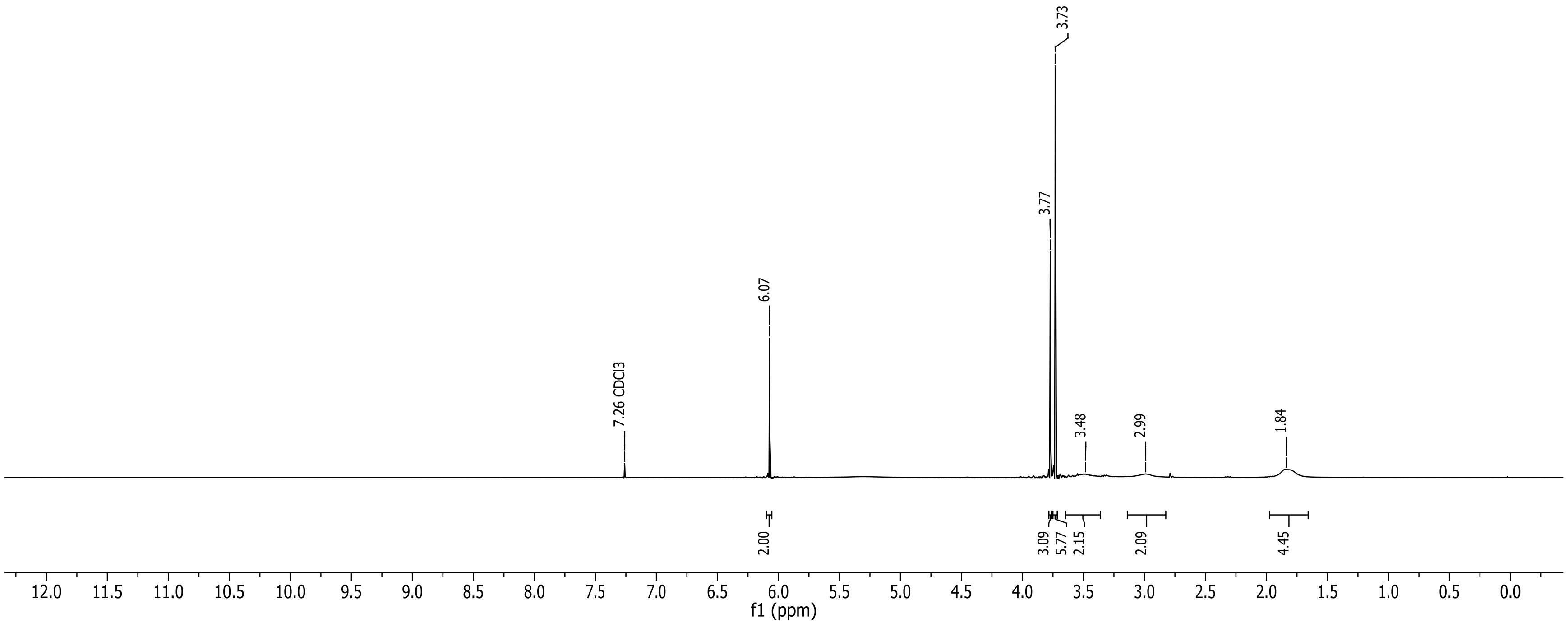

3h

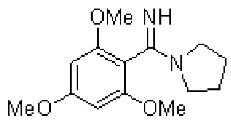

Origin Varian
Spectrometer mercury
Solvent cdcl3
Temperature 25.0
Pulse Sequence s2pul
Experiment 1D
Number of Scans 512
Relaxation Delay 1.0000
Spectrometer Frequency 100.55
Nucleus 13C

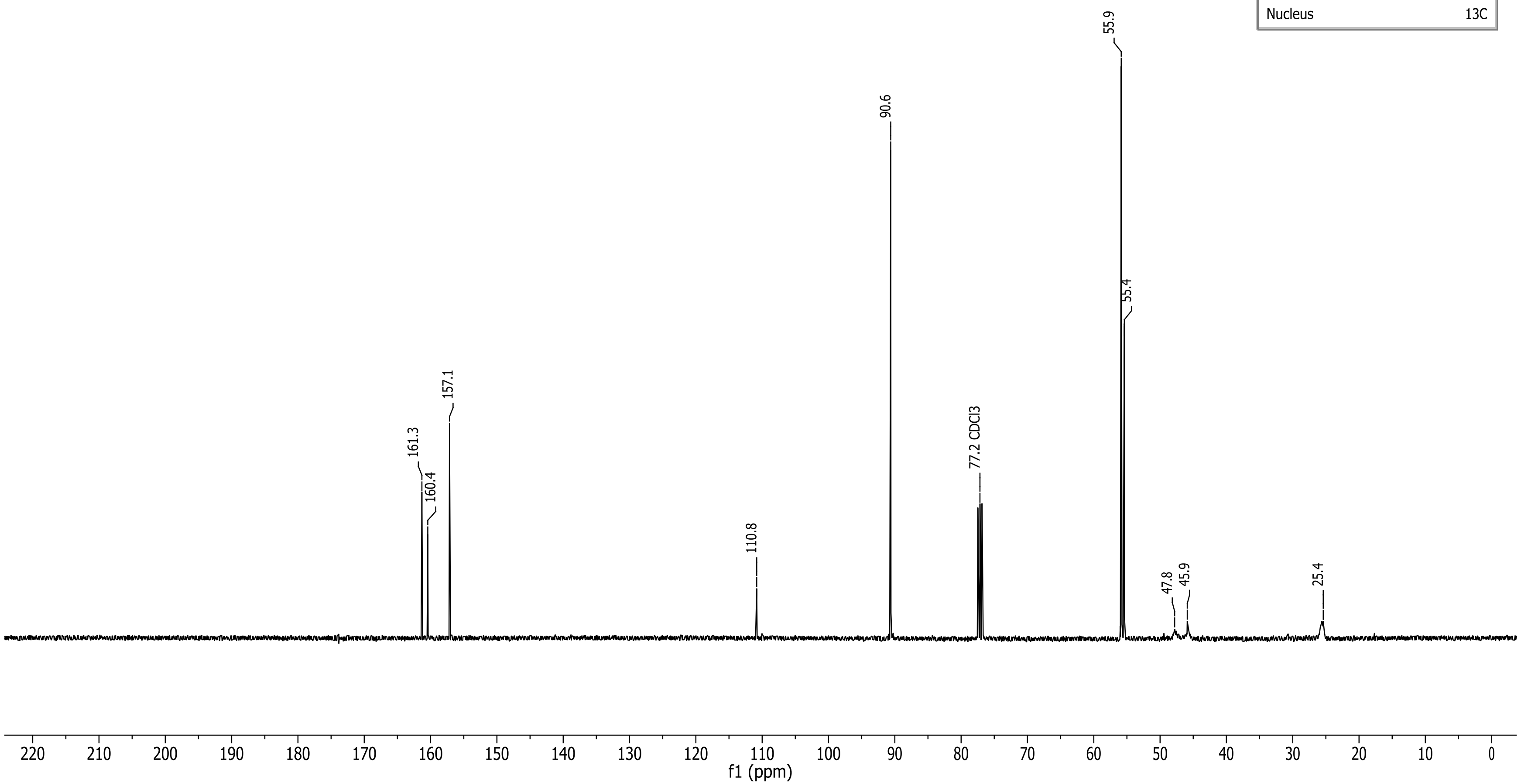

3h

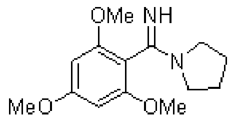

HCl added

Origin	Varian
Spectrometer	mercury
Solvent	dmso
Temperature	25.0
Pulse Sequence	s2pul
Experiment	1D
Number of Scans	1024
Relaxation Delay	1.0000
Spectrometer Frequency	100.56
Nucleus	13C

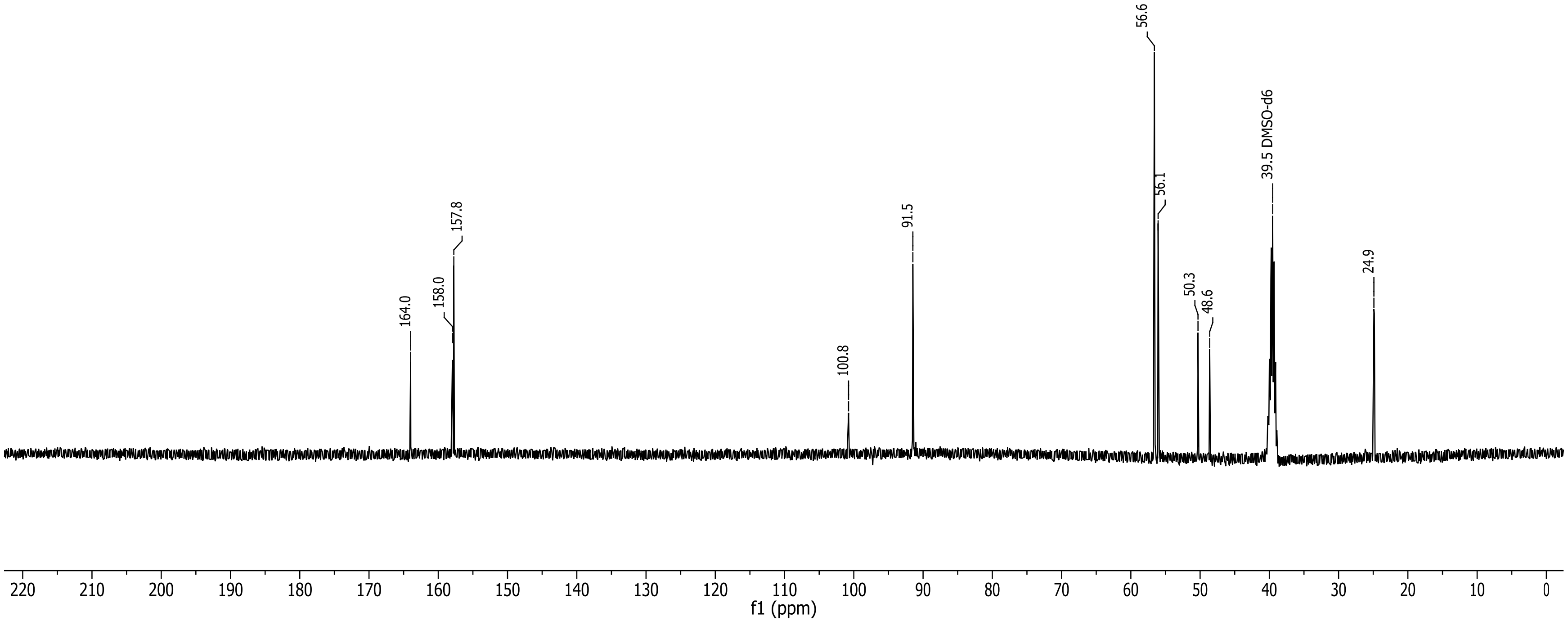

3i

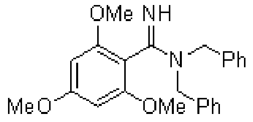
OMe NH
N
Ph
MeO
OMe
Ph

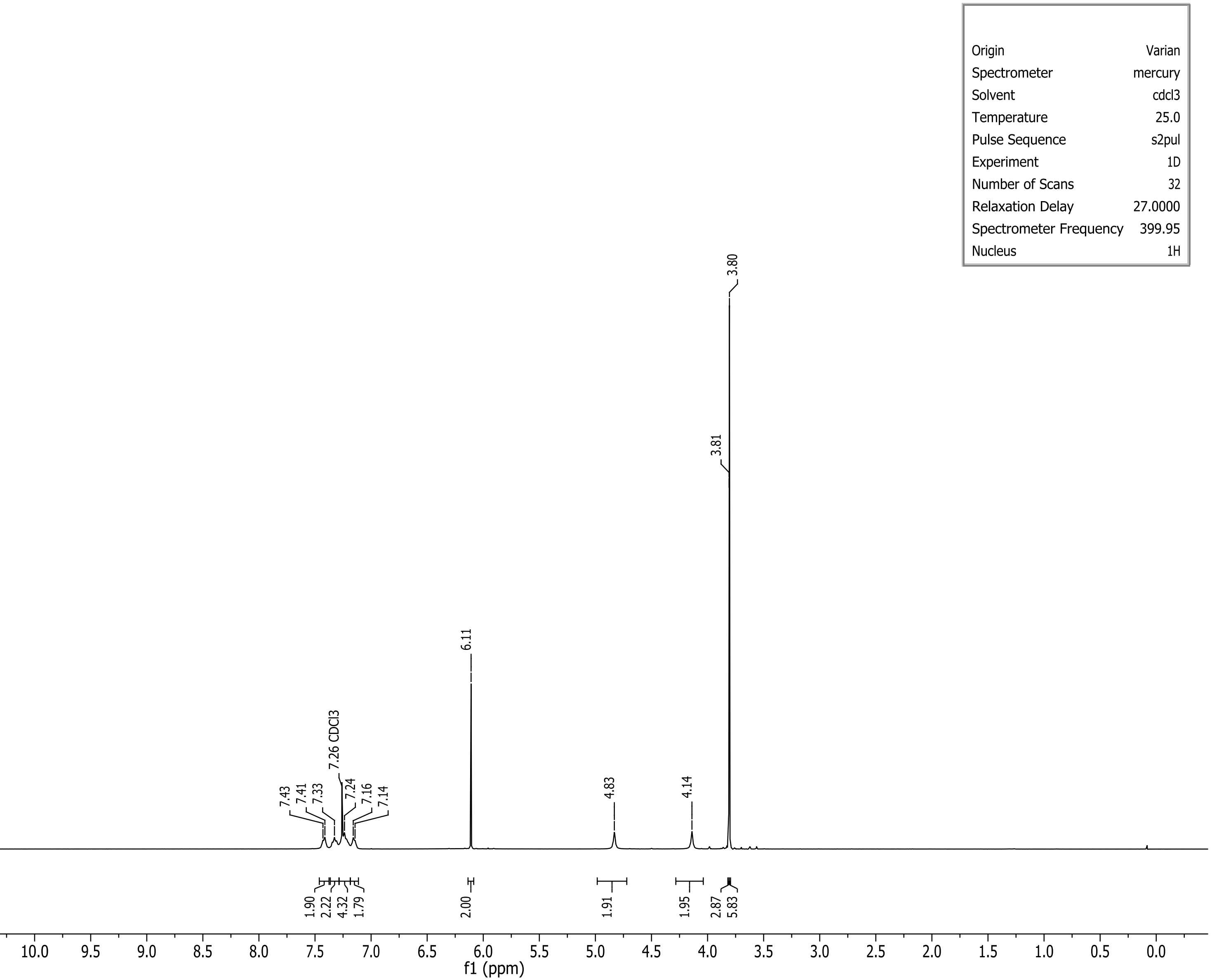
Origin Varian
Spectrometer mercury
Solvent cdcl3
Temperature 25.0
Pulse Sequence s2pul
Experiment 1D
Number of Scans 32
Relaxation Delay 27.0000
Spectrometer Frequency 399.95
Nucleus 1H
3.80
3.81
6.11
7.26 CDCl3
7.43
7.41
7.33
7.24
7.16
7.14
4.83
4.14
1.90
2.22
4.32
1.79
2.00
1.91
1.95
2.87
5.83
12.0 11.5 11.0 10.5 10.0 9.5 9.0 8.5 8.0 7.5 7.0 6.5 6.0 5.5 5.0 4.5 4.0 3.5 3.0 2.5 2.0 1.5 1.0 0.5 0.0
f1 (ppm)

3i

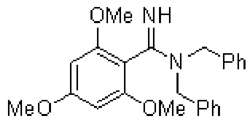

Origin	Varian
Spectrometer	mercury
Solvent	cdcl3
Temperature	25.0
Pulse Sequence	s2pul
Experiment	1D
Number of Scans	1024
Relaxation Delay	1.0000
Spectrometer Frequency	100.55
Nucleus	13C

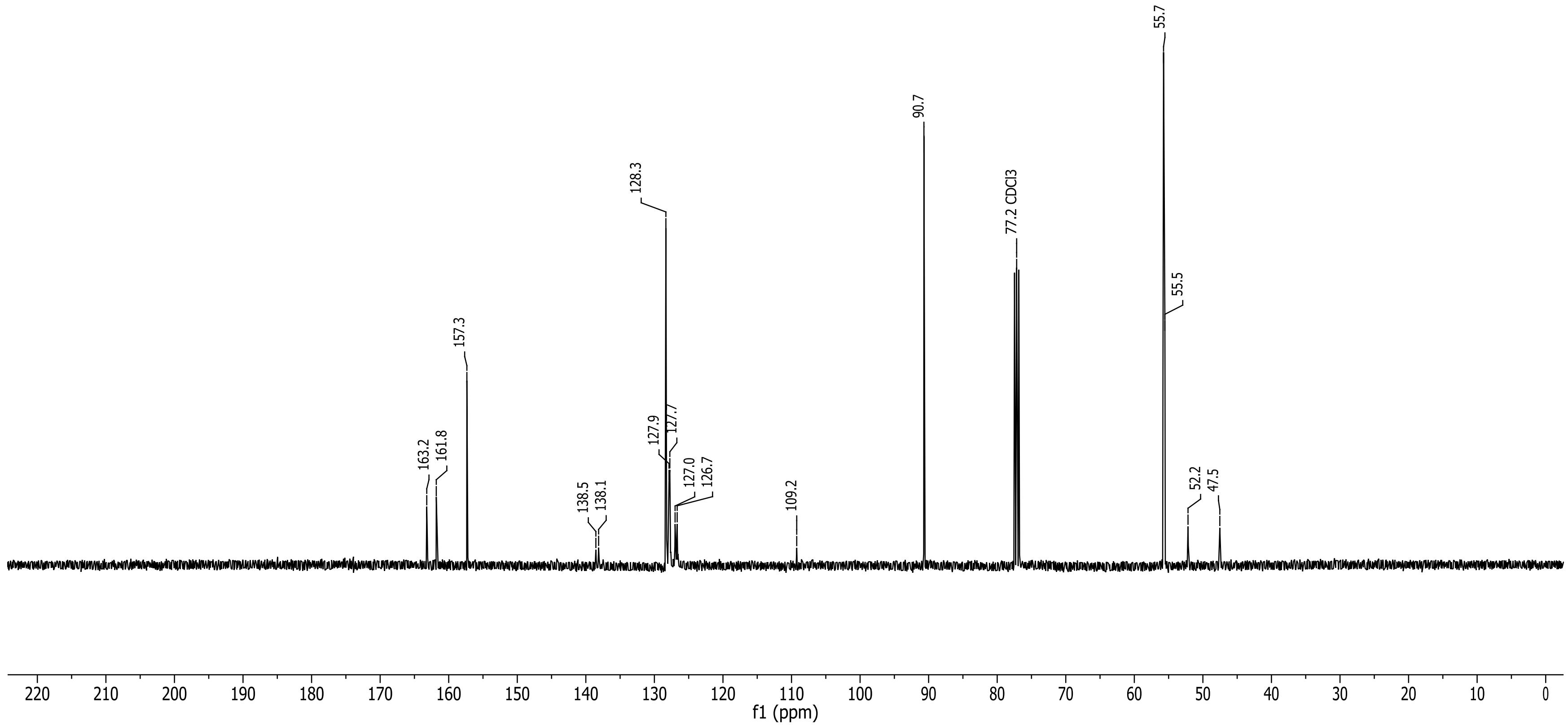

3k

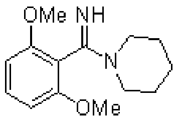

Origin	Varian
Spectrometer	mercury
Solvent	cdcl3
Temperature	25.0
Pulse Sequence	s2pul
Experiment	1D
Number of Scans	32
Relaxation Delay	27.0000
Spectrometer Frequency	399.95
Nucleus	1H

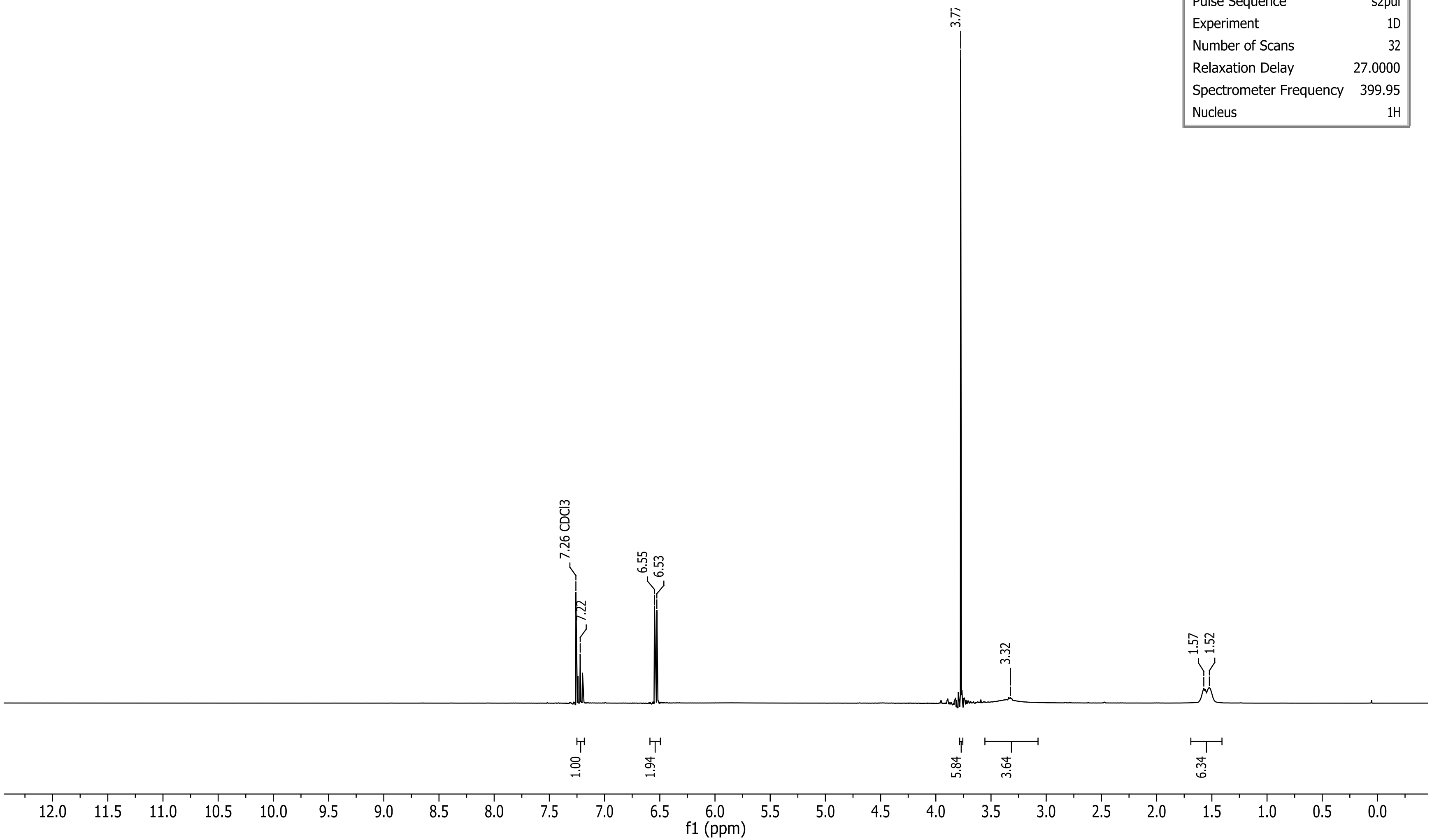

3k

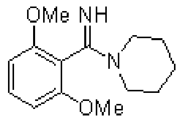

Origin	Varian
Spectrometer	mercury
Solvent	cdcl3
Temperature	25.0
Pulse Sequence	s2pul
Experiment	1D
Number of Scans	4096
Relaxation Delay	1.0000
Spectrometer Frequency	100.58
Nucleus	13C

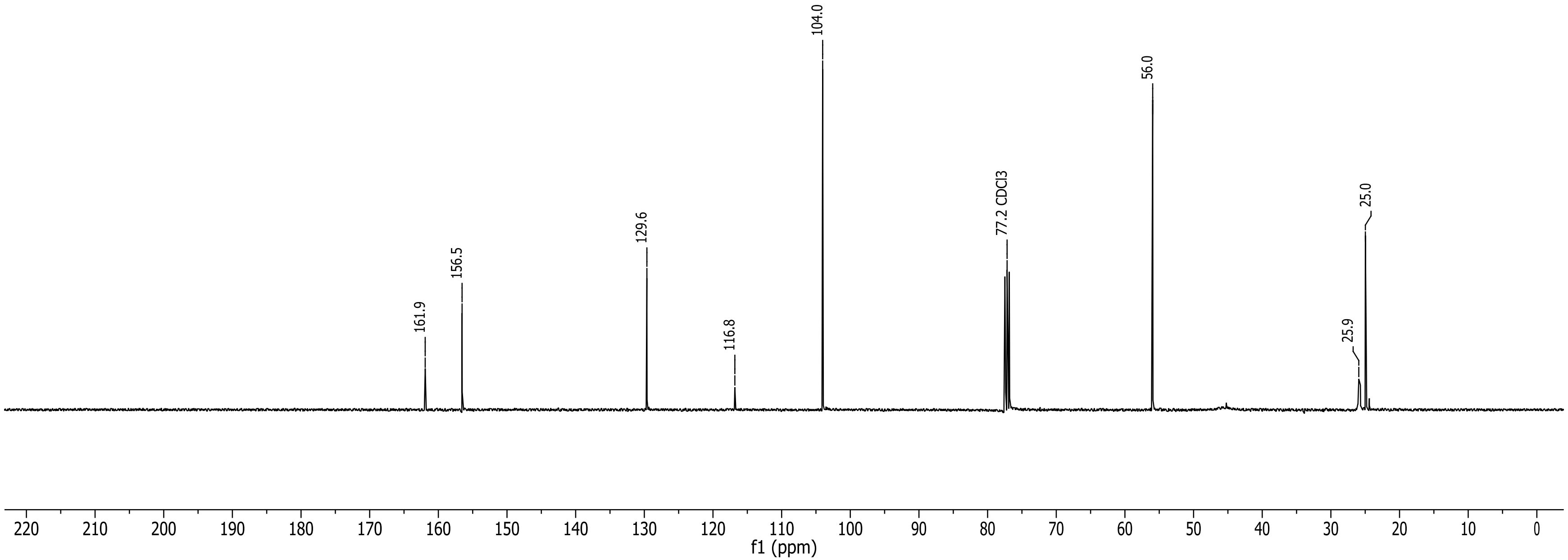

3k

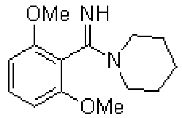

HCl added

Origin	Varian
Spectrometer	mercury
Solvent	cd3od
Temperature	25.0
Pulse Sequence	s2pul
Experiment	1D
Number of Scans	1024
Relaxation Delay	1.0000
Spectrometer Frequency	100.58
Nucleus	13C

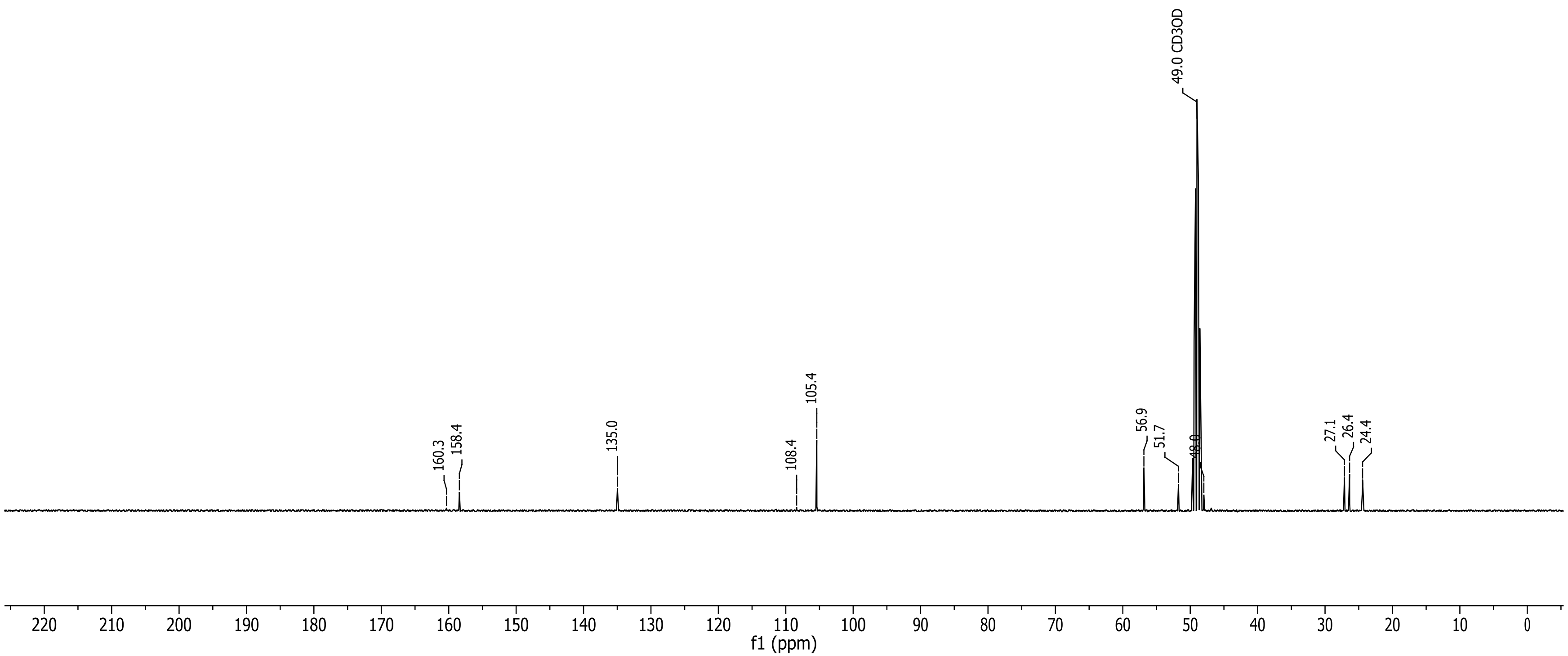

3l

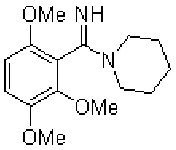

Origin	Varian
Spectrometer	mercury
Solvent	cdcl3
Temperature	25.0
Pulse Sequence	s2pul
Experiment	1D
Number of Scans	32
Relaxation Delay	27.0000
Spectrometer Frequency	399.86
Nucleus	1H

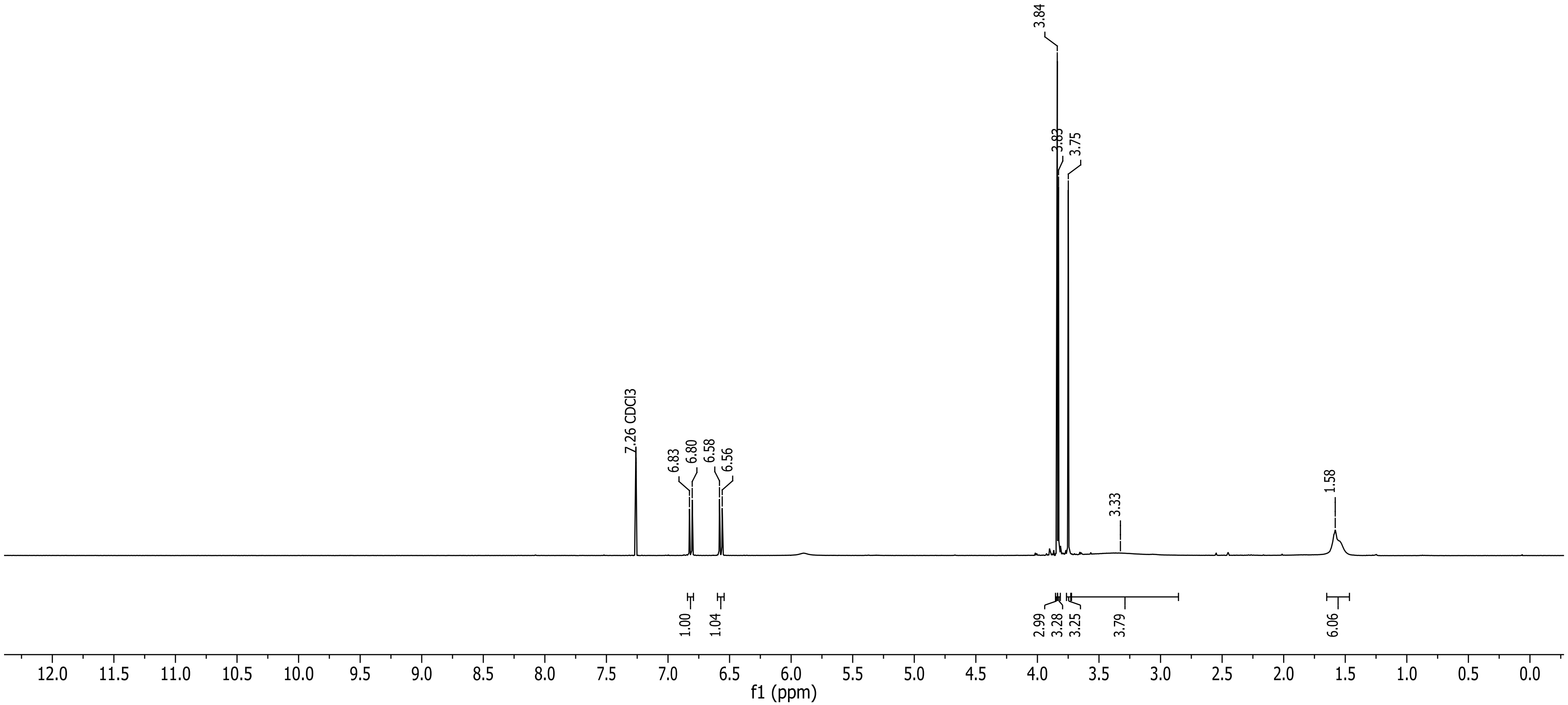

3l

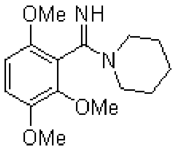

Origin	Varian
Spectrometer	mercury
Solvent	cdcl3
Temperature	25.0
Pulse Sequence	s2pul
Experiment	1D
Number of Scans	4096
Relaxation Delay	1.0000
Spectrometer Frequency	100.55
Nucleus	13C

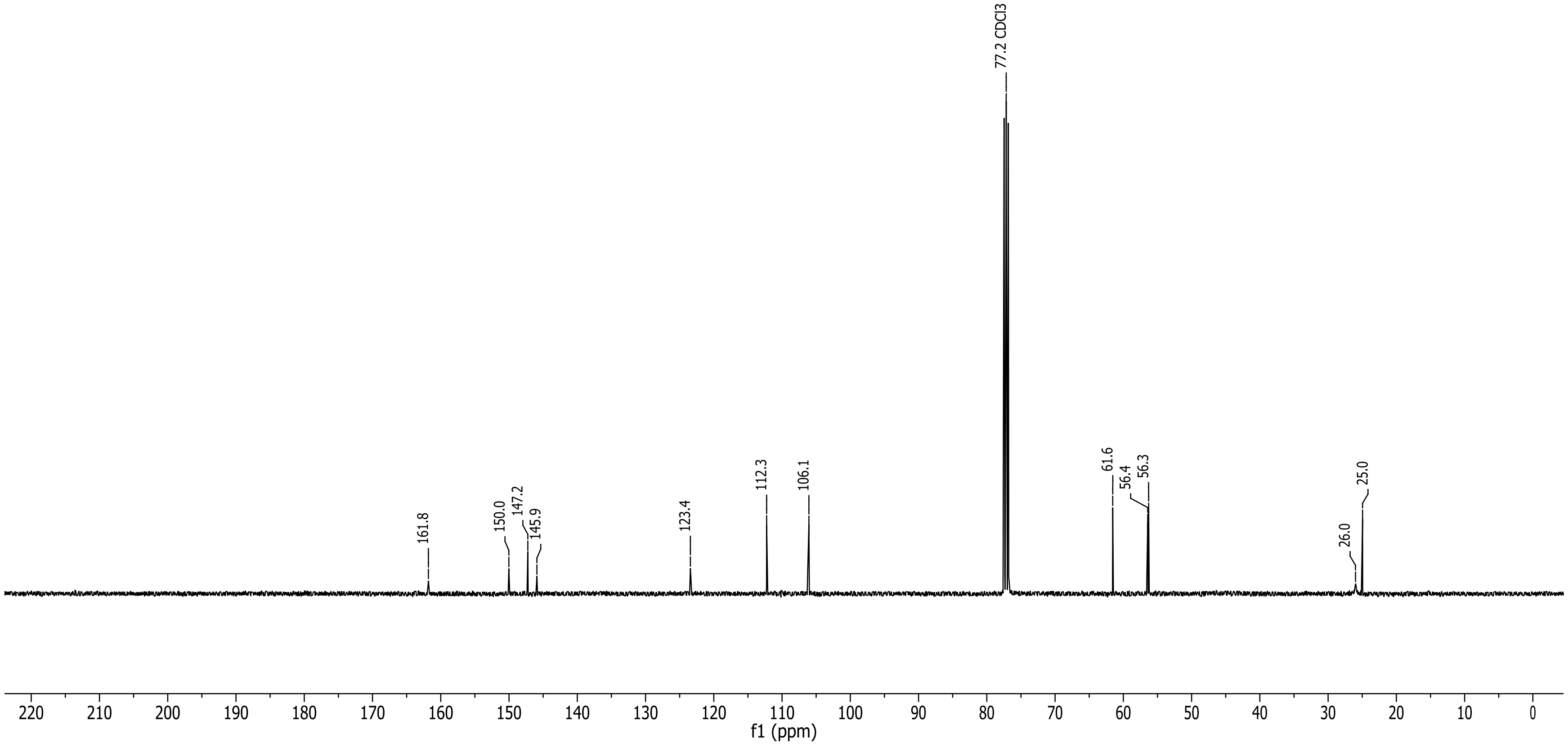

3l

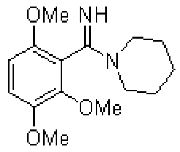

HCl added

Origin	Varian
Spectrometer	mercury
Solvent	cd3od
Temperature	25.0
Pulse Sequence	s2pul
Experiment	1D
Number of Scans	4096
Relaxation Delay	1.0000
Spectrometer Frequency	100.58
Nucleus	13C

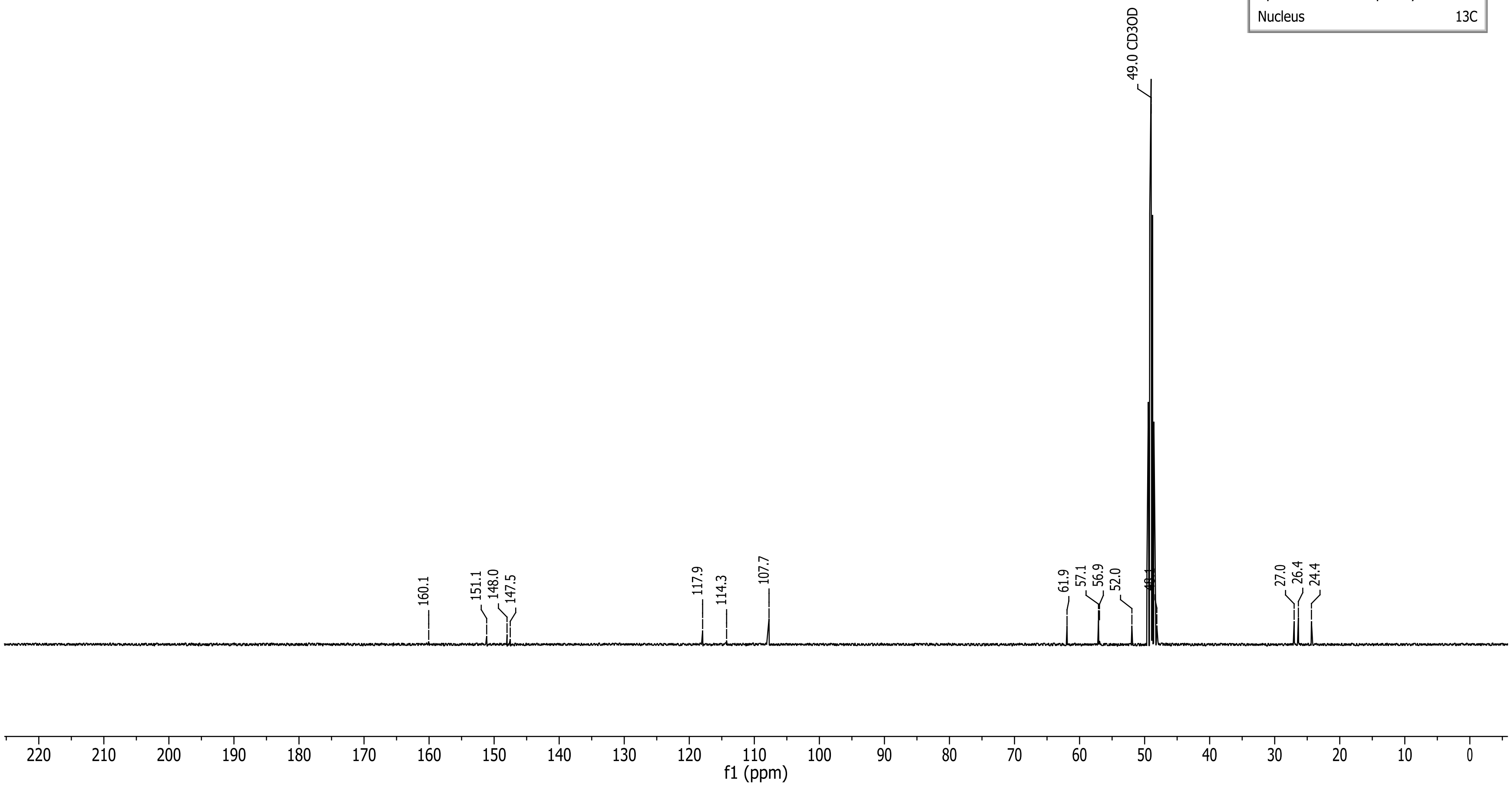

3m

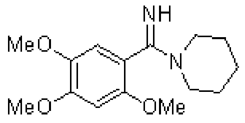

Origin	Varian
Spectrometer	mercury
Solvent	cdcl3
Temperature	25.0
Pulse Sequence	s2pul
Experiment	1D
Number of Scans	32
Relaxation Delay	27.0000
Spectrometer Frequency	399.94
Nucleus	1H

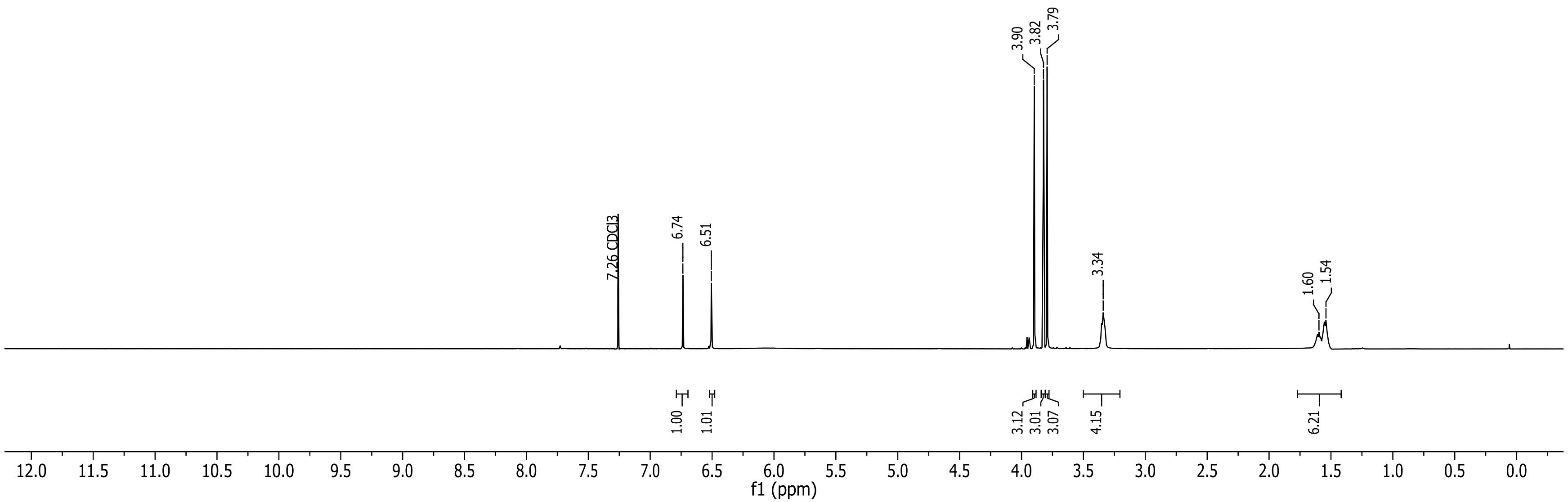

3m

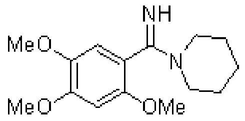

Origin	Varian
Spectrometer	mercury
Solvent	cdcl3
Temperature	25.0
Pulse Sequence	s2pul
Experiment	1D
Number of Scans	1024
Relaxation Delay	1.0000
Spectrometer Frequency	100.58
Nucleus	13C

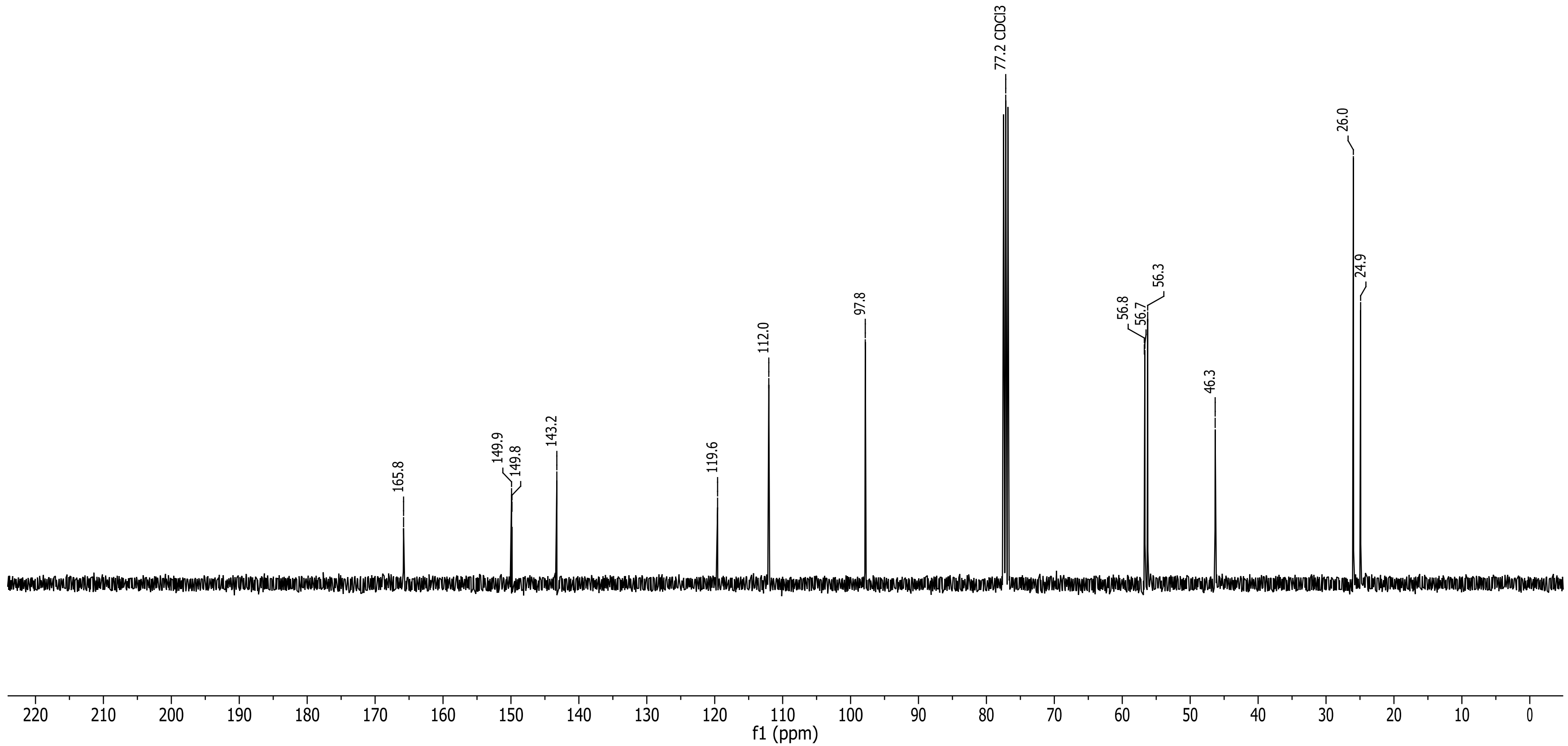

3n

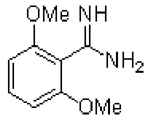

Origin	Varian
Spectrometer	mercury
Solvent	cdcl3
Temperature	25.0
Pulse Sequence	s2pul
Experiment	1D
Number of Scans	32
Relaxation Delay	27.0000
Spectrometer Frequency	399.94
Nucleus	1H

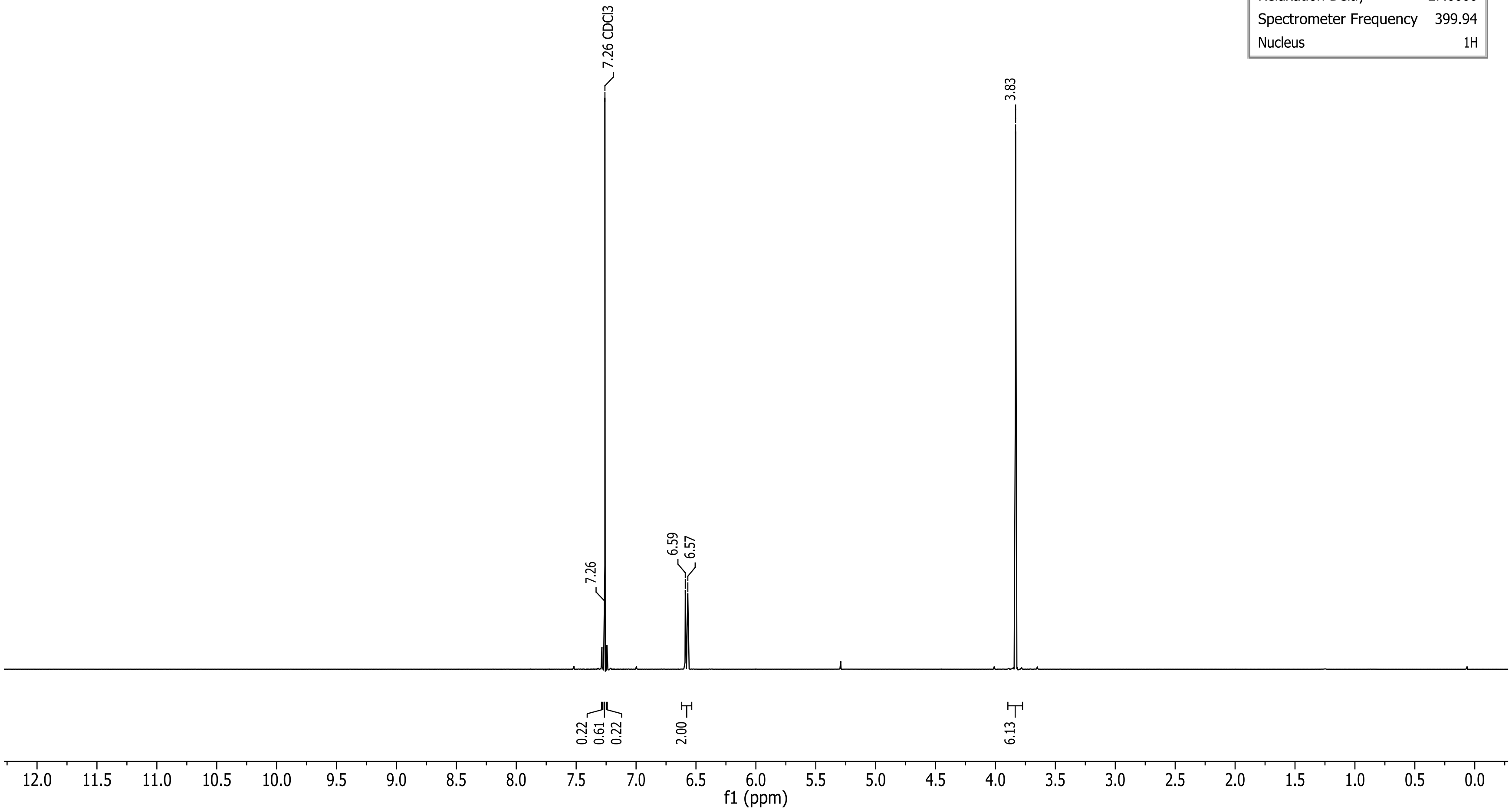

3n

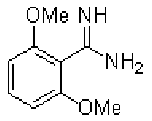

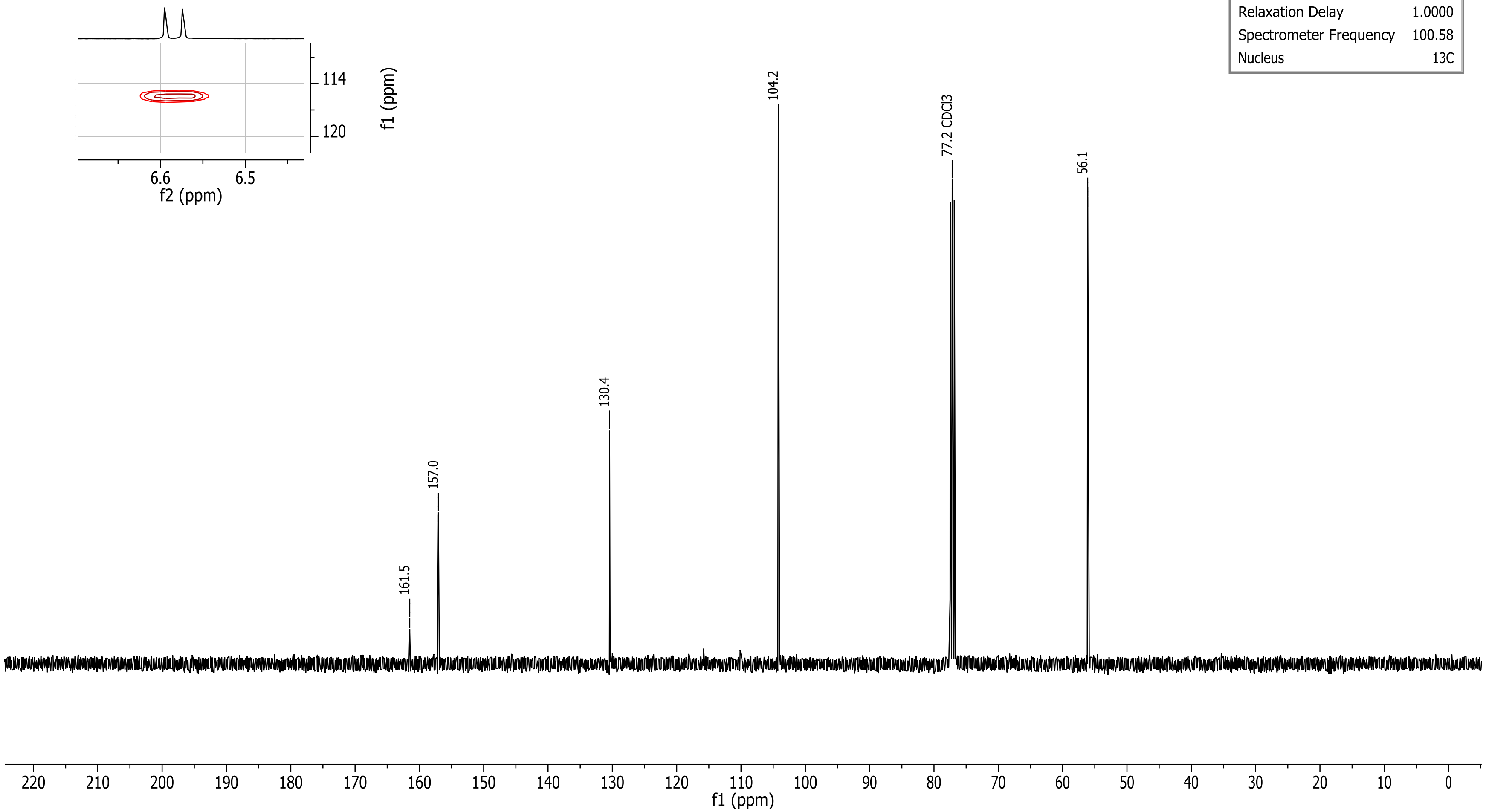

Origin	Varian
Spectrometer	mercury
Solvent	cdcl3
Temperature	25.0
Pulse Sequence	s2pul
Experiment	1D
Number of Scans	1024
Relaxation Delay	1.0000
Spectrometer Frequency	100.58
Nucleus	13C

3o

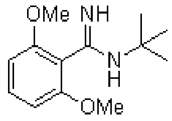

Origin	Varian
Spectrometer	mercury
Solvent	cdcl3
Temperature	25.0
Pulse Sequence	s2pul
Experiment	1D
Number of Scans	32
Relaxation Delay	27.0000
Spectrometer Frequency	399.86
Nucleus	1H

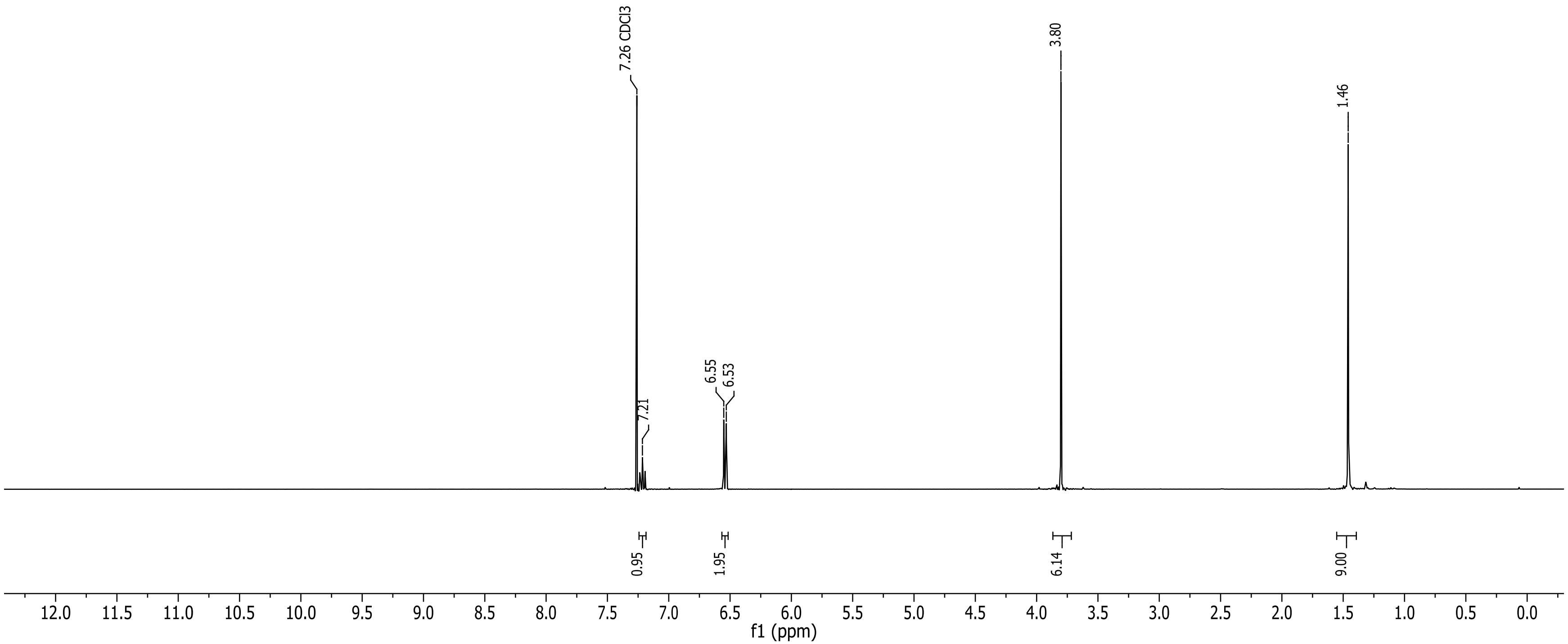

3o

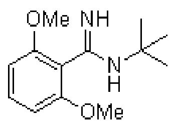

Origin	Varian
Spectrometer	mercury
Solvent	cdcl3
Temperature	25.0
Pulse Sequence	s2pul
Experiment	1D
Number of Scans	4096
Relaxation Delay	1.0000
Spectrometer Frequency	100.55
Nucleus	13C

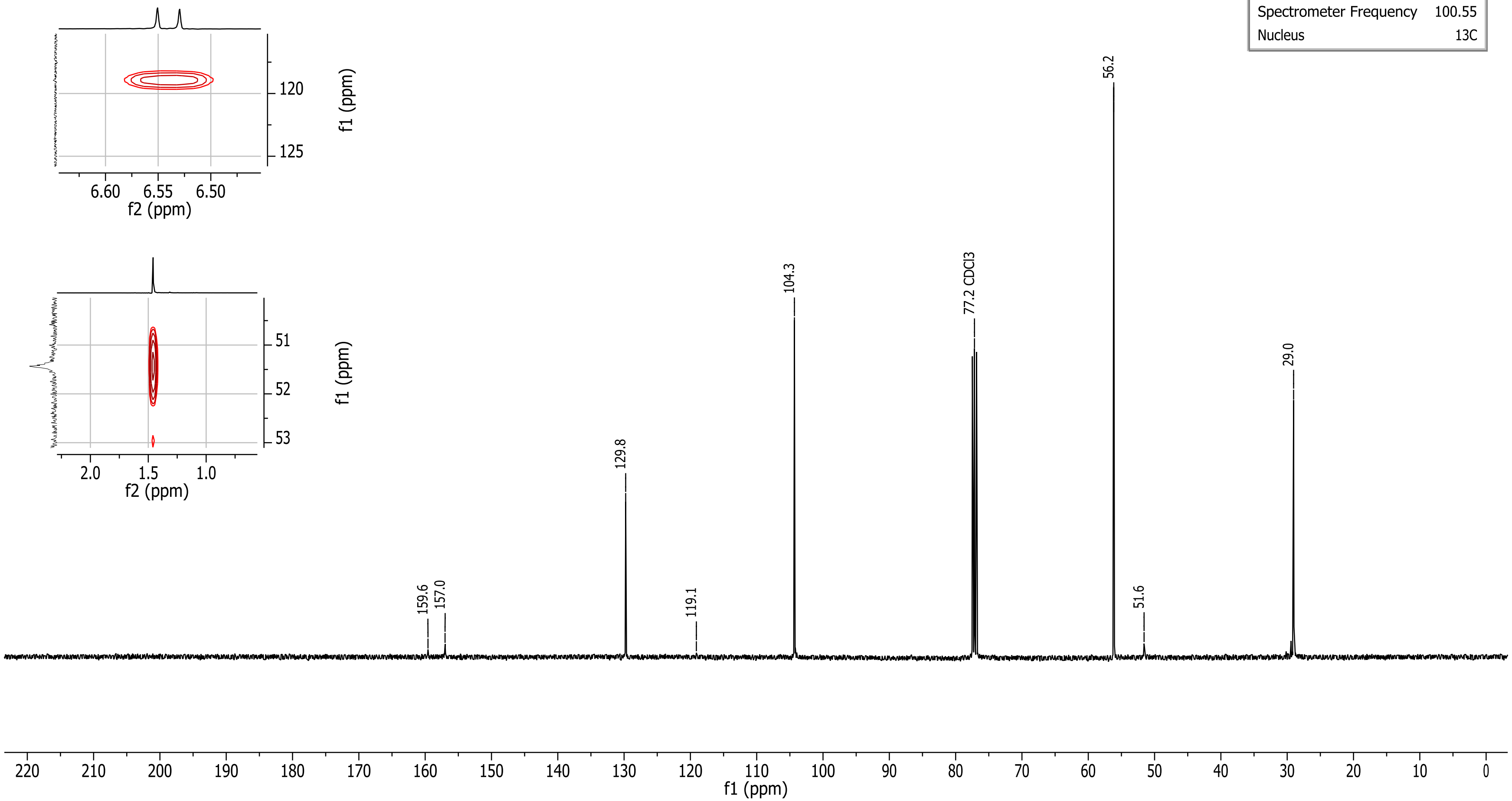

3p

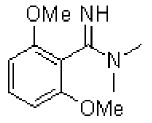
OMe NH
N
OMe

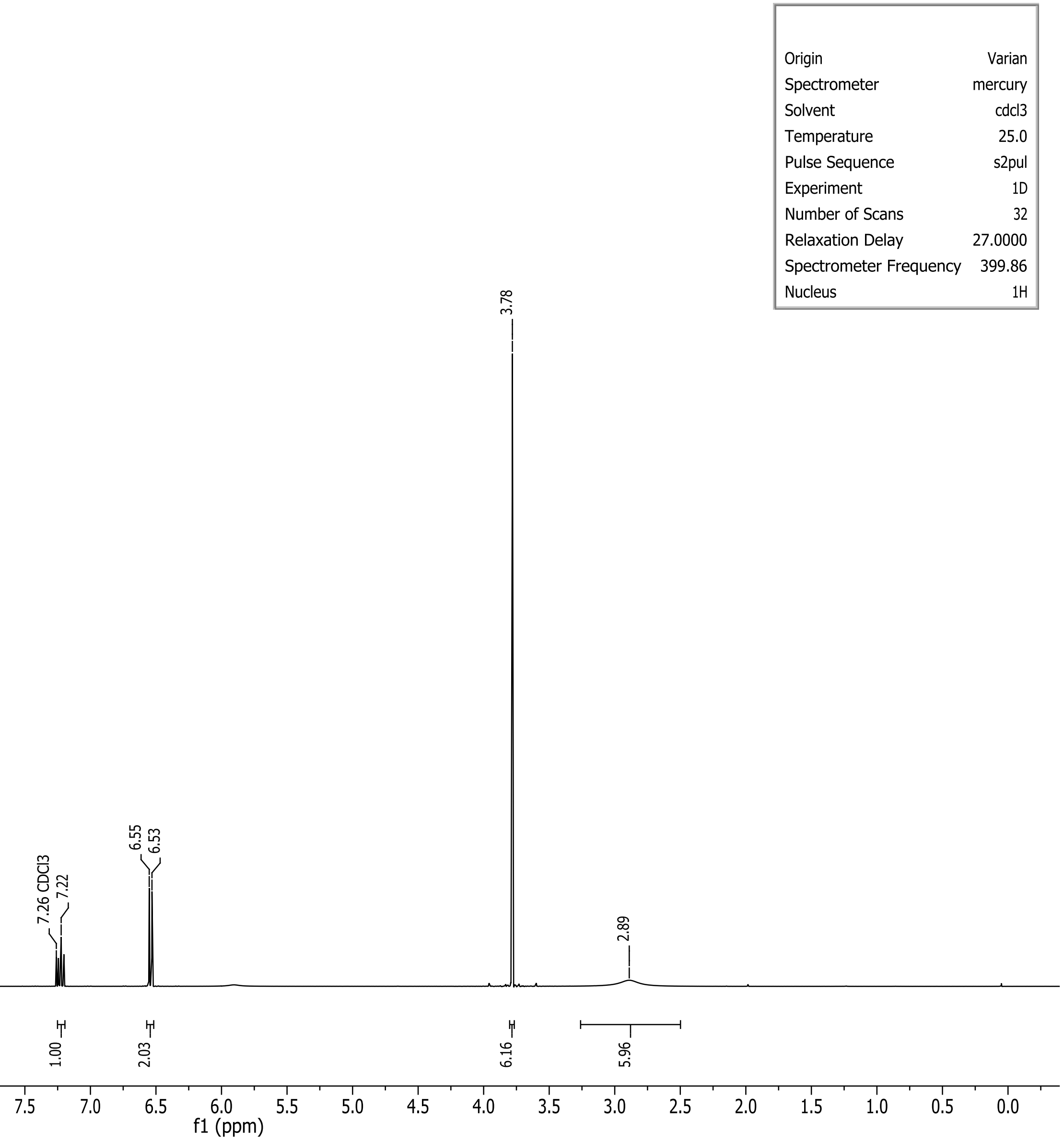
Origin Varian
Spectrometer mercury
Solvent cdcl3
Temperature 25.0
Pulse Sequence s2pul
Experiment 1D
Number of Scans 32
Relaxation Delay 27.0000
Spectrometer Frequency 399.86
Nucleus 1H
3.78
7.26 CDCl3
7.22
6.55
6.53
2.89
1.00
2.03
6.16
5.96
12.0 11.5 11.0 10.5 10.0 9.5 9.0 8.5 8.0 7.5 7.0 6.5 6.0 5.5 5.0 4.5 4.0 3.5 3.0 2.5 2.0 1.5 1.0 0.5 0.0
f1 (ppm)

3p

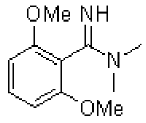

Origin	Varian
Spectrometer	mercury
Solvent	cdcl3
Temperature	25.0
Pulse Sequence	s2pul
Experiment	1D
Number of Scans	1024
Relaxation Delay	1.0000
Spectrometer Frequency	100.55
Nucleus	13C

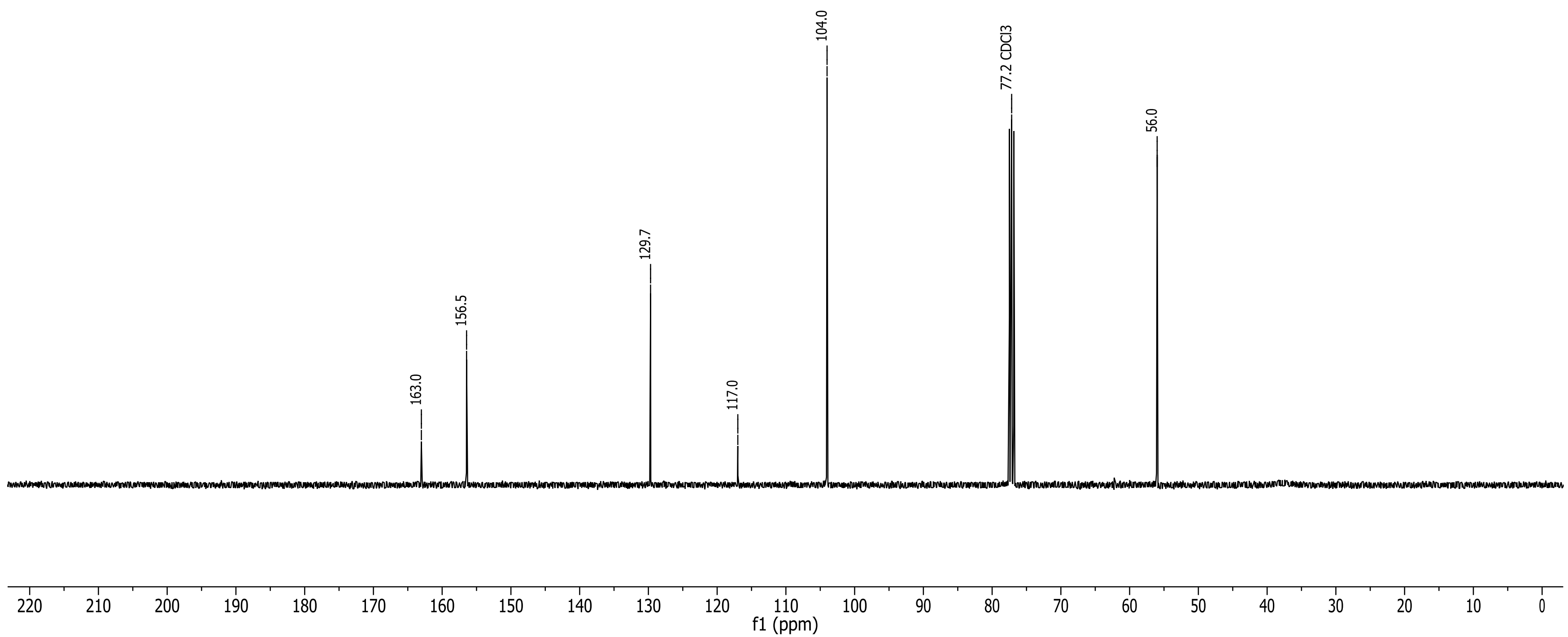

3p

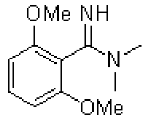

HCl added

Origin	Varian
Spectrometer	mercury
Solvent	cd3od
Temperature	25.0
Pulse Sequence	s2pul
Experiment	1D
Number of Scans	4096
Relaxation Delay	1.0000
Spectrometer Frequency	100.58
Nucleus	13C

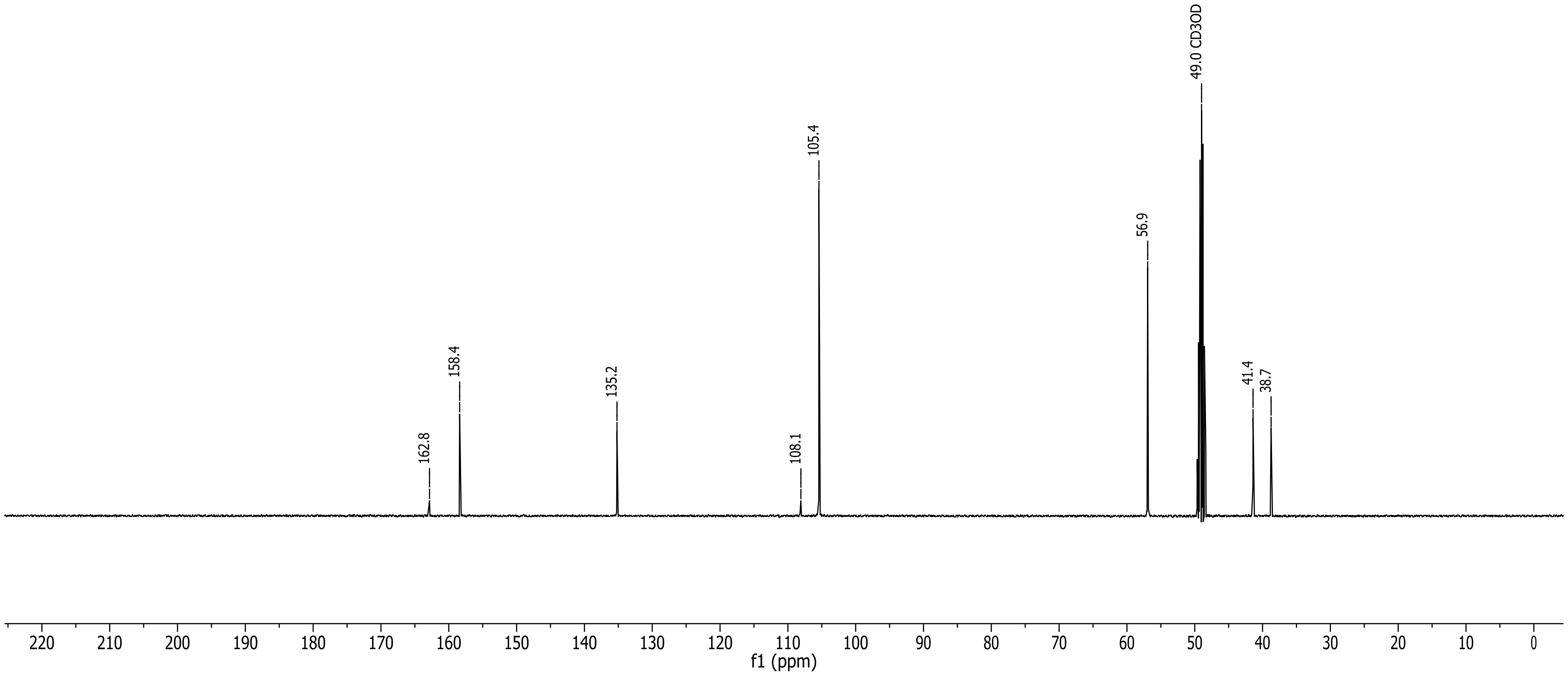

3q

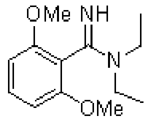

Origin	Varian
Spectrometer	mercury
Solvent	cdcl3
Temperature	25.0
Pulse Sequence	s2pul
Experiment	1D
Number of Scans	32
Relaxation Delay	4.0000
Spectrometer Frequency	399.95
Nucleus	1H

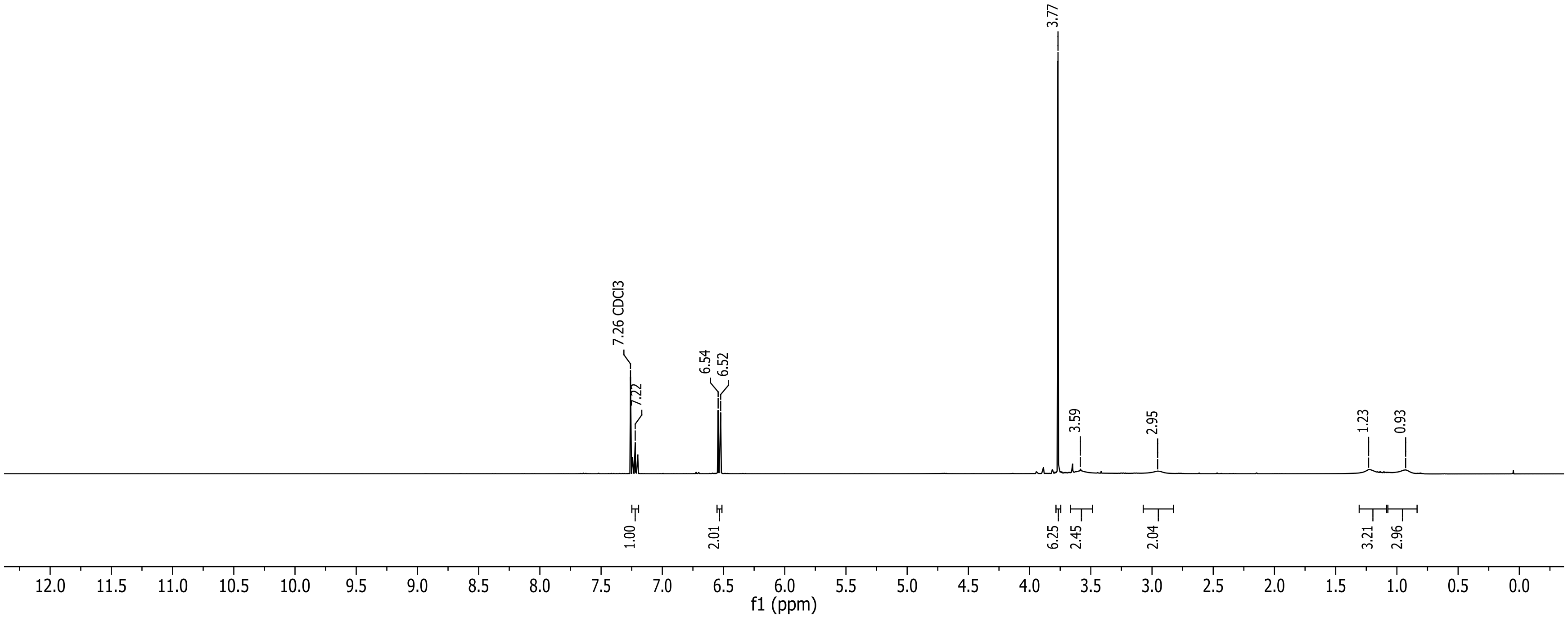

3q

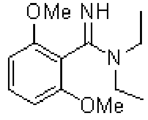

Origin	Varian
Spectrometer	mercury
Solvent	cdcl3
Temperature	25.0
Pulse Sequence	s2pul
Experiment	1D
Number of Scans	512
Relaxation Delay	1.0000
Spectrometer Frequency	100.58
Nucleus	13C

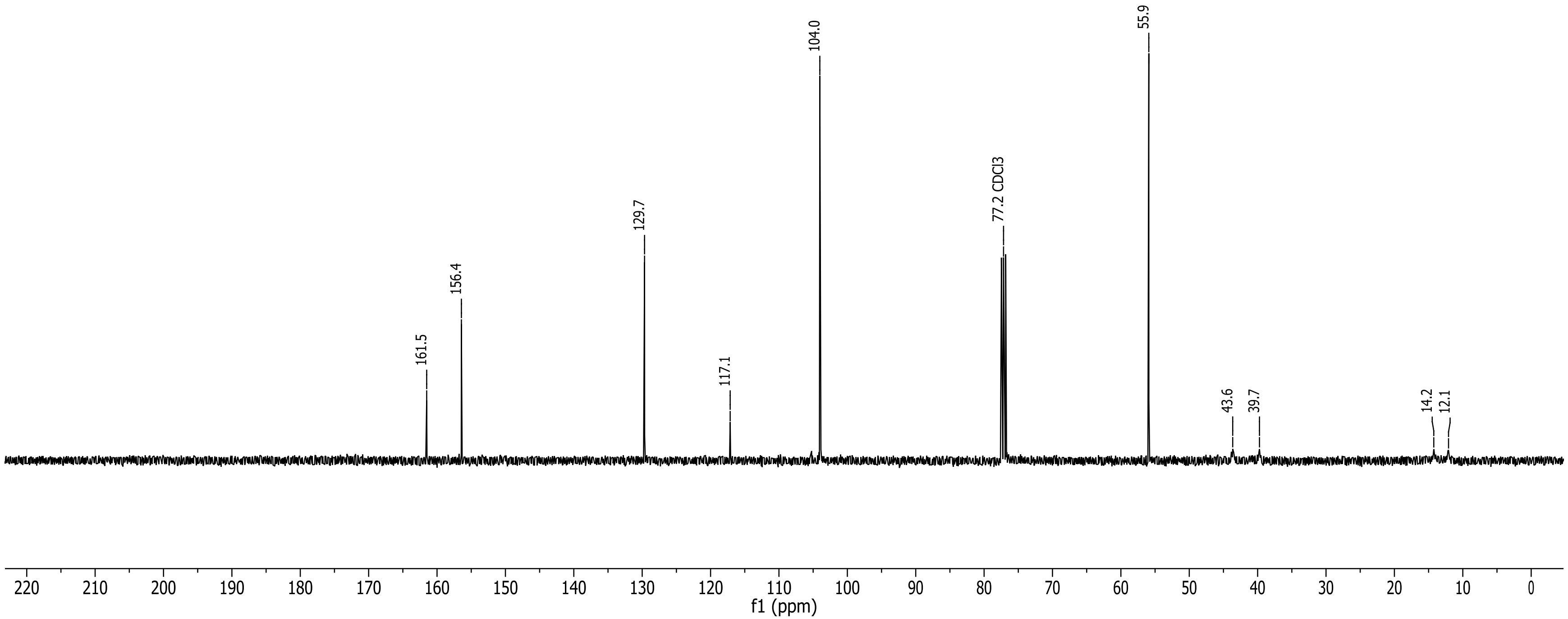

3r

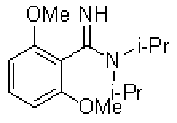

Origin	Varian
Spectrometer	mercury
Solvent	cdcl3
Temperature	25.0
Pulse Sequence	s2pul
Experiment	1D
Number of Scans	32
Relaxation Delay	27.0000
Spectrometer Frequency	399.95
Nucleus	1H

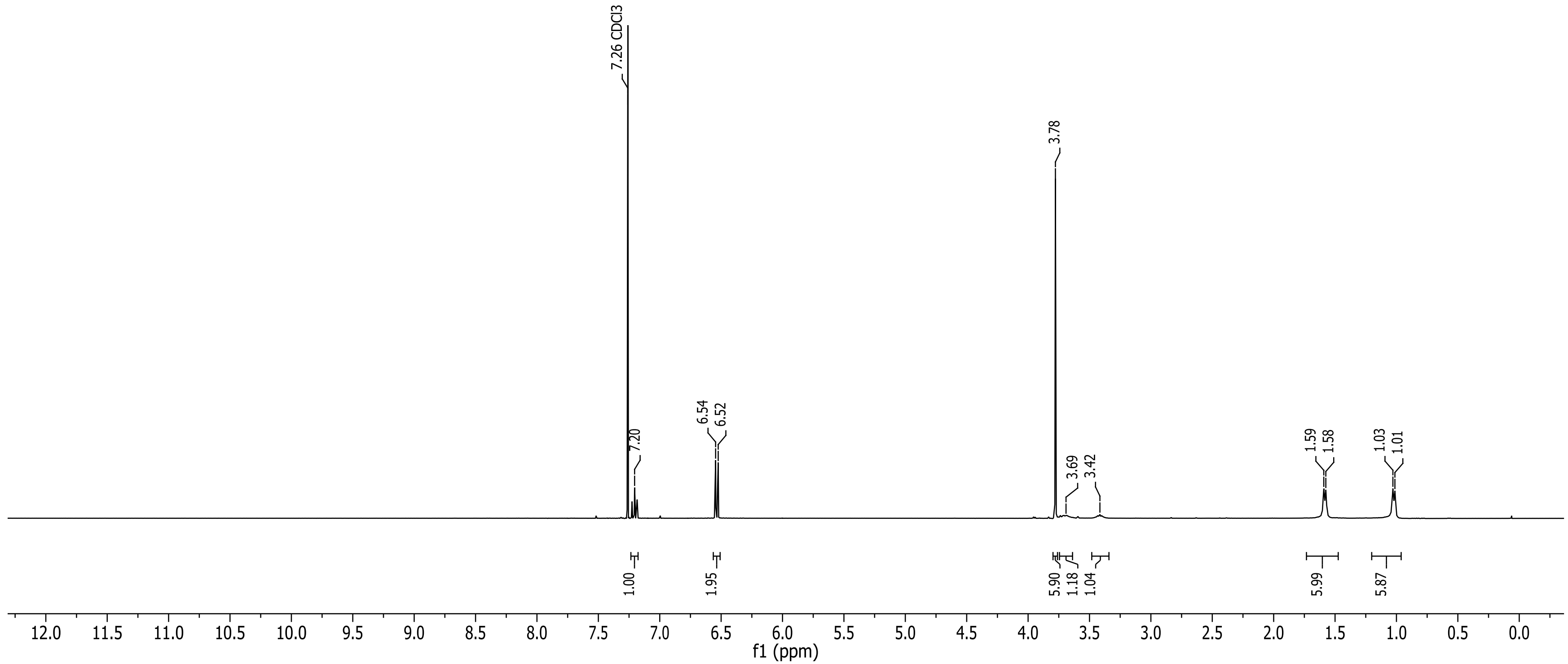

3r

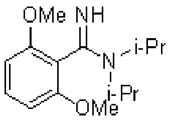

Origin	Varian
Spectrometer	mercury
Solvent	cdcl3
Temperature	25.0
Pulse Sequence	s2pul
Experiment	1D
Number of Scans	4096
Relaxation Delay	1.0000
Spectrometer Frequency	100.58
Nucleus	13C

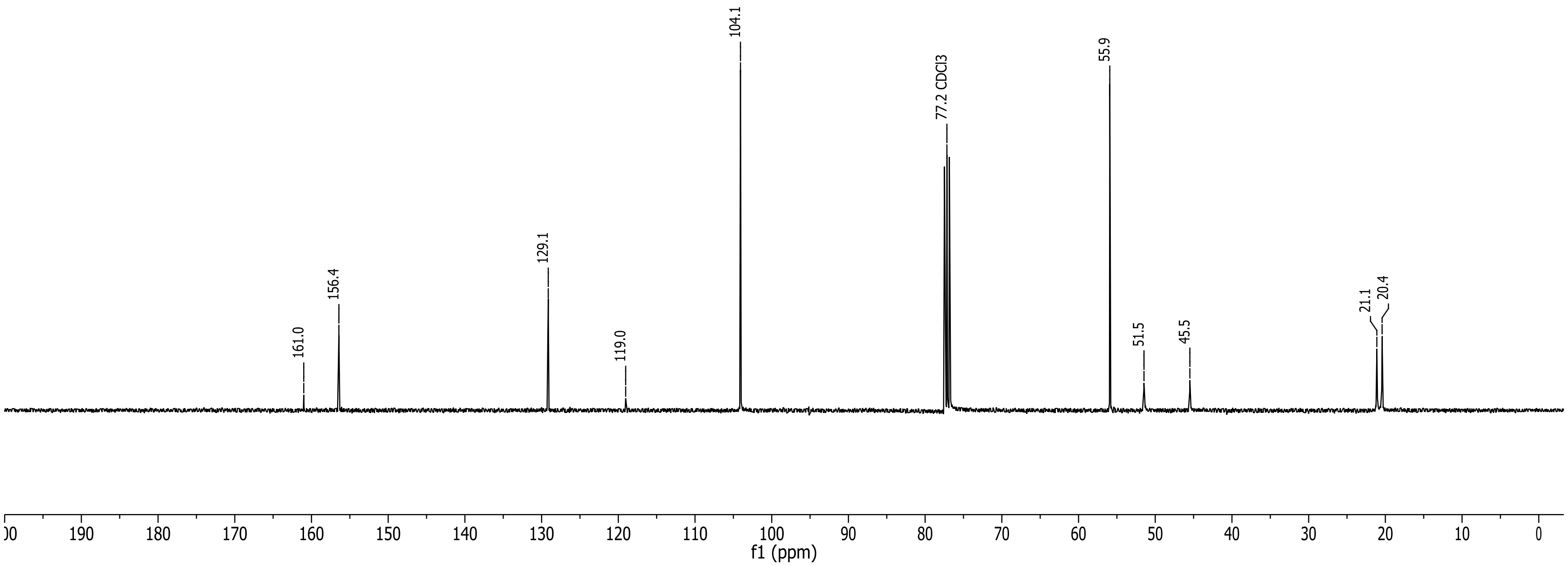

3s

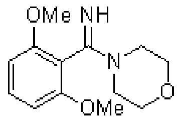

Origin	Varian
Spectrometer	mercury
Solvent	cdcl3
Temperature	25.0
Pulse Sequence	s2pul
Experiment	1D
Number of Scans	128
Relaxation Delay	1.0000
Spectrometer Frequency	399.86
Nucleus	1H

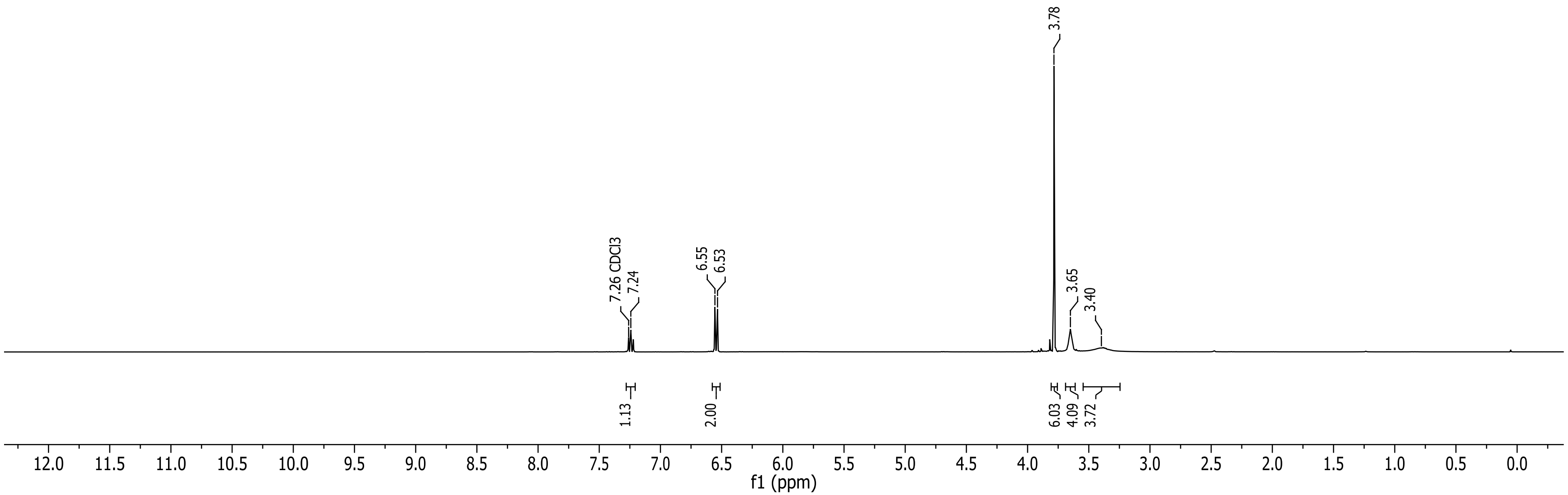

3s

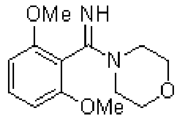

Origin	Varian
Spectrometer	mercury
Solvent	cdcl3
Temperature	25.0
Pulse Sequence	s2pul
Experiment	1D
Number of Scans	1000
Relaxation Delay	1.0000
Spectrometer Frequency	100.55
Nucleus	13C

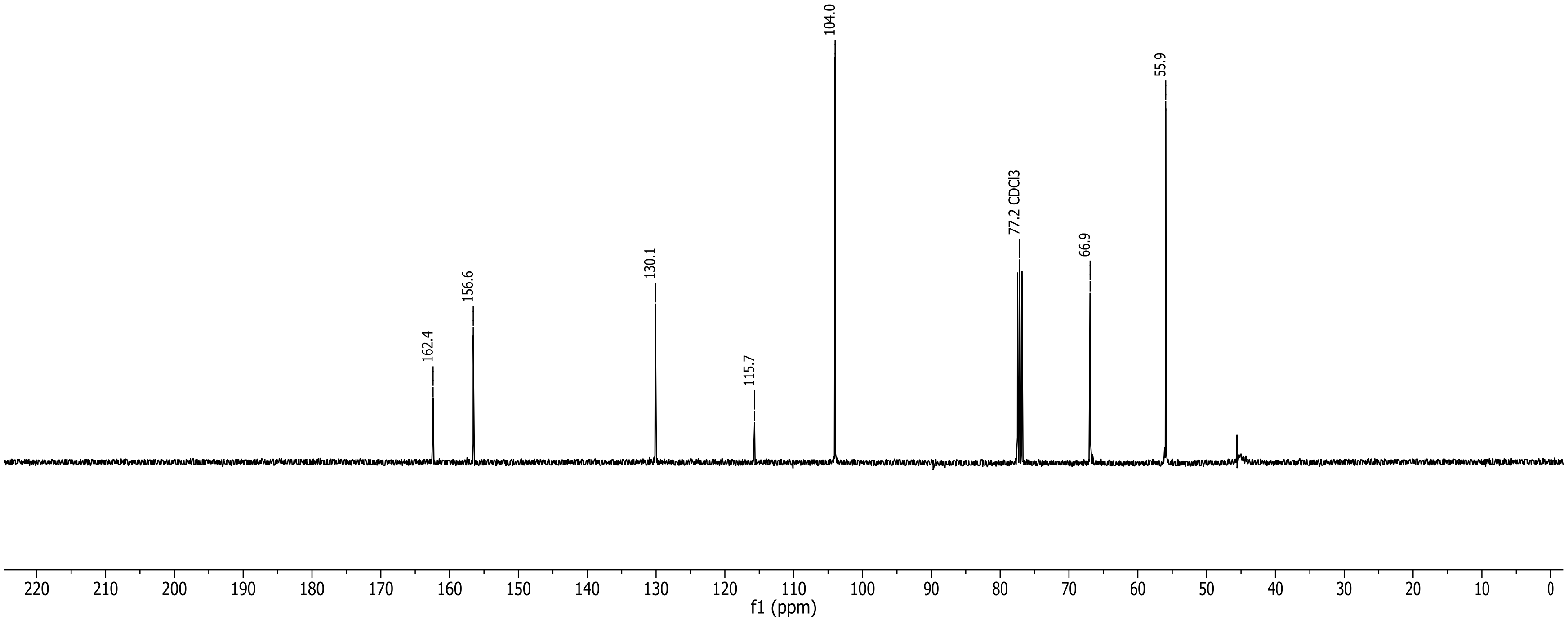

3s

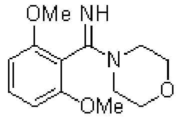

HCl added

Origin	Varian
Spectrometer	mercury
Solvent	cd3od
Temperature	25.0
Pulse Sequence	s2pul
Experiment	1D
Number of Scans	1024
Relaxation Delay	1.0000
Spectrometer Frequency	100.58
Nucleus	13C

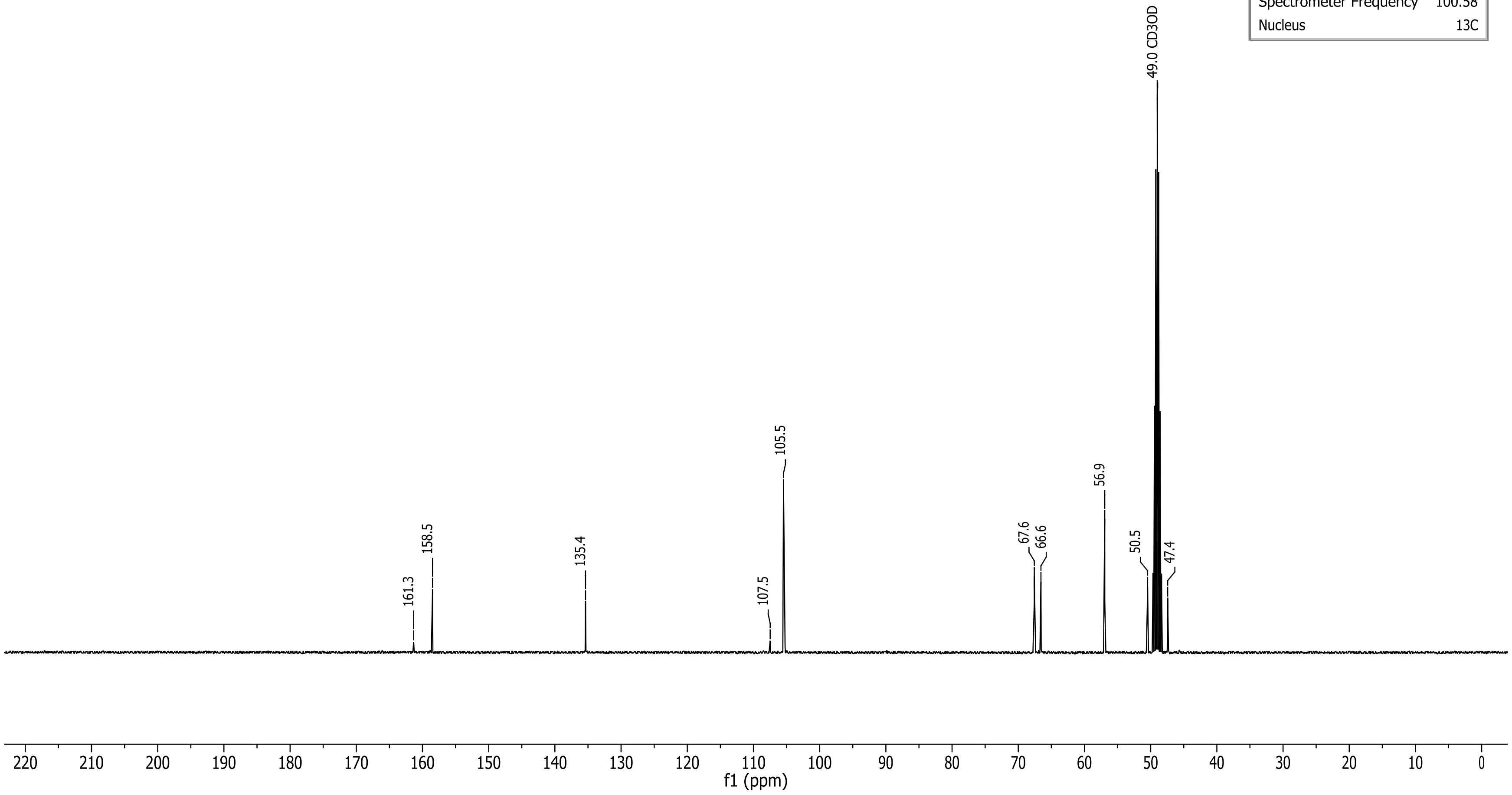

3t

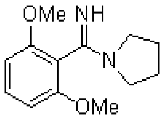

Origin	Varian
Spectrometer	mercury
Solvent	cdcl3
Temperature	25.0
Pulse Sequence	s2pul
Experiment	1D
Number of Scans	32
Relaxation Delay	27.0000
Spectrometer Frequency	399.95
Nucleus	1H

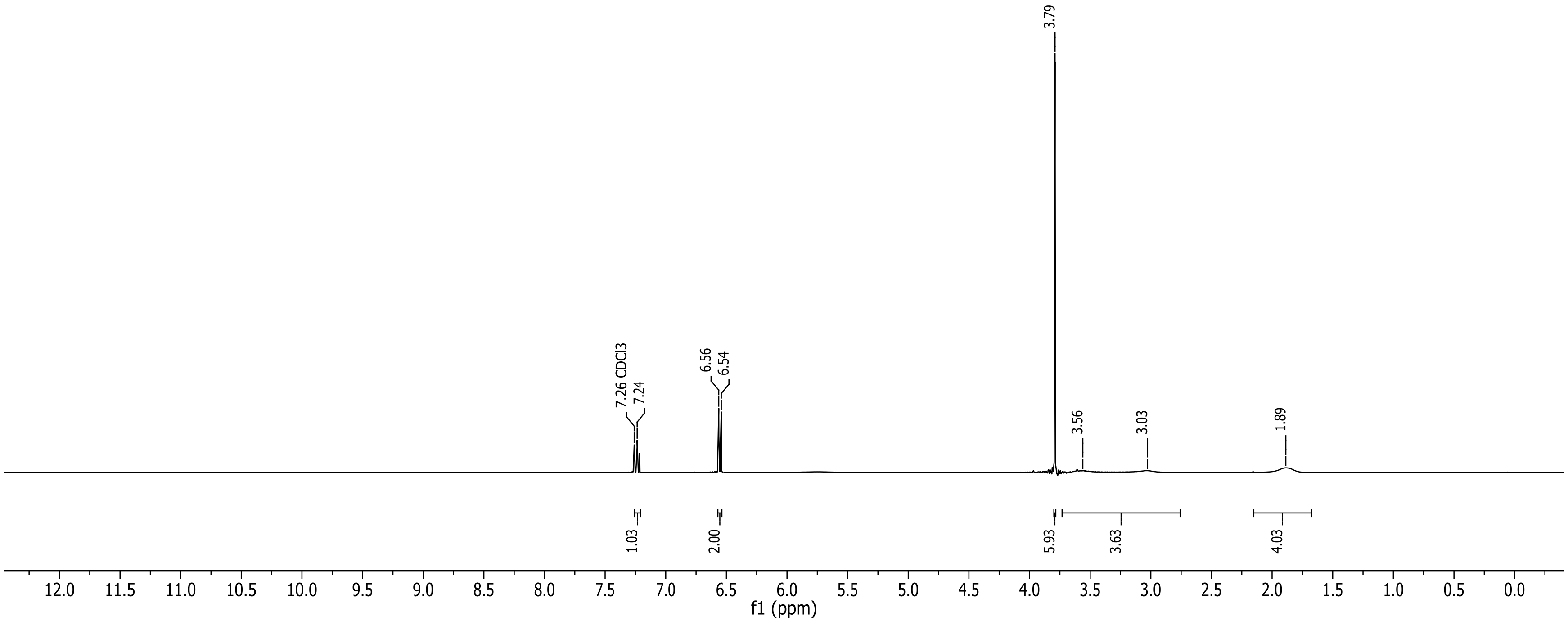

3t

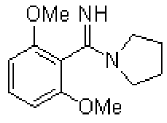

Origin	Varian
Spectrometer	mercury
Solvent	cdcl3
Temperature	25.0
Pulse Sequence	s2pul
Experiment	1D
Number of Scans	4096
Relaxation Delay	1.0000
Spectrometer Frequency	100.58
Nucleus	13C

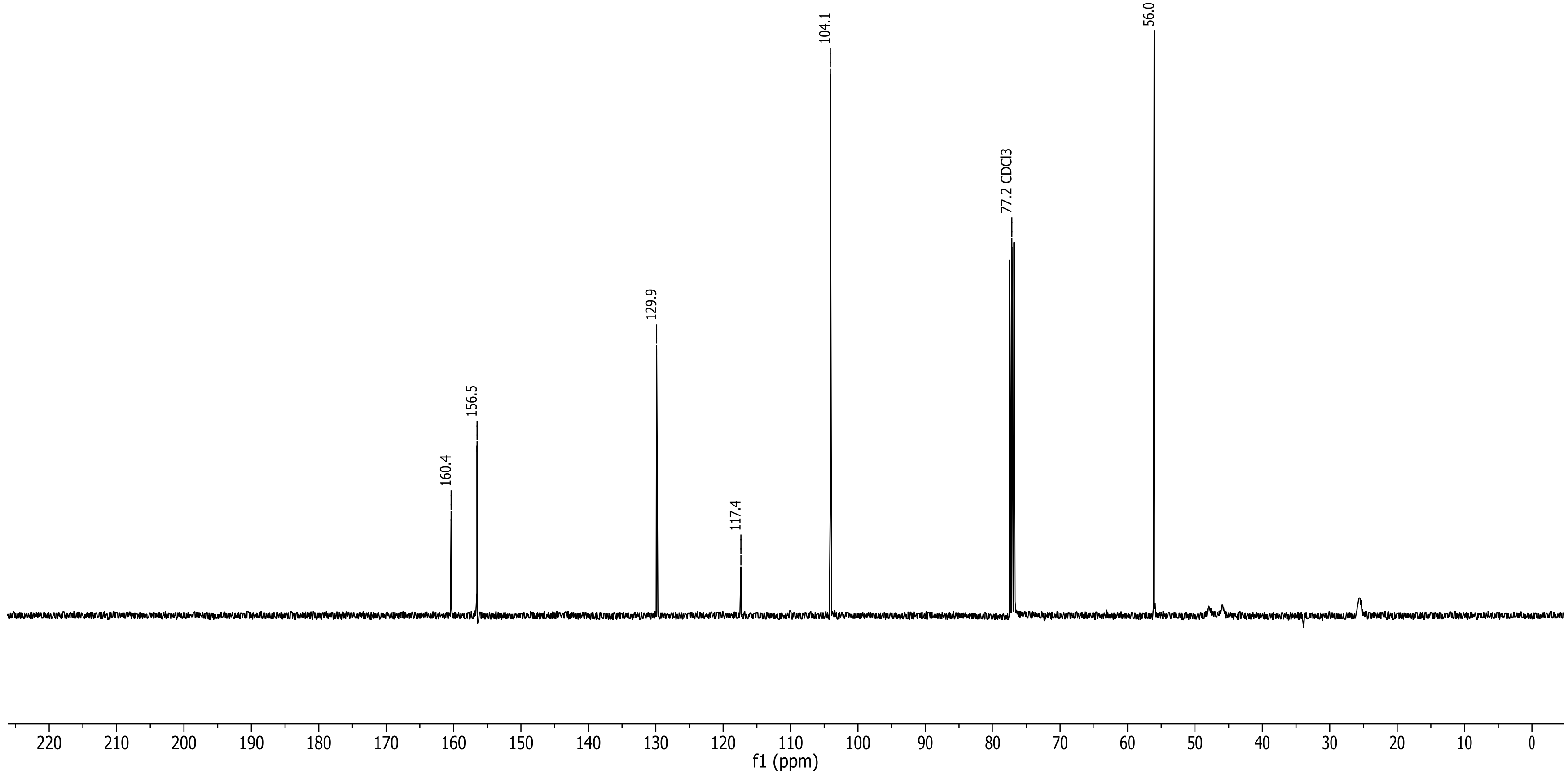

3t

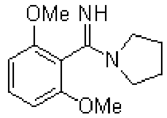

HCl added

Origin	Varian
Spectrometer	mercury
Solvent	cd3od
Temperature	25.0
Pulse Sequence	s2pul
Experiment	1D
Number of Scans	1024
Relaxation Delay	1.0000
Spectrometer Frequency	100.58
Nucleus	13C

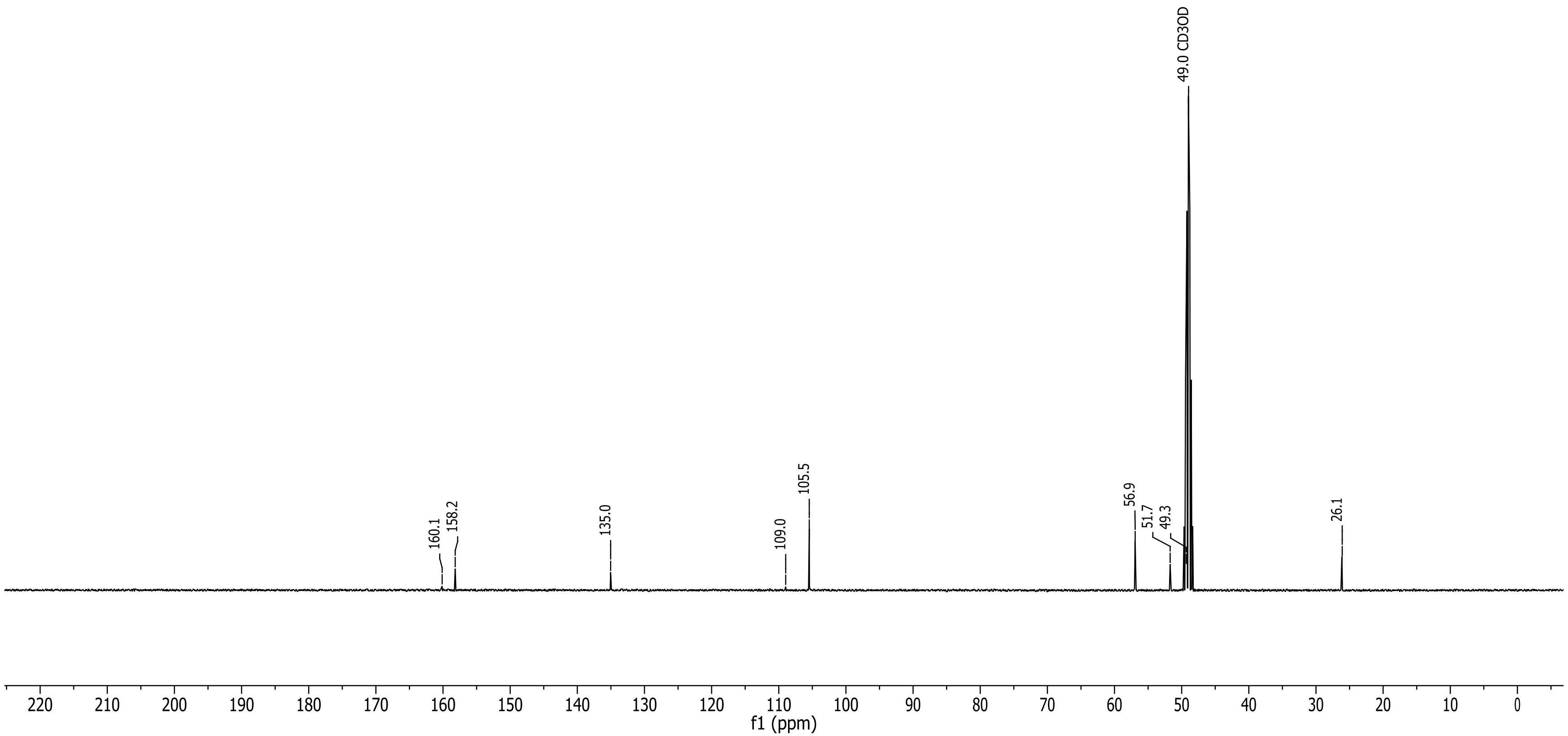

3u

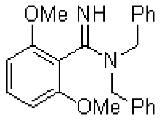

Origin	Varian
Spectrometer	mercury
Solvent	cdcl3
Temperature	25.0
Pulse Sequence	s2pul
Experiment	1D
Number of Scans	32
Relaxation Delay	27.0000
Spectrometer Frequency	399.86
Nucleus	1H

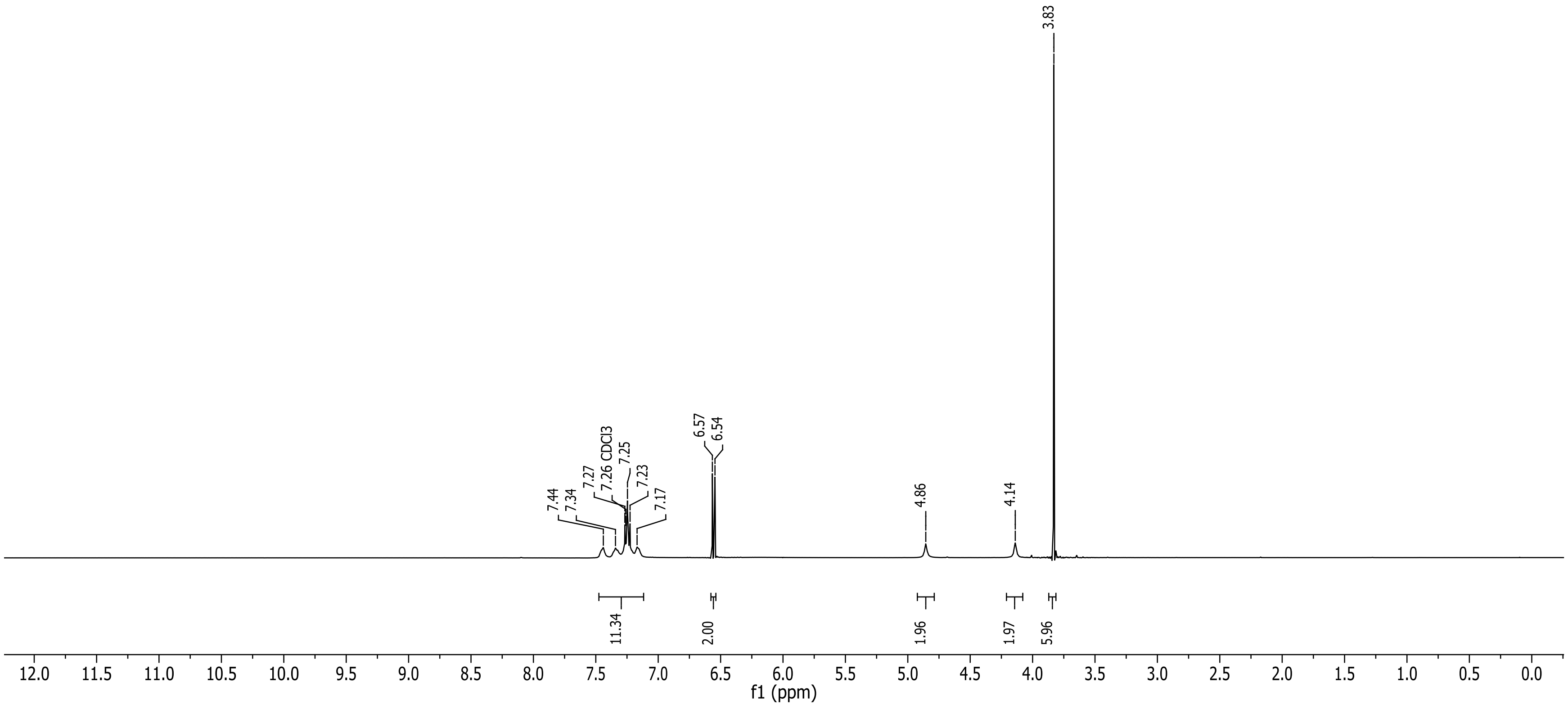

3u

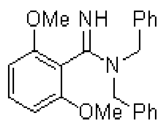

Origin	Varian
Spectrometer	mercury
Solvent	cdcl3
Temperature	25.0
Pulse Sequence	s2pul
Experiment	1D
Number of Scans	4096
Relaxation Delay	1.0000
Spectrometer Frequency	100.55
Nucleus	13C

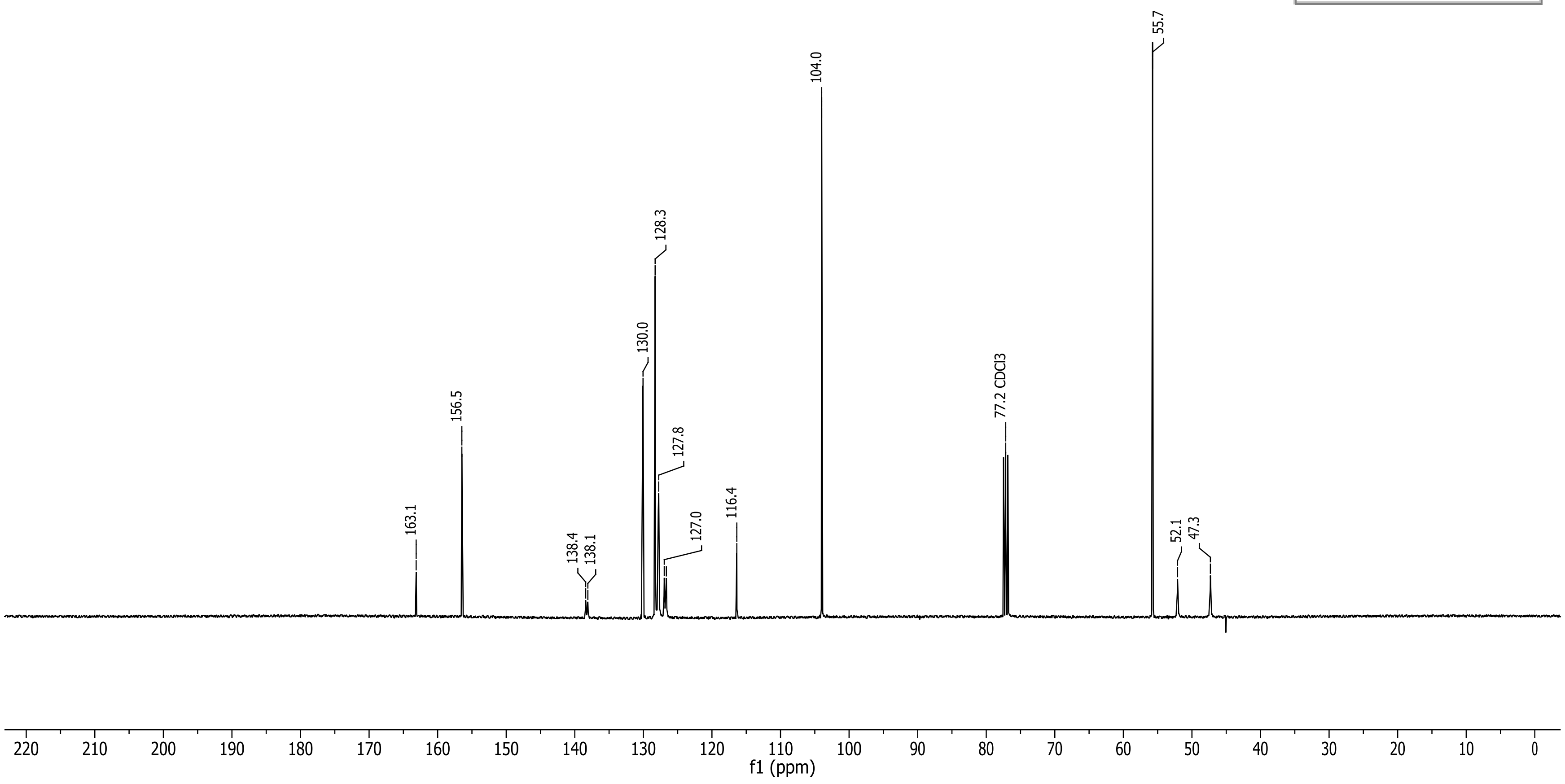

Continuous-Flow Scale-Out

Microwave assisted continuous-flow scale-out was performed on a WaveCraft Arrhenius One system, featuring a non-resonant microwave applicator. The pump used was a Uniqsis Binary Pump Module. The system was equipped with a 70 bar back pressure regulator and the temperature was determined by the built-in online IR-sensor. The system enables quick changes to the reaction temperature which allowed for rapid screening of different temperatures and residence times. Aliquots from different conditions were then analyzed by UHPLC-MS (Dionex Ultra3000 with a Bruker amaZon iontrap mass spectrometer using a Phenomenex Kinetex core-shell C18, 2.6 µm, 4.6 x 50 mm column) and a condition was chosen for scale-out and purification (140 °C, 1 ml/min corresponding to 43 s residence time). Purification was performed according to general procedure.

DFT Study: Energies of Reported Complexes

Energies for the complexes shown in Table 3 in $kJmol^{-1}$.

Complex	1a	1b	1c
**I**	30.1	4.1	48.9
**II**	0	0	0
**III**	56.1	54	76.2
**IV**	47.9	59.4	88.8
**TS-I**	110.3	111.1	138.5
**V**	95.2	86	98.3
**VI**	65.2	58	67.7
**VII**	-25.8	-32.7	-43.4
**VIII**	36.7	21.4	41.9
**TS-II**	85.1	85.2	105.7
**IX**	35.1	27.7	47.3
**X**	-11.7	-2.1	-17.6
**II** + product	36.5	-63.4	-97.7

Energies in a.u. Calculated at B3LYP, LACVP* level of theory.

Molecule	E_{solv}	E_{disp}	$\Delta G_{373.15}$	G_{tot}
Acetic acid	-229.09094	-0.00299	0.02653	-229.06740
Acetate	-228.61045	-0.00250	0.01243	-228.60052
CO_2	-188.58165	-0.00019	-0.01536	-188.59719
**2b**	-148.79901	-0.00073	0.00338	-148.79637
Benzoic acid	-420,82037	-0,00956	0,07311	-420,75682
**1b**	-649.86333	-0.01906	0.12935	-649.75304
**1c**	-764.37729	-0.01510	0.15682	-764.23556
**1a**	-764.38822	-0.01504	0.15702	-764.24624
imidine	-381,07033	-0,01116	0,09813	-380,98336
**3n**	-610.10757	-0.02120	0.15241	-609.97637
2,3,6-trimethoxy imidine	-724.62312	-0.02725	0.17896	-724.47141
**3b**	-724.63406	-0.02631	0.18159	-724.47878

**1a**	E_{solv}	E_{disp}	$\Delta G_{373.15K}$	G_{tot}
**I**	-1118.49806	-0.04248	0.21682	-1118.32372
**II**	-2189.07819	-0.09195	0.47728	-2188.69286
**III**	-1425.15126	-0.05075	0.30989	-1424.89213
**IV**	-1425.14458	-0.06057	0.30986	-1424.89528
**TS-I**	-1425.11766	-0.06041	0.30657	-1424.87150
**V**	-1425.12246	-0.05986	0.30508	-1424.87725
**VI**	-1236.54036	-0.05204	0.30091	-1236.29149
**VII**	-2000.48487	-0.09227	0.47166	-2000.10548
**VIII**	-1385.36627	-0.05935	0.32682	-1385.09880
**TS-II**	-1385.34463	-0.06346	0.32780	-1385.08028
**IX**	-1385.37343	-0.06208	0.33237	-1385.10314
**X**	-2149.29867	-0.10201	0.50421	-2148.89647

**1c**	E_{solv}	E_{disp}	$\Delta G_{373.15K}$	G_{tot}
**I**	-1118.49806	-0.04248	0.21682	-1118.32372
**II**	-2189,06681	-0,09538	0,48354	-2188,67866
**III**	-1425,13713	-0,05132	0,30748	-1424,88096
**IV**	-1425,12315	-0,06132	0,30832	-1424,87616
**TS-I**	-1425,10212	-0,06101	0,30590	-1424,85723
**V**	-1425,11324	-0,06154	0,30491	-1424,86987
**VI**	-1425,13713	-0,05132	0,30748	-1424,88096
**VII**	-2000,46961	-0,09739	0,46989	-2000,09711
**VIII**	-1385,35367	-0,06358	0,32653	-1385,09072
**TS-II**	-1385,32861	-0,06434	0,32652	-1385,06643
**IX**	-1385,35538	-0,06393	0,33065	-1385,08866
**X**	-2149,28226	-0,10683	0,50520	-2148,88389

**1b**	E_{solv}	E_{disp}	$\Delta G_{373.15K}$	G_{tot}
**I**	-1118.49806	-0.04248	0.21682	-1118.32372
**II**	-1539,26650	-0,06458	0,32015	-1539,01093
**III**	-1310,62595	-0,04599	0,28209	-1310,38985
**IV**	-1310,61402	-0,05541	0,28163	-1310,38779
**TS-I**	-1310,59210	-0,05526	0,27927	-1310,36809
**V**	-1310,59978	-0,05560	0,27772	-1310,37766
**VI**	-1122,01706	-0,04743	0,27337	-1121,79111
**VII**	-1771,44011	-0,08358	0,41188	-1771,11181
**VIII**	-1270,84307	-0,05460	0,29624	-1270,60143
**TS-II**	-1270,81911	-0,05842	0,30040	-1270,57714
**IX**	-1270,84630	-0,05720	0,30444	-1270,59906
**X**	-1920,25236	-0,09282	0,44863	-1919,89654

**Benzoic acid**	E_{solv}	E_{disp}	$\Delta G_{373.15K}$	G_{tot}
**I**	-1118.49806	-0.04248	0.21682	-1118.32372
**II**	-1501,965473	-0,05828418	0,310972	-1501,712785
**III**	-1081,588675	-0,03616995	0,227024	-1081,397821
**IV**	-1081,561568	-0,04315878	0,225458	-1081,379269
**TS-I**	-1081,534257	-0,04294922	0,223106	-1081,3541
**V**	-1081,552907	-0,04238194	0,21738	-1081,377909
**VI**	-892,963522	-0,03404461	0,212617	-892,7849496
**VII**	-1081,588675	-0,03616995	0,227024	-1081,397821
**VIII**	-1041,798508	-0,04222611	0,239593	-1041,601141
**TS-II**	-1041,767894	-0,04540648	0,244342	-1041,568958
**IX**	-1041,79696	-0,04443183	0,247955	-1041,593437
**X**	-1462,187949	-0,06441106	0,339142	-1461,913218

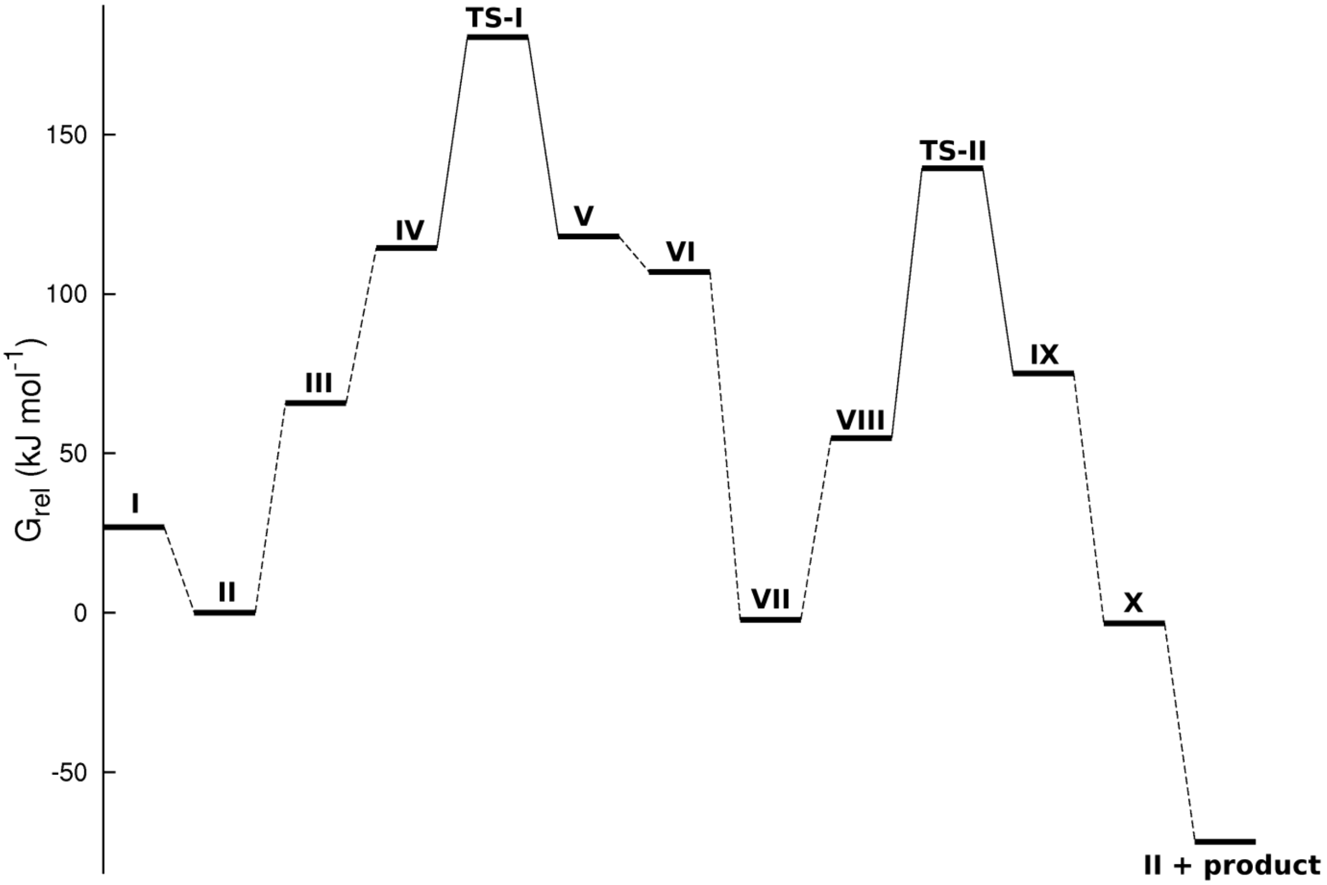

TS-I
TS-II
I
II
III
IV
V
VI
VII
VIII
IX
X
II + product
150
100
50
0
-50
G_{rel} (kJ mol^{-1})

DFT Study: Optimized Geometries of Reported Complexes

All coordinates in ångström.

acetic acid

atom x y z
O1 -2.172071 0.261352 1.386135
C2 -2.359242 -0.213709 0.288816
O3 -1.376543 -0.298326 -0.644924
C4 -3.659633 -0.776955 -0.22881
H5 -3.973028 -0.232686 -1.125918
H6 -3.526763 -1.825984 -0.513616
H7 -4.425392 -0.694792 0.542946
H8 -0.580818 0.084516 -0.229126

acetate

atom x y z
O2 -2.242471 0.257449 1.405392
C3 -2.275257 -0.209445 0.239539
O4 -1.361927 -0.29167 -0.620958
C23 -3.66533 -0.781653 -0.233141
H30 -4.008837 -0.243674 -1.129327
H31 -3.559156 -1.836466 -0.527166
H32 -4.434359 -0.70325 0.546224

CO2

atom x y z
O1 0.0 0.0 1.169708
C2 0.0 0.0 0.0
O3 0.0 0.0 -1.169708

2b

atom x y z
N2 -2.581936 0.69746 0.565153
H3 -2.827948 -0.203664 0.169937
H4 -3.339435 1.368973 0.501948
C4 -1.379476 1.169939 0.179112
N8 -0.322555 1.572936 -0.102877

benzoic acid

atom x y z
O1 -0.508656 -1.608919 0.094104
C2 0.503931 -2.260642 -0.004403
O3 1.698049 -1.634523 -0.195403
C4 0.457167 -6.548729 0.274535
C5 1.524898 -5.924944 -0.37302
C6 1.564599 -4.534446 -0.470044
C7 0.532585 -3.758019 0.076138
C8 -0.547238 -4.393134 0.704042
C9 -0.579967 -5.780778 0.810709
H10 0.429016 -7.632224 0.35406
H11 2.322413 -6.52023 -0.808705
H12 -1.414894 -6.265768 1.308944
H13 2.383998 -4.069075 -1.014254
H14 -1.348738 -3.778943 1.101572
H15 2.426283 -2.269215 -0.098469

1b

atom x y z
O1 2.696573 -2.141651 0.156951
C2 2.110619 -3.181310 -0.028143
O3 0.974892 -3.444664 0.662801
C4 3.580972 -6.143851 -2.821622
C5 2.829688 -6.568061 -1.729453
C6 2.320298 -5.609813 -0.849669
C7 2.563278 -4.227848 -1.022647
C8 3.314540 -3.834341 -2.162012
C9 3.820181 -4.794943 -3.052045
H10 3.981043 -6.882041 -3.511528
H11 2.645900 -7.623774 -1.576178
O12 1.554001 -5.957262 0.238186
O13 3.484920 -2.508625 -2.354541
H14 4.394601 -4.493286 -3.918538
C15 1.300730 -7.331757 0.507707
C16 4.230442 -2.058561 -3.474779
H17 0.698196 -7.347420 1.417090
H18 0.743979 -7.803372 -0.310899
H19 2.236442 -7.875787 0.679283
H20 4.235337 -0.969982 -3.401527
H21 5.263379 -2.428613 -3.447126
H22 3.761815 -2.358188 -4.421563
H23 0.724379 -4.378743 0.534167
H14 -1.432553 1.497675 -5.256104
H15 -5.520617 -0.207043 -1.224027
H16 -5.085469 0.826099 0.171572
H17 -5.268501 1.527087 -1.461617
C18 -4.398093 -3.275406 -2.866063
C19 -1.383600 3.239991 -3.429274
H20 -4.919908 -3.789595 -2.056805
H21 -5.063006 -3.199049 -3.736537
H22 -3.504499 -3.849510 -3.143623
H23 -1.283482 4.079808 -2.739090
H24 -0.384719 2.899718 -3.733117
H25 -1.939228 3.565108 -4.318899
H26 -1.677780 0.326885 -0.516442

1c

atom x y z
atom x y z
O1 -0.367129 -1.979351 0.568891
C2 0.637401 -2.645021 0.488733
O3 1.819894 -2.060294 0.19169
C4 0.582057 -6.954971 0.969251
C5 1.587099 -6.373924 0.203884
C6 1.618255 -4.969744 0.087882
C7 0.661289 -4.146237 0.70202
C8 -0.338124 -4.767181 1.504273
C9 -0.362665 -6.158235 1.621478
O10 2.669979 -4.399721 -0.611592
O11 -1.211607 -3.955109 2.146894
H12 -1.118545 -6.643209 2.226563
C13 2.56332 -4.457233 -2.045892
C14 -2.224042 -4.535433 2.950409
H15 3.462105 -3.974038 -2.433381
H16 2.52672 -5.497211 -2.378934
H17 1.674319 -3.914645 -2.387252
H18 -2.800414 -3.697001 3.345019
H19 -2.885868 -5.184597 2.361519
H20 -1.801607 -5.111352 3.785278
H21 0.526675 -8.031524 1.083596
O22 2.571496 -7.052958 -0.459288
C23 2.588378 -8.466549 -0.344532
H24 3.446095 -8.804062 -0.929167
H25 2.713512 -8.78386 0.699285
H26 1.672341 -8.916917 -0.750654
H27 2.487722 -2.759029 0.022928

1a

atom x y z
O1 -0.427747 -1.974142 0.308788
C2 0.595026 -2.61662 0.349968
O3 1.787831 -1.973392 0.282877
C4 0.56419 -6.946729 0.652172
C5 1.67293 -6.319322 0.079695
C6 1.691803 -4.922072 0.020152
C7 0.633677 -4.11806 0.494593
C8 -0.465762 -4.802764 1.090922
C9 -0.495343 -6.196155 1.165204
H10 2.498179 -6.898039 -0.307084
O11 2.76609 -4.254317 -0.52061
O12 -1.452845 -4.038796 1.603382

H13 -1.322385 -6.728111 1.615396
C14 3.866242 -4.983782 -1.04945
C15 -2.565375 -4.663789 2.22344
H16 4.565353 -4.233008 -1.421034
H17 4.354439 -5.588462 -0.275589
H18 3.548905 -5.627744 -1.877627
H19 -3.212951 -3.847567 2.547447
H20 -3.11194 -5.304183 1.519398
H21 -2.262118 -5.258405 3.095218
O22 0.427734 -8.295164 0.765896
C23 1.460934 -9.132379 0.270062
H24 1.133493 -10.155499 0.463298
H25 1.606092 -8.996392 -0.809576
H26 2.410538 -8.951962 0.790664
H27 2.505028 -2.626087 0.178244

imidine

atom x y z
N1 2.169314 -1.402846 0.93687
C2 1.316735 -2.253936 0.490968
N3 1.753485 -3.527557 0.144699
C4 -2.861201 -1.482957 -0.203237
C5 -2.167398 -2.360242 -1.038058
C6 -0.816102 -2.622261 -0.812206
C7 -0.138136 -2.000479 0.247021
C8 -0.844983 -1.121827 1.080965
C9 -2.196342 -0.865476 0.858601
H10 -3.914869 -1.28147 -0.377857
H11 -2.677452 -2.837375 -1.870854
H12 -2.733388 -0.188758 1.517997
H13 1.118056 -4.288204 0.352112
H14 2.707252 -3.701821 0.442278
H15 -0.334468 -0.655853 1.919846
H16 -0.270757 -3.289265 -1.473367
H17 1.739927 -0.480533 1.023303

3n

atom x y z
N1 -2.527767 0.65089 -0.236993
C2 -3.46292 0.456358 -1.088694
C3 -2.309179 -0.451023 -5.13357
C4 -3.029886 -1.392647 -4.40299
C5 -3.424103 -1.073692 -3.0981
C6 -3.096497 0.162948 -2.516543
C7 -2.383352 1.101226 -3.28766
C8 -1.986292 0.792062 -4.597312
H9 -1.997273 -0.690009 -6.146992
H10 -3.273858 -2.350902 -4.844967
O11 -4.14926 -1.923085 -2.300532
O12 -2.139168 2.299107 -2.695875
H13 -1.435238 1.508746 -5.193764
H14 -5.401804 -0.042427 -1.456122
H15 -5.104209 0.461505 0.118211
C16 -4.417539 -3.232515 -2.775547
C17 -1.381046 3.266201 -3.400025
H18 -4.952973 -3.73876 -1.969766
H19 -5.047392 -3.217568 -3.674987
H20 -3.490347 -3.777543 -2.993441
H21 -1.289988 4.119031 -2.724653
H22 -0.378726 2.892442 -3.647501
H23 -1.884652 3.587105 -4.322257
N24 -4.836412 0.547565 -0.856808
H25 -2.919825 0.847135 0.691596

2,3,6-trimethoxy imidine

N1 -0.528773 -1.78543 0.940844
C2 0.257288 -2.766388 0.688028
N3 -0.261117 -4.03993 0.522664
C4 4.539954 -2.403571 0.380158
C5 3.931519 -2.261774 1.623214
C6 2.525139 -2.394434 1.722534
C7 1.758225 -2.67592 0.588737
C8 2.401972 -2.851951 -0.656535
C9 3.781272 -2.702444 -0.759457
O10 1.905144 -2.339253 2.942135
O11 1.577014 -3.168907 -1.707137
H12 4.287533 -2.819368 -1.710384
C13 1.863202 -1.063367 3.583099
C14 2.145809 -3.250918 -3.002919
H15 1.345882 -1.222576 4.53156
H16 2.868473 -0.674985 3.770778
H17 1.292161 -0.344154 2.981548
H18 1.317313 -3.463297 -3.6816
H19 2.619911 -2.30416 -3.293904
H20 2.88637 -4.059493 -3.071271
H21 0.237051 -4.627006 -0.134036
H22 -1.265717 -4.035403 0.380852
H23 5.614227 -2.301676 0.277808
O24 4.592667 -2.010905 2.794904
C25 6.006205 -1.927447 2.75247
H26 6.32749 -1.752518 3.78108
H27 6.453761 -2.861298 2.386295
H28 6.34558 -1.094989 2.120581
H29 0.009998 -0.932651 1.092407

3b

atom x y z
N1 -0.511116 -1.748328 1.206598
C2 0.226135 -2.669043 0.701492
N3 -0.379643 -3.822052 0.214445
C4 4.555555 -2.5845 0.53755
C5 3.904451 -2.314911 1.742075
C6 2.501996 -2.350231 1.7796
C7 1.73097 -2.658255 0.641652
C8 2.435926 -2.925964 -0.553322
C9 3.827567 -2.887684 -0.61689
H10 4.467615 -2.081829 2.633667
O11 1.802642 -2.09004 2.92096
O12 1.662906 -3.219524 -1.646774
H13 4.373766 -3.087881 -1.528911
C14 2.505941 -1.822757 4.121684
C15 2.298337 -3.422295 -2.900201
H16 1.739398 -1.668823 4.883447
H17 3.14275 -2.667992 4.414253
H18 3.121259 -0.916645 4.039906
H19 1.493267 -3.6006 -3.615699
H20 2.86779 -2.53609 -3.206436
H21 2.966207 -4.293009 -2.878963
H22 0.068342 -4.224023 -0.599363
H23 -1.38024 -3.695572 0.101415
O24 5.910426 -2.575499 0.381575
C25 6.721006 -2.286689 1.508702
H26 7.753071 -2.341537 1.156867
H27 6.525352 -1.279381 1.89986
H28 6.572964 -3.021041 2.311626
H29 0.067149 -1.016568 1.617152

I

Pd1 0.525945 0.529766 -0.090139
O2 -2.064478 0.526254 1.340123
C3 -2.226866 0.033624 0.228604
O4 -1.319385 -0.019027 -0.724707
C5 4.507531 2.965691 1.468231
C6 3.378659 3.578512 0.934921
C7 2.336405 2.785383 0.456698
N8 2.389232 1.423417 0.510359
C9 3.494143 0.818345 1.001296
C10 4.566005 1.581249 1.4907
H11 5.329196 3.563771 1.851378

H12 3.312822 4.65868 0.895748
H13 5.436076 1.066567 1.883668
C14 -0.215889 5.191294 -1.004899
C15 0.944534 4.745962 -0.378106
C16 1.128337 3.376308 -0.162015
N17 0.180412 2.495057 -0.553926
C18 -0.934549 2.913407 -1.169345
C19 -1.170077 4.262104 -1.414734
H20 -0.367032 6.253214 -1.174959
H21 1.697433 5.460307 -0.068071
H22 -2.083196 4.568481 -1.913618
C23 -3.551598 -0.595189 -0.194339
C24 3.577139 -0.676128 1.000247
H25 -1.621368 2.124686 -1.454287
C26 1.180422 -2.191581 -0.501161
O27 0.733967 -1.413142 0.451926
O28 1.725575 -1.808765 -1.536804
C29 0.982689 -3.673332 -0.194811
H30 -3.824119 -0.297091 -1.211635
H31 -3.446852 -1.686176 -0.192369
H32 -4.337808 -0.310589 0.508204
H33 1.4953 -4.280451 -0.944009
H34 1.35706 -3.915123 0.805679
H35 -0.087528 -3.908117 -0.205004
H36 4.536752 -1.003225 1.409488
H37 2.759631 -1.110318 1.582137
H38 3.465176 -1.055824 -0.021303

2,3,6-trimethoxybenzoic acid

II

atom x y z
Pd1 1.584556 -0.645956 0.236647
C2 -3.170014 7.337259 -0.008868
C3 -4.552857 5.14063 0.306393
C4 -3.331746 2.787167 0.474023
N5 -0.785176 2.642844 0.311353
C6 0.600166 4.811383 0.103578
C7 -0.546649 7.173867 -0.083535
H8 -4.104192 9.15866 -0.162365
H9 -6.599139 5.190114 0.423966
C10 -4.912388 0.463159 0.827194
H11 0.58172 8.869957 -0.274679
C12 8.522262 3.646612 0.342966
C13 7.656384 6.121711 0.127375
C14 5.067542 6.569571 0.022371
C15 3.384687 4.535413 0.152412
N16 4.272767 2.14586 0.368559
C17 6.766807 1.689969 0.442109
H18 10.52479 3.216781 0.431092
H19 8.973642 7.693362 0.036615
H20 4.377065 8.487455 -0.155267
H21 -6.654196 0.96671 1.824028
H22 -5.44325 -0.326099 -1.017071
H23 -3.90771 -1.010815 1.852742
O24 -0.76395 -2.605619 -4.321762
C25 -1.646259 -3.764108 -2.504872
O26 -1.071193 -3.383967 -0.140339
C27 -7.397796 -9.395012 -3.871004
C28 -5.147402 -10.098388 -2.664928
C29 -3.285342 -8.301881 -2.160399
C30 -3.663073 -5.761981 -2.898169
C31 -5.915464 -5.084096 -4.084789
C32 -7.809494 -6.898183 -4.582386
H33 -4.876552 -12.059291 -2.140379
O34 -1.042755 -8.82017 -1.018634
O35 -6.388117 -2.586574 -4.654199
C36 -0.630862 -11.288105 -0.064508
C37 -5.892831 -1.90765 -7.230871
H38 1.134892 -11.192631 0.991724
H39 -2.159575 -11.862837 1.218919
H40 -0.481073 -12.682664 -1.598406
H41 -6.389973 0.090983 -7.400381
H42 -3.88697 -2.171564 -7.652187
H43 -7.076764 -3.017399 -8.5183
O44 4.076181 -3.391265 4.458654
C45 4.854149 -4.230294 2.435615
O46 4.246286 -3.426389 0.187793
C47 10.292962 -10.339725 2.659786
C48 7.846381 -10.714802 3.544801
C49 6.089967 -8.705439 3.4113
C50 6.773731 -6.368402 2.408184
C51 9.262823 -6.027667 1.514602
C52 11.000391 -7.997578 1.647322
O53 3.642126 -9.123807 4.15352
O54 9.849598 -3.665218 0.596014
H55 12.922811 -7.755043 0.984217
C56 3.183452 -8.840742 6.809541
C57 12.265171 -3.309404 -0.492722
H58 1.176893 -9.243013 7.081745
H59 4.314496 -10.192616 7.899836
H60 3.590945 -6.898625 7.386543
H61 12.287268 -1.377913 -1.22411
H62 13.783645 -3.524135 0.908208
H63 12.5976 -4.627117 -2.060629
H64 7.317133 -0.279955 0.545135
H65 -8.813469 -10.832141 -4.232648
H66 11.682151 -11.843356 2.736117
O67 6.952949 -12.917259 4.566459
O68 -9.943737 -6.009618 -5.757362
C69 8.666968 -14.955451 4.800825
H70 7.604351 -16.496856 5.669432
H71 9.381028 -15.575404 2.95168
H72 10.280719 -14.466751 6.014649
C73 -11.870303 -7.774849 -6.312573
H74 -13.374661 -6.706373 -7.238651
H75 -11.20388 -9.267926 -7.594753
H76 -12.623087 -8.65946 -4.590171

III

atom x y z
Pd1 1.454275 -0.513667 0.622447
C2 -2.863632 7.337807 -1.210729
C3 -4.253318 5.115853 -1.310603
C4 -3.08865 2.792085 -0.791478
N5 -0.608783 2.725236 -0.193353
C6 0.785552 4.891059 -0.081075
C7 -0.306319 7.229756 -0.586465
H8 -3.754083 9.142518 -1.612602
H9 -6.246435 5.1423 -1.789573
C10 -4.546847 0.36011 -0.889929
H11 0.808718 8.943804 -0.498086
C12 8.411099 3.408176 1.894231
C13 7.722562 5.919025 1.488127
C14 5.237472 6.50172 0.835922
C15 3.472971 4.556731 0.599327
N16 4.192473 2.131998 1.008114
C17 6.575903 1.543266 1.635225
H18 10.326938 2.888819 2.402488
H19 9.106887 7.420578 1.676249
H20 4.691275 8.449223 0.518513
H21 -6.524973 0.730317 -1.350657
H22 -3.763628 -0.920283 -2.314455
H23 -4.458809 -0.639829 0.918437
O24 -0.70373 -3.806194 0.39315
C25 1.242822 -5.102969 1.029889
O26 3.271365 -3.807566 1.423323

C27 1.148232 -13.086123 1.805305
C28 2.97194 -11.983248 0.259892
C29 2.983101 -9.325405 -0.022885
C30 1.198534 -7.863893 1.304727
C31 -0.616912 -9.020262 2.897481
C32 -0.628975 -11.639052 3.123479
O33 4.670543 -8.064128 -1.487541
O34 -2.200123 -7.431716 4.148616
H35 -1.983825 -12.588543 4.326895
C36 5.180085 -8.939905 -4.007729
C37 -4.032417 -8.502819 5.800369
H38 5.865417 -7.284146 -5.031224
H39 6.61249 -10.420553 -4.006219
H40 3.456082 -9.643586 -4.908841
H41 -5.075192 -6.908483 6.588026
H42 -5.338522 -9.741096 4.773027
H43 -3.132314 -9.56596 7.335837
H44 6.964752 -0.450265 1.921385
H45 1.108547 -15.120676 2.039035
O46 4.809493 -13.278724 -1.001421
C47 4.872853 -15.956337 -0.769472
H48 6.468022 -16.57903 -1.915617
H49 5.173823 -16.536364 1.197266
H50 3.129761 -16.822758 -1.485759

IV

atom x y z
Pd1 1.515901 -0.551235 0.532954
O2 -1.140351 -3.252907 0.6414
C3 0.298885 -5.241435 0.928768
O4 -0.30812 -7.425509 1.147175
C5 7.862457 6.036418 0.441474
C6 5.288907 6.559823 0.32365
C7 3.540561 4.577484 0.336322
N8 4.350354 2.146523 0.470385
C9 6.834319 1.636372 0.576057
C10 8.655868 3.529232 0.565476
H11 9.224585 7.570545 0.432184
H12 4.655645 8.502492 0.218367
H13 10.643004 3.035203 0.65281
C14 -2.919778 7.579353 -0.10111
C15 -0.303416 7.35701 0.059553
C16 0.774192 4.958154 0.190902
N17 -0.680411 2.833019 0.17524
C18 -3.224653 3.015185 0.005388
C19 -4.37506 5.402442 -0.134566
H20 -3.798605 9.430591 -0.203166
H21 0.864818 9.03633 0.081134
H22 -6.417298 5.506265 -0.267355
C23 8.326341 -5.218479 1.289114
C24 7.003784 -4.760095 3.529209
C25 4.358927 -4.416861 3.385745
C26 3.127682 -4.369901 0.95815
C27 4.59126 -4.88346 -1.29129
C28 7.177718 -5.28303 -1.101147
O29 2.807638 -4.081105 5.348728
O30 3.249628 -4.986067 -3.437172
H31 8.327719 -5.680594 -2.74673
C32 3.315613 -5.044898 7.853526
C33 4.50296 -5.607282 -5.752615
H34 1.482352 -5.006697 8.791968
H35 4.017817 -6.982521 7.752823
H36 4.66498 -3.842938 8.84135
H37 3.024259 -5.638159 -7.184869
H38 5.915924 -4.170735 -6.233386
H39 5.395281 -7.470205 -5.62634
H40 7.349239 -0.342326 0.669747
C41 -4.821279 0.674094 -0.036396
H42 -6.810881 1.181978 -0.248117
H43 -4.277637 -0.576744 -1.588585
H44 -4.589442 -0.424136 1.698473
O45 8.098253 -4.576208 5.853844
C46 10.704287 -5.209461 6.11189
H47 11.117274 -5.056423 8.124794
H48 11.07744 -7.14557 5.473093
H49 11.910154 -3.88485 5.066152
H50 10.351547 -5.524531 1.380154

TS-I

atom x y z
Pd1 1.409615 -0.491837 0.581626
O2 -0.960746 -3.613024 0.844175
C3 0.530473 -5.407485 1.113514
O4 0.693909 -7.613087 1.406406
C5 7.276043 6.476681 0.190911
C6 4.673695 6.824425 0.053765
C7 3.05313 4.740163 0.158896
N8 4.018151 2.371519 0.397704
C9 6.532037 2.032274 0.52942
C10 8.227488 4.035629 0.431816
H11 8.535976 8.09374 0.110785
H12 3.916108 8.71533 -0.132487
H13 10.240796 3.670311 0.546507
C14 -3.591802 7.314017 -0.371668
C15 -0.964335 7.269603 -0.247322
C16 0.264409 4.953356 0.019753
N17 -1.050718 2.752441 0.161403
C18 -3.59835 2.768324 0.039486
C19 -4.912254 5.053028 -0.229023
H20 -4.587267 9.096002 -0.579335
H21 0.09189 9.018147 -0.359847
H22 -6.959277 5.02204 -0.322684
C23 8.091244 -6.539626 1.382168
C24 7.067736 -5.509149 3.588212
C25 4.893406 -3.974232 3.373694
C26 3.795541 -3.525188 0.953921
C27 4.991179 -4.511307 -1.263184
C28 7.098671 -6.059499 -1.025621
O29 3.728277 -2.81754 5.327257
O30 3.886307 -3.880299 -3.481486
H31 8.008504 -6.898816 -2.656481
C32 3.572684 -3.969096 7.798004
C33 4.779367 -5.046463 -5.750197
H34 2.004032 -3.004109 8.72509
H35 3.161017 -5.987622 7.635712
H36 5.317224 -3.688942 8.856334
H37 3.554803 -4.348766 -7.252172
H38 6.738962 -4.502244 -6.144053
H39 4.633682 -7.108524 -5.629961
H40 7.170553 0.099624 0.720719
C41 -5.000134 0.304554 0.194901
H42 -7.034608 0.621824 0.0464
H43 -4.421579 -0.978515 -1.320476
H44 -4.614197 -0.660348 1.983105
O45 8.072606 -5.820681 5.939094
C46 10.188986 -7.460401 6.23243
H47 10.60797 -7.472797 8.250276
H48 9.74401 -9.386351 5.607964
H49 11.839784 -6.754866 5.194647
H50 9.754035 -7.729781 1.513457

V

atom x y z
C1 7.769569 6.272687 -0.092272
C2 5.174671 6.662806 -0.234035
C3 3.51004 4.613585 -0.10269
N4 4.431769 2.228274 0.162225

C5 6.942678 1.84661 0.310764
C6 8.671972 3.819539 0.19172
H7 9.057017 7.867079 -0.18963
H8 4.453287 8.56682 -0.423066
H9 10.676877 3.416073 0.325849
C10 -3.015468 7.478243 -0.584003
C11 -0.393842 7.2813 -0.618031
C12 0.725267 4.93193 -0.179998
N13 -0.689028 2.831889 0.236367
C14 -3.231243 3.013484 0.307776
C15 -4.442442 5.3333 -0.094723
H16 -3.923009 9.286808 -0.92391
H17 0.741313 8.940873 -0.992497
H18 -6.489149 5.422361 -0.02705
H19 7.543384 -0.092789 0.527855
C20 -4.759226 0.675804 0.827987
H21 -6.746041 1.157818 1.116131
H22 -4.643698 -0.65095 -0.756083
H23 -4.070678 -0.29702 2.517368
Pd24 1.759919 -0.594908 0.306493
C25 7.86395 -7.264803 0.187353
C26 6.481734 -6.801793 2.382807
C27 4.699704 -4.799948 2.366691
C28 4.332541 -3.366957 0.194693
C29 5.69678 -3.885122 -2.030152
C30 7.472026 -5.834879 -2.007422
O32 3.120129 -4.331065 4.391929
O33 5.187903 -2.369855 -4.06914
H34 8.560036 -6.287865 -3.683728
C35 4.280523 -3.573155 6.726699
C36 6.471925 -2.881734 -6.376709
H37 2.727631 -3.209834 8.035314
H38 5.494632 -5.065377 7.470155
H39 5.375071 -1.832439 6.457102
H40 5.761926 -1.502797 -7.735475
H41 8.523664 -2.652117 -6.172592
H42 6.062241 -4.795445 -7.060822
O43 -1.124669 -3.743221 0.410492
C44 -1.006277 -5.859236 1.113806
O45 -1.015546 -7.949603 1.752719
H45 9.239572 -8.780567 0.133923
O46 6.671894 -8.147388 4.56809
C47 8.405916 -10.203625 4.64646
H48 8.233813 -11.009187 6.535922
H49 7.925895 -11.644256 3.235349
H50 10.354185 -9.560531 4.343796

VI

atom x y z
Pd1 0.642254 0.105469 -0.632391
C2 -2.187685 8.610601 -2.248228
C3 -3.624071 6.608077 -3.160686
C4 -2.73648 4.151454 -2.844404
N5 -0.520368 3.658589 -1.694958
C6 0.92569 5.597202 -0.80912
C7 0.104972 8.092614 -1.067227
H8 -2.832374 10.54882 -2.441354
H9 -5.421112 6.910711 -4.098686
C10 7.851398 3.376374 2.456051
C11 7.445434 5.940295 2.084682
C12 5.170892 6.733914 1.02357
C13 3.35553 4.926677 0.398995
N14 3.756246 2.424401 0.810757
C15 5.970242 1.632971 1.791821
H16 9.617806 2.69261 3.241617
H17 8.889177 7.31102 2.581612
C18 -3.997852 -7.128221 -2.904006
C19 -4.806779 -5.057589 -4.336313
C20 -3.691472 -2.633018 -3.982724
C21 -1.85186 -2.34474 -2.131974
C22 -1.106378 -4.464548 -0.767293
C23 -2.090708 -6.855441 -1.085803
H24 -4.834985 -8.973073 -3.19489
O26 -4.532778 -0.618105 -5.389317
O27 0.908253 -3.811751 0.909096
H28 -1.432849 -8.470705 -0.005291
C29 -3.862428 -0.646929 -8.023208
C30 0.360548 -4.243554 3.557028
H31 -4.607615 1.105365 -8.818586
H32 -1.802264 -0.696632 -8.246436
H33 -4.720549 -2.262004 -8.97878
H34 1.940851 -3.467396 4.625241
H35 -1.405435 -3.310369 4.089286
H36 0.224936 -6.277141 3.892887
C37 6.408742 -1.148139 2.186579
H38 5.974675 -1.674668 4.145543
H39 5.24813 -2.303618 0.933181
H40 8.396548 -1.614973 1.86183
H48 1.235518 9.628192 -0.325059
H49 4.85184 8.721436 0.658549
H50 -3.771017 2.530294 -3.545704
O43 -6.64426 -5.160026 -6.124026
C44 -7.88931 -7.507468 -6.564612
H45 -9.289981 -7.12792 -8.028163
H46 -6.561885 -8.951912 -7.234677
H47 -8.844718 -8.17994 -4.85276

VII

atom x y z
Pd1 1.85981 0.004386 -1.732645
C2 -2.816642 6.728187 2.604851
C3 -0.50259 5.794231 3.427067
C4 0.757742 3.999602 1.96026
N5 -0.251182 3.110104 -0.238533
C6 -2.428684 4.112173 -1.121165
C7 -3.754385 5.915788 0.296897
H8 -3.835851 8.115219 3.723494
H9 0.329389 6.487458 5.162635
H10 -5.509731 6.666846 -0.450501
C11 6.95162 2.842469 5.38664
C12 4.485187 3.656372 4.960017
C13 3.326734 3.099731 2.648771
N14 4.548431 1.743677 0.862322
C15 6.917885 0.95523 1.272167
C16 8.203588 1.495021 3.50654
H17 7.871771 3.251002 7.175917
H18 3.481779 4.67629 6.423672
H19 10.122711 0.820392 3.761934
C20 -3.416428 3.340518 -3.667854
H21 7.780747 -0.161827 -0.213288
H22 -4.665781 4.814937 -4.406087
H23 -1.8738 3.019964 -5.001132
H24 -4.491537 1.578993 -3.542367
C25 -4.798497 -4.915442 -5.562184
C26 -4.600858 -4.677516 -2.939375
C27 -2.685947 -3.217255 -1.868493
C28 -0.922595 -1.962698 -3.406369
C29 -1.136262 -2.181069 -6.024118
C30 -3.083734 -3.650478 -7.119893
H31 -5.960185 -5.612218 -1.714163
O32 -2.624988 -2.890342 0.73327
O33 0.426256 -0.772958 -7.567937
C34 -1.703416 -5.023257 2.121826
C35 2.299224 -2.188233 -8.914042
H36 -1.882239 -4.529505 4.121451
H37 -2.851378 -6.712942 1.741479

H38	0.281856	-5.393972	1.674091
H39	3.375366	-0.807336	-10.013239
H40	3.551927	-3.153558	-7.581485
H41	1.418821	-3.548203	-10.203143
O42	4.475236	-4.459861	0.812815
C43	5.126802	-4.313073	-1.415428
O44	4.334548	-2.691705	-3.080435
C45	10.872303	-9.478861	-4.055411
C46	11.441708	-6.924302	-3.820603
C47	9.528487	-5.242157	-3.013779
C48	7.11829	-6.115042	-2.429604
C49	6.575383	-8.716169	-2.667279
C50	8.445071	-10.375949	-3.486093
O51	10.101726	-2.717048	-2.686609
O52	4.15458	-9.4097	-2.093329
H53	8.058911	-12.376731	-3.695558
C54	10.1858	-1.28905	-4.986942
C55	3.486643	-11.984596	-2.339836
H56	10.679687	0.643442	-4.446295
H57	11.637517	-2.03528	-6.258336
H58	8.330603	-1.300576	-5.903213
H59	1.497268	-12.106899	-1.806094
H60	3.712941	-12.654867	-4.293186
H61	4.609624	-13.193798	-1.078038
H62	12.296595	-10.815919	-4.673283
O63	13.752535	-5.861823	-4.317047
C64	15.728701	-7.49982	-5.066222
H65	17.39148	-6.305595	-5.329311
H66	16.133448	-8.925611	-3.611361
H67	15.293246	-8.468416	-6.851657
O68	-3.162523	-3.683016	-9.713517
C69	-5.098377	-5.103413	-10.885106
H70	-4.825484	-4.872194	-12.91864
H71	-4.976728	-7.1211	-10.405027
H72	-6.985679	-4.406486	-10.367332
H73	-6.309659	-6.044947	-6.361536

VIII

atom	x	y	z
Pd1	1.687788	-0.579441	0.632762
N2	-0.852712	-3.532059	0.17709
C3	-1.002292	-5.670312	-0.32121
N4	-1.184713	-8.028679	-1.11597
C5	7.756746	6.30832	0.735066
C6	5.163917	6.724812	0.649497
C7	3.487027	4.679162	0.649435
N8	4.383087	2.274471	0.725071
C9	6.89178	1.873909	0.797633
C10	8.640801	3.83299	0.808465
H11	9.057036	7.895605	0.729682
H12	4.457032	8.641334	0.555099
H13	10.644304	3.403907	0.865136
C14	-3.010897	7.624397	0.490537
C15	-0.400314	7.40517	0.697734
C16	0.707678	5.017017	0.516851
N17	-0.703987	2.899053	0.188118
C18	-3.236215	3.099434	-0.042747
C19	-4.433617	5.458262	0.09555
H20	-3.911633	9.462626	0.630392
H21	0.73718	9.075087	1.014654
H22	-6.471675	5.559355	-0.096648
C23	7.820314	-7.0213	2.610299
C24	7.114103	-5.20903	4.406679
C25	5.314061	-3.377199	3.815278
C26	4.232306	-3.316362	1.376467
C27	4.920121	-5.142383	-0.387692
C28	6.725492	-7.028689	0.218755
H29	7.980565	-5.274706	6.262375
O30	4.515372	-1.53825	5.462416
O31	3.716802	-5.245455	-2.712556
C32	5.528511	-1.535077	7.951185
C33	5.217222	-4.483914	-4.839369
H34	4.625655	0.041786	8.926758
H35	5.093887	-3.299313	8.950748
H36	7.583872	-1.247546	7.937734
H37	3.994828	-4.640907	-6.495023
H38	5.826173	-2.513687	-4.623948
H39	6.86148	-5.714458	-5.065889
H40	7.480978	-0.082022	0.852083
C41	-4.778193	0.750246	-0.453621
H42	-6.752551	1.237984	-0.81362
H43	-4.056996	-0.335973	-2.056725
H44	-4.699222	-0.482833	1.205358
H45	0.19364	-8.508409	-2.372241
H46	-1.627054	-9.372029	0.178173
O47	7.216399	-8.75475	-1.637931
C48	9.091864	-10.620482	-1.154112
H49	9.213594	-11.757103	-2.87025
H50	10.936977	-9.755719	-0.766766
H51	8.558324	-11.835415	0.439998
H52	9.210304	-8.433103	3.129145

TS-II

atom	x	y	z
Pd1	1.588438	-0.504854	0.860755
N2	-1.108054	-3.324255	1.071565
C3	0.22823	-5.131204	1.450782
N4	0.436833	-7.600346	2.034683
C5	8.131924	5.9273	0.154597
C6	5.579611	6.48663	-0.128671
C7	3.768728	4.571839	0.113731
N8	4.498765	2.167306	0.618188
C9	6.962592	1.62963	0.898782
C10	8.844013	3.449706	0.684075
H11	9.537828	7.41013	-0.032214
H12	5.012682	8.410292	-0.531423
H13	10.809753	2.922717	0.929154
C14	-2.536396	7.778583	-0.847525
C15	0.063778	7.43803	-0.668668
C16	1.013256	5.035549	-0.135673
N17	-0.543013	3.017751	0.199963
C18	-3.072406	3.333418	0.049913
C19	-4.109119	5.715384	-0.477135
H20	-3.313057	9.632046	-1.262405
H21	1.3172	9.031154	-0.942212
H22	-6.145945	5.913374	-0.589001
C23	8.079758	-6.70051	2.189046
C24	6.918484	-5.575626	4.284217
C25	4.790892	-4.062206	3.934115
C26	3.841025	-3.644993	1.453411
C27	5.064071	-4.759912	-0.649854
C28	7.184667	-6.353703	-0.272364
H29	7.692616	-5.918072	6.148989
O30	3.510214	-2.899046	5.82464
O31	3.973246	-4.330846	-2.930786
C32	4.206982	-3.426979	8.375372
C33	5.454058	-4.045879	-5.191497
H34	2.868311	-2.384693	9.544383
H35	4.061025	-5.452412	8.786782
H36	6.13109	-2.770195	8.778602
H37	4.222092	-3.045454	-6.509046
H38	7.147271	-2.913701	-4.826206
H39	6.000393	-5.873025	-5.971361
H40	7.409915	-0.325235	1.307316
C41	-4.78024	1.105148	0.461653
H42	-6.75792	1.690439	0.354475

H43 -4.437988 -0.365099 -0.949563
H44 -4.435242 0.24005 2.306762
H45 -1.168238 -8.625904 1.793497
H46 2.046594 -8.469679 1.47431
O47 8.19167 -7.486008 -2.35938
C48 10.387864 -9.012327 -2.062046
H49 10.873012 -9.667663 -3.955168
H50 11.969771 -7.920613 -1.283039
H51 10.012408 -10.64906 -0.845524
H52 9.710131 -7.898401 2.512138

IX

atom x y z
Pd1 1.623728 -0.766063 0.31795
C2 7.593171 6.311887 -0.691581
C3 8.512538 3.8663 -1.020182
C4 6.834867 1.858659 -0.767762
N5 4.38226 2.187178 -0.215211
C6 3.45934 4.551829 0.098073
C7 5.046183 6.656239 -0.138959
H8 8.83257 7.935846 -0.883808
H9 10.480005 3.512846 -1.475005
C10 -4.43346 5.092887 1.111138
C11 -2.986107 7.218106 1.610094
C12 -0.376556 7.071393 1.313057
C13 0.707028 4.777946 0.597337
N14 -0.72026 2.666343 0.248439
C15 -3.266354 2.81462 0.412943
H16 -6.477363 5.154574 1.25245
H17 -3.868468 8.97844 2.187339
C18 -4.854655 0.553049 -0.214883
H19 -6.845023 1.093326 -0.32487
H20 -4.272285 -0.261572 -2.023929
H21 -4.617434 -0.947356 1.183622
N22 -0.992448 -3.403418 0.89241
C23 0.436219 -5.329302 1.128108
N24 -0.296179 -7.758529 1.653516
C25 8.529247 -4.613396 0.820366
C26 7.321759 -4.137761 3.110247
C27 4.640852 -4.237607 3.195593
C28 3.252625 -4.735074 0.925601
C29 4.585094 -5.294858 -1.373918
C30 7.208634 -5.189177 -1.408689
O31 3.267826 -4.125217 5.320148
O32 3.117234 -5.978908 -3.342876
H33 8.268166 -5.604223 -3.110036
C34 3.845852 -2.403322 7.348374
C35 4.284886 -6.438482 -5.734264
H36 2.106827 -2.291809 8.44892
H37 5.39242 -3.109903 8.508972
H38 4.319271 -0.536242 6.593041
H39 2.74481 -6.894683 -7.023473
H40 5.280027 -4.750161 -6.406508
H41 5.60095 -8.034569 -5.627593
H42 4.302852 8.551508 0.064321
H43 0.787693 8.719223 1.653465
H44 7.445311 -0.08219 -1.011421
H45 0.858463 -9.162732 1.062036
H46 -2.166407 -8.119443 1.44946
H47 10.577442 -4.595725 0.744837
O48 8.529022 -3.66978 5.333304
C49 11.220173 -3.729299 5.377539
H50 11.753781 -3.357766 7.333043
H51 11.941858 -5.586724 4.806724
H52 12.02877 -2.261872 4.154512

X

atom x y z
Pd1 1.34354 -0.845649 -0.777099
C2 -2.857217 6.31613 2.976483
C3 -4.108047 3.999027 2.923635
C4 -2.981015 1.988821 1.659083
N5 -0.761834 2.2365 0.459124
C6 0.483789 4.475055 0.475671
C7 -0.542862 6.552639 1.751313
H8 -3.660348 7.921728 3.972369
H9 -5.911122 3.731606 3.863076
C10 7.164963 4.562398 -3.882709
C11 6.457578 6.788093 -2.693612
C12 4.274075 6.811431 -1.226353
C13 2.907204 4.573606 -0.939353
N14 3.685051 2.381363 -2.019153
C15 5.730912 2.358089 -3.531796
H16 8.819083 4.491507 -5.094061
H17 7.560991 8.502815 -2.934843
C18 6.460104 -0.034743 -4.872574
H19 7.918221 -1.05352 -3.808828
H20 4.849315 -1.303138 -5.078497
H21 7.234711 0.420377 -6.738758
N22 -1.301985 -3.212179 0.463208
C23 -2.493928 -4.526967 -1.16786
C25 -0.548448 -4.670509 -9.060868
C26 0.384348 -6.526706 -7.427251
C27 -0.360125 -6.540051 -4.903055
C28 -1.948559 -4.61809 -3.967463
C29 -2.9142 -2.789311 -5.617256
C30 -2.240287 -2.842365 -8.205289
H31 0.017542 -4.713975 -11.029337
H32 1.658635 -7.957523 -8.153375
O33 0.31696 -8.361012 -3.205518
O34 -4.581877 -1.069502 -4.637174
H36 -4.669135 -7.679182 -1.389013
H37 -4.541751 -6.323997 1.47536
C39 2.673433 -9.595997 -3.575492
C40 -4.244168 1.54853 -5.20299
H41 3.020257 -10.673111 -1.852599
H42 2.614168 -10.897754 -5.193748
H43 4.184785 -8.206115 -3.820161
H44 -5.237546 2.574761 -3.709642
H45 -2.238114 2.065047 -5.161205
H46 -5.042398 2.039672 -7.042206
O47 6.188022 -1.582394 1.63819
C48 5.777705 -3.496411 0.358836
O49 3.915811 -3.847216 -1.187855
C50 11.179603 -9.561018 1.313653
C51 11.914975 -7.341435 0.113072
C52 10.114198 -5.399208 -0.238858
C53 7.626254 -5.689251 0.568084
C54 6.916961 -7.953092 1.79027
C55 8.687349 -9.86572 2.158005
H56 12.519292 -11.080904 1.620344
O58 10.843402 -3.212344 -1.453787
O59 4.461812 -8.075212 2.588197
H60 8.171556 -11.606891 3.107042
C61 12.094816 -1.431284 0.163454
C62 3.753264 -10.084181 4.199574
H63 12.557355 0.194827 -1.025243
H64 10.823098 -0.852244 1.687369
H65 13.844221 -2.238039 0.924511
H66 1.798345 -9.708982 4.740782
H67 3.8503 -11.924433 3.238042
H68 4.942188 -10.154995 5.901552
H69 0.460294 8.3364 1.808003
H70 3.648114 8.550914 -0.347035
H71 -3.768802 0.093908 1.609376
O71 -3.352871 -1.055195 -9.730257

C72 -2.639097 -0.990232 -12.304547
H73 -3.696151 0.568607 -13.149918
H74 -0.604053 -0.639718 -12.531193
H75 -3.129434 -2.754971 -13.283106
O75 14.303363 -6.840863 -0.759651
C76 16.171171 -8.716127 -0.38965
H77 17.912801 -7.95425 -1.194498
H78 16.468967 -9.121258 1.626359
H79 15.687609 -10.486077 -1.363464
N78 -4.560168 -6.025286 -0.422153

2,4,6-trimethoxybenzoic acid

II

atom x y z
Pd1 1.348289 -0.652638 0.810071
C2 -2.318955 7.157806 -2.300713
C3 -4.004881 5.318162 -1.514347
C4 -3.125293 3.11407 -0.323899
N5 -0.629623 2.770921 0.054349
C6 1.033926 4.638157 -0.572585
C7 0.2421 6.833505 -1.788031
H8 -2.978386 8.847308 -3.262353
H9 -6.022597 5.538173 -1.809296
C10 -5.007872 1.184422 0.56404
H11 1.591771 8.273491 -2.33003
C12 8.414108 3.342207 2.230593
C13 7.952689 5.631626 1.021837
C14 5.558656 6.09914 0.036502
C15 3.6853 4.248307 0.239831
N16 4.197535 1.996471 1.350272
C17 6.482904 1.557537 2.358444
H18 10.234411 2.919733 3.073539
H19 9.42157 7.057815 0.870451
H20 5.156851 7.896342 -0.856566
H21 -6.615716 2.158483 1.435317
H22 -5.72001 0.095913 -1.048486
H23 -4.180781 -0.145152 1.896949
O24 -0.831404 -2.184191 -4.046374
C25 -1.869822 -3.392994 -2.355903
O26 -1.494152 -3.095984 0.069294
C27 -7.353769 -9.138663 -4.311267
C28 -5.269898 -9.785214 -2.821656
C29 -3.53364 -7.914865 -2.146054
C30 -3.832979 -5.389215 -2.953755
C31 -5.950621 -4.813059 -4.431
C32 -7.726814 -6.656963 -5.12605
H34 -5.050389 -11.736592 -2.248646
O35 -1.438363 -8.381667 -0.748598
O36 -6.235448 -2.321232 -5.082891
H37 -9.3599 -6.155265 -6.24384
C38 -1.026577 -10.835151 0.232026
C39 -8.08882 -1.667583 -6.894817
H40 0.668247 -10.677888 1.396893
H41 -2.618285 -11.461127 1.409025
H42 -0.714224 -12.224359 -1.280051
H43 -7.813415 0.340646 -7.285951
H44 -7.847702 -2.742072 -8.653135
H45 -10.017637 -1.960659 -6.180058
O46 4.565053 -3.205707 5.018716
C47 4.763357 -4.311293 2.975944
O48 3.607612 -3.745853 0.889366
C49 10.275647 -10.321966 2.379076
C50 8.098425 -10.694217 3.83031
C51 6.297127 -8.771366 3.963523
C52 6.627661 -6.470908 2.675249
C53 8.827691 -6.162954 1.241121
C54 10.667769 -8.065965 1.069186
H56 7.868478 -12.482822 4.796055
O57 4.092553 -8.993581 5.282514
O58 9.109676 -3.856806 0.099048
H59 12.362173 -7.77429 -0.032059
C60 3.806849 -11.061943 6.958125
C61 11.121435 -3.50922 -1.626763
H62 2.021398 -10.743856 7.941825
H63 3.716449 -12.872159 5.945256
H64 5.353216 -11.13585 8.339513
H65 10.863514 -1.621825 -2.42144
H66 12.97423 -3.60535 -0.690816
H67 11.062493 -4.905835 -3.159862
H68 6.700495 -0.235605 3.315101
O68 -8.948811 -11.093259 -4.875922
O69 11.935966 -12.303797 2.363138
C70 -11.080241 -10.591943 -6.411862
H71 -12.055555 -12.397013 -6.637752
H72 -12.368218 -9.229277 -5.519268
H73 -10.528301 -9.875227 -8.28074
C73 14.182589 -12.067509 0.929998
H74 15.205405 -13.842884 1.179104
H75 13.781038 -11.788225 -1.087706
H76 15.363853 -10.499649 1.607322

III

atom x y z
Pd1 1.506495 -0.457129 0.552919
C2 -2.946909 7.542103 0.298422
C3 -4.367565 5.346523 0.505094
C4 -3.167506 2.985342 0.595398
N5 -0.621452 2.854592 0.481122
C6 0.803238 4.993777 0.281316
C7 -0.321569 7.369282 0.185524
H8 -3.865202 9.375358 0.226117
H9 -6.413262 5.423862 0.598397
C10 -4.660524 0.582994 0.814377
H11 0.820631 9.060687 0.024969
C12 8.641942 3.339554 0.035162
C13 7.926691 5.873335 -0.076591
C14 5.371764 6.510371 -0.00353
C15 3.565395 4.596097 0.1799
N16 4.311002 2.148112 0.282833
C17 6.762098 1.509755 0.215829
H18 10.61226 2.77854 -0.015178
H19 9.344076 7.349687 -0.218469
H20 4.804219 8.475573 -0.086916
H21 -6.677835 1.003469 0.939494
H22 -4.337218 -0.645332 -0.818737
H23 -4.095996 -0.498381 2.484342
O24 -0.723979 -3.646402 0.795877
C25 1.269015 -5.062654 0.789785
O26 3.372783 -3.791894 0.650979
C27 1.069472 -13.113327 1.222407
C28 3.168292 -11.928299 0.133405
C29 3.232246 -9.294482 -0.002744
C30 1.194791 -7.796384 0.928526
C31 -0.921391 -9.086807 2.014533
C32 -0.968048 -11.706009 2.162467
H34 4.715519 -13.034339 -0.602787
O35 5.177834 -8.041074 -1.074253
O36 -2.796439 -7.624814 2.932364
H37 -2.52978 -12.724305 2.998483
C38 7.229586 -9.427443 -2.128731
C39 -4.90824 -8.801747 4.120969
H40 8.520025 -8.008727 -2.884334
H41 8.205779 -10.5457 -0.68297
H42 6.594018 -10.66706 -3.662716
H43 -6.138756 -7.256599 4.709776
H44 -5.924412 -10.030929 2.799019

H45 -4.323758 -9.89446 5.781169
H46 7.165471 -0.498944 0.31116
O46 0.829361 -15.638015 1.458653
C47 2.807224 -17.246589 0.582735
H48 2.180861 -19.16646 0.99221
H49 3.096594 -17.035246 -1.457988
H50 4.579504 -16.868706 1.587292

IV

atom x y z
Pd1 1.46193 -0.548648 0.584987
O2 -1.180293 -3.280052 0.520858
C3 0.284479 -5.25075 0.800741
O4 -0.281503 -7.453864 0.920196
C5 7.854452 6.00574 0.782222
C6 5.303104 6.540172 0.443428
C7 3.536677 4.57316 0.406794
N8 4.309462 2.143055 0.693272
C9 6.771877 1.625219 1.026477
C10 8.609167 3.501547 1.084465
H11 9.2284 7.529083 0.81426
H12 4.700236 8.481635 0.215289
H13 10.576327 2.998963 1.364654
C14 -2.855301 7.618701 -0.512824
C15 -0.249909 7.376295 -0.238174
C16 0.786321 4.976002 0.082745
N17 -0.694057 2.8721 0.125627
C18 -3.227769 3.076219 -0.132625
C19 -4.341801 5.462592 -0.455583
H20 -3.702648 9.47033 -0.76502
H21 0.939315 9.040724 -0.274749
H22 -6.377329 5.584454 -0.659187
C23 8.298995 -5.377327 1.423277
C24 6.861151 -4.979443 3.636018
C25 4.298963 -4.510914 3.422538
C26 3.083931 -4.288928 0.966413
C27 4.649019 -4.803011 -1.226515
C28 7.223334 -5.276472 -1.011381
H29 7.81617 -5.134771 5.437925
O30 2.729717 -4.241685 5.379704
O31 3.396497 -4.808406 -3.415336
H32 8.367344 -5.628304 -2.665187
C33 3.668227 -4.538266 7.903477
C34 4.683584 -5.418453 -5.71745
H35 2.027215 -4.30904 9.125049
H36 4.481805 -6.422419 8.162343
H37 5.077108 -3.083912 8.336088
H38 3.235468 -5.368353 -7.179495
H39 6.148729 -4.01518 -6.132396
H40 5.509527 -7.312342 -5.612524
H41 7.252865 -0.349686 1.261099
C42 -4.844714 0.7489 -0.073391
H43 -6.833061 1.261364 -0.287845
H44 -4.322771 -0.56472 -1.58093
H45 -4.604382 -0.285029 1.699775
O46 10.745538 -5.84045 1.84112
C47 12.414932 -6.374333 -0.225794
H48 14.255846 -6.707141 0.6342
H49 11.804924 -8.066725 -1.246596
H50 12.53112 -4.761371 -1.518302

TS-I

atom x y z
Pd1 1.38578 -0.484807 0.549379
O2 -0.982837 -3.700923 0.457412
C3 0.388564 -5.548495 0.713944
O4 0.710728 -7.733644 0.889795
C5 7.126303 6.589416 0.864514
C6 4.534929 6.897863 0.547325
C7 2.950099 4.784571 0.463045
N8 3.942311 2.424609 0.691528
C9 6.447821 2.121883 0.999185
C10 8.104357 4.157802 1.095198
H11 8.357416 8.229253 0.931418
H12 3.755812 8.781196 0.370267
H13 10.110619 3.823458 1.347841
C14 -3.676804 7.305496 -0.441572
C15 -1.062704 7.28175 -0.147814
C16 0.172315 4.969454 0.13538
N17 -1.124345 2.754416 0.12533
C18 -3.659392 2.752229 -0.153795
C19 -4.980422 5.029822 -0.441877
H20 -4.675768 9.083509 -0.666144
H21 -0.023043 9.043687 -0.144464
H22 -7.017366 4.983943 -0.66418
C23 8.066215 -6.585822 1.458217
C24 6.862231 -5.646509 3.636555
C25 4.808743 -4.037617 3.365198
C26 3.876839 -3.370521 0.925567
C27 5.201762 -4.281794 -1.223178
C28 7.264338 -5.910748 -0.980959
H29 7.580786 -6.238808 5.45793
O30 3.522415 -3.020351 5.332847
O31 4.294903 -3.484426 -3.482898
H32 8.22276 -6.646014 -2.627296
C33 4.193384 -3.754744 7.845559
C34 5.3634 -4.474422 -5.756556
H35 2.875122 -2.766034 9.08237
H36 3.991561 -5.79969 8.100483
H37 6.132278 -3.181828 8.296697
H38 4.285256 -3.622191 -7.291582
H39 7.35842 -3.94538 -5.93686
H40 5.17332 -6.535293 -5.83394
H41 7.10521 0.194243 1.170744
C42 -5.038314 0.270008 -0.151297
H43 -7.0688 0.570093 -0.374292
H44 -4.383127 -0.945785 -1.691371
H45 -4.717212 -0.757011 1.615202
O46 10.025405 -8.13895 1.920374
C47 11.383185 -9.232935 -0.139282
H48 12.834432 -10.414216 0.722673
H49 10.142028 -10.404978 -1.31198
H50 12.281232 -7.772198 -1.302316

V

atom x y z
C1 7.851899 6.118187 0.717962
C2 5.280562 6.609839 0.472522
C3 3.55109 4.615807 0.316911
N4 4.386495 2.1853 0.407716
C5 6.873083 1.704943 0.647286
C6 8.665216 3.619377 0.807608
H7 9.189598 7.668376 0.839518
H8 4.630177 8.547401 0.410018
H9 10.646857 3.134266 1.000425
C10 -2.859273 7.711455 -0.303499
C11 -0.251054 7.448488 -0.095227
C12 0.788762 5.027473 0.066309
N13 -0.690495 2.931668 0.010337
C14 -3.222187 3.169084 -0.181582
C15 -4.35514 5.556921 -0.340017
H16 -3.704035 9.576516 -0.431608
H17 0.933787 9.114905 -0.064101
H18 -6.394168 5.693665 -0.49227
H19 7.400067 -0.267438 0.706416
C20 -4.812382 0.816382 -0.222572
H21 -6.818379 1.295696 -0.308076

H22 -4.356705 -0.355741 -1.865288
H23 -4.488026 -0.325798 1.470795
Pd24 1.639258 -0.557598 0.17431
C25 7.666744 -7.332389 0.557679
C26 6.642586 -6.363834 2.804106
C27 4.873668 -4.415073 2.673596
C28 4.120022 -3.42222 0.324759
C29 5.164792 -4.41533 -1.893783
C30 6.946554 -6.379099 -1.803462
H31 7.253806 -7.18565 4.575048
O32 3.767379 -3.341028 4.745664
O33 4.334227 -3.351735 -4.09991
H34 7.734802 -7.151119 -3.521934
C35 4.432264 -4.275802 7.184493
C36 5.310335 -4.273298 -6.434017
H37 3.334584 -3.16342 8.5283
H38 3.942776 -6.27952 7.381425
H39 6.453379 -4.020026 7.56365
H40 4.39103 -3.158322 -7.903757
H41 7.36246 -4.007669 -6.552988
H42 4.855793 -6.277173 -6.705202
O43 -1.235276 -3.701201 -0.093592
C44 -0.687624 -5.870721 -0.158062
O45 -0.322372 -8.026512 -0.237316
O46 9.362788 -9.222958 0.879805
C47 10.48517 -10.356733 -1.283581
H48 11.750425 -11.812935 -0.558323
H49 9.056418 -11.233488 -2.503411
H50 11.581245 -8.977811 -2.377591

VI

atom x y z
Pd1 0.594878 0.17633 -0.502414
C2 -1.864814 8.719485 -2.516613
C3 -3.393325 6.738476 -3.320757
C4 -2.620818 4.265951 -2.866161
N5 -0.432298 3.731298 -1.684562
C6 1.09465 5.646721 -0.891801
C7 0.394237 8.160333 -1.292437
H8 -2.415658 10.671822 -2.823489
H9 -5.172027 7.07497 -4.2818
C10 7.836192 3.298825 2.653153
C11 7.583991 5.845588 2.077446
C12 5.375717 6.679679 0.914969
C13 3.471913 4.931679 0.390822
N14 3.725894 2.448739 0.98789
C15 5.873254 1.616458 2.07944
H16 9.545311 2.582184 3.529995
H17 9.095167 7.16792 2.498543
C18 -3.76949 -7.10284 -3.1615
C19 -4.526459 -4.998881 -4.59968
C20 -3.563812 -2.587772 -4.120149
C21 -1.831566 -2.257295 -2.129642
C22 -1.143764 -4.394065 -0.802468
C23 -2.011011 -6.838626 -1.196558
H25 -5.86588 -5.342133 -6.10815
O26 -4.215352 -0.518089 -5.513217
O27 0.736031 -3.758603 1.026599
H28 -1.333011 -8.421052 -0.092727
C29 -5.909684 -0.824459 -7.590304
C30 -0.015082 -4.228924 3.617497
H31 -6.110573 1.048677 -8.429118
H32 -5.130897 -2.128019 -8.997738
H33 -7.762324 -1.502839 -6.958393
H34 1.480007 -3.472306 4.81372
H35 -1.813684 -3.295261 4.024809
H36 -0.181994 -6.266139 3.913528
C37 6.130181 -1.144643 2.701592
H38 5.38605 -1.524402 4.60027
H39 5.110757 -2.335821 1.36028
H40 8.119492 -1.703881 2.702376
H48 1.591301 9.678943 -0.623453
H49 5.173586 8.647729 0.3941
H50 -3.724626 2.663102 -3.483527
O43 -4.842658 -9.319793 -3.853271
C44 -4.16445 -11.558234 -2.524241
H45 -5.255364 -13.075879 -3.39279
H46 -2.140438 -11.964937 -2.719805
H47 -4.656994 -11.418047 -0.514614

VII

atom x y z
Pd1 1.642935 -0.353184 -1.043955
C2 -2.956036 7.47765 0.777019
C3 -0.340374 7.25638 0.880463
C4 0.771148 4.879263 0.608176
N5 -0.651158 2.776327 0.158202
C6 -3.199817 2.95334 0.205281
C7 -4.389775 5.303101 0.497695
H8 -3.854013 9.3135 0.971918
H9 0.81471 8.915562 1.195367
H10 -6.438801 5.382044 0.502854
C11 7.728167 5.909933 2.036473
C12 5.203306 6.46841 1.551815
C13 3.534413 4.502214 0.962538
N14 4.359919 2.091836 0.814151
C15 6.77888 1.536793 1.322249
C16 8.526475 3.404914 1.953064
H17 9.042578 7.419613 2.493677
H18 4.560761 8.409618 1.644576
H19 10.468089 2.887193 2.362165
C20 -4.781667 0.608327 0.002674
H21 7.269644 -0.450106 1.243659
H22 -6.662076 0.964556 0.789057
H23 -4.99228 0.024788 -1.969544
H24 -3.901256 -0.966536 1.005109
C25 -3.679748 -5.51375 -6.328147
C26 -3.354356 -6.152476 -3.782905
C27 -1.913767 -4.556906 -2.243647
C28 -0.807758 -2.336824 -3.168715
C29 -1.169355 -1.759375 -5.713989
C30 -2.611494 -3.316736 -7.323333
H32 -4.251083 -7.854369 -3.080732
O33 -1.563575 -4.996462 0.289287
O34 -0.053638 0.435807 -6.557633
H35 -2.872095 -2.8309 -9.290653
C36 -2.043718 -7.449446 1.246671
C37 -0.04513 0.940069 -9.179974
H38 -1.407476 -7.421312 3.209243
H39 -4.065092 -7.928112 1.190849
H40 -0.968339 -8.880534 0.20152
H41 1.077769 2.655048 -9.426874
H42 0.827581 -0.605262 -10.257379
H43 -1.959066 1.278396 -9.918294
O44 5.280511 -4.117065 1.464201
C45 5.392643 -4.277244 -0.857518
O46 4.182346 -2.90637 -2.491294
C47 10.462399 -9.689357 -4.308474
C48 11.449348 -7.543552 -3.122211
C49 9.791221 -5.811191 -2.024084
C50 7.161537 -6.169532 -2.091112
C51 6.236599 -8.325081 -3.30802
C52 7.861429 -10.108978 -4.41708
H54 13.479486 -7.295913 -3.105464
O55 10.594175 -3.637517 -0.86592
O56 3.671296 -8.592777 -3.305647

H57 7.103643 -11.77696 -5.318744
C58 13.220148 -3.140625 -0.79277
C59 2.568089 -10.520627 -4.796047
H60 13.431597 -1.334116 0.18515
H61 14.250948 -4.605111 0.256196
H62 14.025906 -2.980344 -2.699228
H63 0.533147 -10.195825 -4.709878
H64 3.191774 -10.405739 -6.772212
H65 3.00309 -12.41331 -4.056
O65 12.228442 -11.287646 -5.32111
O66 -5.114702 -7.181817 -7.708389
C67 -5.536705 -6.643757 -10.290122
H68 -6.707651 -8.183856 -11.012167
H69 -6.543228 -4.844853 -10.549576
H70 -3.760988 -6.576479 -11.366047
C70 11.371797 -13.498661 -6.558
H71 13.066953 -14.476568 -7.214853
H72 10.322797 -14.74273 -5.268264
H73 10.167416 -13.049358 -8.188665

VIII

atom x y z
Pd1 1.597852 -0.656215 0.944818
N2 -1.017638 -3.532029 1.15974
C3 -2.101056 -5.440756 1.213374
N4 -3.471272 -7.532282 1.347741
C5 7.889542 6.015792 0.38359
C6 5.33402 6.465157 -0.034465
C7 3.588614 4.486748 0.14795
N8 4.378384 2.11563 0.735625
C9 6.849184 1.685173 1.147763
C10 8.663432 3.578436 0.99108
H11 9.242923 7.552081 0.247046
H12 4.708438 8.360953 -0.477366
H13 10.6311 3.127793 1.34684
C14 -2.737433 7.490215 -1.239691
C15 -0.136098 7.19898 -1.014071
C16 0.836208 4.875255 -0.223566
N17 -0.695608 2.880442 0.280607
C18 -3.223412 3.161665 0.104655
C19 -4.29315 5.463002 -0.649745
H20 -3.530502 9.28018 -1.854031
H21 1.101281 8.765119 -1.45967
H22 -6.332221 5.630866 -0.773123
C23 7.985981 -7.028056 1.970204
C24 6.782354 -6.028618 4.108053
C25 4.868161 -4.23683 3.789466
C26 4.162124 -3.420606 1.365652
C27 5.364169 -4.462972 -0.740211
C28 7.297007 -6.26764 -0.465946
H30 7.391023 -6.679025 5.950179
O31 3.604528 -3.137552 5.761423
O32 4.584408 -3.58012 -3.047977
H33 8.231706 -7.050854 -2.104545
C34 4.339289 -3.774369 8.268829
C35 5.817314 -4.478949 -5.259295
H36 3.133788 -2.651356 9.509349
H37 4.044072 -5.78911 8.66104
H38 6.325983 -3.296724 8.621275
H39 4.921571 -3.501341 -6.839013
H40 7.842998 -4.032473 -5.237882
H41 5.571873 -6.525134 -5.49354
H42 7.349011 -0.242762 1.605528
C43 -4.89437 0.953493 0.735446
H44 -6.871156 1.54373 0.827188
H45 -4.724389 -0.524463 -0.703093
H46 -4.36145 0.120789 2.549777
H47 -4.076814 -8.299862 -0.300701
H48 -3.015272 -8.791082 2.719904
O48 9.832734 -8.746204 2.471805
C49 11.210146 -9.803068 0.424597
H50 12.587016 -11.081887 1.273641
H51 9.97573 -10.875905 -0.851605
H52 12.208647 -8.339414 -0.654775

TS-II

atom x y z
Pd1 1.619072 -0.488893 0.747075
N2 -1.045848 -3.363206 0.761703
C3 0.189636 -5.167173 1.379784
N4 0.394229 -7.610483 2.027264
C5 8.026115 6.094539 1.153309
C6 5.48273 6.636635 0.773985
C7 3.718977 4.670148 0.614539
N8 4.484095 2.234093 0.846439
C9 6.942495 1.709939 1.201308
C10 8.778194 3.581458 1.363517
H11 9.395563 7.617446 1.278194
H12 4.885566 8.585797 0.605546
H13 10.741058 3.065907 1.651887
C14 -2.556295 7.842341 -0.629358
C15 0.014228 7.539006 -0.172196
C16 0.983291 5.108737 0.168545
N17 -0.52897 3.033643 0.074066
C18 -3.025115 3.307947 -0.386254
C19 -4.079734 5.712901 -0.743596
H20 -3.347049 9.716424 -0.900604
H21 1.231075 9.18135 -0.095667
H22 -6.089578 5.877284 -1.110035
C23 7.971195 -6.950331 1.876912
C24 6.918888 -5.79236 4.03298
C25 4.92882 -4.096938 3.735556
C26 3.902921 -3.565441 1.307783
C27 5.024268 -4.751966 -0.815585
C28 7.042552 -6.450108 -0.552981
H30 7.741847 -6.23244 5.854016
O31 3.808563 -2.865583 5.68457
O32 4.013671 -4.125918 -3.080272
H33 7.881148 -7.337259 -2.189896
C34 4.670018 -3.335497 8.19759
C35 4.865678 -5.381857 -5.309607
H36 3.491549 -2.163255 9.415581
H37 4.436058 -5.328671 8.713788
H38 6.656638 -2.791728 8.422118
H39 3.713195 -4.627444 -6.841746
H40 6.862098 -4.971622 -5.68344
H41 4.58296 -7.429426 -5.172679
H42 7.418044 -0.273627 1.358789
C43 -4.673654 1.002054 -0.521479
H44 -6.61445 1.535452 -0.984111
H45 -3.981976 -0.32131 -1.949829
H46 -4.670783 -0.018291 1.275858
H47 2.134483 -8.226259 2.530103
H48 -1.066582 -8.343555 3.032479
O48 9.909637 -8.533306 2.361708
C49 11.143951 -9.783382 0.31555
H50 12.624015 -10.928058 1.178237
H51 9.830913 -11.011325 -0.714502
H52 11.99665 -8.418112 -0.989221

IX

atom x y z
Pd1 1.539433 -0.686177 0.357565
C2 7.605465 6.301721 -0.690153
C3 8.514702 3.832421 -0.816742
C4 6.799686 1.858973 -0.541021
N5 4.323892 2.237937 -0.152231

C6 3.410897 4.624853 -0.031649
C7 5.03451 6.698905 -0.304376
H8 8.871741 7.901255 -0.909873
H9 10.499211 3.432742 -1.138067
C10 -4.521143 5.260786 0.551322
C11 -3.087225 7.42079 0.928842
C12 -0.4669 7.245469 0.77197
C13 0.637881 4.897327 0.304371
N14 -0.781937 2.766497 0.062429
C15 -3.331988 2.931141 0.105396
H16 -6.568839 5.335989 0.595011
H17 -3.986173 9.225337 1.308082
C18 -4.894283 0.618776 -0.386636
H19 -6.890316 1.126271 -0.534256
H20 -4.298188 -0.294911 -2.143622
H21 -4.642078 -0.795053 1.096166
N22 -1.04921 -3.362926 0.912218
C23 0.443744 -5.222865 1.219406
N24 -0.201073 -7.669013 1.833563
C25 8.570876 -4.868105 1.071881
C26 7.306639 -4.363088 3.350768
C27 4.671609 -4.244941 3.344621
C28 3.243938 -4.518832 1.041173
C29 4.636883 -5.147565 -1.218809
C30 7.256255 -5.248317 -1.214816
H32 8.353223 -4.114276 5.085193
O33 3.28205 -3.930305 5.434163
O34 3.197058 -5.685545 -3.23849
H35 8.346733 -5.692947 -2.886536
C36 4.491476 -3.677541 7.836466
C37 4.384561 -6.281623 -5.594181
H38 2.960133 -3.486078 9.199298
H39 5.614049 -5.359129 8.281256
H40 5.687307 -1.987351 7.889017
H41 2.841409 -6.623966 -6.913824
H42 5.534921 -4.694486 -6.260217
H43 5.554134 -7.981538 -5.426105
H44 4.303849 8.609902 -0.256264
H45 0.686738 8.915624 1.026718
H46 7.399077 -0.098188 -0.647184
H47 0.96551 -9.040996 1.186841
H48 -2.063897 -8.077912 1.635365
O48 11.093592 -5.03856 0.873443
C49 12.64072 -4.83634 3.082245
H50 14.574666 -5.08932 2.421168
H51 12.445045 -2.971589 3.961287
H52 12.17446 -6.317581 4.450725

X

atom x y z
Pd1 1.346445 -0.877022 -0.652384
C2 -4.261361 5.660996 2.395925
C3 -5.134907 3.179527 2.256376
C4 -3.556489 1.342743 1.234685
N5 -1.245932 1.902091 0.350344
C6 -0.365201 4.303239 0.452922
C7 -1.857691 6.226791 1.49422
H8 -5.428289 7.141052 3.210159
H9 -6.993349 2.655753 2.947693
C10 6.805221 5.366371 -2.864929
C11 5.576574 7.475762 -1.914516
C12 3.219336 7.169056 -0.791542
C13 2.192494 4.744615 -0.612825
N14 3.450118 2.679919 -1.471728
C15 5.700499 2.966224 -2.628469
H16 8.626641 5.534386 -3.793053
H17 6.417688 9.342383 -2.069209
C18 7.04101 0.695387 -3.661764
H19 8.269948 -0.110834 -2.207397
H20 5.719392 -0.790312 -4.201122
H21 8.203129 1.234095 -5.287813
N22 -1.101629 -3.599923 0.205776
C23 -2.043752 -4.870278 -1.614019
C25 -0.021798 -3.512205 -9.386649
C26 1.083004 -5.523378 -8.081542
C27 0.326728 -6.013037 -5.601573
C28 -1.471229 -4.486516 -4.37711
C29 -2.579013 -2.515114 -5.756011
C30 -1.877743 -2.005464 -8.259944
H32 2.486073 -6.666874 -9.036797
O33 1.206751 -8.002344 -4.228729
O34 -4.353487 -1.120226 -4.492597
H35 -2.748582 -0.473958 -9.291708
H36 -3.709444 -8.249826 -2.300084
H37 -3.830144 -7.279223 0.713761
C39 3.696627 -8.886859 -4.736827
C40 -5.451159 0.981614 -5.725042
H41 4.145384 -10.202444 -3.213175
H42 3.796764 -9.890288 -6.552272
H43 5.041353 -7.317025 -4.698413
H44 -6.738933 1.831241 -4.353591
H45 -4.021364 2.382648 -6.276671
H46 -6.528734 0.413878 -7.406753
O47 6.278521 -1.64715 1.871723
C48 5.818319 -3.529069 0.57254
O49 4.034805 -3.743154 -1.095002
C50 10.745245 -9.941655 1.871936
C51 11.068763 -8.497027 -0.31651
C52 9.427657 -6.479642 -0.775381
C53 7.469343 -5.863801 0.902552
C54 7.196999 -7.356551 3.071731
C55 8.810088 -9.401971 3.580398
H57 12.598298 -8.987305 -1.58295
O58 9.622582 -4.960665 -2.868275
O59 5.261217 -6.687144 4.634833
H60 8.55521 -10.526256 5.265848
C61 11.692147 -5.33858 -4.521907
C62 4.925637 -8.008775 6.930671
H63 11.522085 -3.890506 -5.982805
H64 13.506959 -5.116031 -3.539249
H65 11.634717 -7.21269 -5.41251
H66 3.325034 -7.106141 7.86874
H67 4.484651 -10.013834 6.611957
H68 6.601146 -7.869445 8.150272
H69 -1.149583 8.14287 1.619849
H70 2.198921 8.799393 -0.091542
H71 -4.054377 -0.644227 1.128153
O71 0.822273 -3.173534 -11.808128
O72 12.442414 -11.873251 2.174264
C73 12.277142 -13.369835 4.384688
H74 13.807851 -14.748927 4.254529
H75 12.527943 -12.231774 6.10329
H76 10.463663 -14.374922 4.501371
C76 -0.190394 -1.15478 -13.239731
H77 0.758659 -1.224295 -15.07159
H78 -2.23672 -1.362702 -13.52556
H79 0.18257 0.682977 -12.347219
N78 -3.877629 -6.73828 -1.1293

2,6-dimethoxybenzoic acid

II

atom x y z
Pd1 0.869877 -0.237656 -0.211173
C2 -1.525524 4.054331 0.022124
C3 -2.280684 2.919464 -0.224959
C4 -1.660387 1.665821 -0.339982

N5 -0.317249 1.5506 -0.227461
C6 0.434153 2.663499 0.015685
C7 -0.14704 3.924331 0.146005
H8 -1.997651 5.027512 0.120616
H9 -3.359129 2.978039 -0.323763
C10 -2.497106 0.444217 -0.559269
H11 0.464167 4.794714 0.347227
C12 4.594298 1.902898 0.267296
C13 4.168937 3.219525 0.419421
C14 2.809223 3.506185 0.345734
C15 1.896827 2.467658 0.131872
N16 2.331259 1.193584 0.007817
C17 3.64076 0.910192 0.064014
H18 5.644376 1.635369 0.31229
H19 4.884257 4.018947 0.58926
H20 2.469453 4.52861 0.452813
H21 -3.53212 0.728872 -0.767538
H22 -2.105394 -0.165143 -1.376091
H23 -2.468521 -0.184025 0.338834
O24 2.66019 -1.108716 2.047383
C25 2.676204 -1.935876 1.142118
O26 2.151063 -1.800438 -0.049533
C27 4.625868 -5.702046 1.861942
C28 3.270002 -5.560649 2.15185
C29 2.644464 -4.339124 1.871692
C30 3.368963 -3.270761 1.323814
C31 4.727042 -3.44134 1.039123
C32 5.366333 -4.660082 1.30452
H33 5.118918 -6.647826 2.072567
H34 2.713924 -6.386547 2.580741
O35 1.326598 -4.077897 2.087923
O36 5.360619 -2.346014 0.502999
H37 6.418391 -4.80314 1.088128
C38 0.551907 -4.98139 2.856254
C39 6.741914 -2.45419 0.211349
H40 -0.419265 -4.497038 2.969522
H41 0.425455 -5.946357 2.345694
H42 1.000463 -5.153176 3.843986
H43 7.042578 -1.486069 -0.196169
H44 7.331912 -2.664599 1.113658
H45 6.939229 -3.234295 -0.536095
C46 -1.056942 -2.324296 0.283506
O47 -0.455734 -1.689341 -0.690361
O48 -1.195742 -1.912936 1.434698
C49 -1.604564 -3.679447 -0.155876
H50 -2.384151 -4.010151 0.534971
H51 -1.992159 -3.642005 -1.17813
H52 -0.780526 -4.402021 -0.13823
H53 3.910522 -0.129508 -0.062251

III

atom x y z
Pd1 0.697214 0.132891 -0.073762
C2 -1.525574 4.383227 0.582886
C3 -2.227705 3.226007 0.890332
C4 -1.627666 1.969884 0.720703
N5 -0.361585 1.891012 0.257256
C6 0.343781 3.018278 -0.048515
C7 -0.219986 4.280983 0.106349
H8 -1.985765 5.358052 0.711546
H9 -3.244708 3.275302 1.263465
C10 -2.368141 0.704273 1.043196
H11 0.344671 5.17235 -0.138927
C12 4.24635 2.106919 -1.436365
C13 3.887772 3.452061 -1.355849
C14 2.615297 3.801066 -0.905928
C15 1.717943 2.795387 -0.542708
N16 2.091305 1.495225 -0.631632
C17 3.312322 1.145761 -1.061966
H18 5.227853 1.800778 -1.780969
H19 4.592252 4.227531 -1.640139
H20 2.330256 4.844323 -0.839715
H21 -3.362023 0.936463 1.432507
H22 -2.478286 0.075117 0.153991
H23 -1.82854 0.107078 1.784617
O24 -0.424827 -1.555397 0.37089
C25 0.56973 -2.303284 0.044879
O26 1.625229 -1.660886 -0.340652
C27 0.433702 -6.549333 0.214114
C28 1.010296 -5.937809 -0.896265
C29 1.061877 -4.541658 -0.955756
C30 0.52236 -3.7644 0.102365
C31 -0.062482 -4.418403 1.217933
C32 -0.099947 -5.815402 1.270442
H33 0.399101 -7.63445 0.257626
H34 1.40733 -6.544197 -1.699989
O35 1.569652 -3.85422 -2.003829
O36 -0.521863 -3.617999 2.206288
H37 -0.532533 -6.329079 2.119143
C38 2.086337 -4.578777 -3.117796
C39 -1.078442 -4.216665 3.374558
H40 2.425715 -3.822053 -3.826219
H41 2.93299 -5.209866 -2.823262
H42 1.310471 -5.196152 -3.585663
H43 -1.366375 -3.385748 4.019762
H44 -1.964556 -4.814343 3.130895
H45 -0.341075 -4.842447 3.890964
H46 3.516025 0.08037 -1.097266

IV

atom x y z
Pd1 -0.309435 0.311572 -0.001848
O2 -2.314843 0.278419 -0.004454
C3 -2.518221 -1.014086 -0.006634
O4 -3.561974 -1.615653 -0.009496
C6 4.532802 0.455896 -0.001296
C7 3.753308 1.608201 -0.000685
C8 2.3582 1.506676 -0.000406
N9 1.763317 0.286116 -0.00075
C10 2.514445 -0.826817 -0.001263
C11 3.903975 -0.787119 -0.001548
H12 5.615836 0.531395 -0.001532
H13 4.233459 2.578535 -0.000387
H15 4.47098 -1.71166 -0.001979
C16 1.031199 5.041798 0.002746
C17 1.936614 3.984403 0.001114
C18 1.454075 2.677338 0.000582
N19 0.116892 2.414223 0.001358
C19 -0.776047 3.430431 0.003301
C21 -0.325576 4.760526 0.003994
H22 1.385819 6.067968 0.003185
H23 3.000611 4.183964 0.00039
H25 -1.05891 5.55928 0.005527
C26 0.515614 -4.00721 -0.002879
C27 0.11916 -3.474548 1.227091
C28 -0.725227 -2.36246 1.234618
C29 -1.12869 -1.73426 -0.00477
C30 -0.721129 -2.361573 -1.243275
C31 0.123216 -3.473689 -1.233809
H32 1.156032 -4.885996 -0.002144
H33 0.437907 -3.948211 2.147267
O34 -1.240571 -1.808957 2.333811
O35 -1.232749 -1.807176 -2.343767
H36 0.444948 -3.946653 -2.153319
C40 -0.974325 -2.390295 3.618044
C41 -0.962057 -2.387178 -3.627686

H42 -1.539259 -1.788089 4.328425
H43 -1.32139 -3.427785 3.650867
H44 0.094455 -2.338966 3.852945
H45 -1.52457 -1.784217 -4.339351
H46 0.107532 -2.335534 -3.858826
H47 -1.308978 -3.424644 -3.662781
H48 1.977345 -1.767367 -0.001498
C46 -2.247781 3.142097 0.004912
H49 -2.811012 4.078013 0.008061
H50 -2.538751 2.555068 -0.870436
H51 -2.535952 2.550861 0.878357

TS-I

atom x y z
Pd1 -0.360172 0.317866 -4.7e-05
O2 -2.428362 0.099107 -0.000139
C3 -2.594544 -1.131955 6.4e-05
O4 -3.390454 -2.002557 0.000229
C6 4.457575 0.594638 -0.000638
C7 3.648718 1.726582 0.000147
C8 2.256749 1.592649 0.000389
N9 1.691757 0.355984 5.9e-05
C10 2.47235 -0.738726 -0.000788
C11 3.860024 -0.663127 -0.001185
H12 5.538335 0.697369 -0.000905
H13 4.103471 2.709051 0.000411
H15 4.448723 -1.574007 -0.001955
C16 0.845793 5.100975 0.001028
C17 1.779793 4.06842 0.001258
C18 1.329298 2.748504 0.000631
N19 0.002632 2.454297 -4.5e-05
C19 -0.913376 3.445682 -0.000355
C21 -0.505792 4.787127 0.000147
H22 1.174188 6.135803 0.00147
H23 2.837866 4.297727 0.001899
H25 -1.258668 5.567664 -0.000153
C26 -0.32526 -4.484093 0.000202
C27 -0.392811 -3.819437 1.2265
C28 -0.542361 -2.430772 1.228679
C29 -0.673018 -1.707712 7.3e-05
C30 -0.542447 -2.430911 -1.228472
C31 -0.392904 -3.819576 -1.226166
H32 -0.210617 -5.564859 0.000263
H33 -0.325258 -4.38439 2.147961
O34 -0.609831 -1.679084 2.346359
O35 -0.609968 -1.679341 -2.346231
H36 -0.325437 -4.38462 -2.14758
C40 -0.646777 -2.329741 3.622427
C41 -0.647024 -2.330118 -3.622234
H42 -0.7574 -1.529646 4.354063
H43 -1.500188 -3.013063 3.687717
H44 0.284642 -2.875035 3.81132
H45 -0.757721 -1.530093 -4.353937
H46 0.284382 -2.875424 -3.811169
H47 -1.500432 -3.013457 -3.687392
H48 1.957904 -1.690486 -0.001134
C46 -2.372931 3.090336 -0.001289
H49 -2.985778 3.994663 -0.001823
H50 -2.632791 2.49339 -0.88103
H51 -2.633982 2.493632 0.878264

V

atom x y z
C1 -1.206655 3.82515 1.387915
C2 0.156034 3.611847 1.209269
C3 0.617566 2.401928 0.680261
N4 -0.270393 1.428029 0.341208
C5 -1.590055 1.631686 0.513658
C6 -2.097203 2.817172 1.032271
H7 -1.561804 4.765311 1.798618
H8 0.855723 4.391064 1.481228
H9 -3.168899 2.933553 1.151511
C10 4.377809 2.764691 0.492052
C11 3.046521 3.067589 0.759499
C12 2.058951 2.128598 0.452959
N13 2.374337 0.923807 -0.087262
C14 3.660343 0.630332 -0.369708
C15 4.68586 1.540978 -0.085499
H16 5.159788 3.481155 0.724635
H17 2.794266 4.024417 1.197241
H18 5.711374 1.277832 -0.321062
H19 -2.237141 0.814211 0.229651
C20 3.978483 -0.696907 -0.999912
H21 5.025309 -0.733355 -1.310409
H22 3.805497 -1.515866 -0.29377
H23 3.351472 -0.875669 -1.878533
Pd24 0.55819 -0.32486 -0.354815
C25 -3.713954 -2.410965 -0.876134
C26 -3.260944 -1.529307 -1.857393
C27 -2.001189 -0.929662 -1.706053
C28 -1.21848 -1.219945 -0.578217
C29 -1.684694 -2.120867 0.387353
C30 -2.945529 -2.720064 0.245539
H31 -4.68983 -2.873756 -0.991682
H32 -3.879987 -1.320638 -2.721971
O33 -1.46453 -0.035082 -2.5905
O34 -0.825377 -2.368798 1.430688
H35 -3.323655 -3.416551 0.98447
C36 -2.202137 0.282686 -3.768614
C37 -1.253151 -3.258041 2.462565
H38 -1.5841 0.990571 -4.322692
H39 -2.375917 -0.608754 -4.382971
H40 -3.163808 0.751213 -3.525011
H41 -0.437094 -3.286805 3.186045
H42 -2.161545 -2.888681 2.95254
H43 -1.430806 -4.266283 2.06978
O44 1.42895 -2.358447 -0.858407
C45 1.013766 -3.435633 -0.610374
O46 0.661455 -4.516757 -0.392022

VI

atom x y z
Pd1 0.305586 0.078091 -0.28244
C2 -0.987099 4.61653 -1.274502
C3 -1.7962 3.576901 -1.720272
C4 -1.388235 2.264106 -1.50328
N5 -0.230982 1.969309 -0.88192
C6 0.577064 2.974346 -0.442398
C7 0.20744 4.308212 -0.630568
H8 -1.277801 5.65263 -1.418162
H9 -2.736917 3.764647 -2.226288
C10 4.139711 1.694569 1.417273
C11 4.009386 3.046967 1.133761
C12 2.842267 3.500543 0.52441
C13 1.833252 2.582365 0.232011
N14 1.964359 1.263341 0.527794
C15 3.099275 0.81103 1.099019
H16 5.042947 1.306132 1.875918
H17 4.810355 3.741419 1.368162
C18 -2.035254 -3.728181 -1.725059
C19 -2.419503 -2.619602 -2.48299
C20 -1.890156 -1.343221 -2.222941
C21 -0.979117 -1.19611 -1.166568
C22 -0.624418 -2.334354 -0.462847
C23 -1.108398 -3.61309 -0.687348
H24 -2.458548 -4.699589 -1.961551
H25 -3.12853 -2.760447 -3.29026

O26	-2.212347	-0.237783	-2.956641
O27	0.373439	-2.005366	0.504319
H28	-0.779849	-4.471967	-0.11194
C29	-3.103552	-0.378358	-4.063039
C30	-0.016516	-2.273669	1.874565
H31	-3.189111	0.616772	-4.502364
H32	-2.698364	-1.070549	-4.809554
H33	-4.093256	-0.720868	-3.738578
H34	0.777619	-1.881869	2.509076
H35	-0.967737	-1.78401	2.101952
H36	-0.102685	-3.354084	2.01468
C37	3.233649	-0.65507	1.406034
H38	2.845357	-0.869692	2.410074
H39	2.689676	-1.274574	0.6903
H40	4.285791	-0.952873	1.396713
H48	0.840715	5.104834	-0.26068
H49	2.737634	4.546334	0.264843
H50	-1.972191	1.422672	-1.846955

VII

atom	x	y	z
Pd1	0.822354	0.296337	-0.196447
C2	3.120542	3.345836	2.907488
C3	1.893797	3.623981	2.315381
C4	1.389849	2.756368	1.343598
N5	2.053862	1.617168	0.99077
C6	3.280457	1.381166	1.508382
C7	3.827966	2.233735	2.478185
H8	3.526983	4.004984	3.669439
H9	1.344702	4.512301	2.600122
H10	4.80952	2.006968	2.88109
C11	-1.678943	4.522066	-0.027171
C12	-0.581931	4.258673	0.788929
C13	0.148814	3.080153	0.591399
N14	-0.212836	2.194803	-0.361609
C15	-1.25642	2.452834	-1.162456
C16	-2.016193	3.614616	-1.028199
H17	-2.253793	5.433056	0.114721
H18	-0.303486	4.96852	1.558148
H19	-2.850693	3.794575	-1.697774
C20	4.082442	0.210836	1.019885
H21	-1.462753	1.715412	-1.928409
H22	5.135427	0.344136	1.287682
H23	3.72478	-0.723143	1.463197
H24	3.993675	0.102543	-0.063926
C25	2.543892	-4.178348	0.281501
C26	2.940374	-3.396669	-0.802917
C27	2.491322	-2.068485	-0.884018
C28	1.662721	-1.513831	0.094934
C29	1.284253	-2.322197	1.174316
C30	1.721913	-3.653191	1.279512
H31	2.887202	-5.207467	0.358008
H32	3.594756	-3.810632	-1.563592
O33	2.880735	-1.213865	-1.889778
O34	0.472126	-1.725379	2.113484
H35	1.427207	-4.277277	2.116108
C36	3.204355	-1.769104	-3.154943
C37	-0.084804	-2.535642	3.129064
H38	3.277615	-0.923925	-3.842335
H39	4.165928	-2.303272	-3.139751
H40	2.412305	-2.445452	-3.49733
H41	-0.752281	-1.886448	3.700837
H42	-0.664115	-3.370272	2.710925
H43	0.684337	-2.939817	3.803697
O44	-0.101306	0.178273	-3.112754
C45	-0.719742	-0.635538	-2.426747
O46	-0.618858	-0.819177	-1.145648
C47	-3.70304	-3.152395	-4.278083
C48	-3.974761	-1.799908	-4.081857
C49	-3.002055	-0.994903	-3.474952
C50	-1.772166	-1.528395	-3.065329
C51	-1.527964	-2.89352	-3.276363
C52	-2.490606	-3.712202	-3.884046
H53	-4.453205	-3.78236	-4.749607
H54	-4.928487	-1.391236	-4.393905
O55	-3.183571	0.341851	-3.217592
O56	-0.304165	-3.340889	-2.869377
H57	-2.300686	-4.76559	-4.052861
C58	-4.396931	0.941965	-3.627307
C59	-0.057252	-4.735951	-2.869698
H60	-4.323227	1.99549	-3.346371
H61	-4.539206	0.870697	-4.714038
H62	-5.264639	0.495993	-3.121992
H63	0.913639	-4.861321	-2.387877
H64	-0.82007	-5.277961	-2.295149
H65	-0.020299	-5.142997	-3.890284

VIII

atom	x	y	z
Pd1	0.411548	0.15987	0.009019
N2	2.421051	-0.30304	-0.049312
C3	3.468223	-0.805201	-0.008858
N4	4.685865	-1.324464	-0.028724
C5	-4.35805	1.112423	-0.000463
C6	-3.402339	2.122117	-0.027455
C7	-2.03965	1.803188	-0.019195
N8	-1.641355	0.504366	0.016435
C9	-2.567126	-0.471258	0.043625
C10	-3.932602	-0.21126	0.03639
H11	-5.415046	1.360825	-0.006801
H12	-3.723662	3.155117	-0.052206
H13	-4.635436	-1.037073	0.059729
C14	-0.262734	5.136041	-0.098557
C15	-1.293505	4.20312	-0.098086
C16	-0.980328	2.841925	-0.03944
N17	0.305353	2.40983	0.009728
C18	1.309719	3.310606	0.01704
C19	1.048624	4.685559	-0.036583
H20	-0.48366	6.198231	-0.143887
H21	-2.320677	4.540143	-0.143762
H22	1.877787	5.384705	-0.02932
C23	-0.28501	-4.582225	0.016591
C24	-0.189511	-3.912193	-1.202325
C25	0.04622	-2.527204	-1.201754
C26	0.17199	-1.834094	0.009086
C27	0.083837	-2.526897	1.223327
C28	-0.152761	-3.911578	1.231739
H29	-0.470169	-5.652857	0.019628
H30	-0.299672	-4.46206	-2.129738
O31	0.145279	-1.770849	-2.33967
O32	0.21928	-1.770725	2.358119
H33	-0.234554	-4.461267	2.162204
C34	-0.031607	-2.412521	-3.597257
C35	0.066438	-2.410515	3.619746
H36	0.069872	-1.627417	-4.348307
H37	0.732797	-3.180843	-3.769527
H38	-1.026425	-2.868578	-3.679876
H39	0.186083	-1.62516	4.367952
H40	-0.928071	-2.862821	3.72362
H41	0.831284	-3.181632	3.777061
H42	-2.184463	-1.482271	0.070618
C43	2.726848	2.814551	0.088199
H44	3.42673	3.653235	0.122605
H45	2.875469	2.199918	0.981759
H46	2.966108	2.196299	-0.782499
H47	5.29366	-1.137561	0.761113

H48 4.796991 -2.235947 -0.460032

TS-II

atom x y z
Pd1 0.401307 0.04079 0.205469
C2 -0.902006 4.063193 -2.428761
C3 -1.854639 3.065202 -2.288322
C4 -1.558308 1.910716 -1.548904
N5 -0.3489 1.755581 -0.966847
C6 0.589071 2.732362 -1.092827
C7 0.3381 3.896495 -1.821406
H8 -1.117031 4.961301 -3.000189
H9 -2.833461 3.162766 -2.744964
C10 -2.592358 0.832836 -1.395695
C11 4.268794 1.961404 0.890418
C12 4.131219 3.168332 0.209253
C13 2.935661 3.440206 -0.446994
C14 1.892067 2.506864 -0.417543
N15 2.044252 1.33672 0.251631
C16 3.199131 1.074125 0.885662
H17 5.181919 1.705533 1.416933
H18 4.940945 3.89134 0.186997
H19 -3.518074 1.122476 -1.898506
H20 -2.240615 -0.11237 -1.817889
H21 -2.806258 0.642004 -0.34049
N22 -1.141051 -1.344463 0.21336
C23 -0.674662 -2.199838 0.922215
N24 -0.829413 -3.325392 1.642213
C25 2.99074 -3.087294 2.799041
C26 2.178852 -2.170403 3.468874
C27 1.312998 -1.360225 2.720828
C28 1.246847 -1.481558 1.306213
C29 2.110891 -2.406958 0.659824
C30 2.971966 -3.222992 1.407876
H31 3.663474 -3.714175 3.377921
H32 2.233206 -2.09088 4.54789
O33 0.487759 -0.435032 3.260435
O34 2.022995 -2.438544 -0.691046
H35 3.633581 -3.933856 0.927737
C39 0.388739 -0.334198 4.682524
C40 2.810745 -3.382176 -1.4187
H41 -0.365584 0.430564 4.868683
H42 0.067376 -1.284353 5.124082
H43 1.342352 -0.022672 5.124422
H44 2.551917 -3.231534 -2.467118
H45 3.881611 -3.196903 -1.275017
H46 2.571823 -4.411273 -1.125313
H47 1.092465 4.666458 -1.91968
H48 2.821504 4.377341 -0.97659
H49 3.251897 0.122267 1.398828
H50 -1.678853 -3.85034 1.454957
H51 0.002117 -3.880387 1.803762

IX

atom x y z
Pd1 0.976817 0.173204 -0.072823
C2 -1.749905 4.13961 -1.111304
C3 -2.342273 2.886603 -1.255002
C4 -1.567088 1.758506 -1.007479
N5 -0.279522 1.831357 -0.637629
C6 0.312658 3.041733 -0.502062
C7 -0.412295 4.216769 -0.7367
H8 -2.316245 5.047356 -1.296531
H9 -3.379261 2.779934 -1.555077
C10 4.460105 2.957723 0.287375
C11 3.811965 4.180965 0.356301
C12 2.442029 4.228854 0.11487
C13 1.755625 3.043483 -0.148739
N14 2.391408 1.839851 -0.142057
C15 3.732031 1.786299 0.026731
H16 5.532804 2.886799 0.431231
H17 4.363456 5.090794 0.573448
C18 4.449069 0.474981 -0.09886
H19 5.529537 0.638443 -0.116906
H20 4.192265 -0.200331 0.720489
H21 4.14944 -0.039 -1.017363
N22 2.146109 -1.288305 0.606673
C23 1.281567 -2.221351 0.666891
N24 1.498079 -3.527557 1.036514
C25 -2.811475 -1.692994 -0.554834
C26 -1.863509 -2.007775 -1.528015
C27 -0.514351 -2.09879 -1.159091
C28 -0.114539 -1.815773 0.192815
C29 -1.135847 -1.53429 1.163618
C30 -2.47707 -1.448493 0.778575
H31 -3.85808 -1.644002 -0.845367
H32 -2.179728 -2.208906 -2.54389
O33 0.4687 -2.476949 -1.985921
O34 -0.70182 -1.416859 2.429393
H35 -3.255059 -1.226424 1.49813
C36 0.17117 -2.741003 -3.361252
C37 -1.63469 -1.081287 3.462589
H38 1.123695 -3.013256 -3.814814
H39 -0.534963 -3.572737 -3.457264
H40 -0.230727 -1.847067 -3.851505
H41 -1.044931 -1.022094 4.377034
H42 -2.105308 -0.112302 3.262327
H43 -2.402027 -1.856207 3.566424
H44 0.060814 5.186288 -0.644375
H45 1.923255 5.178765 0.135811
H46 -1.976343 0.759508 -1.105974
H47 0.713949 -4.020275 1.4452
H48 2.385343 -3.701795 1.496007

X

atom x y z
Pd1 0.735434 -0.353094 -0.338438
C2 -2.216479 3.105528 1.300357
C3 -2.684577 1.794974 1.223508
C4 -1.853513 0.821368 0.679351
N5 -0.63036 1.113529 0.211455
C6 -0.158283 2.381597 0.267411
C7 -0.944149 3.399814 0.821533
H8 -2.829832 3.891377 1.732328
H9 -3.669197 1.52117 1.588522
C10 3.606089 2.940372 -1.553361
C11 3.00071 4.044525 -0.974086
C12 1.764879 3.884508 -0.354285
C13 1.191644 2.612408 -0.307114
N14 1.821576 1.527255 -0.825894
C15 2.993529 1.679738 -1.471447
H16 4.555598 3.031289 -2.0715
H17 3.470018 5.0235 -1.018176
C18 3.645615 0.492409 -2.113369
H19 4.367251 0.04298 -1.424422
H20 2.917094 -0.281971 -2.355346
H21 4.174751 0.80237 -3.021567
N22 -0.535964 -1.780598 0.210852
C23 -1.036787 -2.523014 -0.696589
N24 -2.016988 -3.480484 -0.366502
C25 0.049844 -2.211379 -4.847057
C26 0.583718 -3.236627 -4.072883
C27 0.179345 -3.35943 -2.73646
C28 -0.724338 -2.448728 -2.169337
C29 -1.263718 -1.435479 -2.982112
C30 -0.879868 -1.312593 -4.322846
H31 0.354168 -2.112971 -5.886314

H32 1.29587 -3.932729 -4.501419
O33 0.59682 -4.365443 -1.9152
O34 -2.158241 -0.598141 -2.372422
H35 -1.289104 -0.532019 -4.953297
C36 1.892831 -4.910076 -2.138488
C37 -2.643251 0.512819 -3.102712
H38 2.11637 -5.52124 -1.262184
H39 1.918084 -5.545104 -3.035073
H40 2.63495 -4.109934 -2.216377
H41 -3.287564 1.06483 -2.414713
H42 -1.825579 1.166079 -3.436521
H43 -3.232829 0.201773 -3.975935
O44 3.452929 -0.697419 0.860496
C45 3.161952 -1.709724 0.219796
O46 2.166424 -1.853191 -0.598583
C47 5.664959 -5.188649 0.802246
C48 6.035338 -4.245041 -0.153173
C49 5.212684 -3.128173 -0.359153
C50 4.030901 -2.952802 0.371389
C51 3.688913 -3.917543 1.332944
C52 4.49974 -5.040413 1.551724
H53 6.299218 -6.056101 0.967905
H54 6.948029 -4.382415 -0.721086
O55 5.491544 -2.148211 -1.279329
O56 2.537689 -3.667303 2.01902
H57 4.235937 -5.784115 2.294663
C58 6.717598 -2.215688 -1.98225
C59 2.148095 -4.559669 3.043809
H60 6.752114 -1.32445 -2.613003
H61 7.578062 -2.209135 -1.299373
H62 6.774668 -3.108816 -2.619887
H63 1.225334 -4.149767 3.458702
H64 1.952609 -5.568532 2.653429
H65 2.906793 -4.624296 3.836311
H66 -0.567653 4.413588 0.883859
H67 1.259719 4.740923 0.075381
H68 -2.119817 -0.229257 0.623571
H69 -1.920721 -4.334646 -0.90784
H70 -2.002281 -3.678069 0.630848

Benzoic acid

II

atom x y z
Pd1 0.826952 -0.245463 0.345664
C2 -1.479623 3.791112 -1.014184
C3 -2.27701 2.737131 -0.600552
C4 -1.695558 1.580482 -0.056522
N5 -0.357171 1.488205 0.063827
C6 0.432888 2.551521 -0.263066
C7 -0.102261 3.70775 -0.823633
H8 -1.919295 4.67938 -1.458285
H9 -3.357184 2.782919 -0.694689
C10 -2.585399 0.468282 0.420932
H11 0.539332 4.533356 -1.106301
C12 4.442718 2.053407 1.002342
C13 4.098817 3.266696 0.412346
C14 2.795204 3.459145 -0.037812
C15 1.86627 2.423061 0.086249
N16 2.236117 1.232455 0.619603
C17 3.480137 1.050777 1.089167
H18 5.438831 1.874191 1.392514
H19 4.829396 4.064597 0.316157
H20 2.507299 4.40925 -0.47095
H21 -3.42675 0.891709 0.981082
H22 -2.99564 -0.077967 -0.435693
H23 -2.047793 -0.244916 1.04312
O24 -0.757489 -0.808459 -2.120788
C25 -0.975896 -1.687795 -1.28067
O26 -0.541421 -1.680683 -0.047122
C27 -3.456616 -5.054342 -2.305919
C28 -2.871521 -4.99285 -1.039148
C29 -2.064901 -3.909373 -0.692319
C30 -1.838386 -2.879014 -1.613878
C31 -2.42503 -2.946802 -2.883639
C32 -3.231503 -4.029468 -3.228384
H33 -4.084672 -5.900566 -2.574833
H34 -3.04215 -5.791838 -0.321652
H35 -3.683389 -4.077266 -4.216338
O36 2.672529 -1.542163 2.49049
C37 2.686657 -2.17615 1.426883
O38 2.117753 -1.801752 0.315259
C39 4.880841 -5.869565 1.175998
C40 4.911857 -5.102563 2.343371
C41 4.194721 -3.909938 2.412249
C42 3.443378 -3.474541 1.313948
C43 3.412667 -4.246628 0.145853
C44 4.129677 -5.440703 0.079281
H45 5.439282 -6.80129 1.12215
H46 5.493836 -5.436267 3.199168
H47 4.101227 -6.03885 -0.82826
H48 3.660385 0.094383 1.565627
H49 -1.599884 -3.851811 0.286204
H50 -2.229492 -2.140808 -3.583952
H51 4.197986 -3.298495 3.309116
H52 2.822329 -3.902434 -0.696723

III

atom x y z
Pd1 0.697802 0.133264 -0.071879
C2 -1.449581 4.380415 0.773896
C3 -2.128519 3.223867 1.131396
C4 -1.549096 1.966224 0.908484
N5 -0.324726 1.886457 0.343144
C6 0.357669 3.0136 -0.014388
C7 -0.187307 4.27671 0.192095
H8 -1.894291 5.355989 0.944281
H9 -3.111773 3.274332 1.585819
C10 -2.266722 0.703148 1.287717
H11 0.358855 5.167435 -0.094112
C12 4.123625 2.08594 -1.727751
C13 3.780382 3.432814 -1.61565
C14 2.552487 3.78812 -1.058871
C15 1.683829 2.78724 -0.621357
N16 2.042498 1.484985 -0.742264
C17 3.220346 1.129016 -1.275659
H18 5.070171 1.774661 -2.155524
H19 4.462281 4.204763 -1.958525
H20 2.279213 4.832699 -0.968376
H21 -3.236578 0.938792 1.731337
H22 -2.42702 0.062563 0.41472
H23 -1.684625 0.119107 2.007582
O24 -0.384012 -1.558217 0.481247
C25 0.574897 -2.304337 0.054007
O26 1.591828 -1.667324 -0.435434
C27 0.429882 -6.539815 0.201486
C28 1.55262 -5.902548 -0.335775
C29 1.60497 -4.51375 -0.385799
C30 0.525428 -3.761327 0.105437
C31 -0.601287 -4.402008 0.646335
C32 -0.644771 -5.791388 0.691588
H33 0.392568 -7.62507 0.238843
H34 2.38374 -6.490324 -0.713743
H35 -1.51308 -6.293334 1.107796
H36 3.415801 0.063078 -1.32951
H37 2.46984 -4.004566 -0.79869
H38 -1.427572 -3.806742 1.021423

IV

atom	x	y	z
Pd1	-0.268545	0.266821	-0.045767
O2	-2.266584	0.28469	0.103346
C3	-2.523398	-0.942453	0.528016
O4	-3.536107	-1.326387	1.062821
C5	4.549614	0.46082	0.176644
C6	3.763867	1.60551	0.080841
C7	2.373857	1.489206	-0.013239
N8	1.794369	0.261893	-0.015821
C9	2.547544	-0.843341	0.08288
C10	3.933067	-0.788497	0.180593
H11	5.629121	0.547299	0.251456
H12	4.234542	2.580411	0.087333
H13	4.505536	-1.706206	0.258738
C14	1.00306	4.995054	-0.253456
C15	1.92167	3.952349	-0.164714
C16	1.455948	2.641675	-0.104245
N17	0.119272	2.359089	-0.132171
C18	-0.787619	3.360529	-0.23129
C19	-0.348263	4.693854	-0.288076
H20	1.343304	6.024867	-0.299877
H21	2.982953	4.164002	-0.14815
H22	-1.090962	5.480152	-0.363108
C23	0.548947	-3.812801	-0.407885
C24	0.093953	-3.687994	0.923777
C25	-0.832316	-2.716841	1.258739
C26	-1.326125	-1.839969	0.25853
C27	-0.844909	-1.954607	-1.076728
C28	0.095644	-2.950875	-1.397982
H29	1.246252	-4.606947	-0.661307
H30	0.452727	-4.379666	1.680469
H31	0.426394	-3.068502	-2.425519
H32	2.016747	-1.787975	0.078545
C33	-2.257447	3.069677	-0.290129
H34	-2.813127	4.002188	-0.408897
H35	-2.498533	2.406442	-1.125318
H36	-2.602382	2.561839	0.613815
H37	-1.234717	-2.644446	2.264419
H38	-1.320442	-1.384633	-1.870675

TS-I

atom	x	y	z
Pd1	-0.356009	0.391678	0.011271
O2	-2.402054	0.262876	0.003902
C3	-2.570534	-0.97996	-0.00426
O4	-3.41722	-1.805706	-0.014612
C5	4.475229	0.505732	0.029946
C6	3.70246	1.662893	0.017608
C7	2.307304	1.572069	0.010537
N8	1.704131	0.353685	0.016267
C9	2.449256	-0.765389	0.028127
C10	3.838681	-0.732712	0.035177
H11	5.558628	0.574279	0.035921
H12	4.188044	2.63041	0.015098
H13	4.398519	-1.661497	0.045117
C14	1.00076	5.118606	-0.028693
C15	1.903192	4.058316	-0.021975
C16	1.414622	2.753166	-0.000885
N17	0.078032	2.499393	0.011097
C18	-0.809627	3.517741	0.007196
C19	-0.359031	4.845573	-0.012873
H20	1.359924	6.142907	-0.045608
H21	2.967637	4.254996	-0.033781
H22	-1.088225	5.64812	-0.016001
C23	0.117626	-4.31295	-0.006022
C24	-0.106917	-3.663578	1.215695
C25	-0.547828	-2.344903	1.222791
C26	-0.781275	-1.658434	0.001133
C27	-0.534337	-2.333917	-1.223857
C28	-0.093384	-3.652289	-1.223989
H29	0.453455	-5.346546	-0.008925
H30	0.056462	-4.192576	2.150185
H31	0.080694	-4.172495	-2.161372
H32	1.908586	-1.702149	0.032251
C33	-2.280614	3.215706	0.025276
H34	-2.85726	4.143235	0.033352
H35	-2.576267	2.631461	-0.851439
H36	-2.553966	2.629386	0.907733
H37	-0.741207	-1.844735	2.168378
H38	-0.717155	-1.825123	-2.166827

V

atom	x	y	z
C1	-1.173143	3.857348	1.423034
C2	0.188987	3.634516	1.247825
C3	0.643238	2.413806	0.735601
N4	-0.251753	1.44064	0.410078
C5	-1.571045	1.656397	0.575976
C6	-2.07101	2.85151	1.078832
H7	-1.523393	4.804898	1.820626
H8	0.892357	4.414212	1.508361
H9	-3.14215	2.977263	1.193204
C10	4.408352	2.746575	0.588925
C11	3.075545	3.061657	0.834847
C12	2.085075	2.130339	0.51484
N13	2.399146	0.924204	-0.022406
C14	3.687496	0.613817	-0.272722
C15	4.71644	1.515807	0.026476
H16	5.191912	3.457643	0.831999
H17	2.825727	4.020464	1.269633
H18	5.744021	1.240615	-0.184396
H19	-2.226689	0.844426	0.294596
C20	4.000327	-0.724225	-0.883086
H21	5.068764	-0.812383	-1.093148
H22	3.717904	-1.538665	-0.207582
H23	3.453587	-0.864755	-1.820977
Pd24	0.569519	-0.31976	-0.304936
C25	-3.615898	-2.594418	-0.718267
C26	-3.087335	-1.899338	-1.807542
C27	-1.872319	-1.213066	-1.684923
C28	-1.194516	-1.234182	-0.460694
C29	-1.718671	-1.92577	0.637149
C30	-2.933895	-2.609001	0.499916
H31	-4.558484	-3.124458	-0.818916
H32	-3.616107	-1.886624	-2.757021
H33	-3.34312	-3.149456	1.349521
O34	1.378575	-2.309435	-1.058698
C35	0.764303	-3.299867	-1.26743
O36	0.214717	-4.293964	-1.483803
H37	-1.474379	-0.667826	-2.537801
H38	-1.201742	-1.934909	1.593969

VI

atom	x	y	z
Pd1	0.54401	-0.047917	-0.332371
C2	-1.310426	4.360122	-1.141224
C3	-2.074362	3.24759	-1.482925
C4	-1.545494	1.98195	-1.254811
N5	-0.323794	1.804217	-0.718129
C6	0.440129	2.877776	-0.37488
C7	-0.048617	4.170889	-0.584816
H8	-1.688169	5.364972	-1.302928
H9	-3.063208	3.345585	-1.917365
C10	4.218082	1.936006	1.296221
C11	3.893969	3.278621	1.13485
C12	2.660123	3.626443	0.588247

C13 1.778116 2.609354 0.215823
N14 2.109804 1.306414 0.379027
C15 3.29794 0.956401 0.905058
H16 5.172208 1.639677 1.718556
H17 4.595252 4.053018 1.430482
C18 -2.494634 -3.31705 -1.812234
C19 -1.923015 -2.468376 -2.764292
C20 -1.122781 -1.394223 -2.360441
C21 -0.880757 -1.216882 -0.995156
C22 -1.481832 -2.028667 -0.0279
C23 -2.274869 -3.103199 -0.450623
H24 -3.126505 -4.139808 -2.133752
H25 -2.095662 -2.639917 -3.823343
H26 -2.737091 -3.751134 0.288973
C27 3.580817 -0.514566 1.051479
H28 2.853893 -0.984806 1.725583
H29 3.520997 -1.021011 0.079853
H30 4.578958 -0.690253 1.46036
H31 0.551383 5.03071 -0.314543
H32 2.404654 4.670396 0.457807
H33 -2.094809 1.083668 -1.503015
H34 -0.677565 -0.737193 -3.103967
H35 -1.347614 -1.84 1.036031

VII

atom x y z
Pd1 0.835636 0.301669 -0.336664
C2 3.301692 3.091175 2.837182
C3 2.002683 3.363929 2.42049
C4 1.425852 2.577959 1.422586
N5 2.098944 1.526212 0.873973
C6 3.392022 1.311618 1.205135
C7 4.008304 2.080766 2.202455
H8 3.765097 3.687739 3.61774
H9 1.458322 4.196455 2.848141
H10 5.043075 1.877374 2.458113
C11 -1.928424 4.198406 0.691557
C12 -0.754057 3.880406 1.370497
C13 0.100546 2.911434 0.834422
N14 -0.213799 2.260484 -0.307608
C15 -1.329167 2.582914 -0.975984
C16 -2.213265 3.55652 -0.510672
H17 -2.606164 4.943493 1.098709
H18 -0.521862 4.374421 2.306612
H19 -3.10781 3.790345 -1.078804
C20 4.193679 0.270737 0.473302
H21 -1.47922 2.033587 -1.900598
H22 5.245885 0.572646 0.445624
H23 4.126534 -0.702996 0.967775
H24 3.825799 0.143247 -0.546178
C25 2.614826 -3.996032 0.877816
C26 2.494809 -3.650292 -0.470363
C27 1.985302 -2.401427 -0.838896
C28 1.612252 -1.473271 0.1407
C29 1.723643 -1.827859 1.492049
C30 2.220048 -3.08497 1.85842
H31 3.004702 -4.970818 1.161218
H32 2.790166 -4.359016 -1.241385
H33 2.295363 -3.349345 2.911553
O34 -0.553635 0.603197 -3.134941
C35 -0.87806 -0.453479 -2.572088
O36 -0.505165 -0.840211 -1.389091
C37 -3.548574 -3.181645 -4.57333
C38 -3.223589 -1.95331 -5.154105
C39 -2.357255 -1.079703 -4.499135
C40 -1.811474 -1.424689 -3.257155
C41 -2.138537 -2.658121 -2.679112
C42 -3.003492 -3.532491 -3.336033
H43 -4.222966 -3.864867 -5.084762
H44 -3.644681 -1.679169 -6.118623
H45 -3.252064 -4.489831 -2.883792
H46 1.868091 -2.158437 -1.890551
H47 1.424621 -1.12967 2.271179
H48 -2.085032 -0.122142 -4.932184
H49 -1.705265 -2.916597 -1.718678

VIII

atom x y z
Pd1 0.415977 0.161509 0.009483
N2 2.426978 -0.319622 0.00711
C3 3.461299 -0.840247 0.115806
N4 4.653814 -1.404071 0.167429
C5 -4.363222 1.115649 0.084184
C6 -3.406593 2.124397 0.043346
C7 -2.044688 1.804237 0.007044
N8 -1.647537 0.503784 0.018073
C9 -2.574963 -0.470907 0.057334
C10 -3.93988 -0.209245 0.089585
H11 -5.419337 1.365904 0.113047
H12 -3.727052 3.157885 0.045148
H13 -4.643296 -1.034199 0.120845
C14 -0.274012 5.139683 -0.1486
C15 -1.302133 4.204497 -0.114503
C16 -0.986326 2.84519 -0.038773
N17 0.300806 2.418011 -0.00463
C18 1.303385 3.319933 -0.035456
C19 1.039629 4.69343 -0.106183
H20 -0.499515 6.200124 -0.20787
H21 -2.330473 4.539216 -0.152002
H22 1.866851 5.394446 -0.12891
C23 -0.273756 -4.582427 -0.078938
C24 -0.184976 -3.863319 -1.272673
C25 0.049443 -2.482931 -1.250166
C26 0.185477 -1.826192 -0.022955
C27 0.112376 -2.542331 1.175671
C28 -0.122966 -3.922469 1.142303
H29 -0.46001 -5.652459 -0.100446
H30 -0.30015 -4.372354 -2.22654
H31 -0.189815 -4.477948 2.074594
H32 -2.198309 -1.484232 0.063608
C33 2.721976 2.824265 0.009144
H34 3.423545 3.661904 -0.008846
H35 2.898701 2.242756 0.919806
H36 2.933623 2.174471 -0.845763
H37 5.322526 -1.069219 0.851307
H38 4.731095 -2.380131 -0.096794
H39 0.113412 -1.933805 -2.186183
H40 0.225186 -2.039593 2.132882

TS-II

atom x y z
Pd1 0.42918 0.237424 -0.051491
N2 2.465285 -0.080373 -0.034704
C3 2.459155 -1.280834 0.11865
N4 3.161271 -2.393359 0.396325
C5 -4.405928 0.917045 0.009044
C6 -3.503576 1.974806 0.0486
C7 -2.127047 1.721508 0.026614
N8 -1.668778 0.446545 -0.035044
C9 -2.540607 -0.5738 -0.077173
C10 -3.917595 -0.38523 -0.056838
H11 -5.473972 1.111434 0.023829
H12 -3.87576 2.990484 0.085269
H13 -4.581571 -1.24197 -0.095198
C14 -0.483352 5.119359 0.152424
C15 -1.473021 4.142019 0.188111
C16 -1.11016 2.802202 0.041148

N17	0.188892	2.428984	-0.114481
C18	1.154204	3.37259	-0.17526
C19	0.8338	4.731669	-0.042942
H20	-0.742651	6.167323	0.268003
H21	-2.506784	4.42754	0.332952
H22	1.628821	5.467596	-0.092483
C23	-0.626688	-4.410125	0.062822
C24	-0.262368	-3.819159	-1.152417
C25	0.302498	-2.542116	-1.161992
C26	0.490177	-1.838585	0.047792
C27	0.145244	-2.458293	1.268694
C28	-0.420199	-3.733479	1.271289
H29	-1.059275	-5.406833	0.069267
H30	-0.414653	-4.353432	-2.08626
H31	-0.694281	-4.204258	2.211236
H32	-2.111491	-1.565988	-0.129886
C33	2.580904	2.95764	-0.394698
H34	3.212803	3.837706	-0.536918
H35	2.961322	2.385788	0.456447
H36	2.67128	2.310653	-1.271446
H37	2.771221	-3.276637	0.094567
H38	4.171043	-2.307099	0.330073
H39	0.597433	-2.087238	-2.104247
H40	0.322521	-1.941807	2.207832

IX

atom	x	y	z
Pd1	0.917092	0.172278	-0.028687
C2	-1.607117	4.058241	-1.676117
C3	-2.203643	2.803173	-1.779661
C4	-1.497201	1.696726	-1.319377
N5	-0.272511	1.795289	-0.781784
C6	0.325552	3.005297	-0.68662
C7	-0.330551	4.159118	-1.13128
H8	-2.120877	4.948217	-2.026712
H9	-3.190994	2.677515	-2.210919
C10	4.337878	2.974075	0.615666
C11	3.670051	4.188086	0.603805
C12	2.340245	4.216815	0.192798
C13	1.709651	3.02292	-0.152746
N14	2.353909	1.824683	-0.060766
C15	3.664794	1.790651	0.272048
H16	5.385754	2.917626	0.889826
H17	4.177306	5.104632	0.889285
C18	4.418861	0.494192	0.243576
H19	5.493255	0.689699	0.289202
H20	4.133112	-0.152291	1.076953
H21	4.19325	-0.066503	-0.667052
N22	2.104737	-1.298966	0.613435
C23	1.332349	-2.312545	0.477578
N24	1.714372	-3.630197	0.492505
C25	-2.850918	-1.494851	-0.145923
C26	-2.145193	-2.249109	-1.105596
C27	-0.788301	-2.484165	-0.954571
C28	-0.109184	-1.98385	0.185081
C29	-0.819827	-1.20517	1.137616
C30	-2.196084	-0.965922	0.957574
H31	-3.919529	-1.338962	-0.267291
H32	-2.672684	-2.657814	-1.962692
H33	-2.743639	-0.397763	1.703607
H34	0.154552	5.125583	-1.077394
H35	1.803733	5.156486	0.152744
H36	-1.914767	0.697679	-1.37274
H37	1.042107	-4.306953	0.83351
H38	2.673172	-3.80682	0.771153
H39	-0.33764	-0.929785	2.071344
H40	-0.239328	-3.075239	-1.681303

X

atom	x	y	z
Pd1	0.768349	-0.140675	-0.150232
C2	-2.312656	3.425327	0.978009
C3	-2.674848	2.102444	1.22725
C4	-1.781887	1.093272	0.880523
N5	-0.600973	1.359231	0.302204
C6	-0.22597	2.635434	0.05584
C7	-1.077838	3.69394	0.394952
H8	-2.976373	4.243393	1.243092
H9	-3.623803	1.850133	1.688501
C10	3.628667	3.091521	-1.602694
C11	2.822454	4.216691	-1.515759
C12	1.546943	4.085005	-0.978411
C13	1.114942	2.826821	-0.551597
N14	1.901065	1.723445	-0.662231
C15	3.148869	1.847195	-1.167117
H16	4.634289	3.155499	-2.005021
H17	3.178793	5.184407	-1.857517
C18	4.030558	0.637825	-1.234889
H19	4.223743	0.263613	-0.223547
H20	3.540552	-0.175171	-1.776888
H21	4.981399	0.881652	-1.716983
N22	-0.540997	-1.508592	0.433537
C23	-0.929212	-2.477425	-0.304071
N24	-1.881759	-3.375069	0.211999
C25	0.092716	-3.293906	-4.41426
C26	0.233183	-4.289965	-3.446735
C27	-0.100139	-4.026277	-2.117922
C28	-0.562258	-2.759996	-1.73474
C29	-0.702862	-1.768442	-2.713211
C30	-0.379802	-2.033515	-4.043461
H31	0.34974	-3.498652	-5.450688
H32	0.60892	-5.271765	-3.724499
H33	-0.499791	-1.254774	-4.792978
O34	3.102164	-1.29077	1.34099
C35	2.951489	-1.920777	0.287294
O36	2.077874	-1.652238	-0.640617
C37	5.646869	-5.167536	-0.601545
C38	5.762364	-4.452461	0.593206
C39	4.879314	-3.410679	0.869995
C40	3.871827	-3.07414	-0.042499
C41	3.757571	-3.795241	-1.237714
C42	4.643145	-4.837267	-1.514866
H43	6.337343	-5.979289	-0.819838
H44	6.542489	-4.707303	1.306966
H45	4.551888	-5.392526	-2.445822
H46	-0.777167	4.720046	0.221767
H47	0.89857	4.949066	-0.904802
H48	-1.959973	0.03418	1.056308
H49	-1.910064	-4.285693	-0.228632
H50	-1.875115	-3.417075	1.224979
H51	0.036771	-4.800817	-1.366621
H52	-1.069069	-0.787544	-2.424217
H53	4.948942	-2.837593	1.789315
H54	2.972052	-3.529992	-1.937592

ESI-MS Study: General Procedure

This study used a 3200 Q Trap mass spectrometer manufactured by AB Sciex (Concord, ON, Canada). The reaction mixture was diluted 100 times with methanol and introduced by continuous infusion with the aid of a syringe pump at a flow-rate of 5-10 µL/min through a fused silica capillary (with a 50 µm inner and a 184 µm outer diameter). The ion source used was a Turbo V source in positive ESI mode. The following MS conditions were used: temperature (TEM) 200°C, curtain gas (CUR) 15 psi, ion source gas 1 (GS1) 25 psi, ion source gas 2 (GS2) 15 psi, ion spray voltage (IS) 5500 V, the declustering potential (DP) was 40 V and entrance potential (EP) 10 V for all measurements. MS data were collected in enhanced MS mode (EMS) and MS/MS data were collected in enhanced product ion mode (EPI) and neutral loss mode. The collision gas parameter (CAD) was set to an arbitrary number, 11, for EMS (linear ion trap MS scan) and high for the EPI, which corresponds to a pressure reading of $4.1 \cdot 10^{-5}$ Torr. The collision energy (CE) was 25 eV for all experiments aside from the EMS where it was set to 10 eV. Acquisition and processing of the MS data was performed with Analyst 1.4.2 (AB Sciex).

ESI-MS Study: Observed species

Reaction I: Pd(TFA)2, 6-methyl-2,2'-bipyridyl, TFA, 2,4,6-trimethoxybenzoic acid, N-cyanopiperidine
Reaction II: Pd(TFA)2, 6-methyl-2,2'-bipyridyl, TFA, 2,6-dimethoxybenzoic acid, N-cyanopiperidine
Reaction III: Pd(TFA)2, 6-methyl-2,2'-bipyridyl, TFA, 2,4,6-trimethoxybenzoic acid, N-cyanomorpholine
Reaction IV: Pd(TFA)2, 2,2'-bipyridyl, TFA, 2,4,6-trimethoxybenzoic acid, N-cyanopiperidine

Reaction:	I	II	III	IV
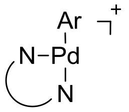	443/445	413/415	443/445	429/431
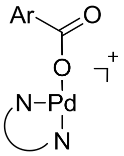	487/489	457/459	487/489	473/475
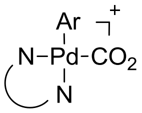	487/489	457/459	487/489	473/475
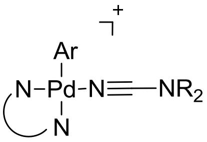	553/555	523/525	555/557	539/541
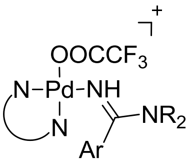	667/669	637/639	669/671	653/655

ESI-MS Study: Spectra

+EMS: 20 MCA scans from Sample 1 (TuneSampleID) of 2.Original_EMS300-1200.wiff (Turbo Spray) Max. 1.3e8 cps.

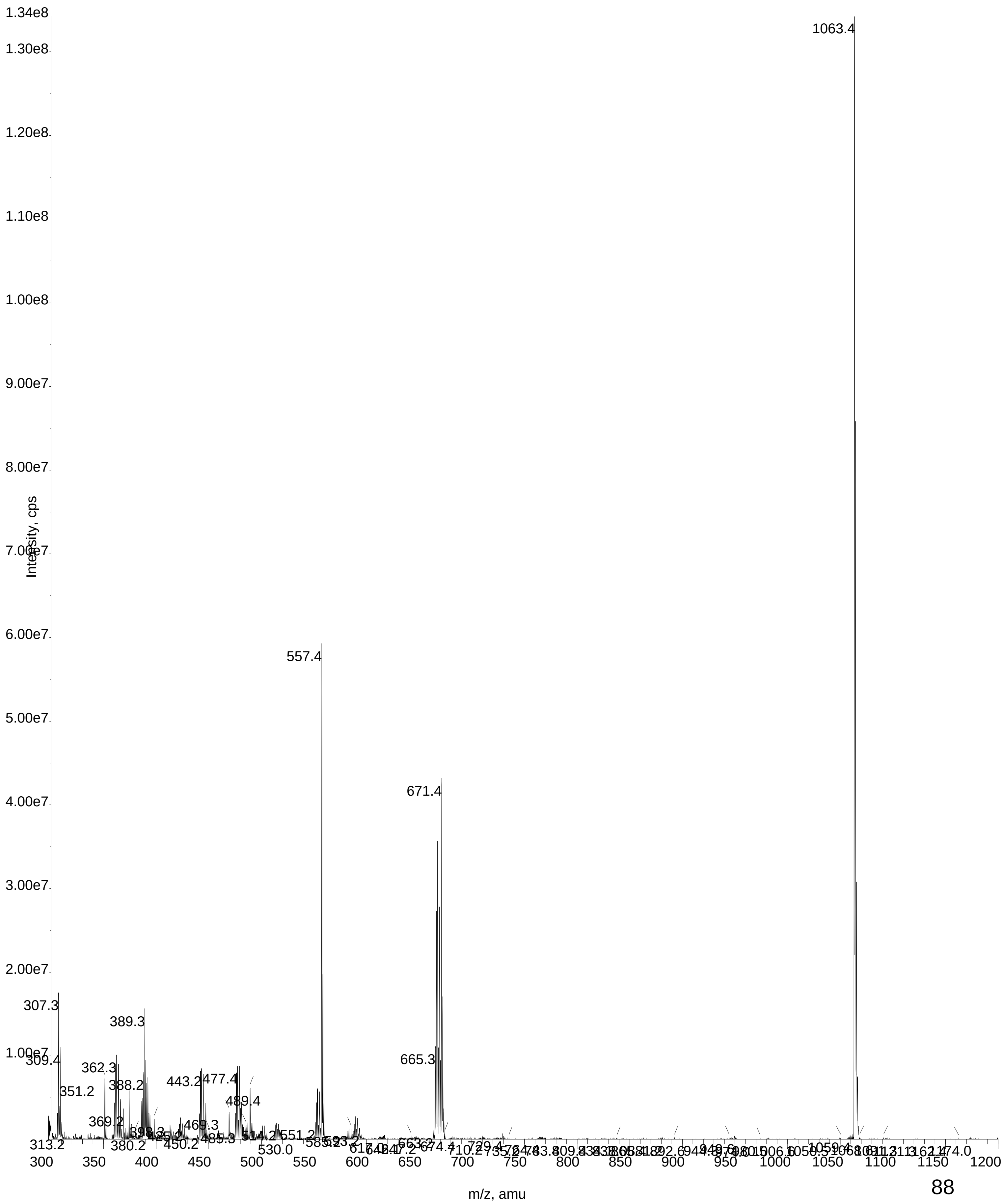

+EMS: 20 MCA scans from Sample 1 (TuneSampleID) of 51.COOH_EMS300-800.wiff (Turbo Spray) Max. 3.9e7 cps.

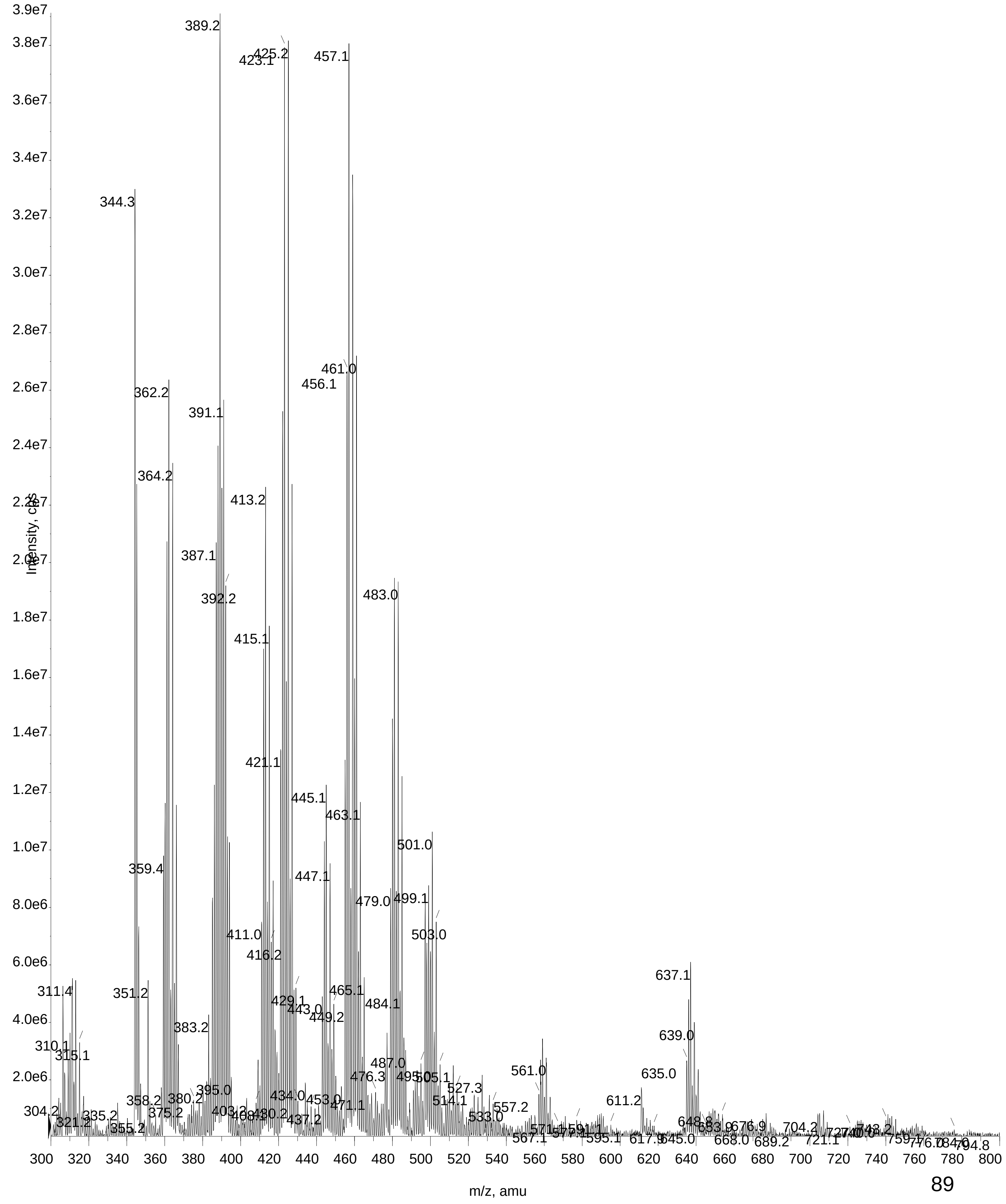

+EMS: 14 MCA scans from Sample 1 (TuneSampleID) of 2.CN_EMS300-1200.wiff (Turbo Spray) Max. 3.6e7 cps.

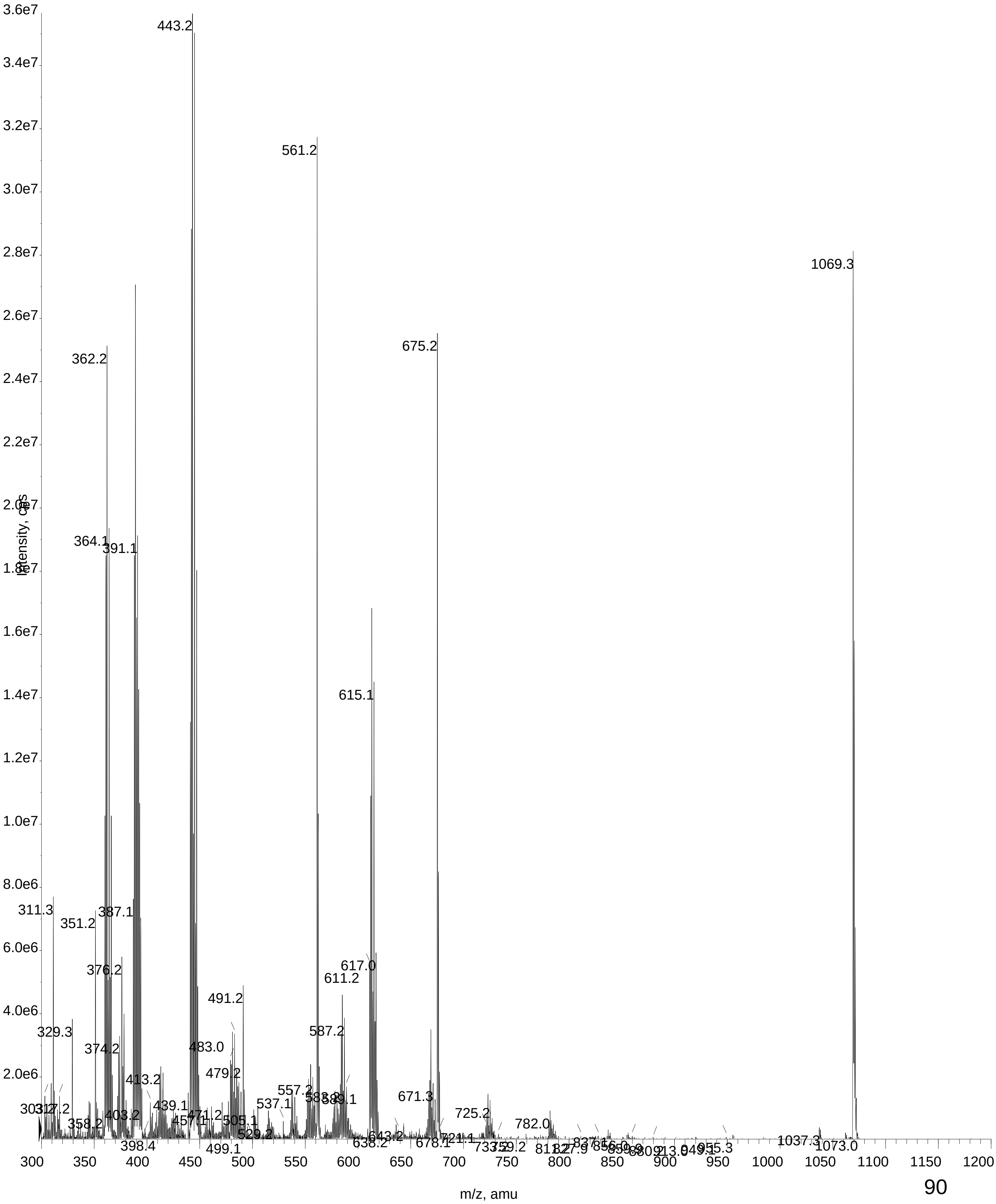

+EMS: 20 MCA scans from Sample 1 (TuneSampleID) of 41.UL_EMS300-1200.wiff (Turbo Spray) Max. 1.4e8 cps.

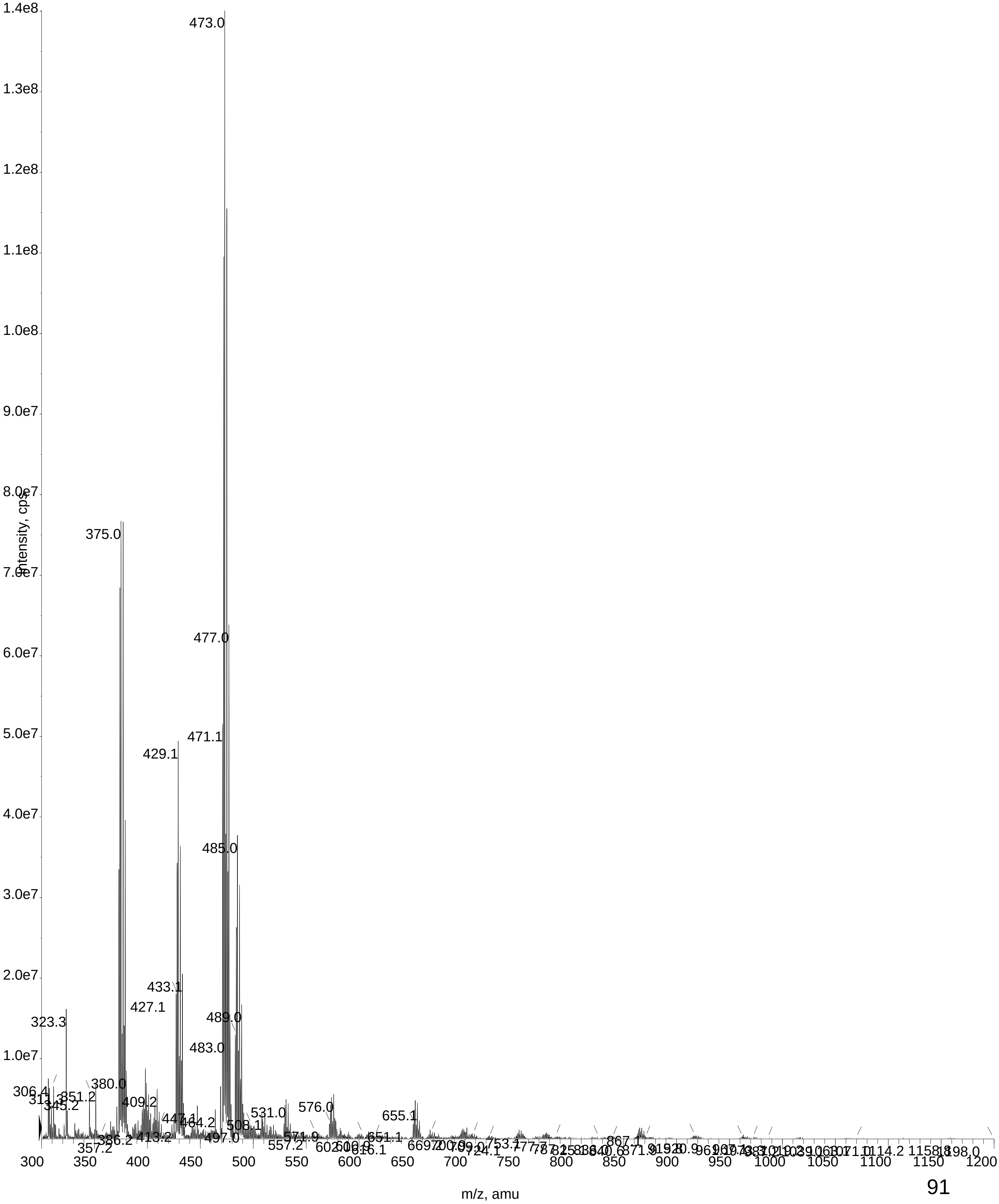

+EMS: 50 MCA scans from Sample 2 (TuneSampleID) of 42.UL_EMS500-1200.wiff (Turbo Spray) Max. 2.2e7 cps.

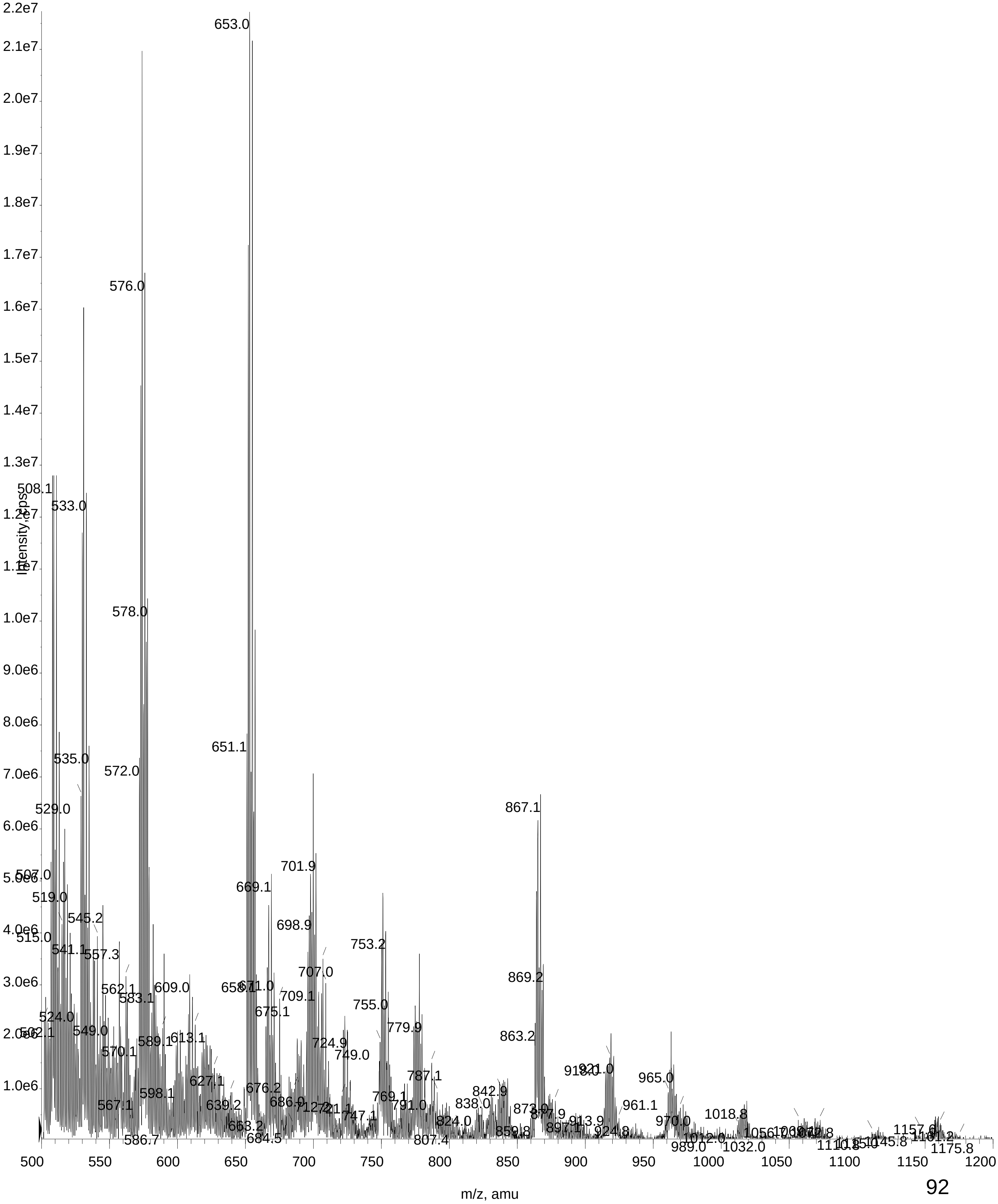

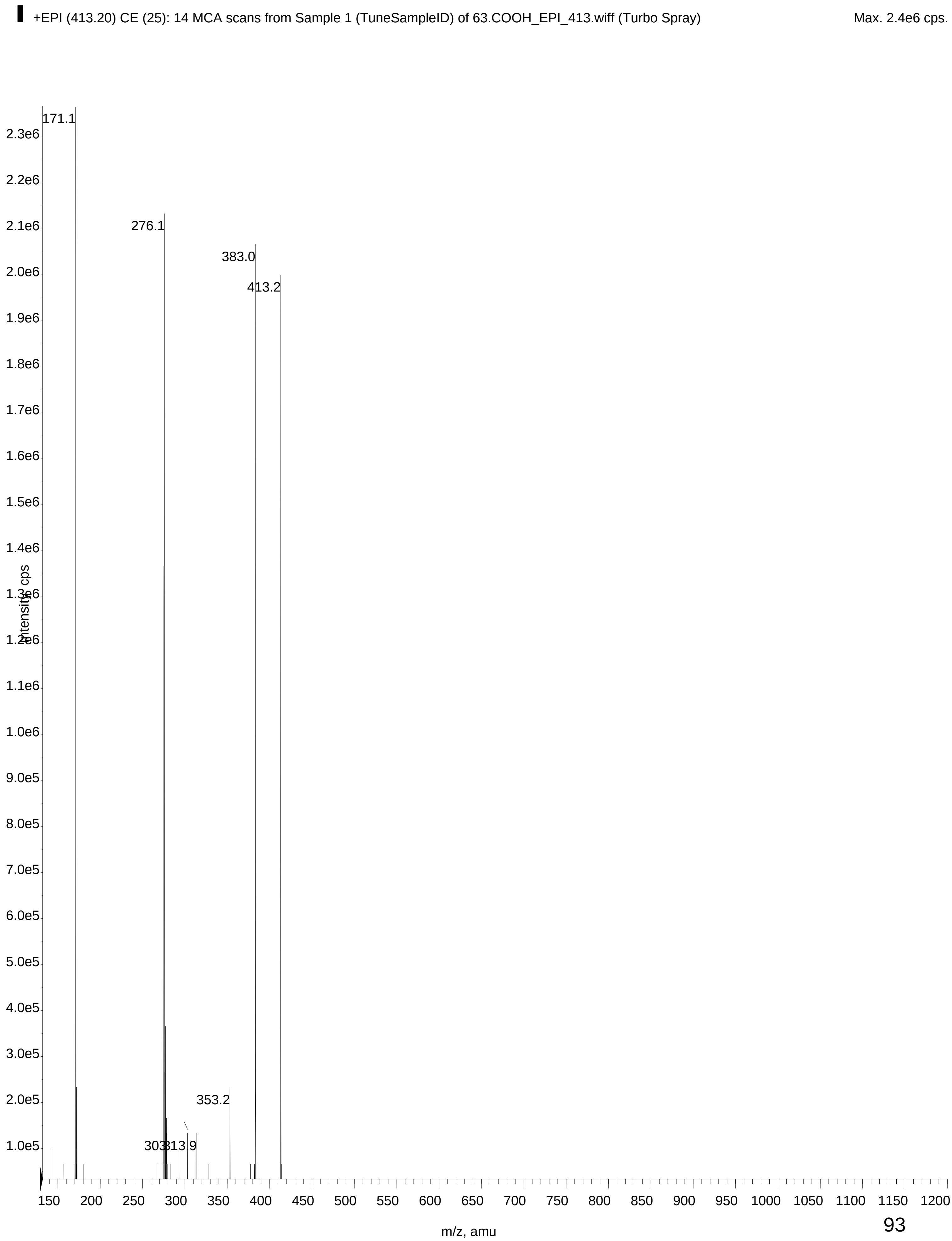
+EPI (413.20) CE (25): 14 MCA scans from Sample 1 (TuneSampleID) of 63.COOH_EPI_413.wiff (Turbo Spray)
Max. 2.4e6 cps.
171.1
276.1
383.0
413.2
353.2
303313.9
Intensity, cps
m/z, amu

+EPI (415.20) CE (25): 16 MCA scans from Sample 1 (TuneSampleID) of 64.COOH_EPI_415.wiff (Turbo Spray) Max. 2.1e6 cps.

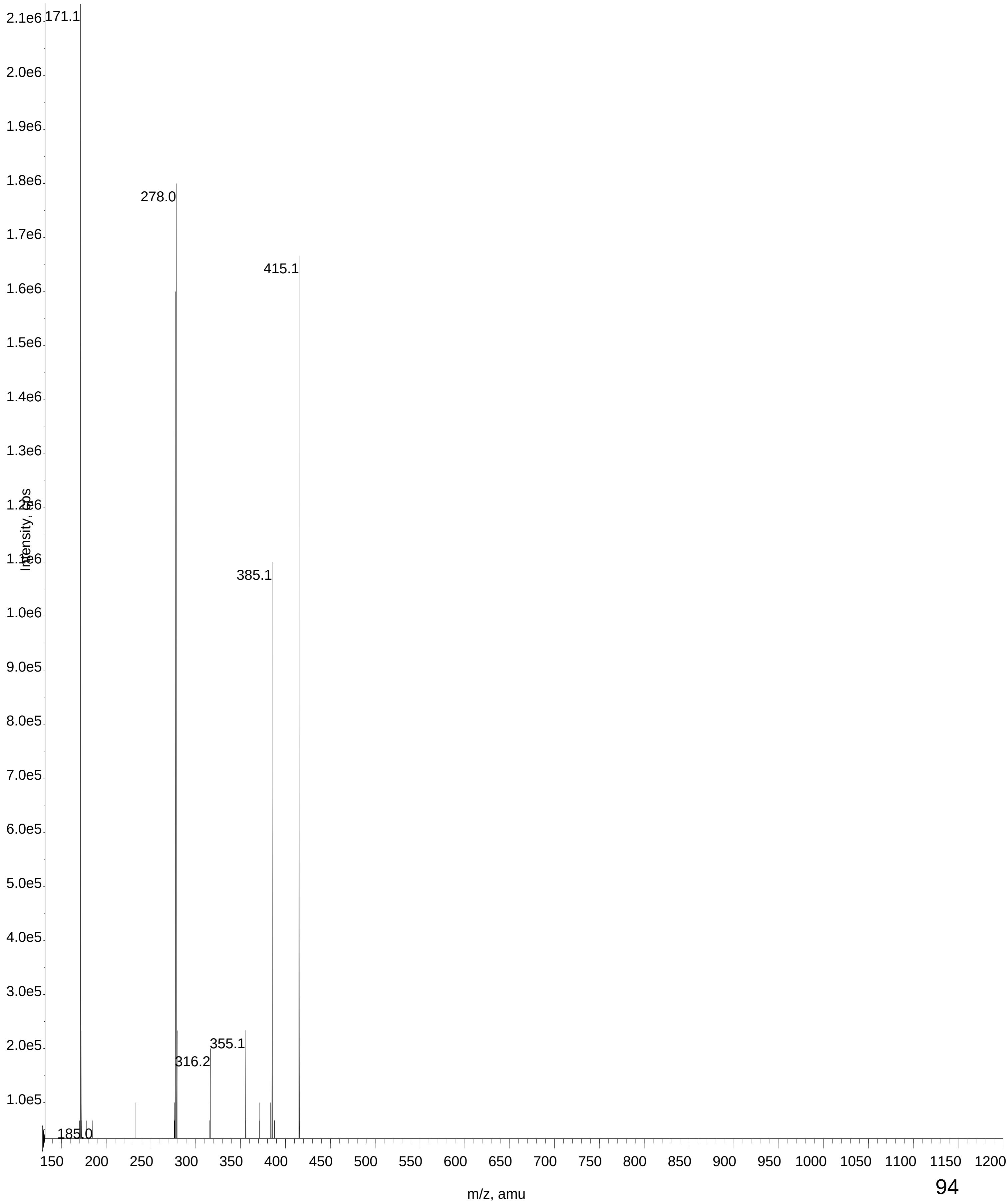

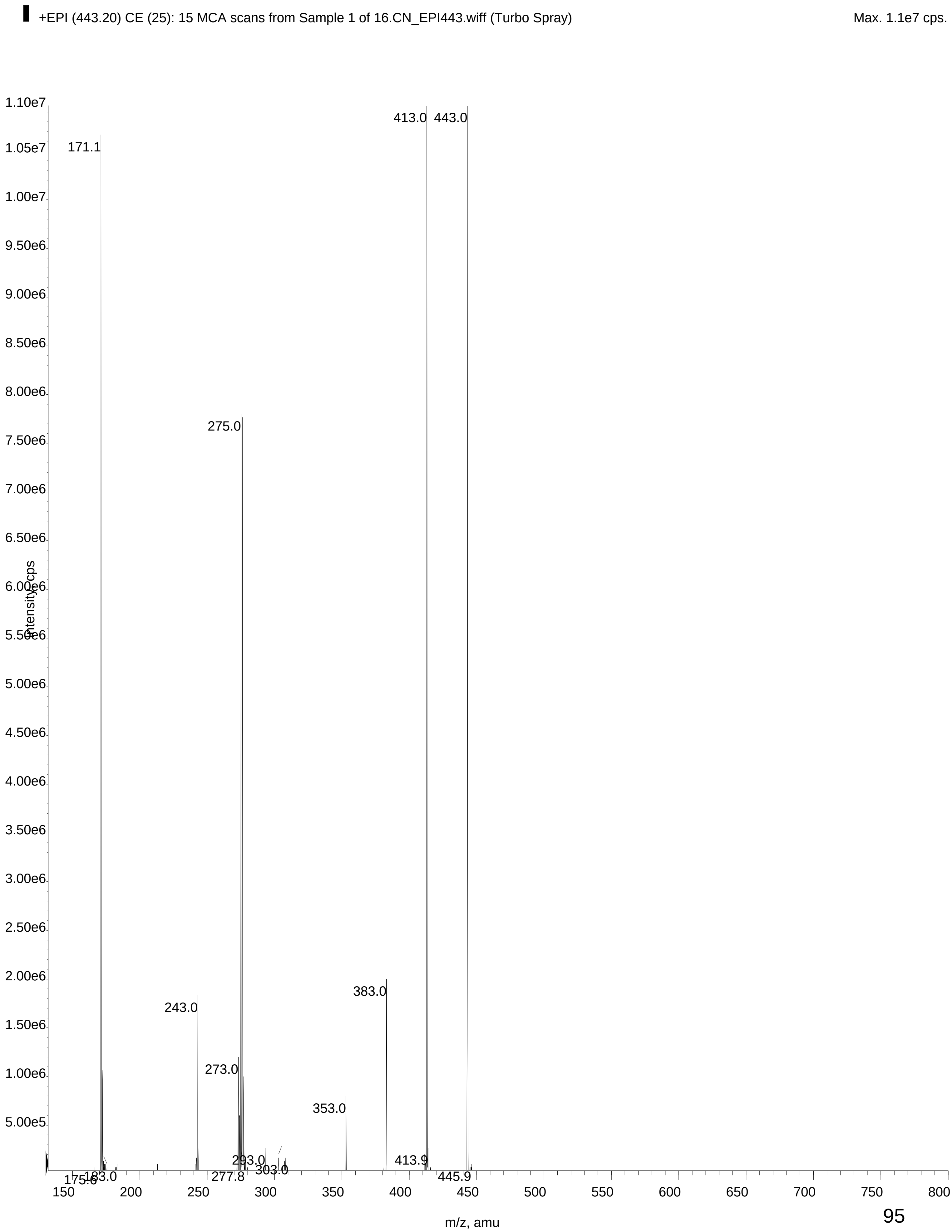
+EPI (443.20) CE (25): 15 MCA scans from Sample 1 of 16.CN_EPI443.wiff (Turbo Spray)
Max. 1.1e7 cps.
Intensity, cps
m/z, amu
171.1
413.0
443.0
275.0
383.0
243.0
273.0
353.0
293.0
303.0
413.9
175.6
183.0
277.8
445.9

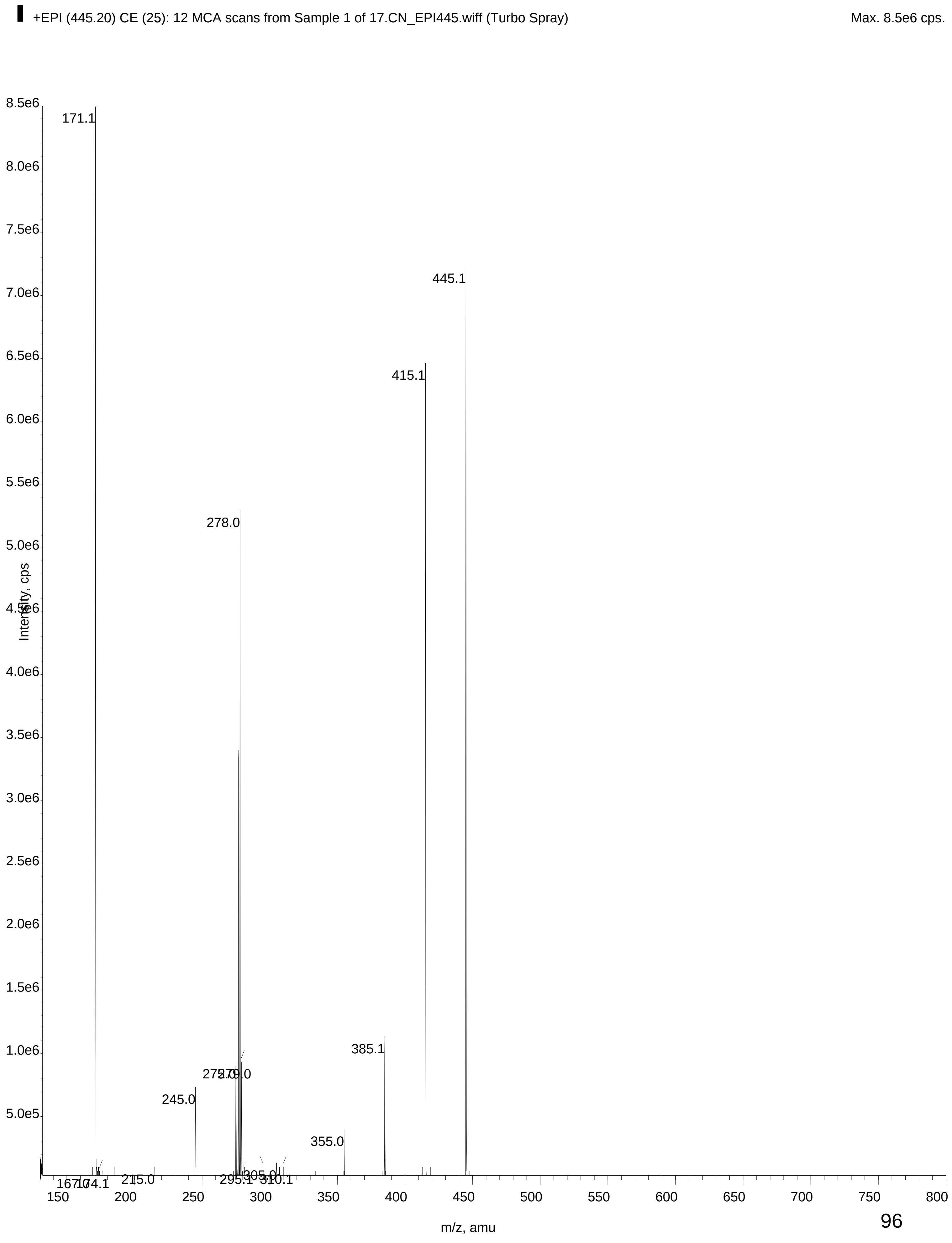
+EPI (445.20) CE (25): 12 MCA scans from Sample 1 of 17.CN_EPI445.wiff (Turbo Spray)
Max. 8.5e6 cps.
Intensity, cps
m/z, amu
171.1
445.1
415.1
278.0
385.1
245.0
355.0
215.0
305.0
310.1
295.1

+EPI (429.00) CE (25): 20 MCA scans from Sample 1 (TuneSampleID) of 47.UL_EPI429.wiff (Turbo Spray)   Max. 9.3e6 cps.

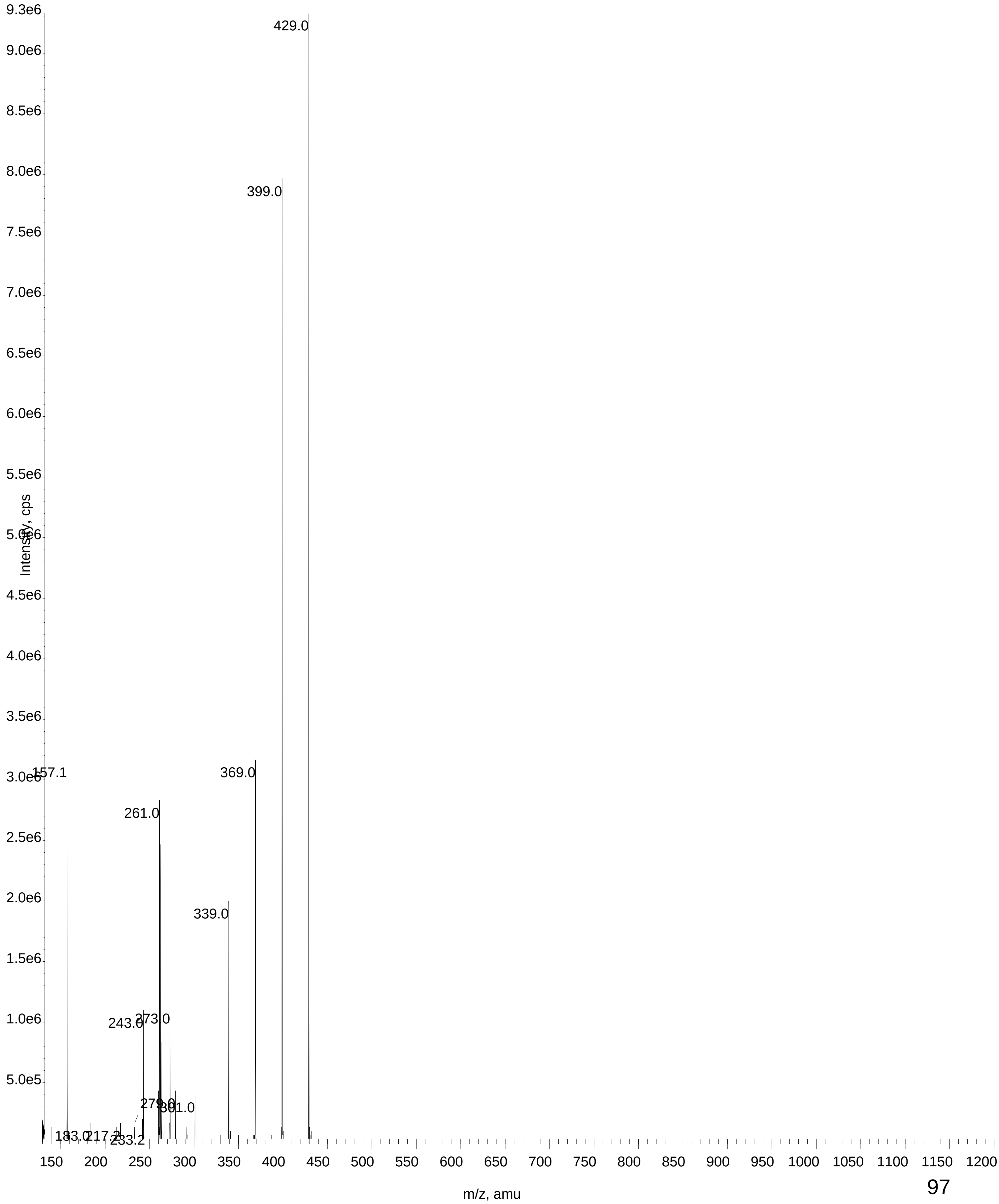

+EPI (431.00) CE (25): 15 MCA scans from Sample 1 (TuneSampleID) of 48.UL_EPI431.wiff (Turbo Spray) Max. 5.7e6 cps.

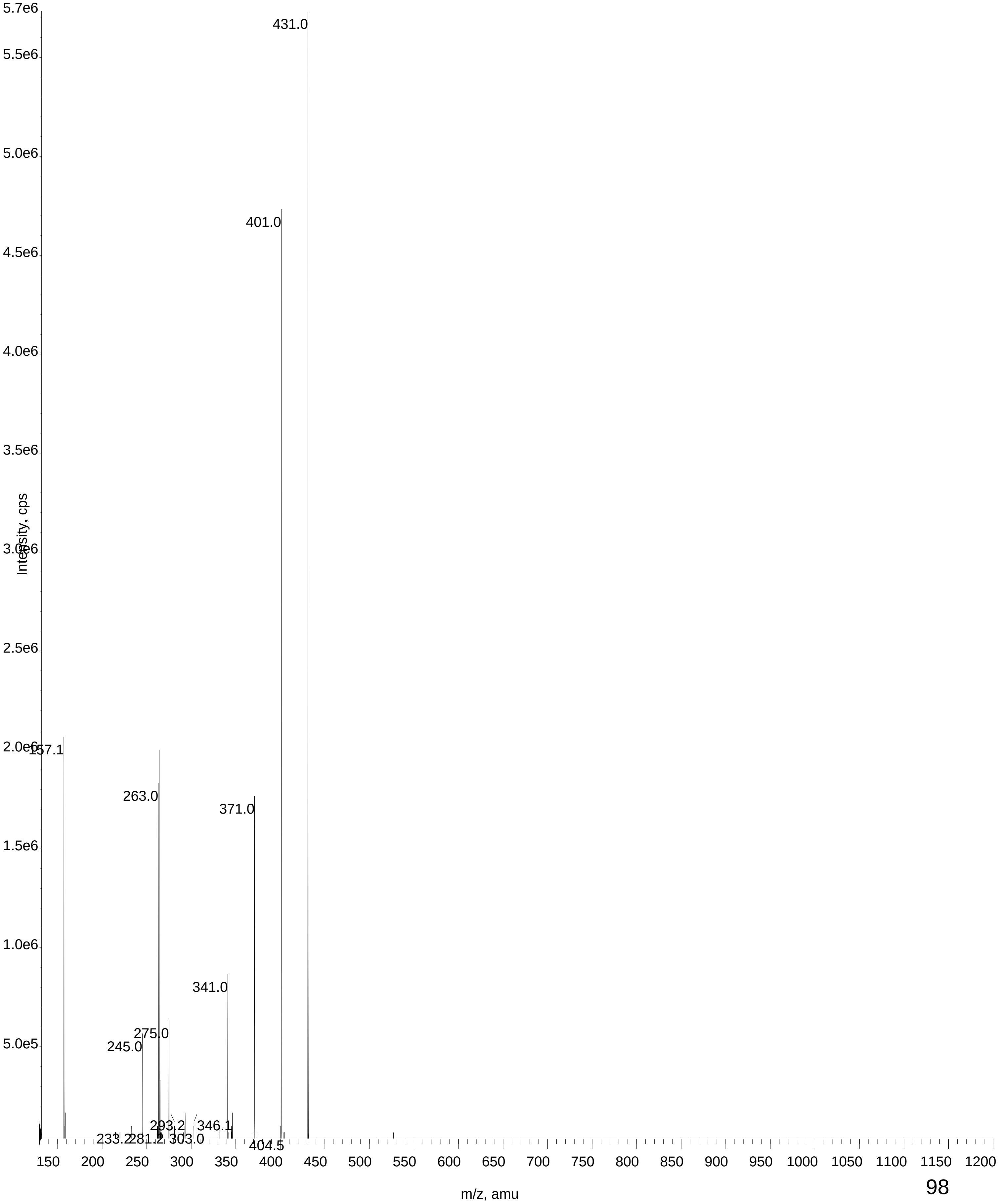

+EPI (487.10) CE (25): 50 MCA scans from Sample 5 (TuneSampleID) of 20.Original_EPI487.wiff (Turbo Spray) Max. 3.2e6 cps.

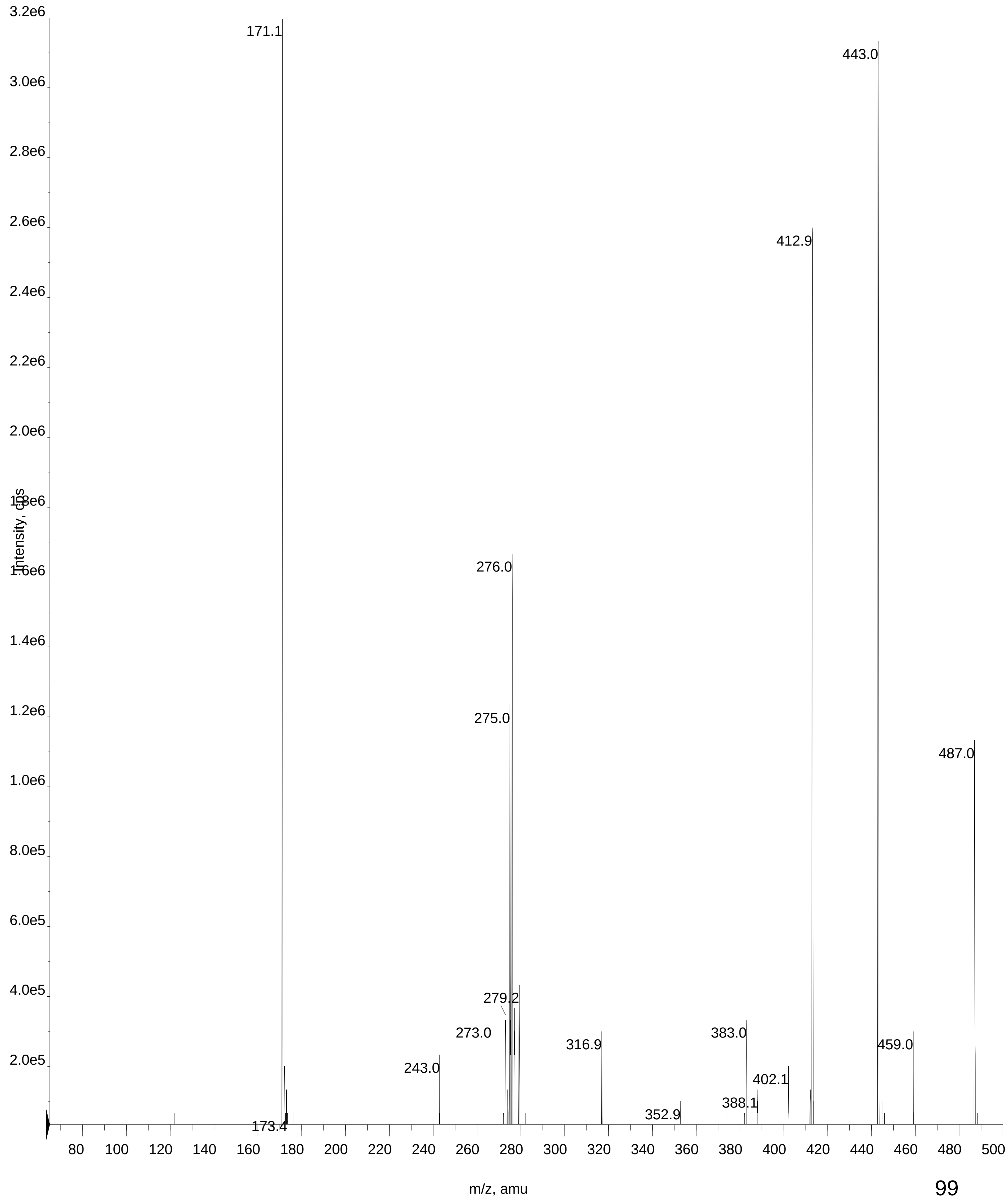

+EPI (489.10) CE (25): 50 MCA scans from Sample 1 (TuneSampleID) of 21.Original_EPI489.wiff (Turbo Spray) Max. 1.1e7 cps.

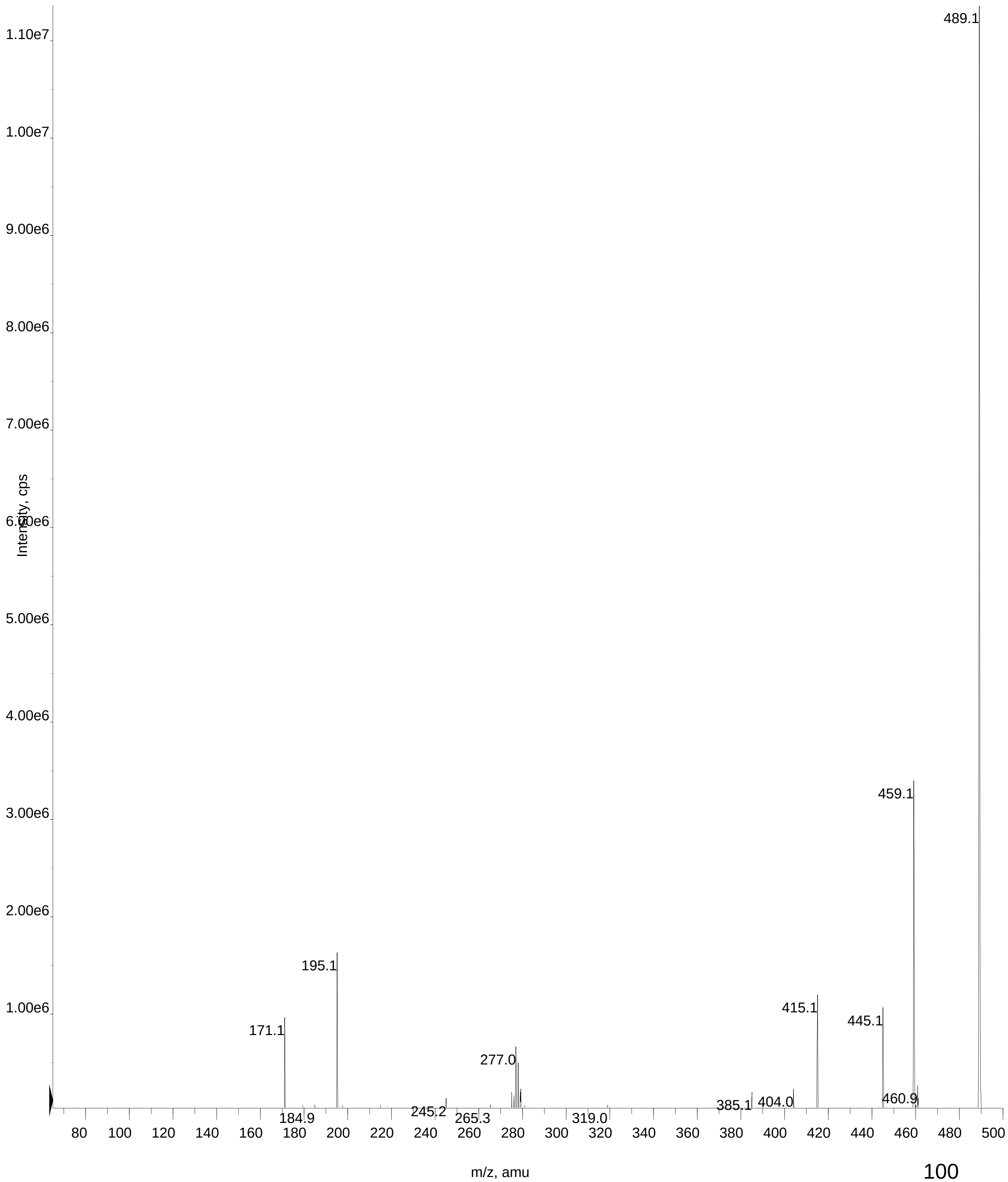

+EPI (457.10) CE (25): 18 MCA scans from Sample 2 (TuneSampleID) of 69.COOH_EPI_457.wiff (Turbo Spray) Max. 2.1e7 cps.

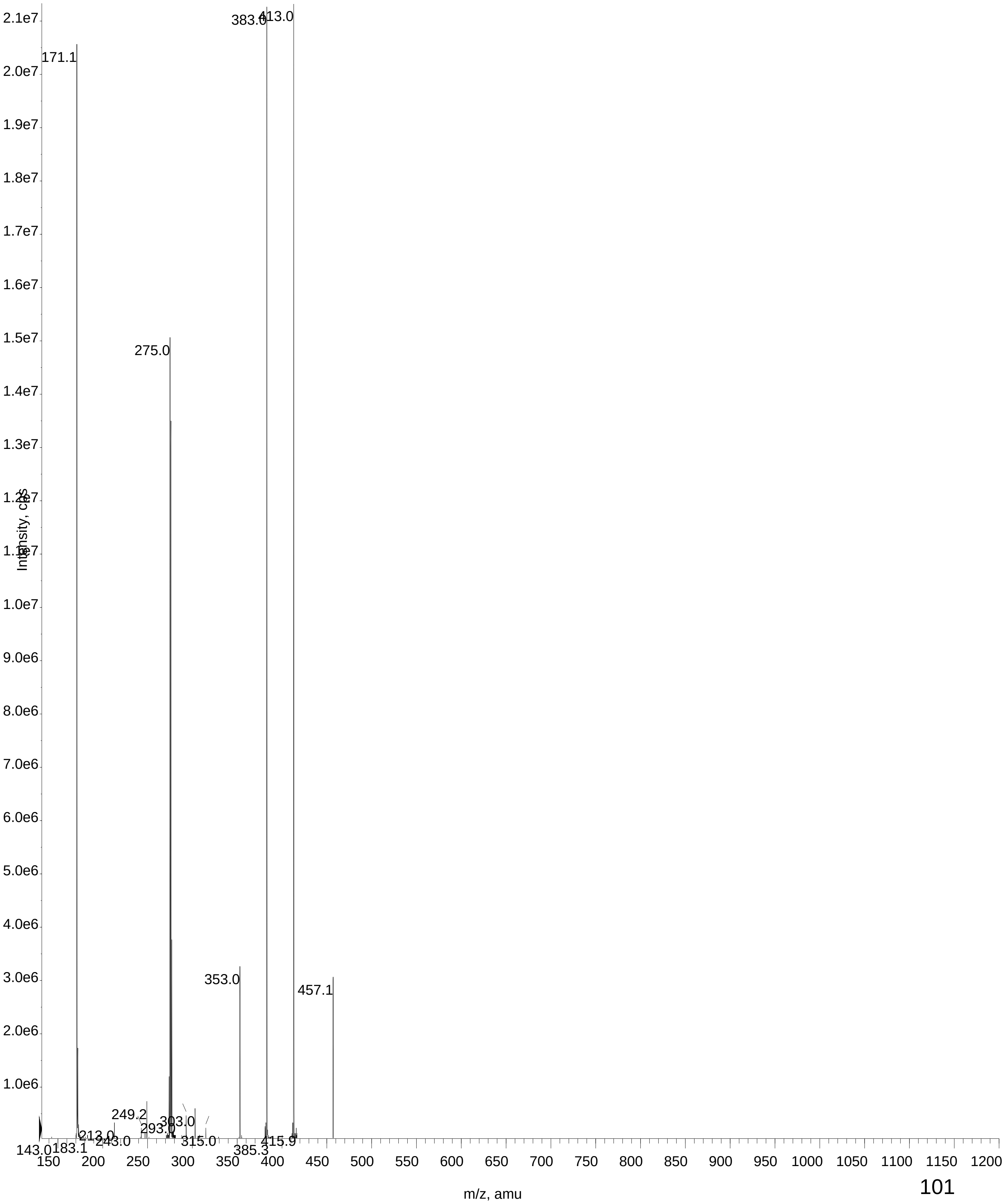

+EPI (459.10) CE (25): 16 MCA scans from Sample 1 (TuneSampleID) of 70.COOH_EPI_459.wiff (Turbo Spray) Max. 1.3e7 cps.

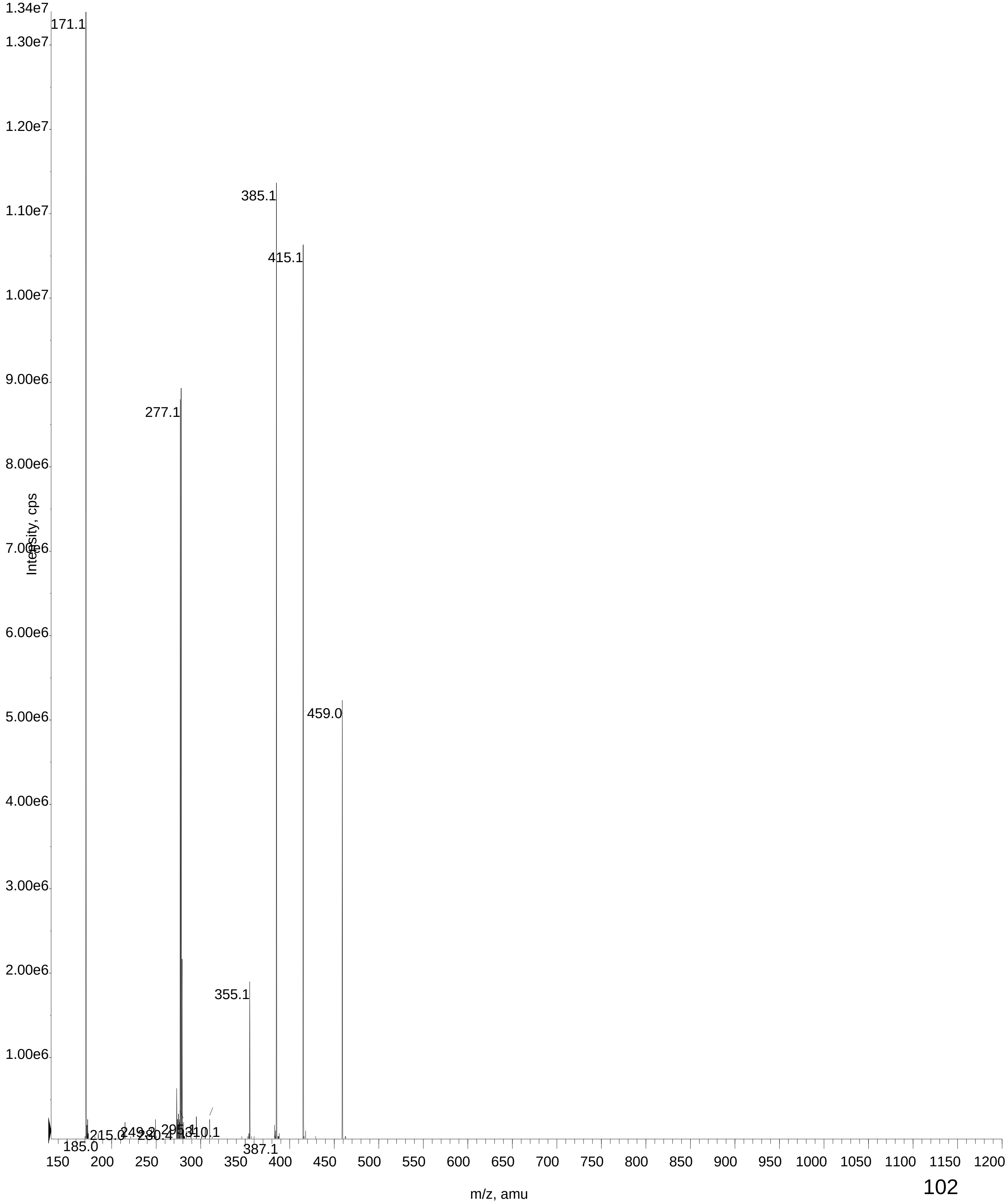

+EPI (487.00) CE (25): 40 MCA scans from Sample 3 of 32.CN_EPI487.wiff (Turbo Spray) Max. 2.8e6 cps.

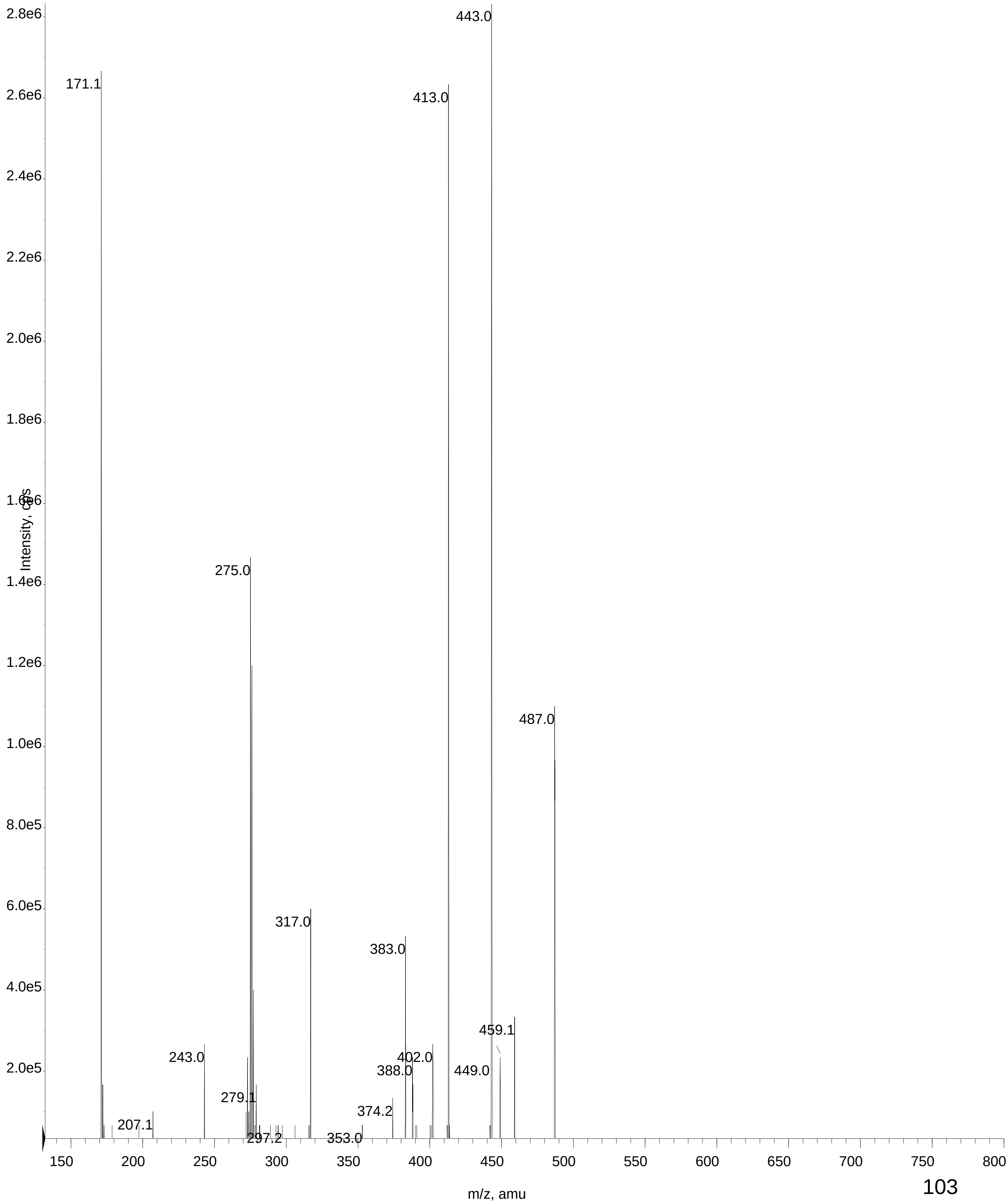

+EPI (489.00) CE (25): 40 MCA scans from Sample 1 of 33.CN_EPI489.wiff (Turbo Spray) Max. 9.7e5 cps.

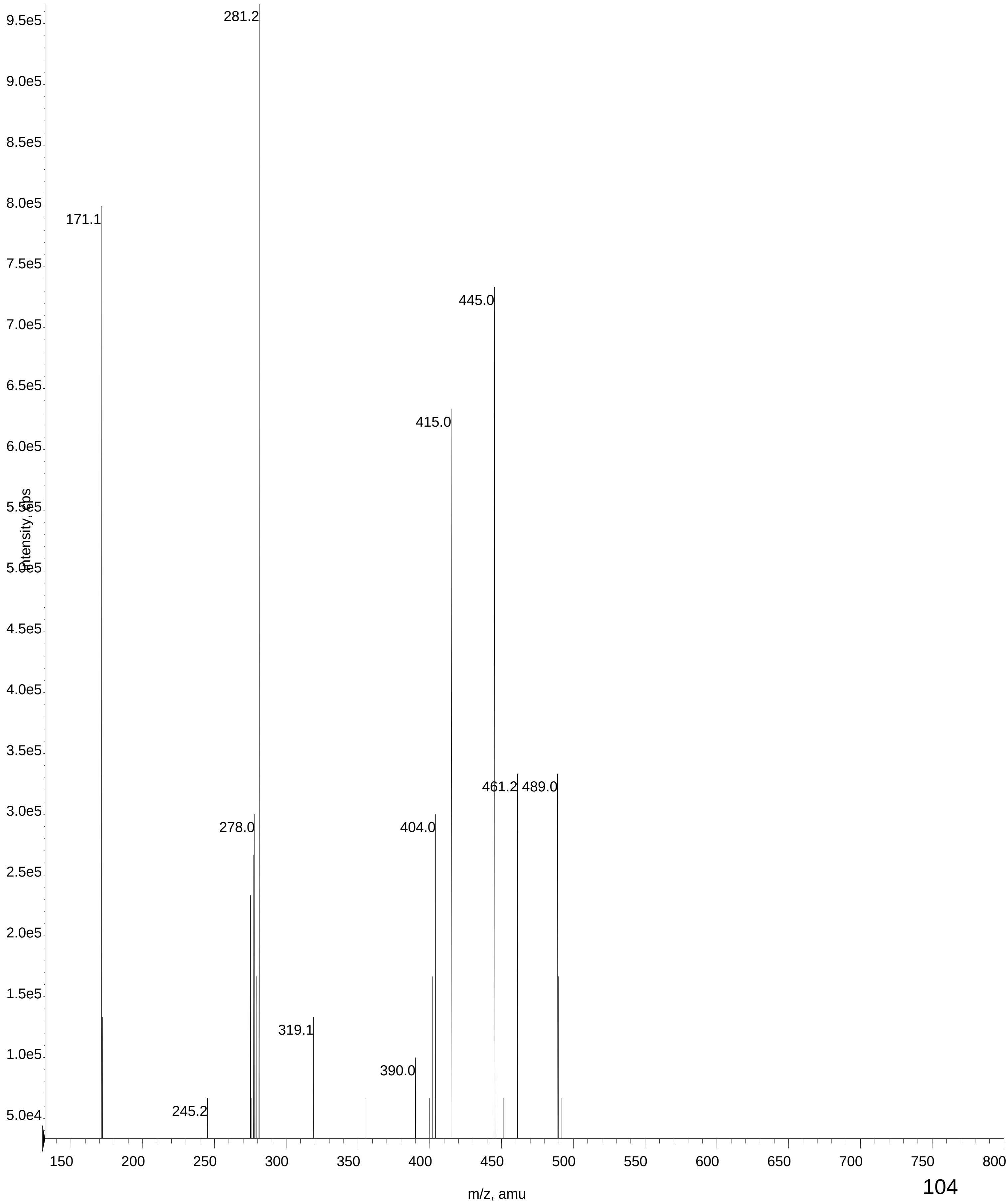

+EPI (473.00) CE (25): 16 MCA scans from Sample 1 (TuneSampleID) of 49.UL_EPI473.wiff (Turbo Spray) Max. 5.1e7 cps.

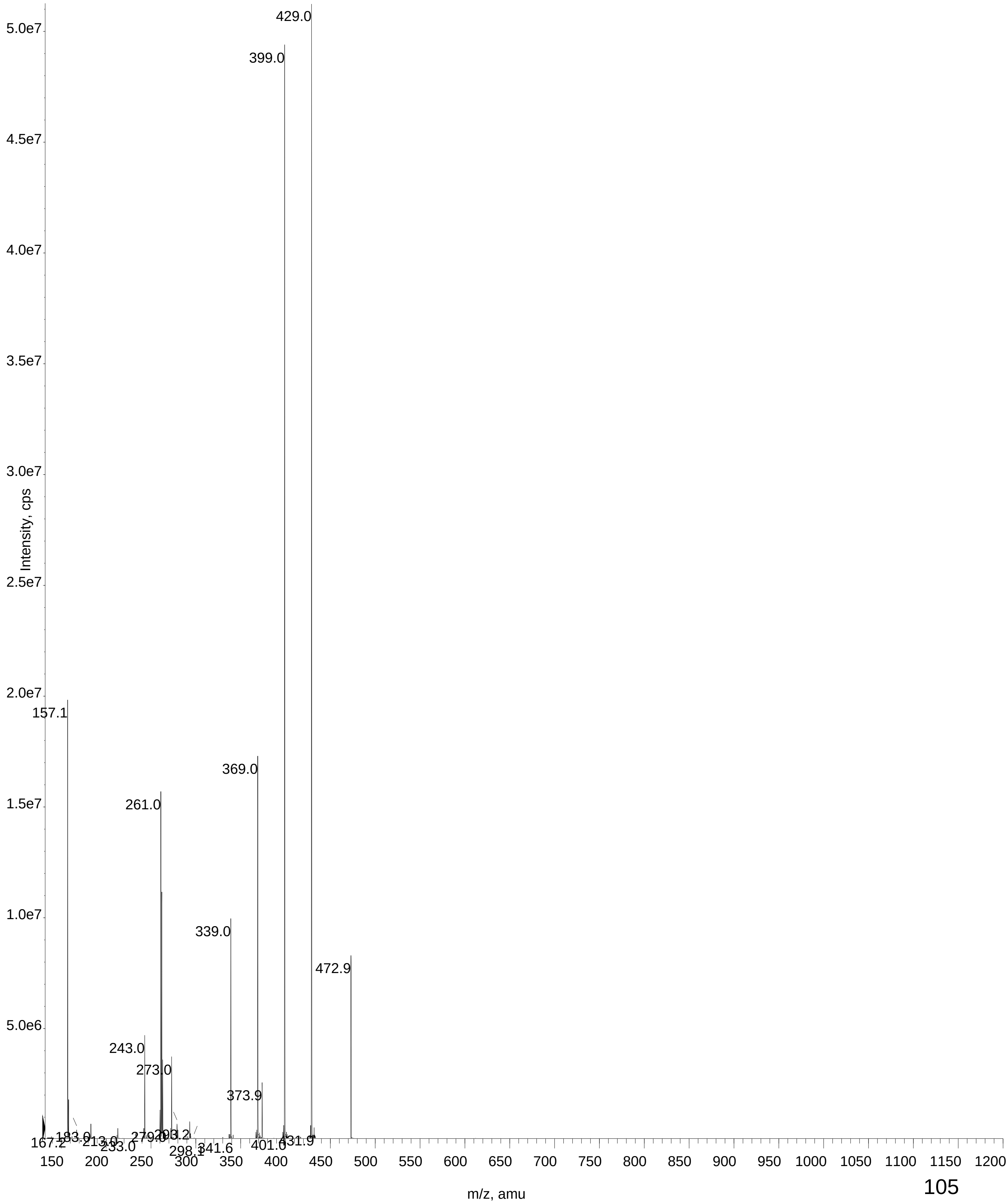

+EPI (475.00) CE (25): 11 MCA scans from Sample 1 (TuneSampleID) of 50.UL_EPI475.wiff (Turbo Spray) Max. 3.3e7 cps.

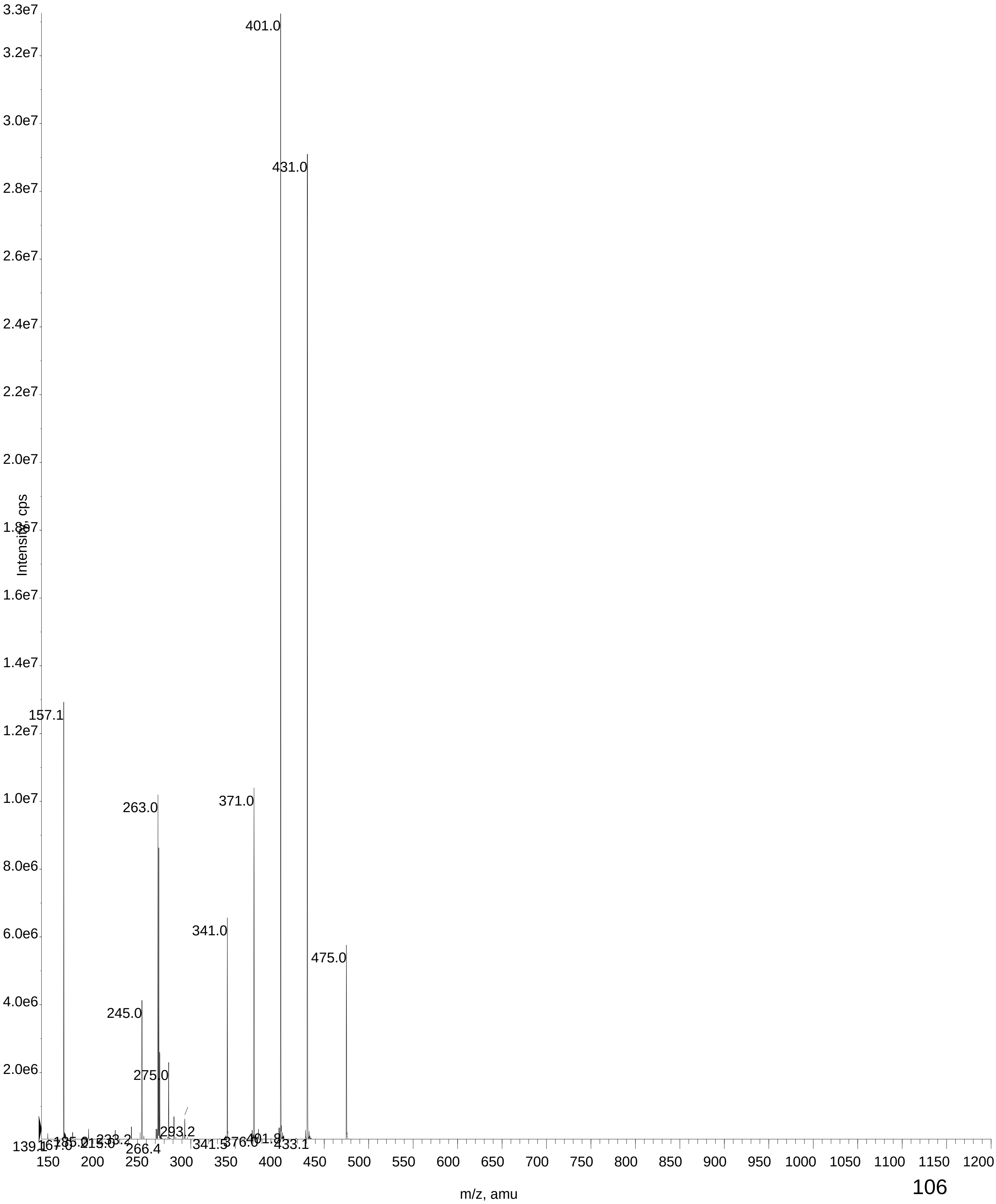

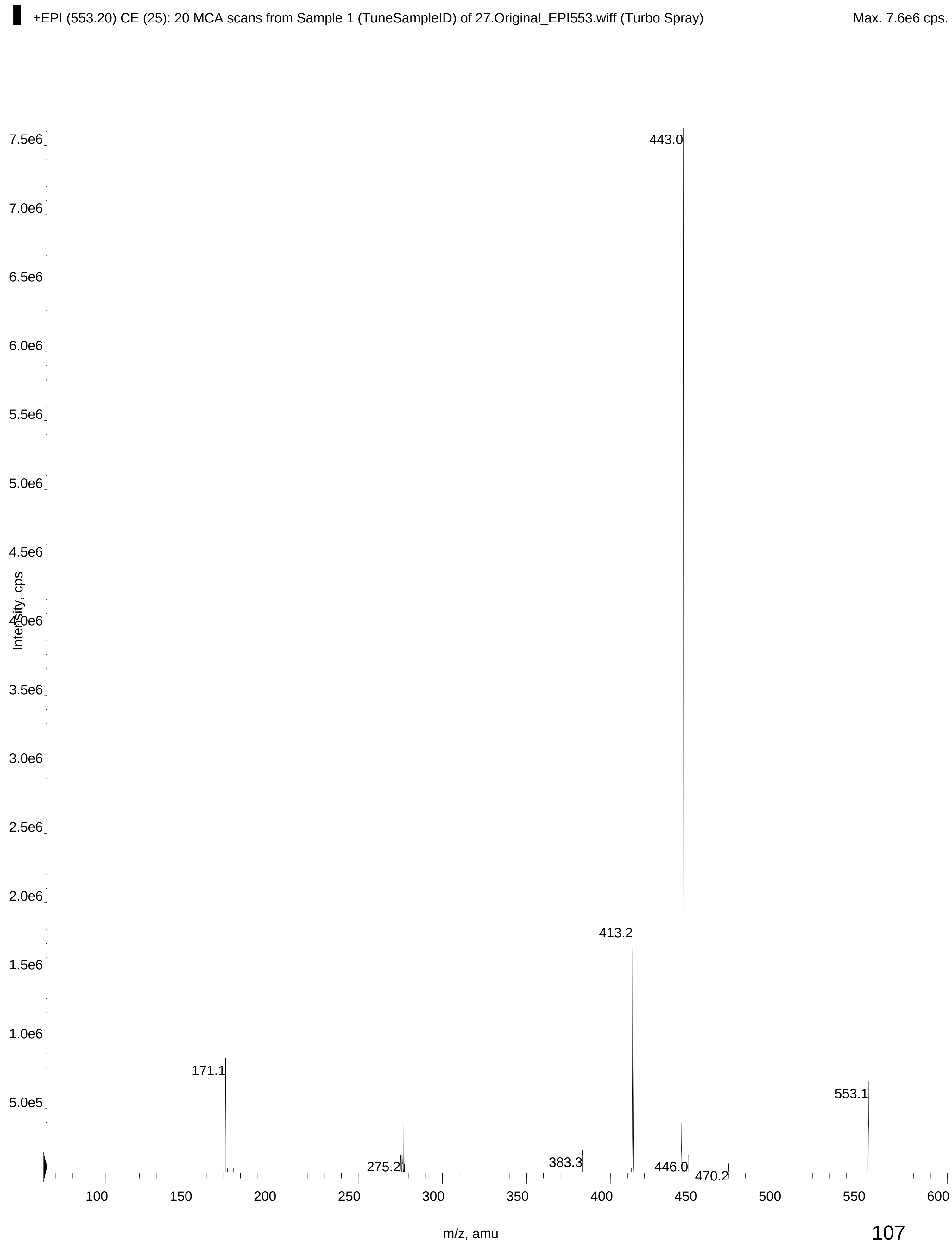
+EPI (553.20) CE (25): 20 MCA scans from Sample 1 (TuneSampleID) of 27.Original_EPI553.wiff (Turbo Spray)
Max. 7.6e6 cps.
Intensity, cps
m/z, amu
443.0
413.2
171.1
553.1
275.2
383.3
446.0
470.2

+EPI (555.20) CE (25): 20 MCA scans from Sample 1 (TuneSampleID) of 28.Original_EPI555.wiff (Turbo Spray) Max. 7.1e6 cps.

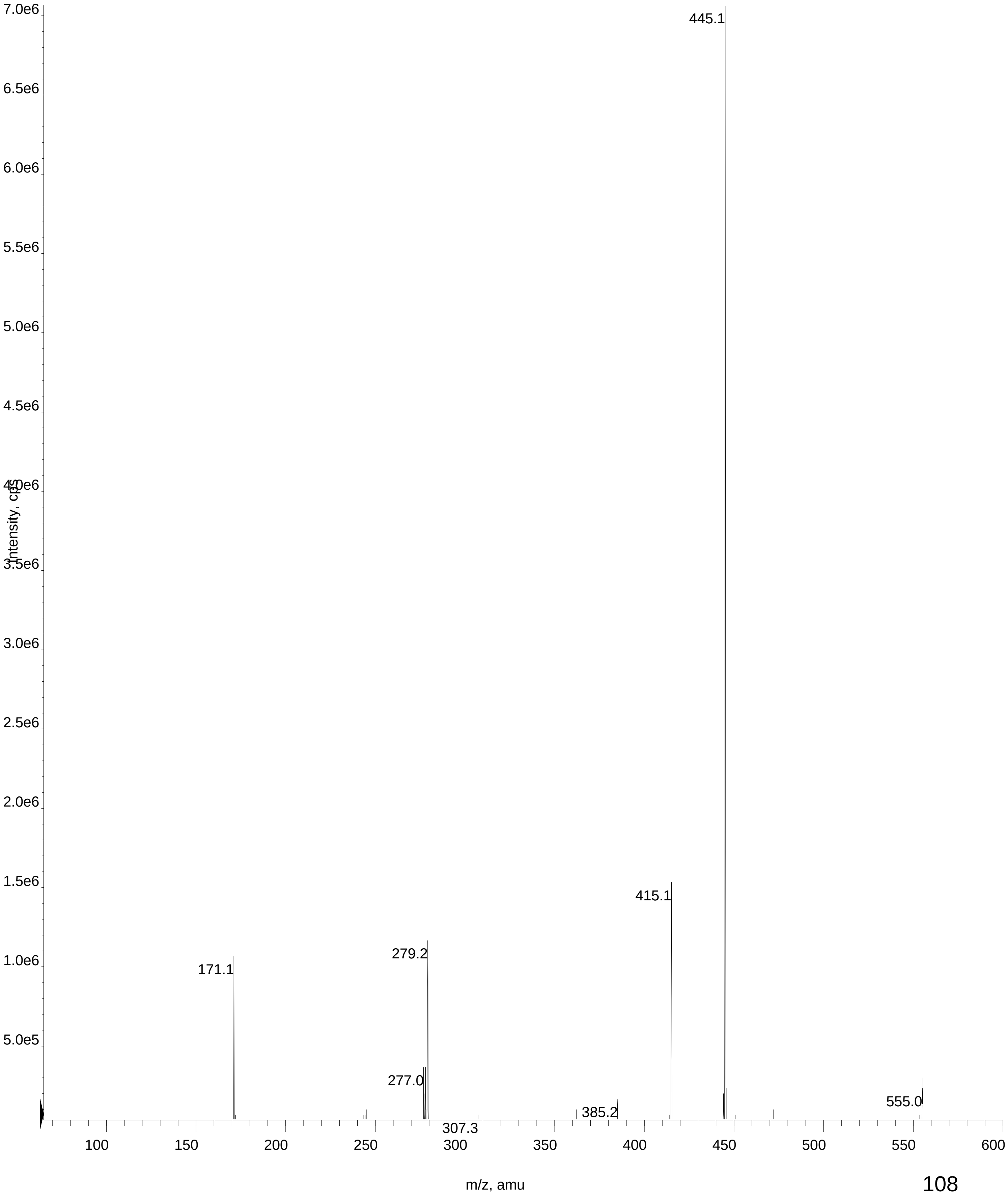

+EPI (523.10) CE (25): 20 MCA scans from Sample 1 (TuneSampleID) of 75.COOH_EPI_523.wiff (Turbo Spray) Max. 1.0e6 cps.

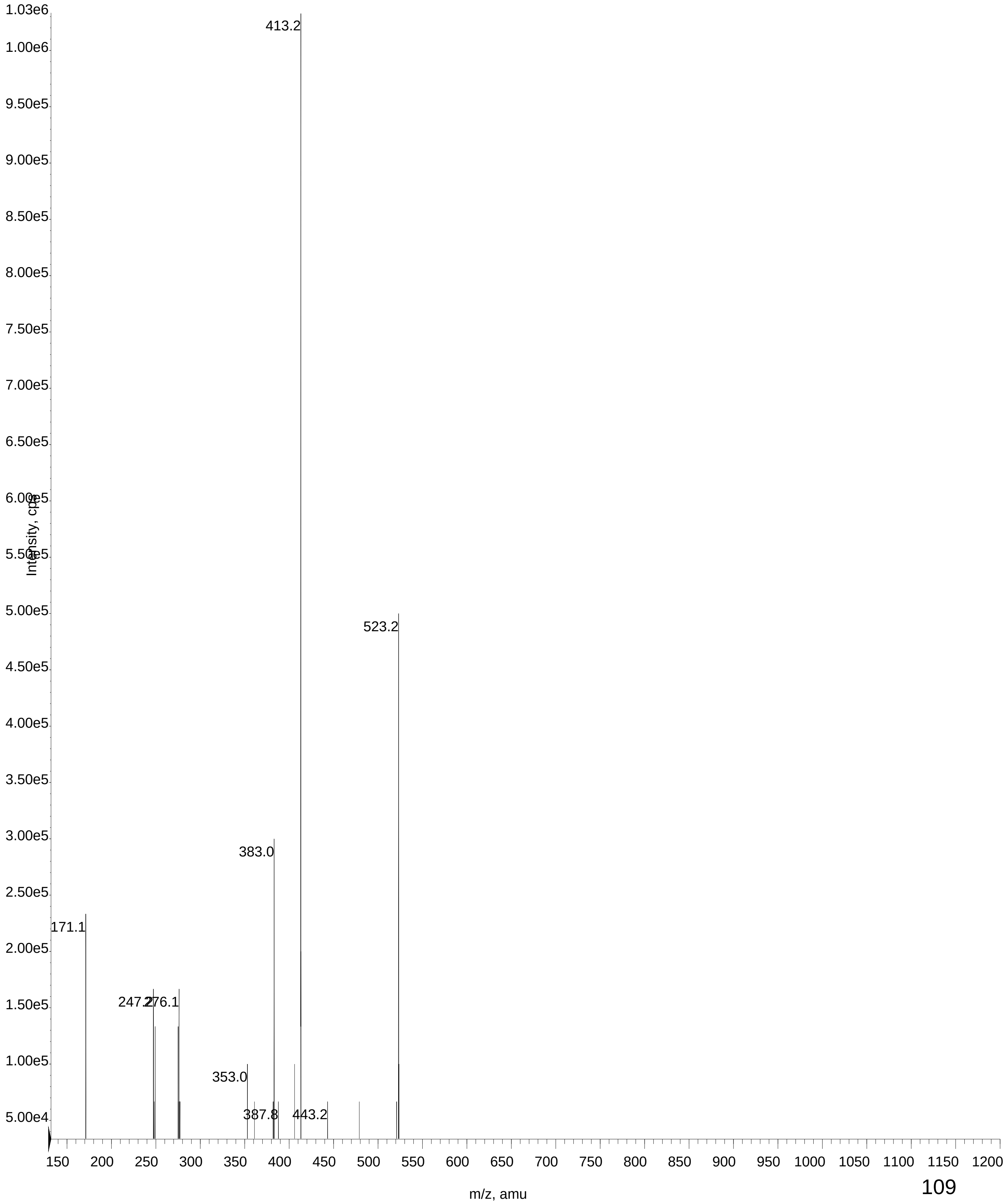

+EPI (525.10) CE (25): 20 MCA scans from Sample 1 (TuneSampleID) of 76.COOH_EPI_525.wiff (Turbo Spray) Max. 1.4e6 cps.

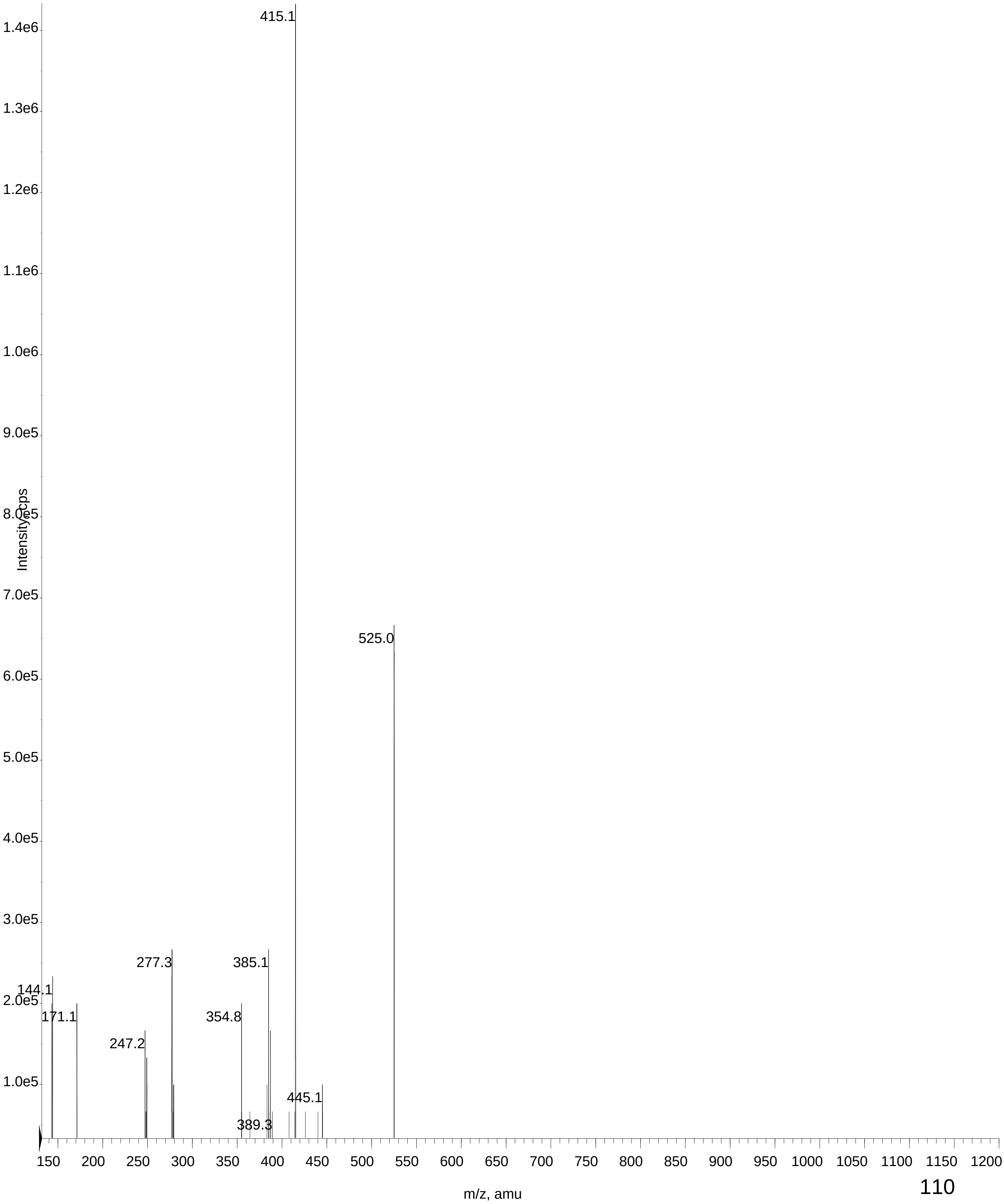

+EPI (555.10) CE (25): 20 MCA scans from Sample 1 of 22.CN_EPI555.wiff (Turbo Spray) Max. 2.8e6 cps.

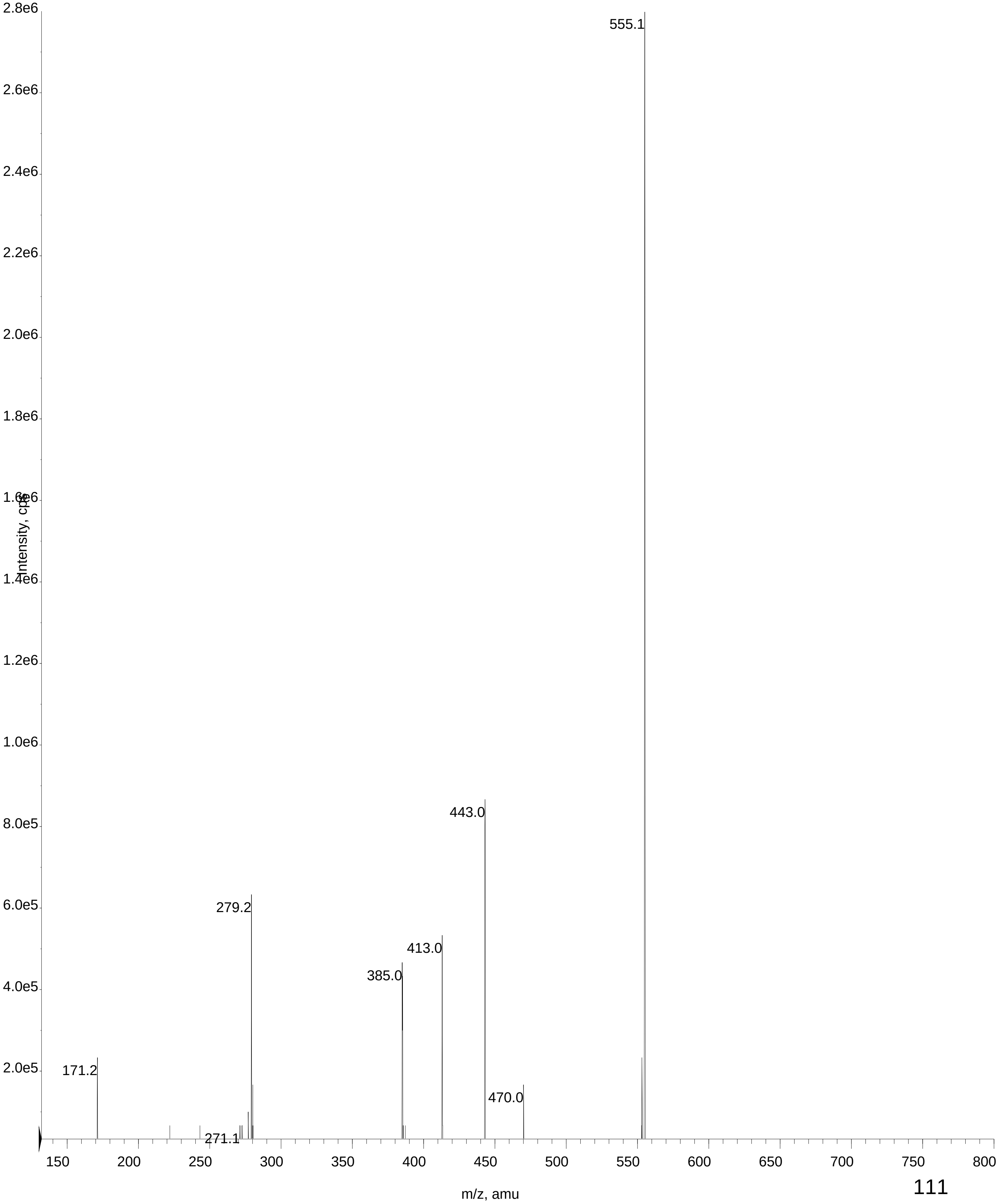

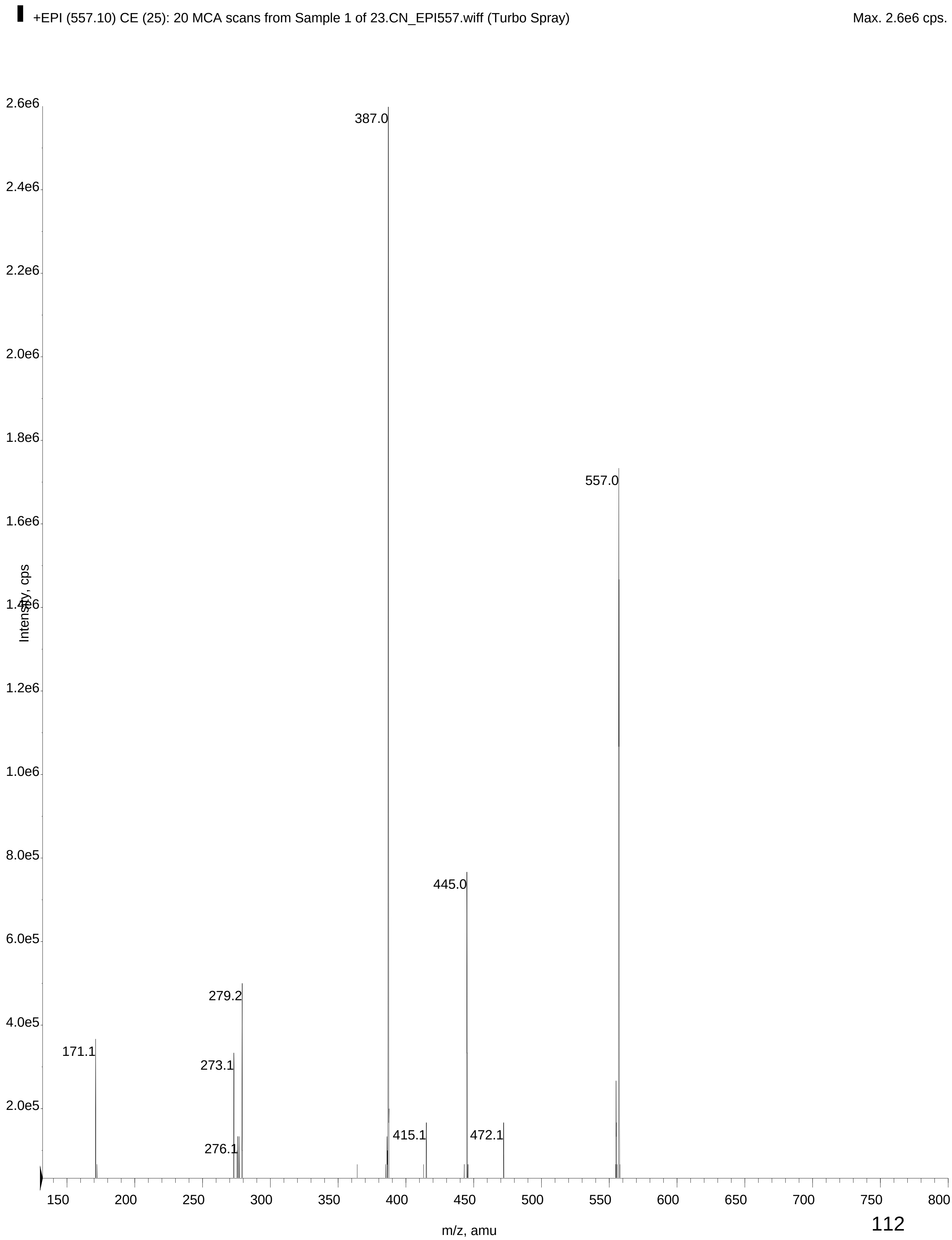
+EPI (557.10) CE (25): 20 MCA scans from Sample 1 of 23.CN_EPI557.wiff (Turbo Spray)
Max. 2.6e6 cps.
Intensity, cps
m/z, amu
387.0
557.0
445.0
279.2
171.1
273.1
276.1
415.1
472.1
2.6e6
2.4e6
2.2e6
2.0e6
1.8e6
1.6e6
1.4e6
1.2e6
1.0e6
8.0e5
6.0e5
4.0e5
2.0e5
150
200
250
300
350
400
450
500
550
600
650
700
750
800

+EPI (539.00) CE (25): 50 MCA scans from Sample 1 (TuneSampleID) of 71.UL_EPI539.wiff (Turbo Spray)

Max. 9.0e5 cps.

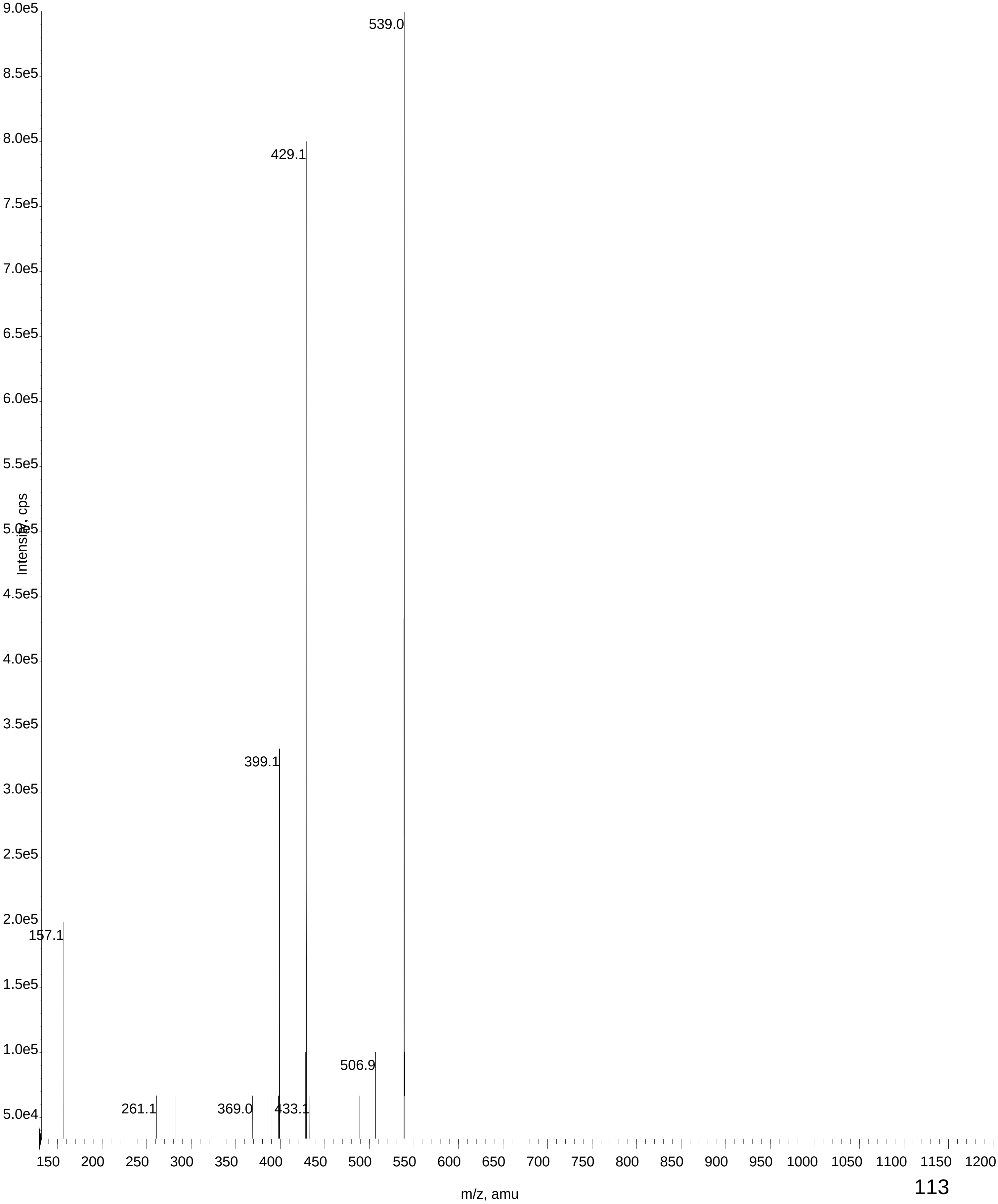

+EPI (541.00) CE (25): 50 MCA scans from Sample 1 (TuneSampleID) of 72.UL_EPI541.wiff (Turbo Spray) Max. 6.7e5 cps.

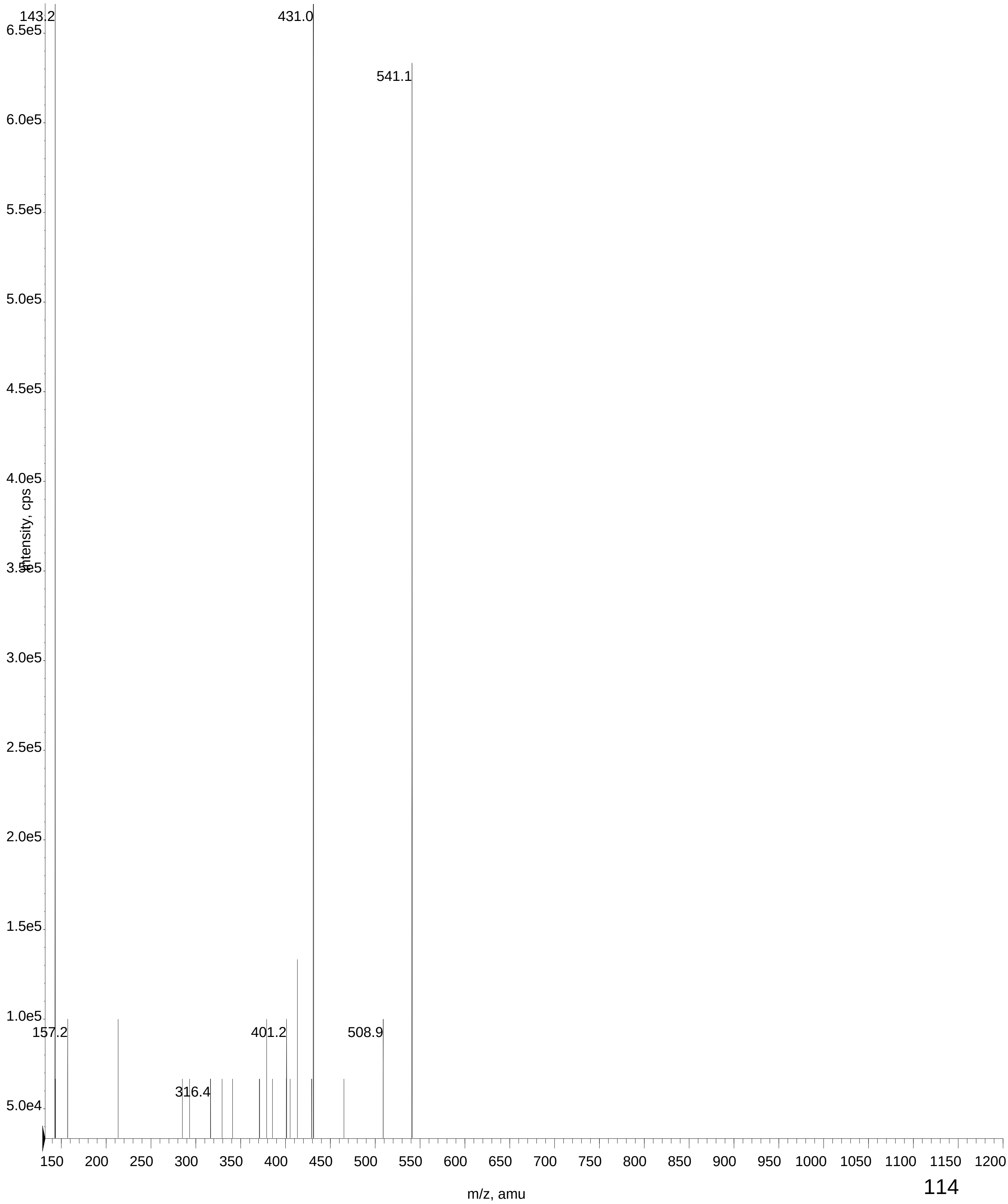

+EPI (667.20) CE (25): 19 MCA scans from Sample 1 (TuneSampleID) of 33.Original_EPI667.wiff (Turbo Spray) Max. 3.0e7 cps.

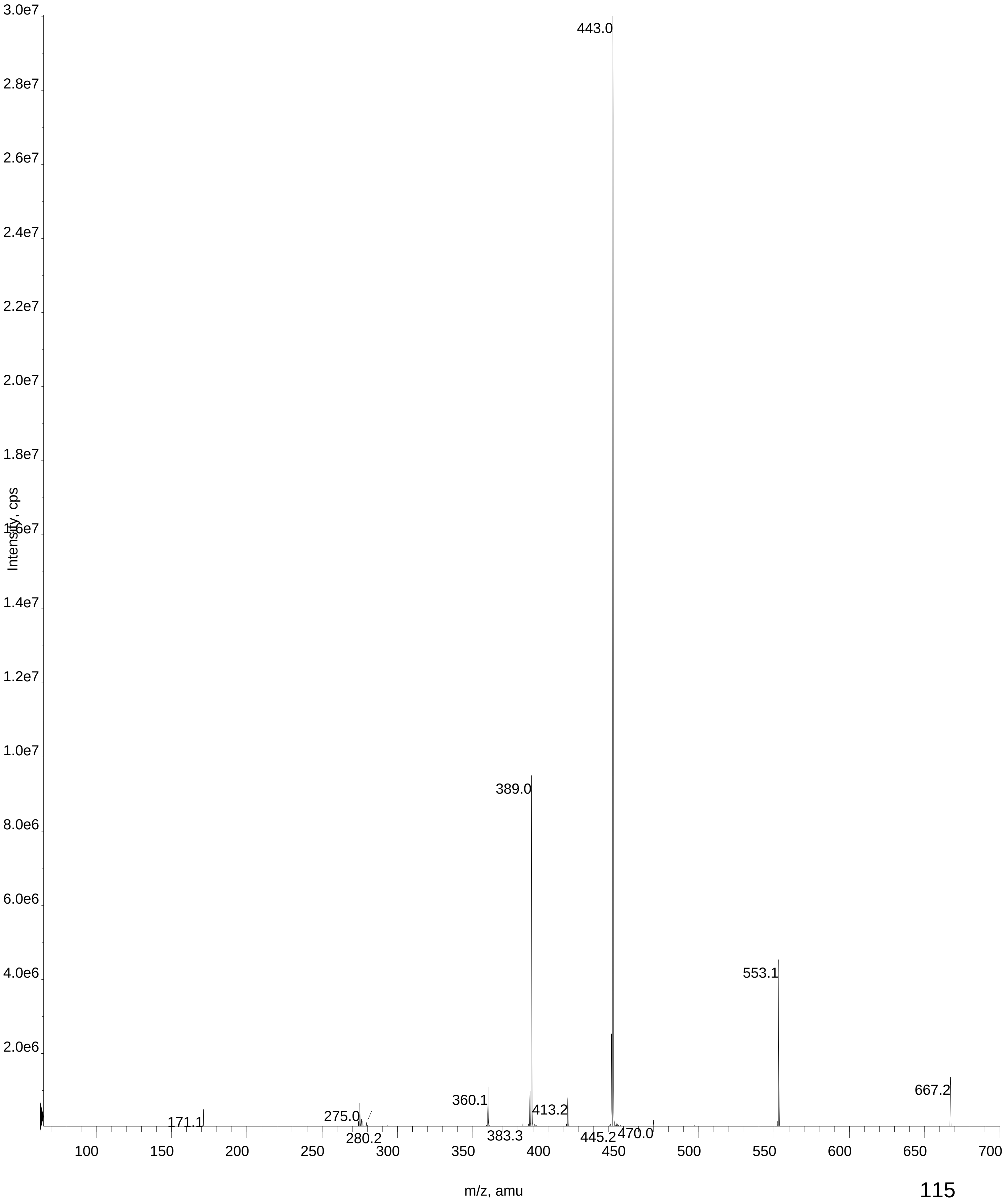

+EPI (669.20) CE (25): 19 MCA scans from Sample 1 (TuneSampleID) of 34.Original_EPI669.wiff (Turbo Spray) Max. 2.8e7 cps.

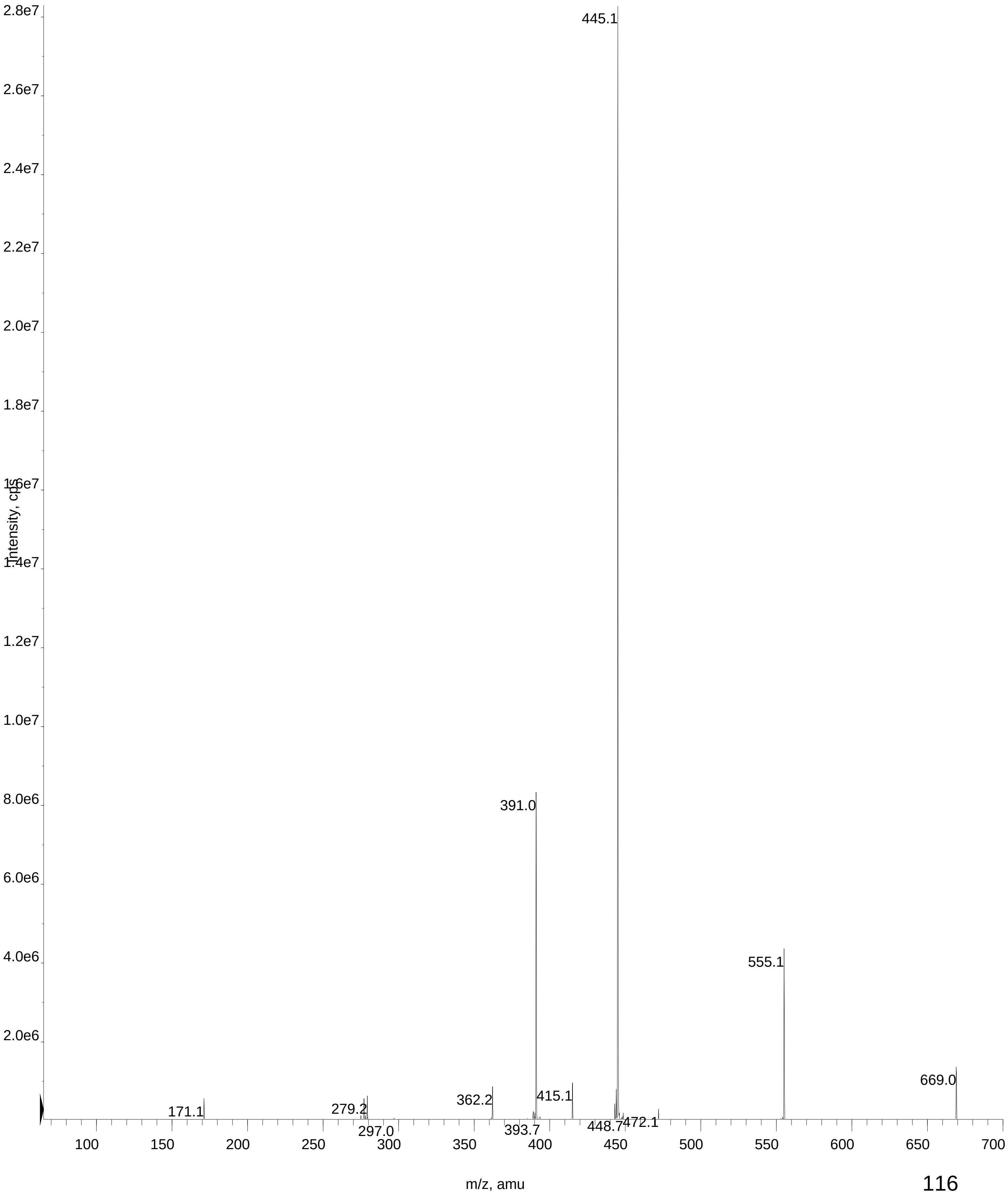

+EPI (637.10) CE (25): 20 MCA scans from Sample 1 (TuneSampleID) of 77.COOH_EPI_637.wiff (Turbo Spray) Max. 5.3e6 cps.

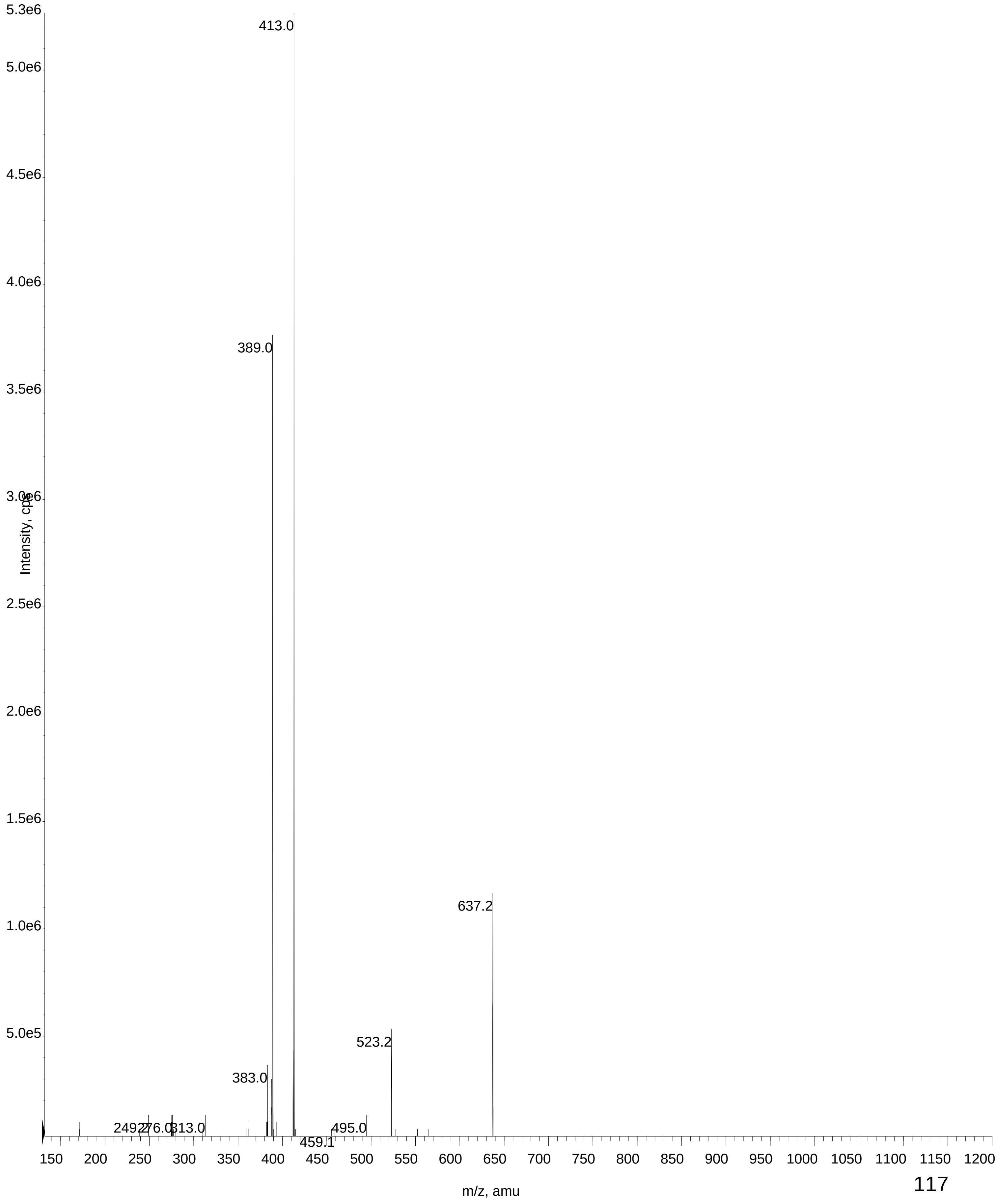

+EPI (639.10) CE (25): 20 MCA scans from Sample 1 (TuneSampleID) of 78.COOH_EPI_638.wiff (Turbo Spray) Max. 3.6e6 cps.

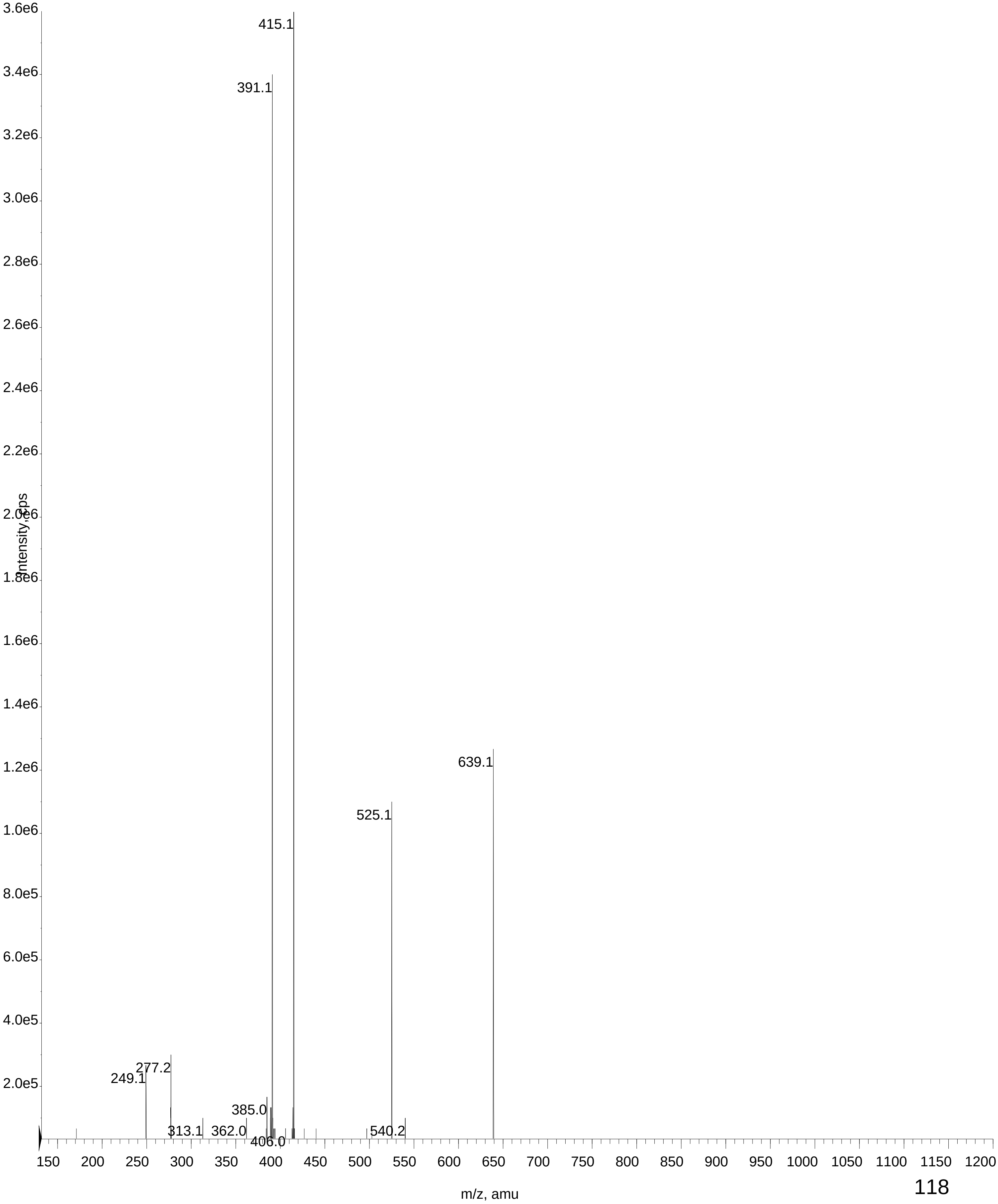

+EPI (669.00) CE (25): 18 MCA scans from Sample 1 of 36.CN_EPI669.wiff (Turbo Spray)

Max. 3.9e6 cps.

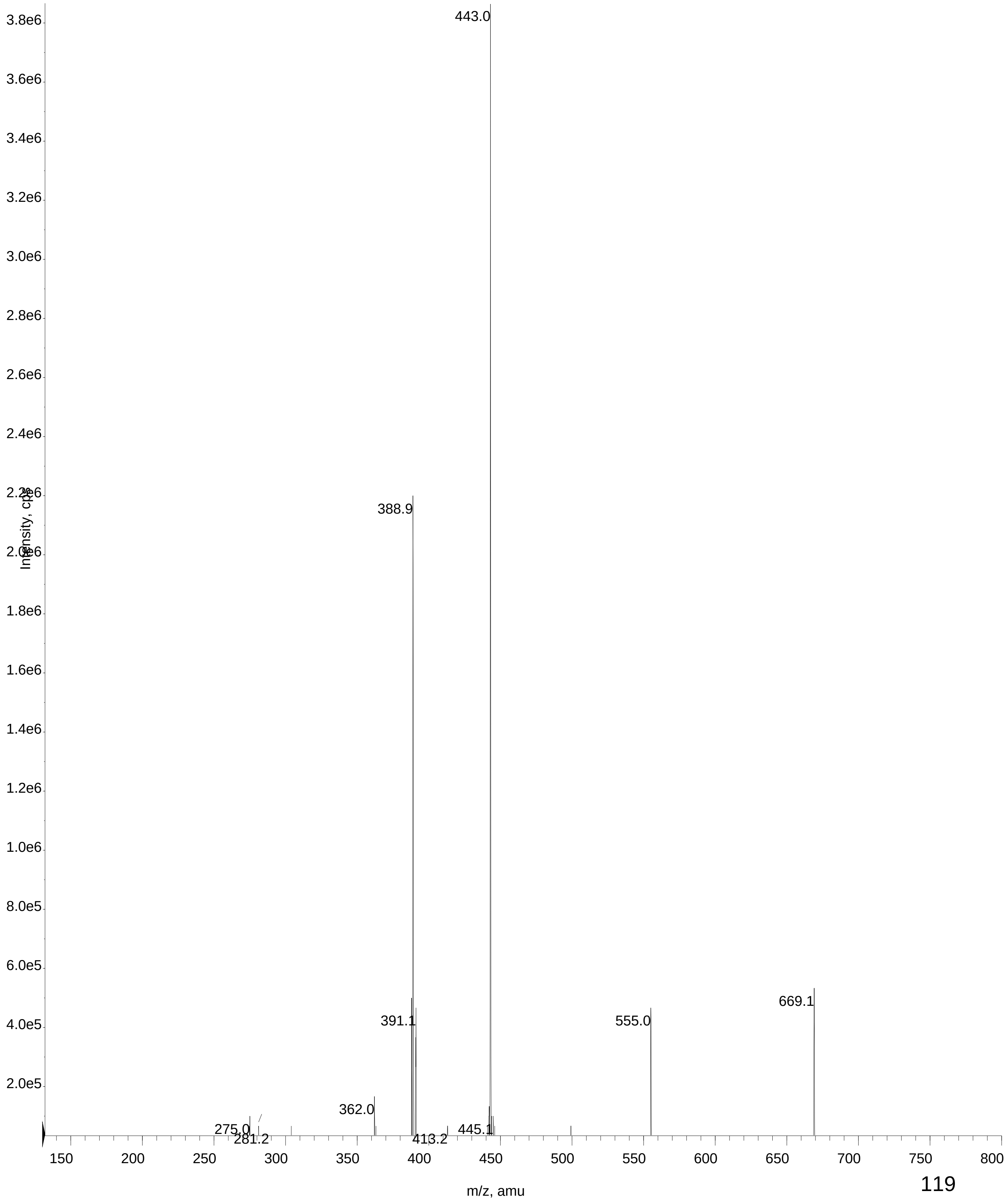

+EPI (671.00) CE (25): 27 MCA scans from Sample 1 of 37.CN_EPI671.wiff (Turbo Spray) Max. 4.7e6 cps.

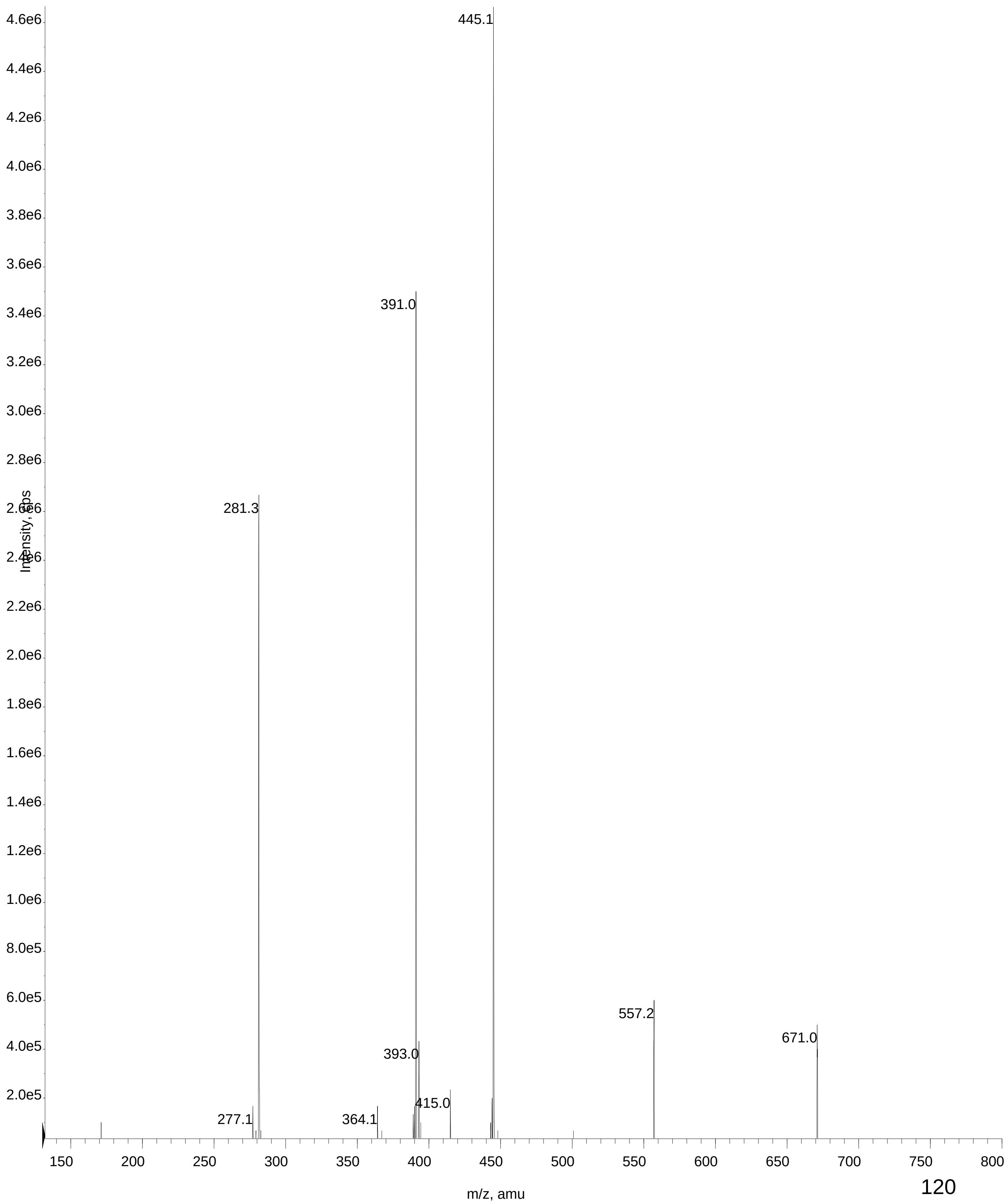

+EPI (653.00) CE (25): 10 MCA scans from Sample 3 (TuneSampleID) of 57.UL_EPI653.wiff (Turbo Spray) Max. 2.8e6 cps.

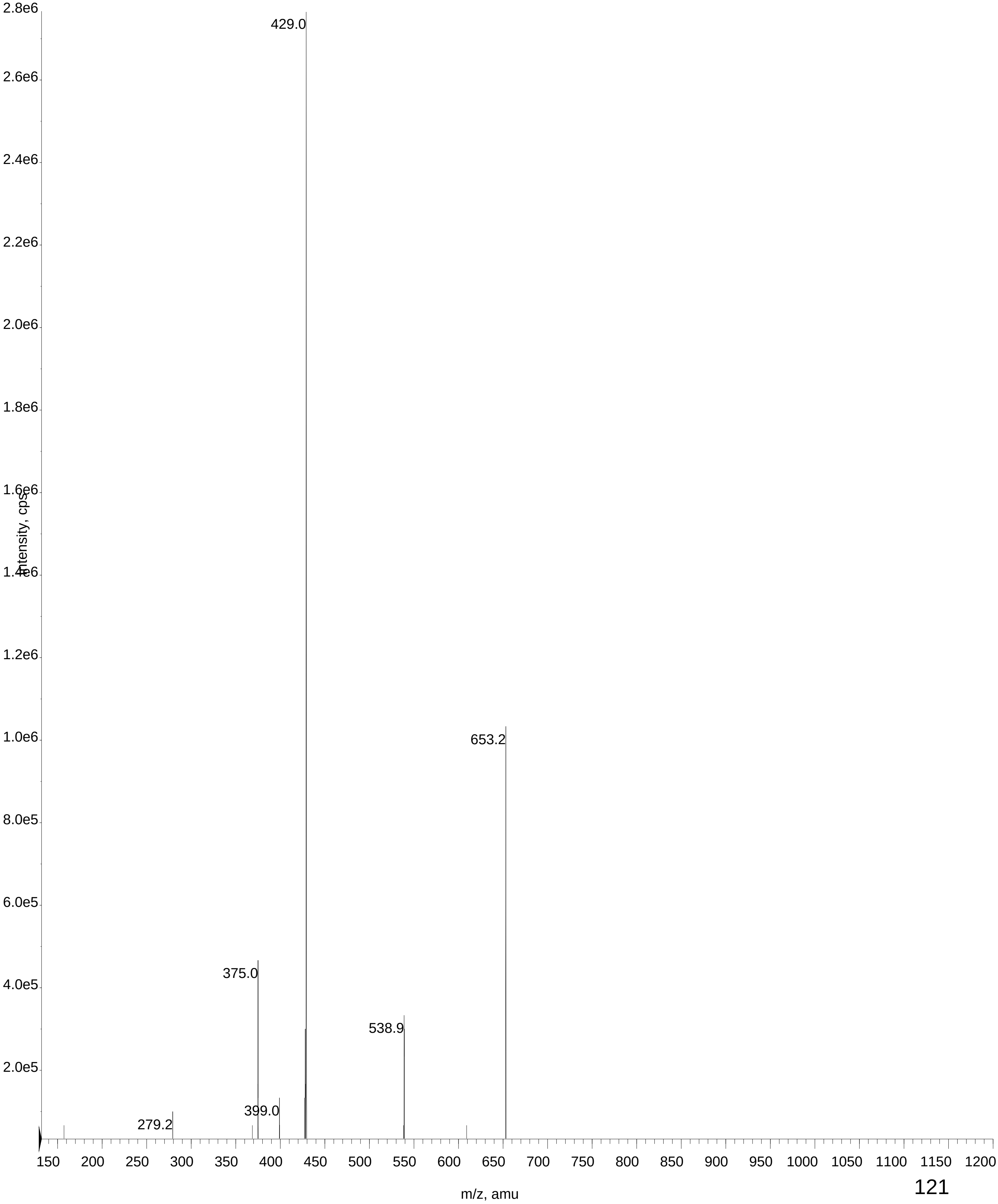

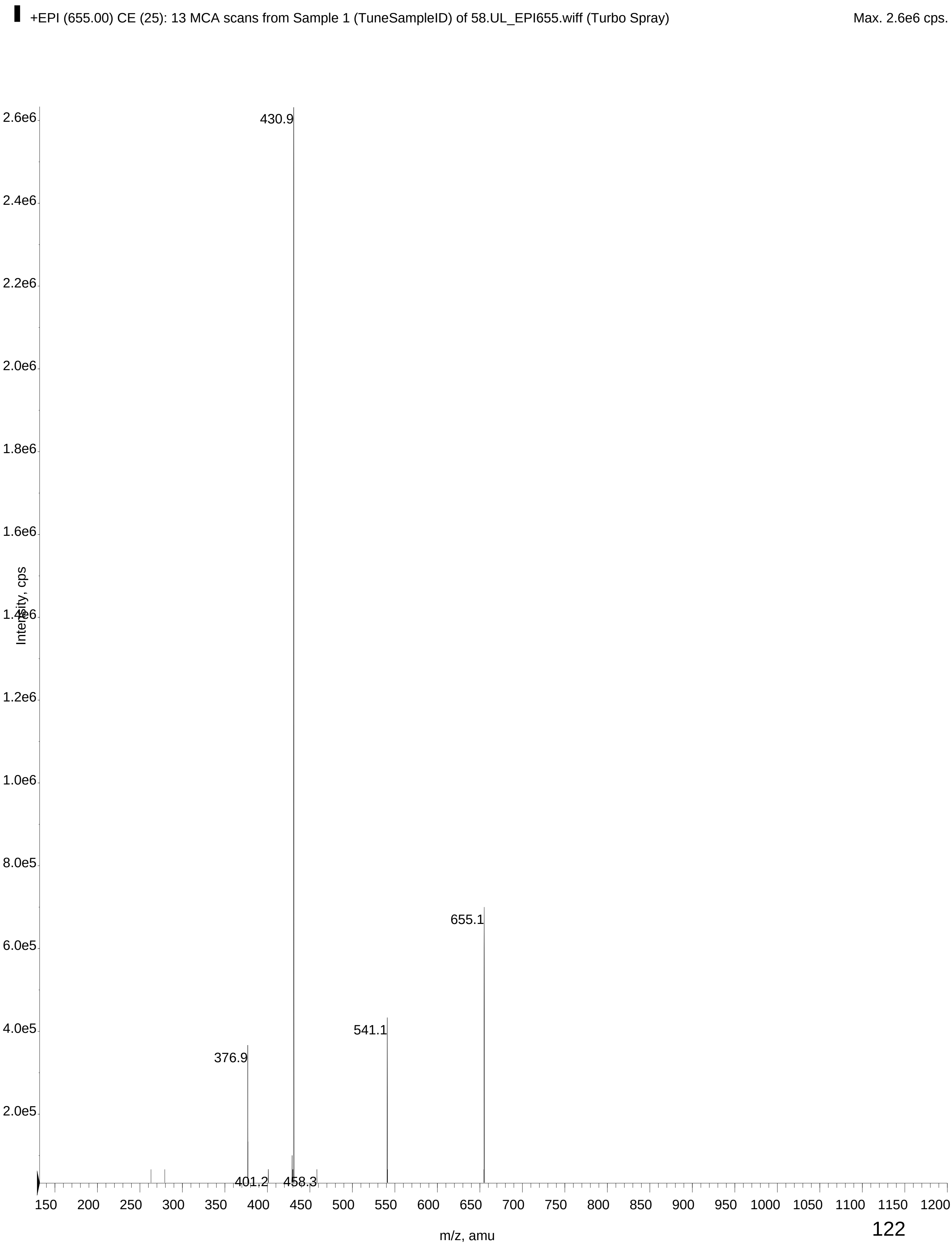
+EPI (655.00) CE (25): 13 MCA scans from Sample 1 (TuneSampleID) of 58.UL_EPI655.wiff (Turbo Spray)
Max. 2.6e6 cps.
Intensity, cps
2.6e6
2.4e6
2.2e6
2.0e6
1.8e6
1.6e6
1.4e6
1.2e6
1.0e6
8.0e5
6.0e5
4.0e5
2.0e5
430.9
655.1
541.1
376.9
401.2
458.3
150 200 250 300 350 400 450 500 550 600 650 700 750 800 850 900 950 1000 1050 1100 1150 1200
m/z, amu

+NL (44.00): 20 MCA scans from Sample 1 (TuneSampleID) of 5.Original_NL44_300-800.wiff (Turbo Spray) Max. 1.7e4 cps.

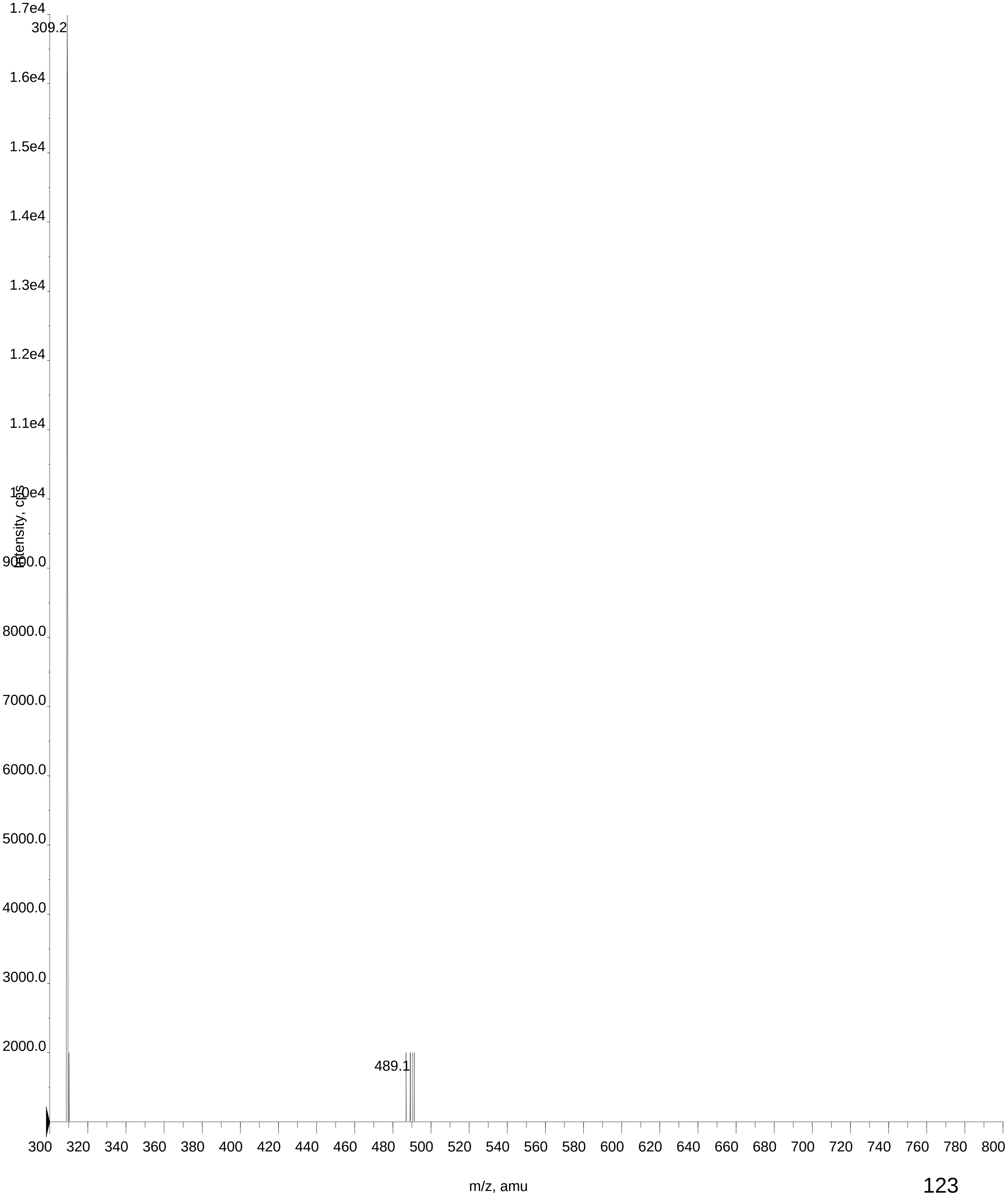

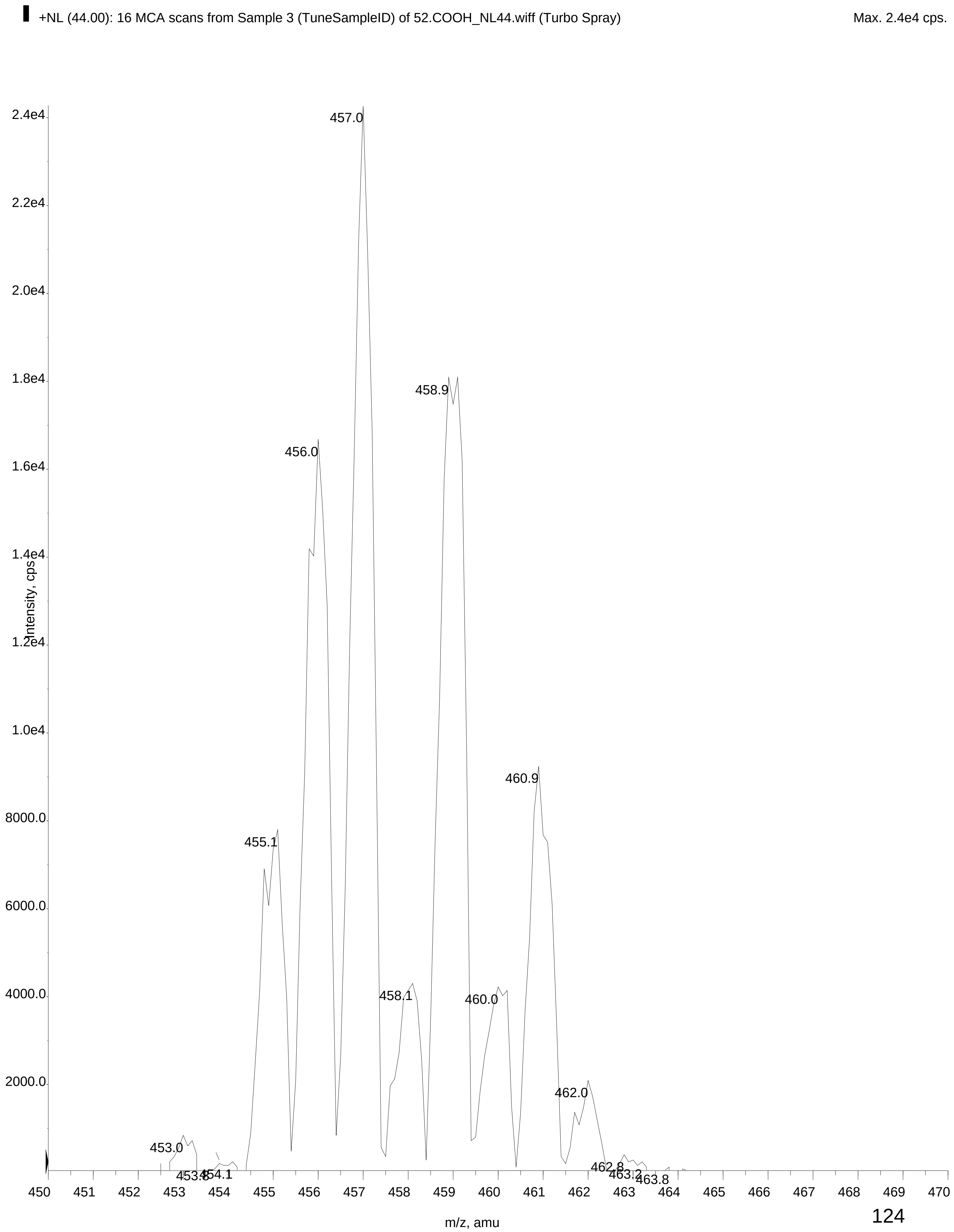
+NL (44.00): 16 MCA scans from Sample 3 (TuneSampleID) of 52.COOH_NL44.wiff (Turbo Spray)
Max. 2.4e4 cps.
Intensity, cps
2.4e4
2.2e4
2.0e4
1.8e4
1.6e4
1.4e4
1.2e4
1.0e4
8000.0
6000.0
4000.0
2000.0
457.0
458.9
456.0
460.9
455.1
458.1
460.0
462.0
453.0
454.1
462.8
463.8
450
451
452
453
454
455
456
457
458
459
460
461
462
463
464
465
466
467
468
469
470
m/z, amu

+NL (44.00): 20 MCA scans from Sample 1 (TuneSampleID) of 3.CN_NL44.wiff (Turbo Spray) Max. 2.2e4 cps.

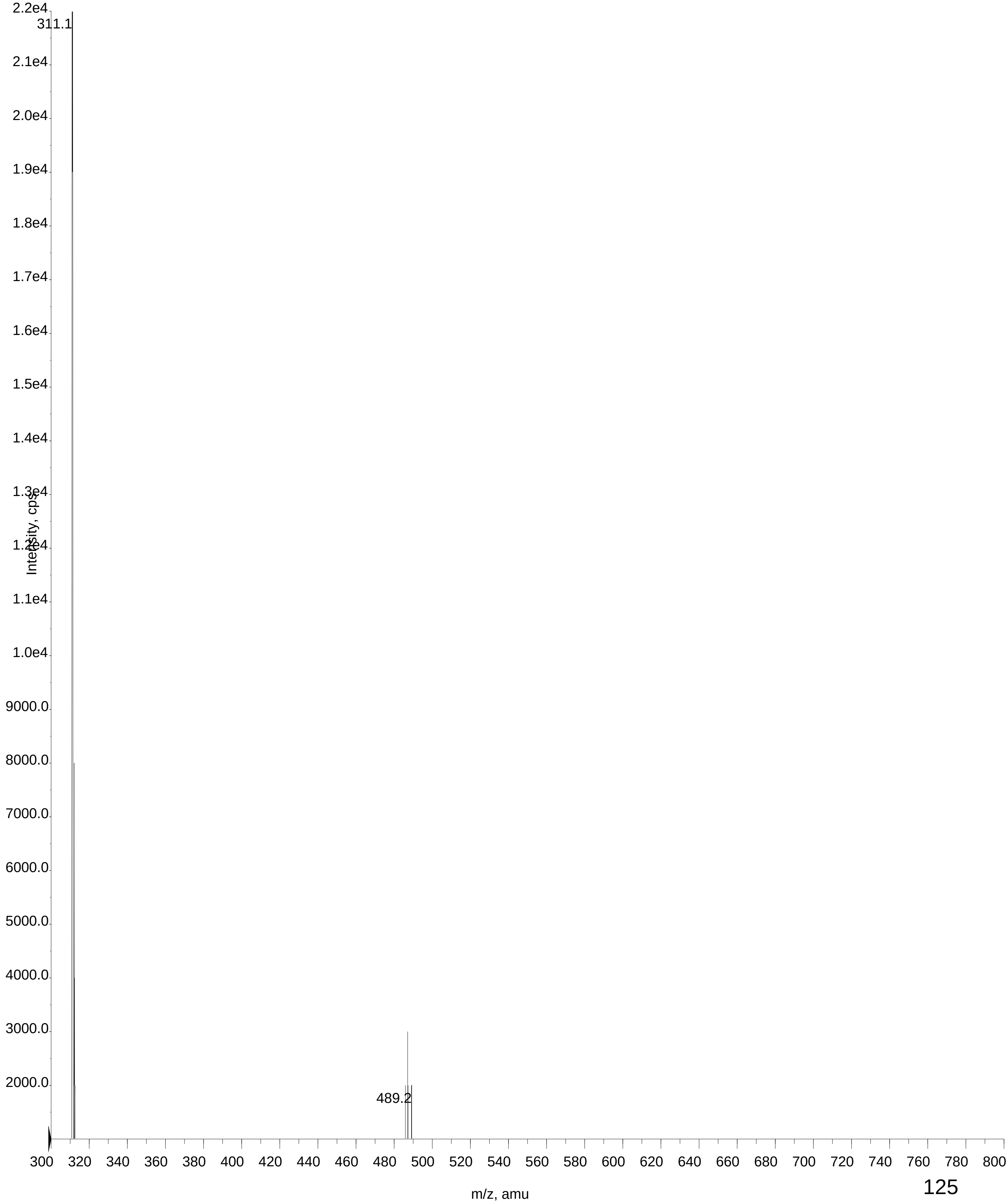

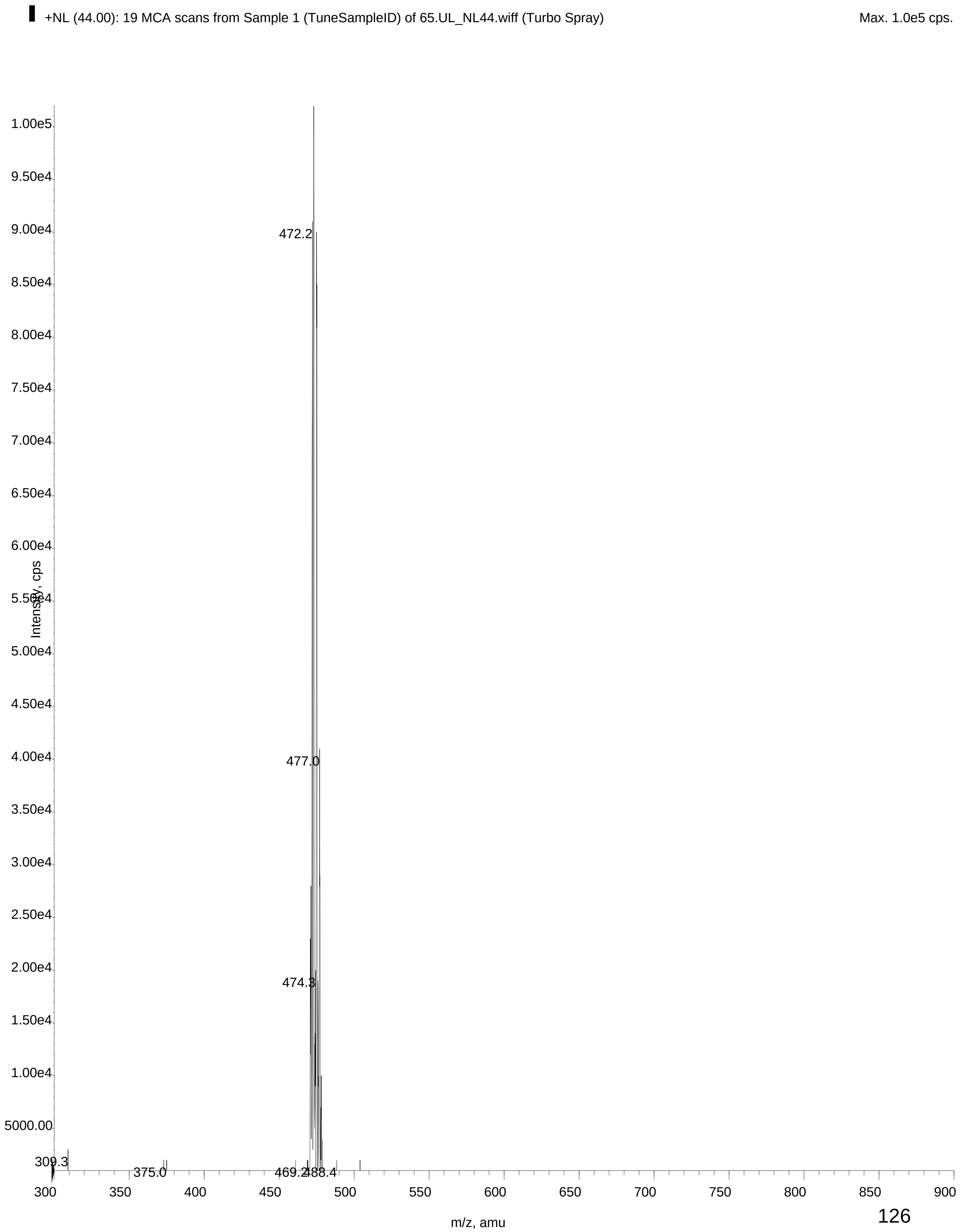
+NL (44.00): 19 MCA scans from Sample 1 (TuneSampleID) of 65.UL_NL44.wiff (Turbo Spray)
Max. 1.0e5 cps.
Intensity, cps
m/z, amu
472.2
477.0
474.3
309.3
375.0
469.2
488.4

+NL (110.00): 20 MCA scans from Sample 1 (TuneSampleID) of 6.Original_NL110_300-800.wiff (Turbo Spray) Max. 1.2e4 cps.

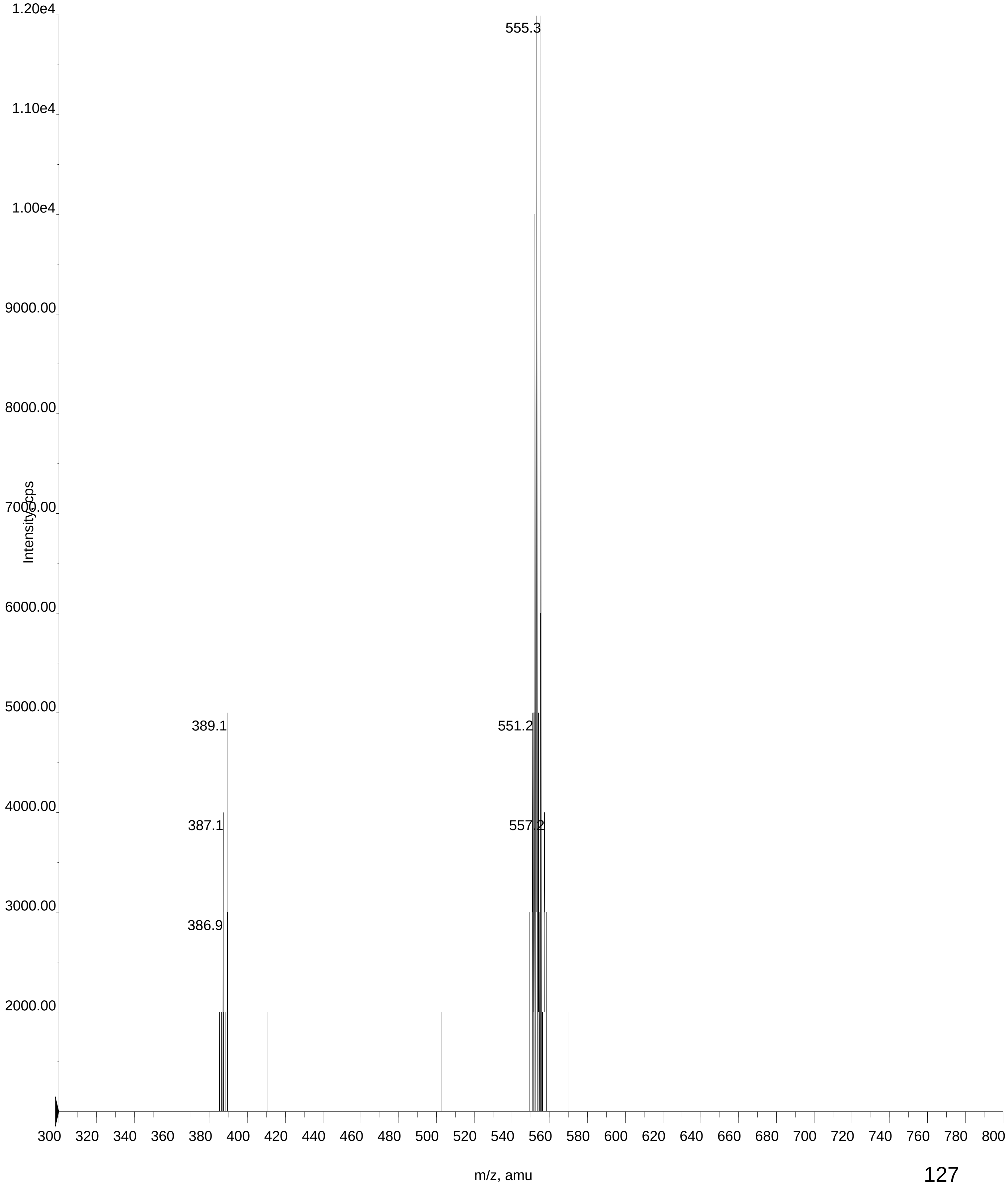

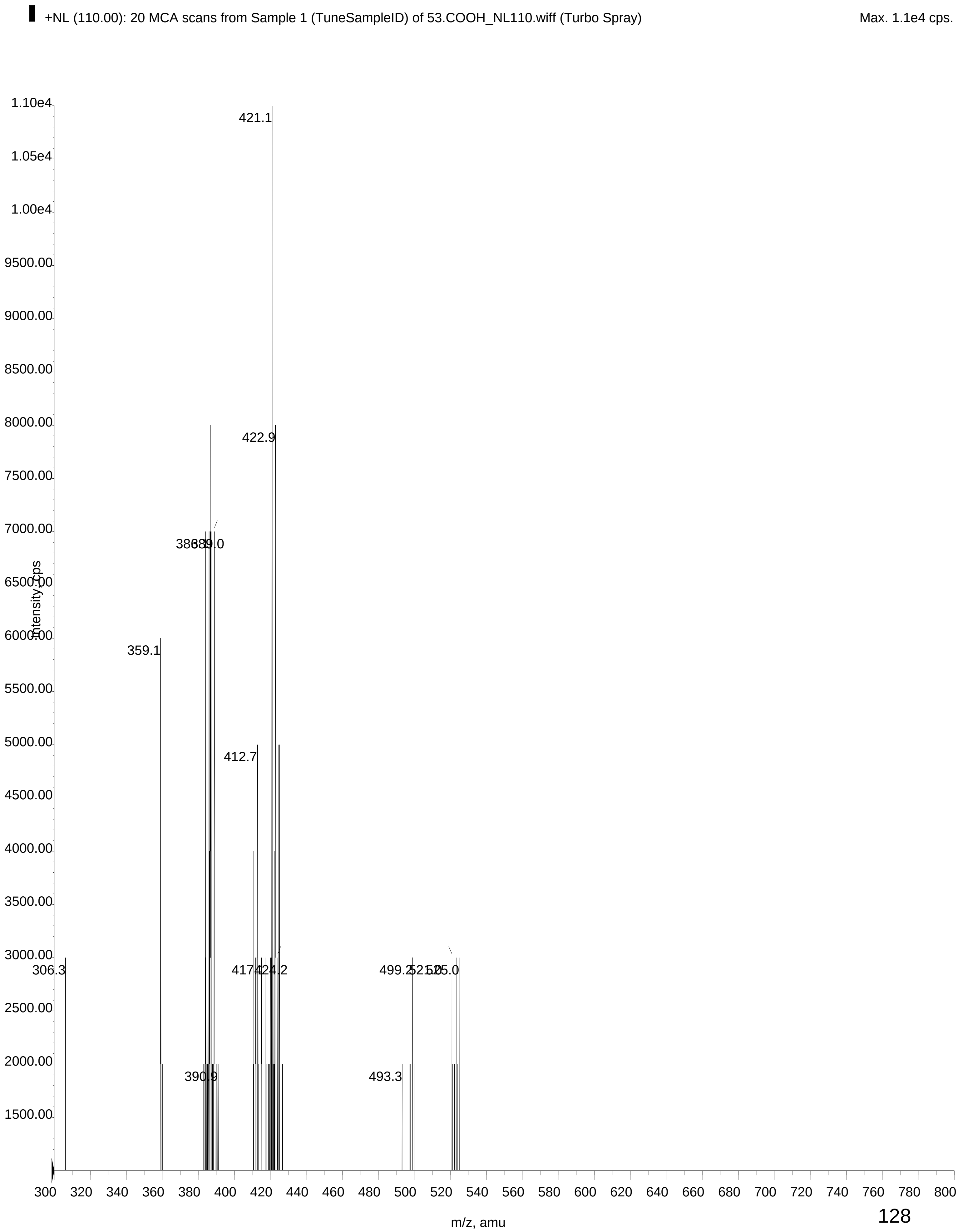

+NL (110.00): 20 MCA scans from Sample 1 (TuneSampleID) of 53.COOH_NL110.wiff (Turbo Spray)
Max. 1.1e4 cps.
421.1
422.9
388.9
389.0
359.1
412.7
306.3
417.4
424.2
499.2
521.0
525.0
390.9
493.3
Intensity, cps
m/z, amu

+NL (112.00): 20 MCA scans from Sample 1 (TuneSampleID) of 5.CN_NL112.wiff (Turbo Spray) Max. 7001.4 cps.

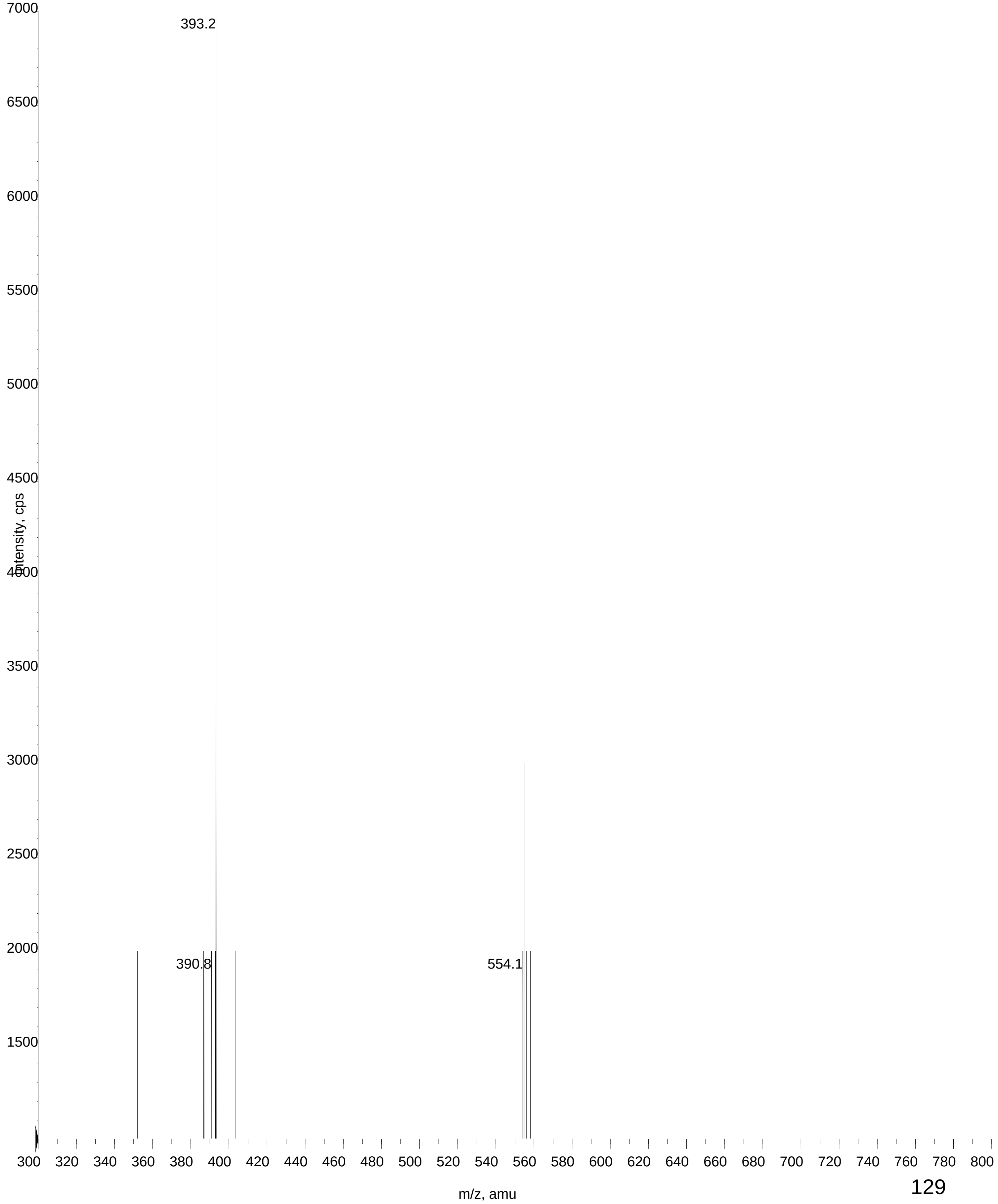

+NL (110.00): 14 MCA scans from Sample 1 (TuneSampleID) of 66.UL_NL110.wiff (Turbo Spray) Max. 1.2e4 cps.

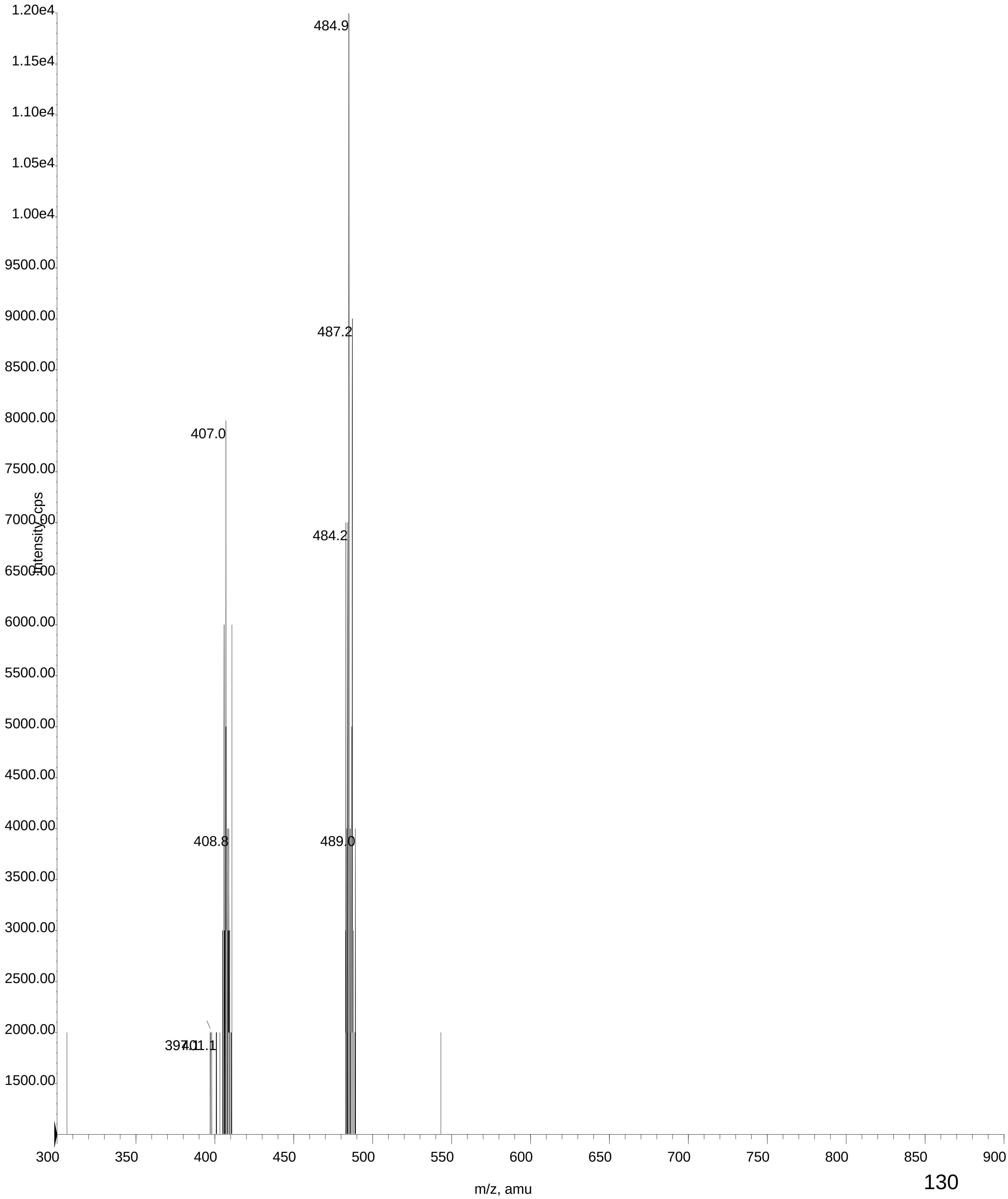

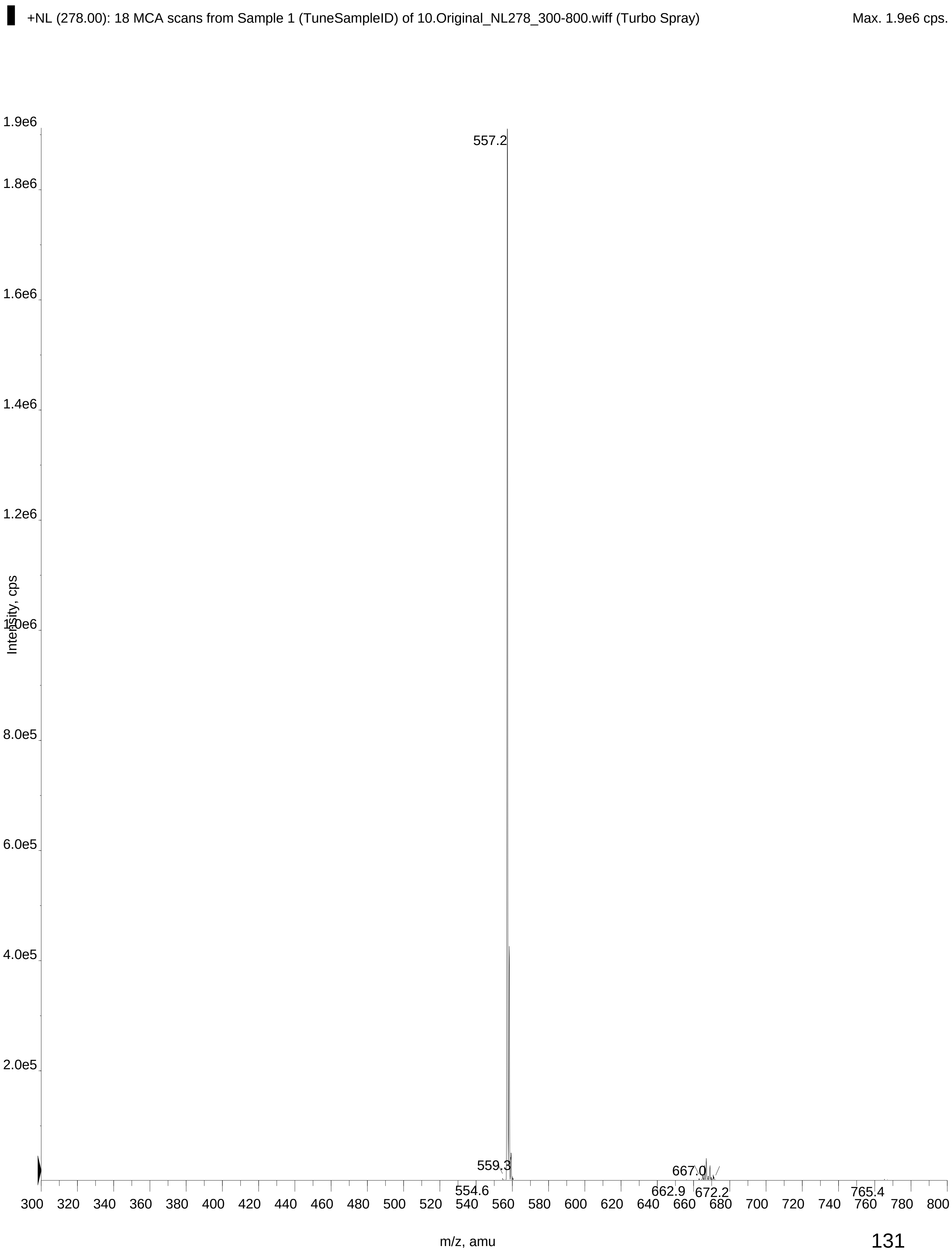

+NL (278.00): 18 MCA scans from Sample 1 (TuneSampleID) of 10.Original_NL278_300-800.wiff (Turbo Spray)
Max. 1.9e6 cps.
Intensity, cps
m/z, amu
557.2
559.3
554.6
667.0
662.9
672.2
765.4

+NL (248.00): 18 MCA scans from Sample 1 (TuneSampleID) of 58.COOH_NL248_300-800.wiff (Turbo Spray) Max. 1.1e6 cps.

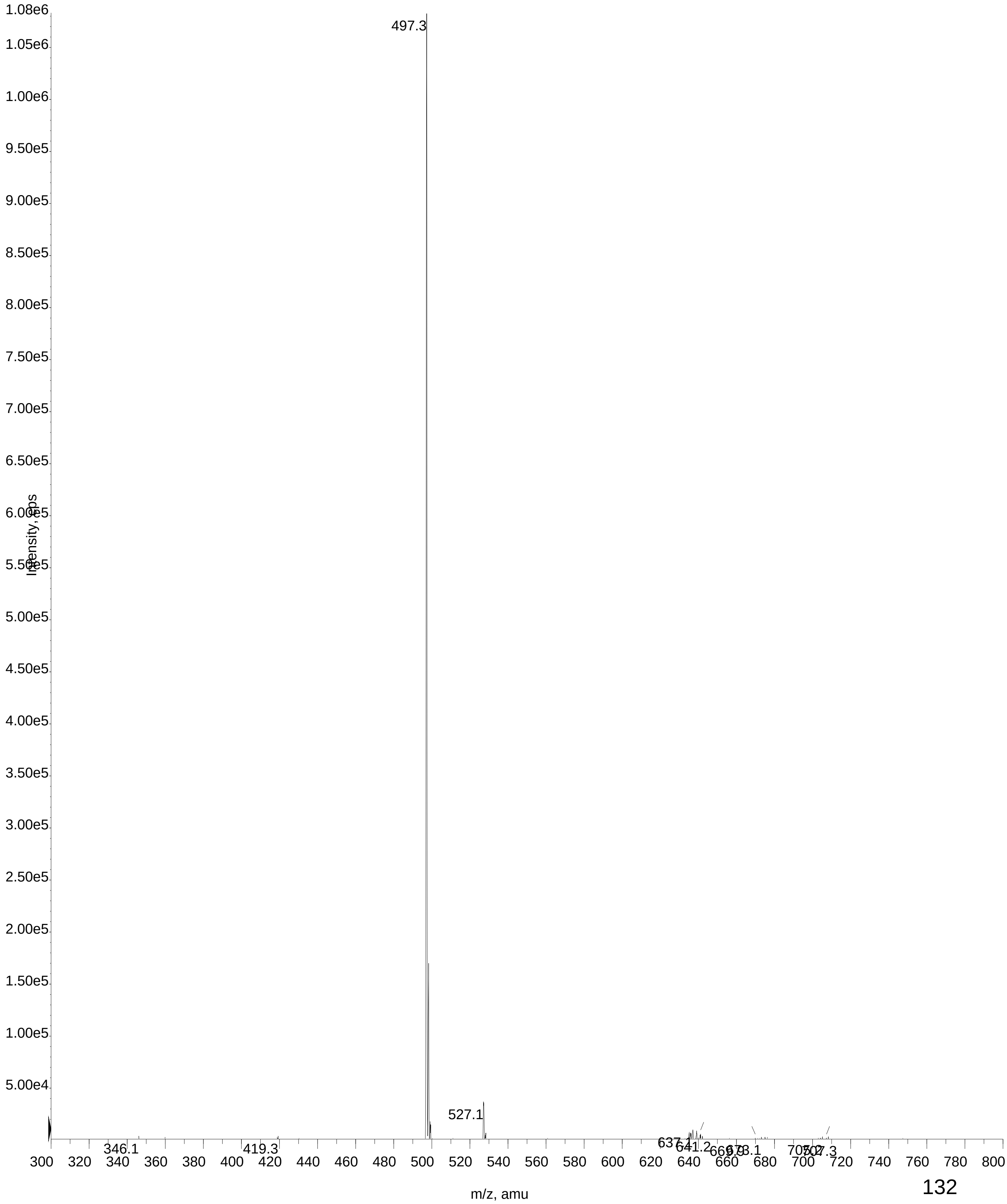

+NL (280.00): 20 MCA scans from Sample 1 (TuneSampleID) of 4.CN_NL280.wiff (Turbo Spray) Max. 2.7e6 cps.

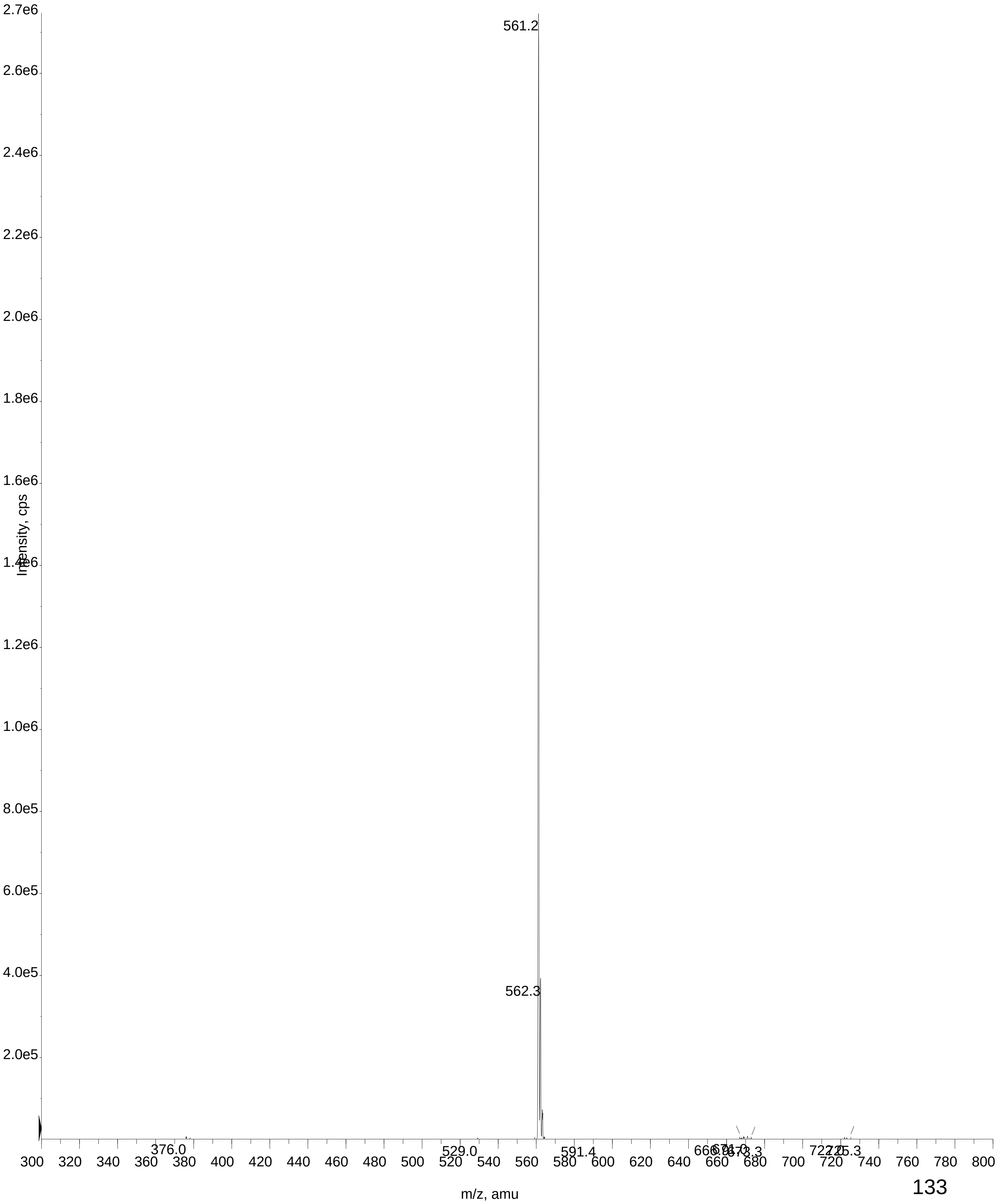

+NL (278.00): 18 MCA scans from Sample 1 (TuneSampleID) of 67.UL_NL278.wiff (Turbo Spray) Max. 2.2e4 cps.

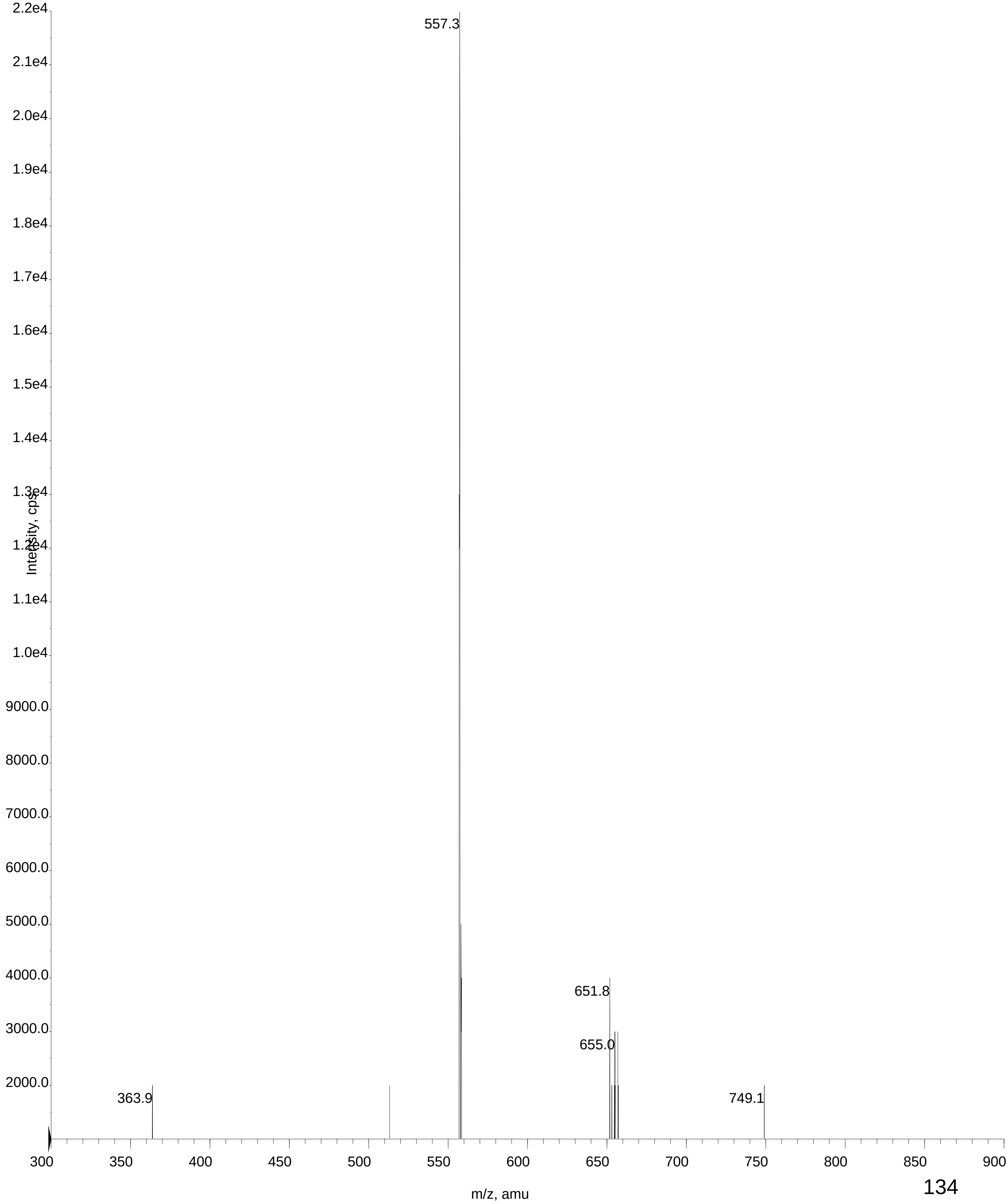

Supplement: Supplementary file 1 [file chem0019-13803-sd1.pdf]
